# Two novel PIWI families: roles in inter-genomic conflicts in bacteria and Mediator-dependent modulation of transcription in eukaryotes

**DOI:** 10.1186/1745-6150-8-13

**Published:** 2013-06-08

**Authors:** A Maxwell Burroughs, Lakshminarayan M Iyer, L Aravind

**Affiliations:** 1 National Center for Biotechnology Information, National Library of Medicine, National Institutes of Health, Bethesda, MD 20894, USA

## Abstract

**Background:**

The PIWI module, found in the PIWI/AGO superfamily of proteins, is a critical component of several cellular pathways including germline maintenance, chromatin organization, regulation of splicing, RNA interference, and virus suppression. It binds a guide strand which helps it target complementary nucleic strands.

**Results:**

Here we report the discovery of two divergent, novel families of PIWI modules, the first such to be described since the initial discovery of the PIWI/AGO superfamily over a decade ago. Both families display conservation patterns consistent with the binding of oligonucleotide guide strands. The first family is bacterial in distribution and is typically encoded by a distinctive three-gene operon alongside genes for a restriction endonuclease fold enzyme and a helicase of the DinG family. The second family is found only in eukaryotes. It is the core conserved module of the Med13 protein, a subunit of the CDK8 subcomplex of the transcription regulatory Mediator complex.

**Conclusions:**

Based on the presence of the DinG family helicase, which specifically acts on R-loops, we infer that the first family of PIWI modules is part of a novel RNA-dependent restriction system which could target invasive DNA from phages, plasmids or conjugative transposons. It is predicted to facilitate restriction of actively transcribed invading DNA by utilizing RNA guides. The PIWI family found in the eukaryotic Med13 proteins throws new light on the regulatory switch through which the CDK8 subcomplex modulates transcription at Mediator-bound promoters of highly transcribed genes. We propose that this involves recognition of small RNAs by the PIWI module in Med13 resulting in a conformational switch that propagates through the Mediator complex.

**Reviewers:**

This article was reviewed by Sandor Pongor, Frank Eisenhaber and Balaji Santhanam.

## Background

The PIWI module, found in the PIWI/AGO superfamily of proteins, is a common functional denominator for a wide range of biological processes in eukaryotes. These include, but are not limited to, germline maintenance [1], post-transcriptional gene silencing/RNA interference (RNAi) [2], chromatin dynamics, regulation of transcription [3,4], regulation of alternative splicing [5], DNA elimination in ciliates [6,7] and suppression of viral infection [8]. It acts by binding a double-stranded RNA duplex, typically consisting of a targeting RNA strand, referred to as the “guide strand”, and the targeted RNA strand complementary to the guide strand. Binding of the guide strand to the target strand results in either the silencing of specific RNA transcripts, as in the case of transposon silencing during germline maintenance [1,7] and mRNA silencing during RNAi [2], or is thought to localize crucial factors for regulating processes like transcription [3] and alternative splicing [5]. The PIWI module contains an RNase H fold domain with a conserved triad of residues required for nuclease activity that might participate both in processing the guide strand precursor as well as in cleaving target RNAs complementary to the guide strand [9-16]. On several independent occasions the PIWI module has lost the RNase H fold catalytic residues; these inactive versions are still capable of silencing activity by interfering with translation or facilitating degradation of guide strand-bound mRNAs by other nucleases [17].

While the PIWI/AGO superfamily was initially discovered in eukaryotes, orthologs were also identified in a wide range of prokaryotes spanning both the archaeal and bacterial superkingdoms [18,19]. Despite extensive characterization of these proteins in eukaryotes, the roles of the prokaryotic PIWI (pPIWI) proteins and the nature of their potential double-stranded nucleotide targets have remained murky. Recent analysis detected association with genes encoding several distinct, predicted nucleases, and a general preference for pPIWI genes to be localized in genomic neighborhoods containing genes belonging to known phage-defense systems. This led to a proposal advocating a role for pPIWI proteins as components of novel prokaryotic systems involved in defense against invasive mobile elements [20]. Earlier structural studies observed a tighter binding propensity for single-stranded DNA relative to single-stranded RNA guide strands in pPIWI proteins [21,22]. They also found, in stark contrast to the eukaryotic PIWI protein, the favored double-stranded substrate for the pPIWI domains to be a DNA-RNA hybrid. These observations suggested that pPIWI proteins might act on DNA-RNA hybrids.

Given recent increase in available genome data, we surveyed the complete scope of eukaryotic and prokaryotic PIWI domains to gain a better understanding of their relationship. Here we report the discovery of two distinctive PIWI families resulting from this survey; the first novel PIWI families to be discovered in well over ten years. One of these is a previously unrecognized bacterial family predicted to be a key component of a RNA-dependent restriction system. The second family is found in the eukaryotic Med13 protein, one of four protein components of the repressive CDK8 subcomplex of the multi-subunit, transcription regulatory Mediator complex. Identification of a PIWI module in Med13 generates a new testable hypothesis regarding the transcription modulatory role of the CDK8 subcomplex.

## Results and discussion

### Discovery of two novel PIWI families

**Figure 1:**
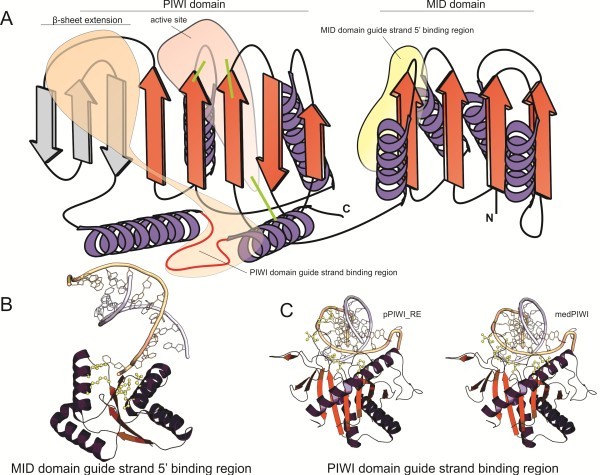
**Spatial conservation of active site and nucleotide-binding residues in the MID and PIWI domains.** (**A**) Topology diagram depicting the structural features and critical binding regions in the domains. MID and PIWI designations are provided at the top of the diagram. The β-sheet extension unique to the PIWI clade of the RNase H fold is labeled and shaded in grey. Locations of key active site residues are marked with green lines. Active site and general regions of nucleotide-binding are shaded and labeled. (**B** and **C**) Cartoon renderings of active site and nucleotide binding regions of a solved PIWI domain structure in complex with double-stranded nucleotide guide/passenger strands (PDB: 3HO1 [30]). Residues in the structure involved in guide strand binding with cognate conserved residues in pPIWI-RE and MedPIWI families are colored in yellow. The guide strand is colored in tan, passenger strand in light blue.

The PIWI module as presently defined in the Pfam database [23] consists of two distinct but functionally tightly coupled domains: an N-terminal three-layered α/β sandwich of the Rossmannoid type, with a four-stranded central β-sheet reminiscent of the TOPRIM domain and the β-sheet crossover occurring after the first β-strand [24] (see Figure 1A). This domain contributes crucial residues that bind the 5′ end of the small RNA guide strand [21,22,25-30]. The second domain is the core RNase H domain, which contributes additional, critical residues for guide strand-binding and when preserving the nuclease active site also cleaves the target strand. Prior structural studies on the PIWI module have labeled these two domains as the “MID” and “PIWI” domains, respectively [9,31]; a convention we adopt henceforth.

We performed profile–profile comparisons using the HHpred program initiated with both single sequences and a HMM derived from a multiple alignment of complete PIWI modules as queries against the complete set of HMMs found in the Pfam and Interpro databases. Interestingly, we observed statistically significant relationships between the PIWI module and two distinct protein families defined by the models “domain of unknown function” DUF3893 and Med13_C (corresponding to a conserved region in the eukaryotic Mediator complex Med13 proteins) from the Pfam database. For instance, a search initiated with a pPIWI module from *Mycobacterium* sp. KMS (gi: 119855142) recovers the DUF3893 profile with p-value = 7×10^-6^; 94% probability and the Med13_C profile with p-value = 3.4×10^-4^; 90% probability. To further investigate this relationship, we systematically collected all proteins corresponding to the DUF3893 and Med13_C models using iterative PSI-BLAST searches. The DUF3893-containing proteins were sporadically distributed across a wide range of bacterial lineages including firmicutes, actinobacteria, α/β/γ-proteobacteria, cyanobacteria, and chloroflexi. The Med13 proteins are widely distributed across eukaryotes including most plants, fungi, animals, slime molds, and stramenopiles as well as basal eukaryotes such as the parabasalid *Trichomonas vaginalis* and the heterolobosean *Naegleria gruberi* (see Additional file 1). In certain lineages additional Med13 paralogs were identified, including those resulting from a duplication event that occurred early in vertebrates [32].

We then constructed multiple sequence alignments of the proteins matching these modules, used them to predict secondary structure, and checked for congruence with existing structures of PIWI modules to determine the precise boundaries of the MID and PIWI domains. This showed that the DUF3893 and Med13_C models currently present in Pfam imprecisely define the domain architectures and boundaries within these proteins, notably excluding regions from both the MID and PIWI domains. Accordingly, we emended the domain boundaries of the DUF3893 and Med13_C models to completely match the predicted structural elements of the two constituent domains (see Figure 1A). Reciprocal HHpred searches initiated with both single sequences and HMMs derived from the above alignments against a database of HMMs constructed from multiple alignments built using Protein Data Bank (PDB) chains as seeds confirmed relationships with the PIWI domain: an emended representative version of the module matching Pfam DUF3893 (gi: 228927677 from *Bacillus thuringiensis*) recovers the PIWI module from *Archaeoglobus fulgidus*, PDB: 2W42, p-value = 6.7×10^-5^, probability 90%). Iterative sequence searches with PSI-BLAST further confirmed this relationship: e.g. a search with an emended representative of the module matching Pfam DUF3893 (gi: 269125748 from *Thermomonospora curvatae)* recovers a classical pPIWI domain (gi: 295689105 from *Caulobacter segnis* with e-value = 9×10^-15^, iteration 4). Similarly, a representative of the emended Med13 module (gi: 393215315 from *Fomitiporia mediterranea)* recovers a classical pPIWI module from *Pyrococcus furiosus* in a HHpred search (PDB: 1U04, p-value = 2.1×10^-4^; probability 87%).

### Characterization of the novel bacterial PIWI family

#### Structural and architectural features

**Figure 2:**
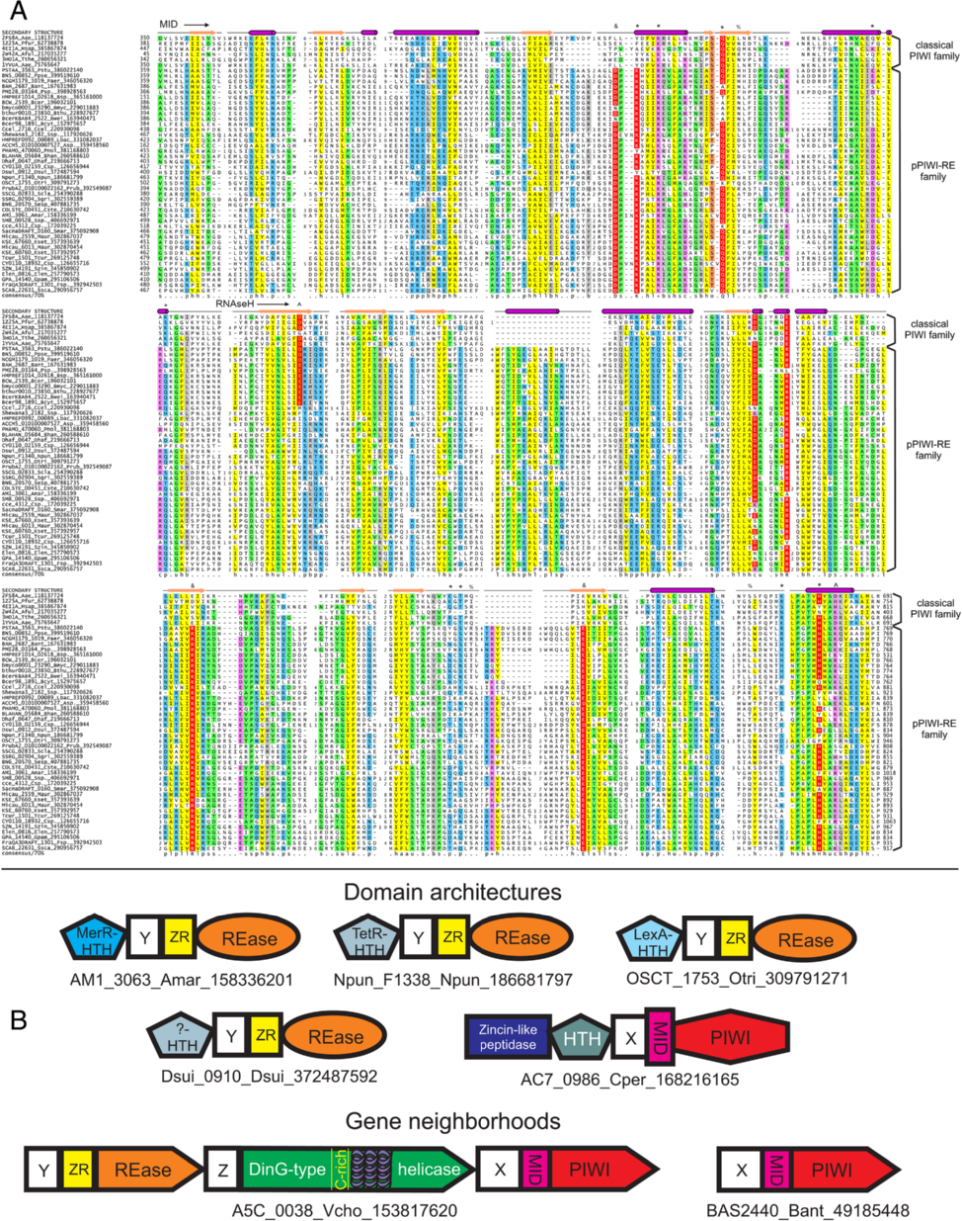
**Multiple sequence alignment and contextual information of the pPIWI-RE family.** (**A**) An alignment along with representatives of the classical PIWI module is shown. Regions of poor conservation are replaced with numbers representing the length of the excision. Consensus sequence is provided on the bottom line. Strongly-conserved residues are shaded red and colored white. Residues involved in catalytic RNase H activity are shaded red and colored yellow. Columns in alignment are color-coded based on conservation of shared chemical properties: yellow, hydrophobic/aliphatic (h/l); green, small/tiny (s/u); purple, charged (c/+/−); blue, polar (p); orange, hydroxyl group-containing (o); grey, large (b). Conserved residues involved in nucleotide binding across classical and pPIWI-RE PIWI modules or residues contributing to nuclease activity are denoted above columns by “*” and “^”, respectively. Predicted salt bridge-forming arginine and glutamate residues unique to pPIWI-RE are denoted by “&”. Residues conserved in classical PIWI modules but not in the pPIWI-RE module are denoted by “%”. Boundaries of the MID and PIWI domains are noted above the secondary structure. Sequences are labeled to the left of the alignment with gene name, organism abbreviation, and gene identifier number (gi number), demarcated by underscores. (**B**) Representative domain architectures and conserved gene neighborhoods involving the pPIWI-RE module. Genes within a conserved neighborhood are depicted as arrows with the arrowhead pointing 5′ to 3′. Labels below each architecture or neighborhood provide gene name, organism abbreviation, and gi number for a representative protein. Characteristic C-rich and helical regions of the DinG-type helicase are represented by yellow lettering and purple coils, respectively. Domain abbreviations: ZR, zinc ribbon; X, conserved globular region N-terminal to MID and pPIWI-RE domains; Y, conserved, largely α-helical domain with conserved arginine residue N-terminal to ZR and REase domains; Z, largely α-helical domain N-terminal to DinG-type helicase. Organism abbreviations in Additional file 1.

The above-identified bacterial family which overlaps with the Pfam DUF3893 model displayed two unique, absolutely conserved residues: an arginine and a glutamate (see Figure 2A). Hence, we refer to this family as the pPIWI-RE family (prokaryotic PIWI with conserved R and E residues). Secondary structure predictions indicated that the pPIWI-RE family is distinguished from all previously known PIWI domains by the presence of an additional α-helical element following the initial three-stranded beta-meander characteristic of the RNase H fold (see Figures 1A, 2A). We mapped all strongly-conserved residues found in the pPIWI-RE family onto available structures of classical PIWI modules and compared those positions to those required for RNase activity or nucleic acid binding in the latter modules (see Figures 1B-C, 2A). This showed that the conserved residues in the PIWI and MID domains of the pPIWI-RE family corresponded well to the positions known to be critical for nucleic acid-binding in the cognate domains of classical PIWI modules (see Figures 1, 2A). In particular, the conserved positions in the MID domain were all clustered in the cleft that specifically binds the 5′ end of the guide strand. This suggests that, like classical PIWI domains [33], the pPIWI-RE is likely to recognize small guide strands by anchoring them via the 5′ end. The arginine and glutamate characteristic of the pPIWI-RE family mapped to the β-sheet extension, which is unique to the PIWI-like clade (PIWI and Endonuclease V) of the RNase H fold (see Figures 1A, 2A). We predict that these two residues form a salt bridge across this β-sheet, which probably stabilizes its tertiary structure, and maintains a conformation specific to this family that is required to recognize the guide strand. The RNase catalytic residues are retained only in a subset of the pPIWI-RE family, suggesting that similar to the classical PIWI family they include both active and inactive versions.

The classical PIWI modules are typically fused to several N-terminal RNA-binding domains. In eukaryotic PIWI proteins, in order from the N-terminus, these include the so-called “N-term” domain implicated in unwinding of the double-stranded guide and passenger strands and also guide-target duplexes [34] and the single-stranded RNA-binding PAZ domain which interacts with 3′ ends of guide strands. Certain classical PIWI family proteins from kinetoplastids show an OB fold domain instead of the “N-term” domain. Previously studied prokaryotic PIWI proteins display a distinct architecture: in lieu of a PAZ domain they feature the so-called APAZ (Analogous to PAZ) domain suggesting analogous functions for the two domains [20]. Additionally, few pPIWI domains may contain extreme N-terminal fusions to predicted Sir2-domains [20]. The large N-terminal region of the pPIWI-RE family contains a distinct, conserved globular domain that partly overlaps with the Pfam DUF3962 model. Secondary structure predictions indicate that it is likely to adopt a β-strand-rich fold. It neither showed strong congruence with the secondary structural elements of the PAZ or APAZ domain nor did it display the well-conserved sequence motifs characteristic of the PAZ or APAZ domains (see Additional file 1). Furthermore, profile-profile searches did not point to any relationship between the N-terminal region of the pPIWI-RE family and these domains. Hence, this N-terminal region is likely to contain at least one distinct globular domain, which might nevertheless function analogously to the N-terminal domains in the classical PIWI proteins in mediating additional nucleic acid contacts (see Figure 2B).

#### Contextual associations of the pPIWI-RE module

Given the value of contextual information in gleaning insight into the functions of genes [35,36], we systematically collected conserved gene neighborhoods and domain fusions for the pPIWI-RE domains. Consequently, we observed two distinct genomic contexts for the pPIWI-RE genes with mutually exclusive phyletic patterns (see Figure 2B): (1) occurrence as a standalone gene (restricted to several *Bacillus* species, proteobacteria *Magnetospirillum gryphiswaldense*, *Pseudomonas putida* and *Azotobacter vinelandii*, and actinobacteria from the genera *Streptomyces* and *Thermomonospora;* Additional file 1). On rare occasions, this version of the pPIWI-RE module might occur fused to an N-terminal Zincin-like metallopeptidase domain. (2) Occurrence as part of a widely distributed three-gene neighborhood. Of the two genes that co-occur with the pPIWI-RE gene we found the first to encode a protein with a conserved restriction endonuclease (REase) fold domain by using profile-profile comparisons with the HHpred program (probability 94% using gi: 158336201 from *Acaryochloris* as a query). These proteins also contain a helical domain with a conserved arginine and Zinc ribbon (ZnR) domain at the N-terminus of the REase domain (see Figure 2B). Moreover, on at least four different occasions these proteins have also acquired further N-terminal HTH domains belonging to the LexA, TetR, MerR and a previously uncharacterized clade [37] (see Figure 2B). The second gene codes for a Superfamily II (SF-II) DNA helicase. Within SF-II it can be confidently assigned to the DinG-like clade on the basis of two unique structural features that typify them: namely, an iron-binding cysteine-rich region found after strand-2 of the helicase domain [38,39] and a large helical region inserted between conserved helix-4 and strand-5 which precede the C-terminal P-loop NTPase fold repeat unit characteristic of helicases [40,41]. The former domain apparently acts as an intracellular sensor of redox potential to regulate activity of the DinG helicase domains [42]. The gene order within this triad is strictly conserved with the REase gene coming first followed by the DinG SF-II helicase and pPIWI-RE genes (see Figure 2B and Additional file 1). Furthermore, the three genes have either overlapping or very closely spaced termini suggesting that they are transcribed as a single polycistronic message.

#### Functional implications of pPIWI-RE coding systems: A novel RNA-dependent restriction system

The widespread but patchy distribution of the above-described pPIWI-RE containing gene-triads across numerous phylogenetically distant bacteria (Additional file 1) is consistent with this system being disseminated by horizontal gene transfer (HGT). This pattern is reminiscent of bacteriophage restriction systems that confer a selective advantage on recipients due to their role in countering bacteriophage infections [43]. The presence of a gene coding for an REase protein without an associated methylase gene in the pPIWI-RE containing gene-triads is reminiscent of restriction systems such as the Mcr systems that target modified invading DNA [44]. The fact that the REase gene is always the first gene in the operon implies that it would be made before any of the other products and be available to cleave DNA. Hence, like the REases from the Mcr systems, it should have some means of specifically targeting non-self DNA rather than suicidally cleaving the cellular genome upon production. DinG serves as a helicase partner for multiple nuclease domains such as the RNase T-like and RNase D-like nuclease domains (both of which belong the RNAse H fold) [45-47]. Hence, it could function as a helicase partner for either the REase or pPIWI-RE or both. Given that these gene triads are parallel to type I and type III restriction-modification (R-M) systems in that they combine REase with helicase genes [48,49], it is conceivable that the DinG helicase plays a role comparable to the helicases that translocate the target DNA in those R-M systems. However, recent studies on DinG-like helicases, which show that it acts on RNA-DNA duplexes *in vitro*[50] and R-loops (bubble-like structures forming via displacement of one strand of a DNA double helix by a complementary RNA strand [51]) *in vivo*[52], point to further functional complexities. DinG-like helicases are specifically involved in unwinding of R-loops during replication across active transcriptional units [52]. Interestingly, DinG-like helicases have also been found to be components of Type-U CRISPR/Cas systems [53], supporting their action in the context of DNA-RNA hybrid duplexes.

**Figure 3:**
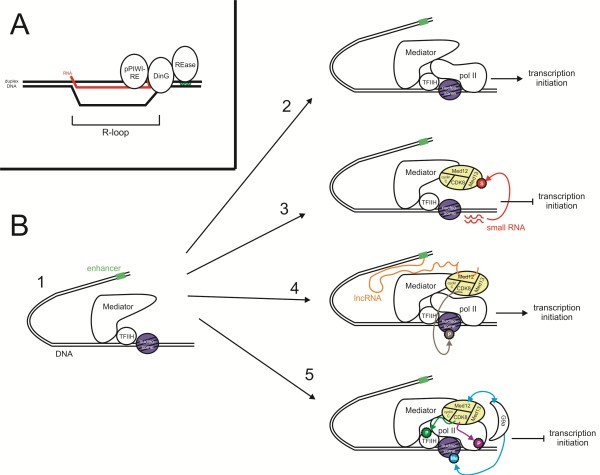
**Schematic representation of predicted functions of the pPIWI-RE and MedPIWI domains.** (**A**) pPIWI-RE domain associates with DNA-RNA hybrid structure present during R-loop formation in an invasive DNA element, resulting in recruitment of the DinG helicase and endoDNAse REase domains. (**B**) Regulation of the core Mediator complex via the CDK8 subcomplex is depicted, beginning at left with 1) simplified representation of the PIC, poised for initiation of transcription. 2) In absence of CDK8 subcomplex, the core Mediator complex recruits pol II and transcription is initiated. 3) Kinase activity-independent repression of transcription: the CDK8 subcomplex (depicted as yellow oval) transiently associates with core Mediator complexes across the genome [62]; availability of a small RNA binding substrate for the MedPIWI domain in the Med13 component of the CDK8 subcomplex triggers a shift from transient association to repressive role of CDK8 subcomplex, triggering a conformational switch in the Mediator-CDK8 combined complex which blocks pol II re-association. 4) lncRNA-mediated transcriptional activation: association of Med12 with activating lncRNA transcribed and looping from distal enhancer element (depicted as box colored in green) facilitates CDK8 kinase-mediated phosphorylation of transcriptional-activating histone H3 serine 10, resulting in association of pol II and transcriptional activation [80]. 5) Additional layers of CDK8 subcomplex-mediated transcriptional repression: CDK8 kinase phosphorylates TFIIH [68] or C-terminal tail of pol II [67] and Med12-mediated recruitment of SET domain methyltransferase (G9a) methylates histone H3 lysine 9 [71], all resulting in repression of transcription. Abbreviations: P, phosphorylation event; Me, methylation event; S, switch resulting in conformational change.

Taken together, these observations allow us to propose a model that can account for the most likely activities of all three products of these gene triads (see Figure 3A). On the basis of the DinG helicase we posit that the initiating signal recognized by these systems is likely to be a DNA-RNA hybrid structure. These are known to primarily form during transcription and replication of phages [54] or plasmids [55,56] and relatively infrequently during transcription of the endogenous genome [51]. Therefore, specifically targeting these structures could provide an effective means of restricting transcriptionally active and replicating invasive genomes and their transcripts. In this system the pPIWI-RE module is likely to be deployed as a sensor for the DNA-RNA hybrid, in a manner comparable to the classical pPIWI domain for which there is accumulating evidence for preferential binding to DNA/RNA hybrids [20,22,29]. The catalytically active pPIWI-RE modules might additionally cleave the RNA strand of such hybrid duplexes. Recognition of the DNA-RNA hybrid by the pPIWI-RE module is likely to recruit the DinG helicase for the unwinding and/or the translocation of R-loops, which could further provide a suitable dsDNA substrate for cleavage by the REase domain. Importantly, this hypothesis of DNA-RNA hybrid-directed restriction can explain why the REase protein, which is the first to be transcribed and translated, is unlikely to act on self DNA upon its production. The diverse HTH domains, which are occasionally fused to the N-termini of the REase proteins, could either function as autoregulators of transcription of the gene triad or in providing sequence specificity during restriction.

In the case of pPIWI-RE genes occurring independently of the above-described three gene restriction system we found no evidence for the presence of related REase or DinG genes in the same genomes. A simple interpretation would be that these pPIWI-RE modules function similarly to the aforementioned versions, but instead of recruiting restriction machinery they function by themselves. It is possible in these cases they modulate gene expression by cleaving transcripts, physically interfering with transcription (an echo of the action of eukaryotic PIWI proteins), or blocking the release of transcripts from the template DNA [3,57].

### The PIWI module in eukaryotic Med13

#### Structural and architectural features of the MedPIWI module

**Figure 4:**
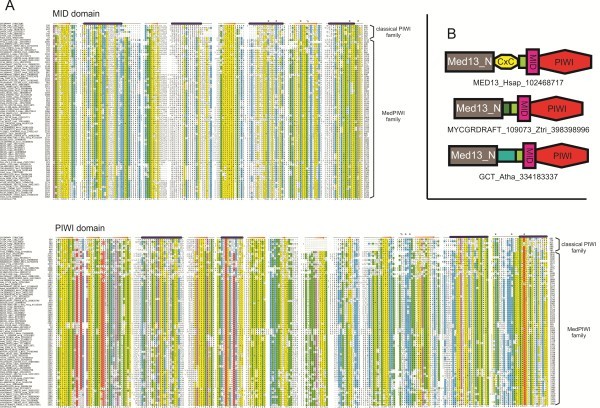
**Multiple sequence alignment and domain architectures of the MedPIWI family.** (**A**) Multiple sequence alignment with representatives of the classical PIWI module is shown. Organization, numbering, labeling, consensus abbreviations, and coloring of the alignment are as described in the legend to Figure 2. Conserved residues involved in nucleotide binding across both the classical and MedPIWI modules are denoted above the appropriate column in the alignment by “*”. Residues which may be conserved in classical PIWI modules but not in the MedPIWI module are denoted by “%”. (**B**) Representative domain architectures of the MedPIWI module. The small green box immediately upstream of the MID domain represents the conserved, small “linker” domain. Other unlabeled domains represent potential lineage-specific domains while C×C refers to the animal-specific, potential zinc-binding domain (see Additional file 1). Organism abbreviations may be found in Additional file 1.

Given the presence of this PIWI module in the Med13 subunit of the Mediator complex, we hereafter refer to it as the MedPIWI module. An inspection of the multiple sequence alignment of the novel eukaryotic family revealed extensive conservation at the positions crucial for nucleic acid-binding in the classical PIWI module including residues interacting with the 5′ end of the guide strand in the MID domain (see Figures 1, 4A). However, this family shows certain distinctive features: 1) absence of the first catalytic aspartate/glutamate found near the C-terminus of strand 1 of the RNase H fold’s core β-sheet. 2) The second conserved residue of the catalytic triad, located at the C-terminus of strand-4 of the RNase H fold, is absent with no identifiable compensatory residues. 3) Another charged residue contributing directly to the active site from the C-terminal segment of the final helix of the RNase H fold is also absent (see Figure 4A). 4) Its RNase H fold shows a reasonably well-conserved aspartate in the loop between strand-1 and strand-2, which is suitably positioned to contact the bound nucleic acid, based on comparisons to classical PIWI domains [58]. 5) The MedPIWI RNase H fold also shows a near-absolutely conserved aspartate at the C-terminus of strand 2 (see Figure 4A) that is unlikely to have any role in nucleic acid substrate recognition. Taken together, these observations suggest that none of the MedPIWI modules might be catalytically active. However, they are likely to bind double-stranded nucleic acid substrates, just as the classical PIWI modules.

The MedPIWI modules are distinguished from all other PIWI modules by the presence of extensive disordered regions, often occurring as lineage-specific inserts within both the MID and PIWI domains and also in between the two (indicated by numbers in Figure 4A). This family is also distinguished by a small domain consisting of a predicted beta-hairpin followed by a single alpha-helix located immediately N-terminal to the MID domain and might be compared to the small “linker” domains observed in classic PIWI families [20]. Beyond this domain is the Med13-N module corresponding to the Pfam model Med13_N (see Figure 4B). The conserved core of this region is predicted to adopt an α + β structure with a prominent stretch of 6–7 contiguous β strands which could adopt a barrel or sandwich-like fold (Additional file 1). This module is present in all eukaryotic Med13s except those from *Entamoebidae*, where it appears to have been displaced or has degenerated*.* Thus, the Med13-N module was likely associated with the MedPIWI even in the stem eukaryotes, and is comparable in its location, though not necessarily in function, to the N-terminal domains, such as PAZ, APAZ and that found in the pPIWI-RE family (see above). Some additional lineage-specific globular domains might be present along with an extensive disordered region in the linker connecting the Med13-N module to the rest of the protein. These include a potential Zn-binding domain with two CxC motifs (where “C” is a cysteine residue and “x” is any residue) in animals and other unrelated modules in plants and fungi (see Figure 4B, Additional file 1). The size and frequency of the lineage-specific inserts and disordered regions roughly corresponds to the total number of units comprising the Mediator complex in a given lineage [32]. Thus, they might represent secondary adaptations for increased inter-subunit contacts within the Mediator complex.

#### Partners and physical interactions of Med13: functional implications for the MedPIWI module in eukaryotic transcription regulation

The Mediator complex, along with several basal or general transcription factors, is part of the Preinitiation Complex (PIC), which is needed for transcription at promoters of genes transcribed by RNA polymerase II (pol II) in eukaryotes [59,60]. The Mediator complex has two basic forms (see Figure 3B): 1) the core Mediator complex, which is a strong transcriptional coactivator [61] and occupies promoters across the genome [62,63] and 2) the Mediator-CDK8 complex, which usually has a negative regulatory role and while found to transiently associate across all promoters, associates strongly with only a subset of genes that typically show higher expression levels [62-66]. The latter complex is characterized by the addition of a four subunit subcomplex, CDK8, which, in addition to the MedPIWI-containing Med13, also contains Med12, cyclin C, and the CDK8 kinase. Negative regulation by the CDK8 subcomplex appears to utilize multiple independent, but apparently synergistic, actions of its distinct subunits (see Figure 3B). The cyclin/kinase pair of the subcomplex phosphorylates the pol II C-terminal tail disrupting the association between pol II and the core Mediator complex [67]. It might also phosphorylate cyclin H in the TFIIH complex and inhibit activation of translation transcription by the latter complex [68]. However, previous studies have shown that negative regulation of transcription by the CDK8 subcomplex also occurs independently of the CDK8 kinase activity: the interaction between the CDK8 subcomplex and the core Mediator acts as a modulatory “switch” that allosterically affects the core Mediator-pol II interaction [69,70] and determines the shift between transient and stable CDK8 subcomplex promoter occupancy. This switch is believed to be dependent on Med12 and Med13 [70,71], although the exact mode of their action remains murky. In this regard, recent studies utilizing an *in vitro* chromatin-based transcriptional system demonstrated that Med13 is critical for physically linking the CDK8 subcomplex to the core Mediator complex and is specifically required to repress previously activated promoters by barring re-association of a pol II enzyme with the PIC [70].

Given these studies our discovery of a PIWI module in Med13 provides a previously unexplored vista to investigate the mechanism of transcriptional modulation by the CDK8 subcomplex (see Figure 3B). As the MedPIWI module displays the conserved features related to binding double stranded substrates (see above, Figures 1B-C, 4A), we posit that this activity is central to the molecular switch that modulates the core Mediator-pol II interactions. We predict two plausible candidates for the substrate oligonucleotide bound by the MedPIWI modules that are consistent with published laboratory studies: 1) it is conceivable that the MedPIWI module retained the ancestral ability to bind DNA-RNA hybrid duplexes, a feature that the ancestral eukaryotic PIWI modules would have presumably possessed when they were acquired from the prokaryotic progenitors. DNA-small RNA hybrids could form close to the transcription start site (TSS) from the small RNA byproducts of polymerase stalling or backtracking [72,73]. Indeed, such small transcripts have been detected (commonly referred to as TSSa [74] or tiRNA transcripts [75]) in several global deep-sequencing datasets across a range of animal species [76] and even in association with classical PIWI domains [77]. These could either re-associate with DNA opened as part of the transcriptional bubble formed during re-initiation events or remain associated with open DNA in the wake of repeated pol II passages. This proposal has the attractive feature of explaining the preferential association of Med13 with highly transcribed genes [62-66,70] because such genes are known to be enriched in small TSS-associated transcripts [75], thereby increasing the chances of formation of DNA-RNA hybrids substrates for the MedPIWI module. The observation that the CDK8 subcomplex association occurs only after initiation of at least a single round of transcription by pol II following PIC assembly [70] also suggests that its association might require the availability of previously-transcribed RNA byproducts. Another potential source for small RNAs that could form DNA-RNA hybrids is the small processed antisense transcripts that have been found to be associated with the promoter sites of transcriptionally active genes [3]. 2) Alternatively, like most characterized eukaryotic PIWI modules, the MedPIWI module might bind dsRNA substrates. In this case its action can be compared to the classical eukaryotic PIWI protein AGO2, which has been shown to regulate the positioning of pol II while binding sense-antisense RNA duplexes derived from transcriptionally active genes [3]. Interestingly, these antisense small RNA-AGO2 complexes increase in abundance concomitant with transcriptional activation upon stimuli such as heat shock [3]. It is possible that the MedPIWI module acts in a comparable manner to associate with such promoter-derived small RNAs that could form dsRNA duplexes during active transcription.

In conclusion, we hypothesize that the modulatory switch mediated by the CDK8 subcomplex probably depends on the ability of the MedPIWI module to recognize small transcripts associated with active promoters that form either DNA-RNA or dsRNA duplexes. This binding induces a conformational change that propagates through the rest of the complex to allosterically impact the interaction of the Mediator with pol II. Binding of duplexes by the MedPIWI module might also influence the deployment of the additional layers of control that depend on the CDK8 subcomplex, such as the activity of the CDK8 kinase [67,68] and Med12-mediated histone H3K9 SET domain methyltransferase (G9a) recruitment [71] (see Figure 3B). Intriguingly, in a small number of cases, association of the CDK8 subcomplex with the core Mediator results in Med13- and Med12- dependent transcriptional activation rather than repression [78,79]. While this manuscript was under review, a study was published demonstrating the role of enhancer-associated long non-coding RNAs (lncRNAs) in facilitating this process of activation of transcription by the CDK8 subcomplex along with the core Mediator [80] (see Figure 3B). It was demonstrated that in animals these activating lncRNAs interact with the Med12 subunit of the CDK8 complex and cause it to catalyze Histone H3 serine 10 phosphorylation rather than the above-mentioned negative regulatory phosphorylations of Cyclin H and the RNA polymerase C-terminal tail. H3S10 phosphorylation has a positive regulatory role probably by inhibiting the repressive H3K9 methylation among other actions. We suspect that interaction with these enhancer-derived lncRNAs is unlikely to be the primary function of the MedPIWI module because it is conserved across eukaryotes and appears to be required for actions of the CDK8 complex beyond activated transcription. However, we cannot rule out that the lncRNA might interact with processed small RNAs to form duplexes that might be recognized by the MedPIWI module to regulate transcription in certain conditions.

### Evolutionary considerations

The new PIWI families reported here also offer an opportunity to reassess the natural history of the PIWI/AGO superfamily. The pPIWI-RE family shows a relatively smaller spread across the prokaryotic tree (see Additional file 1) compared to the classical pPIWI proteins [20]. Hence, it is possible that pPIWI-RE descended from an RNase-active classical pPIWI module in bacteria and was subsequently dispersed to diverse lineages via HGT. The multiple independent losses of the RNase H fold catalytic residues in the pPIWI-RE family are comparable to the classical PIWI modules [20]. Thus, not just active processing of RNA, but also non-catalytic binding of duplexes containing RNA appears to have been widely used across the PIWI/AGO superfamily. Indeed, this function appears to have been the dominant theme in the case of the MedPIWI family. The phyletic patterns of Med13 are closely correlated with the three other subunits of the CDK8 complex. They are present in several basal eukaryotes and are widespread across the eukaryotic tree strongly supporting the presence of a complete CDK8 complex in the last eukaryotic common ancestor (LECA). Thus, the CDK8 subcomplex and an ancestral version of the core Mediator complex appear to have been in place by the LECA, suggesting that antagonistic regulatory interactions of these complexes was a feature of transcription regulation in the common ancestor of extant eukaryotes.

Earlier studies had indicated that at least one member of the classical PIWI family was already present in the LECA [81]. Prior to LECA, in the eukaryotic stem lineage, this PIWI protein appears to have undergone a duplication giving rise to a version with a dedicated role in transcription regulation and a second version primarily involved as a standalone protein in diverse processes involving small non-coding RNAs. The former version appears to have functionally associated with the other emerging subunits of the CDK8 complex with a corresponding rapid divergence in sequence. At least in the latter version there appears to have been a specificity shift towards dsRNA from the likely ancestral pPIWI preference for binding DNA/RNA hybrid duplexes [20,22,29]. The classical PIWI family is also widely conserved across archaea [19], suggesting that the stem eukaryotes could have possibly inherited the ancestral PIWI protein directly from their archaeal progenitor. Given the functional connections now known or inferred across the PIWI/AGO superfamily (each of the two families discussed here and the classical PIWI proteins) to regulation of transcription, it is conceivable that even in archaea (and possibly other prokaryotes) PIWI proteins function in transcription regulation, beyond the proposed role in defense against genomic parasites. If this were the case, then the two primary eukaryotic versions merely reflect partitioning of the ancestral roles into distinct proteins. Thus, our identification of a novel eukaryotic PIWI family could also have implications for the functions of the prokaryotic PIWI domains.

## Conclusions

The two novel families of PIWI modules described here are the first such discoveries since the initial characterization of the PIWI/Argonaute family in eukaryotes and their close prokaryotic counterparts over a decade ago [18,82,83]. While considerably divergent from these earlier-characterized versions, both families are predicted to bind double-stranded substrates based on the strong conservation of residues at positions corresponding to nucleic acid binding sites in the classical PIWI modules in both of the novel families (see Figures 1, 2, and 4). Moreover, their predicted functions fit within the spectrum of previously observed functional roles for different members of the PIWI superfamily. Thus, despite the considerable divergence from the classical PIWI family at the sequence level the new families appear to have maintained the characteristic ability of this clade of RNase H fold proteins to operate on RNA-containing duplexes. Nevertheless, the predicted functions of the two newly described families present some previously unobserved features. The pPIWI-RE family offers the first example for a potential RNA-dependent restriction system in prokaryotes that is distinct from the previously characterized CRISPR/Cas-type systems [53]. In particular it presents some parallels to the Type-II CRISPR/Cas systems which combine a RNase H fold nuclease with a HNH endoDNase that is also found in several restriction systems [53]. Thus, it emerges as the first clear example of a PIWI family member directing and coordinating a DNA- and RNA- based defensive response against genomic parasites in bacteria. This system could potentially be developed as a reagent to cleave target DNA using a RNA guide. Our prediction implicating the MedPIWI family in recognition of RNA-containing duplexes offers an entirely new mechanism for the action of the CDK8 subcomplex both in terms of the modulation of transcription at the promoters of highly expressed genes and providing the first delineation of the criterion underlying the transition from transient CDK8 subcomplex co-occupancy at sites of core Mediator occupancy to sustained CDK8 subcomplex association resulting in repressive activity [62] (see Figure 3B). This research also further fuels the broader emerging theme implicating ncRNAs in modulation of transcription at sites of initiation [3,80]. This hypothesis could be investigated via a combination of ChIP-seq experiments on CDK8 subcomplex members and MedPIWI module immunoprecipitation-sequencing.

## Methods

Iterative profile searches with the PSI-BLAST [84] and JACKHMMER [85] programs were used to retrieve homologous sequences in the protein non-redundant (NR) database at the National Center for Biotechnology Information (NCBI). For most searches a cut-off e-value of 0.01 was used to assess significance. In each iteration, the newly detected sequences that had e-values lower than the cut-off were examined for conserved motifs to detect potential homologs in the twilight zone. Similarity-based clustering was performed using the BLASTCLUST program (http://ftp.ncbi.nih.gov/blast/documents/blastclust.html) to cluster sequences at different thresholds. Multiple sequence alignments were built using the Kalign [86] and MUSCLE [87] programs, followed by manual adjustments based on profile–profile alignment, secondary structure prediction and structural alignments. Consensus secondary structures were predicted using the JPred program [88]. Remote sequence similarity searches were performed using profile-profile comparisons with the HHpred program [89]. Gene neighborhoods were extracted and analyzed using a custom PERL script that operates on the Genbank genome or whole genome shotgun files. The protein sequences of all neighbors were clustered using the BLASTCLUST program to identify related sequences in gene neighborhoods. Each cluster of homologous proteins was then assigned an annotation based on the domain architecture or shared conserved domain. A complete list of Genbank gene identifiers for proteins investigated in this study is provided in the Additional file 1. Structure similarity searches were conducted using the DALIlite program [90] and structural alignments were generated by means of the MUSTANG program [91].

## Reviewer’s comments

### Reviewer 1: prof. Sandor Pongor, International Centre for Genetic Engineering and biotechnology (ICGEB), Italy

The PIWI domain plays a role in dsRNA guided hydrolysis of ssRNA in a variety of cellular pathways involved in binding and cleaving of RNA. Ever since its discovery in the PIWI/ARGO superfamily, the PIWI module is being identified in a growing number of cellular pathways such as RNA interference, chromatin organization, germline maintenance, and was found to bind different classes of small noncoding RNAs that guide Argonaute proteins to their targets. Based on profile-profile comparisons, Burroughs and coworkers describe two new subfamilies of PIWI, both showing a residue conservation pattern characteristic of guide-strand binding but not those of catalytic activity. One of the subfamilies, PIWI-RE is found in bacteria, and the conservation is supported by similar chromosomal contexts which leads the authors to suggest that it plays a part in a novel RNA-dependent restriction system. The other subfamily, MedPIWI is found in the Med13 subunit of the Mediator complex in eukaryotes. MedPIWI shows distinctive residue conservation patterns that indicate an involvement in ds nucleic acid binding but no catalytic activity. The authors hypothesize that MedPIWI’s role may be an ssRNA-mediated activation of the conformational switch through which the CDK8 subcomplex modulates transcription at Mediator-bound promoters. Both subfamilies are widely distributed, PIWI-RE is found in firmicutes, actinobacteria, α/β/γ-proteobacteria, cyanobacteria, and chloroflexi. MedPIWI is found in plants, fungi, animals, slime molds, and stramenopiles as well as basal eukaryotes.

I find the analysis straightforward and highly convincing, and the conclusions, even though daring and imaginative, are well within the expected limits of scientific interpretation. The structure of the manuscript is logical, even though the description of two subfamilies within one article may somewhat divide the attention of the reader. In conclusion, I recommend publication of this manuscript without further changes.

Authors’ response: We appreciate the positive evaluation of our work. While the two disparate functional themes might indeed divide the reader’s attention, we sought to present it as one article to due to the common theme provided by the previously known functional features of the PIWI superfamily itself.

### Reviewer 2: Dr. Frank Eisenhaber, Bioinformatic Institute, Singapore

This work is a pretty nice continuation of the series of articles by Aravind et al. gene function hypotheses/discoveries are presented in the meticulous combination of sequence-analytic findings and hints from the experimental biological literature. Starting with the serendipitous observation of two PFAM domains with unknown functions showing some HHpred-derived similarity to the PIWI/AGO model, the authors show that two divergent subfamilies of PIWI/AGO in the bacterial world and among eukaryotes do exist. Lots of additional information with regard to 3D structural details, binding properties, domain evolution, etc. is derived with the classical sequence-analytic procedures and many of these conclusions can be validated experimentally.

Given the very nicely written main text, the summary reads like an unloved extra, apparently composed after the authors were tired from putting together text and figures. I suggest to go carefully through the text and complement the summary with all the detailed conclusions about the two new subfamilies.

Author’s response: We appreciate the positive evaluation of the work presented in this article. We have now revised the summary to better incorporate more of the conclusions reached in the text. Moreover, at the behest of a similar suggestion by Reviewer #3, we have added a figure that provides a one-stop pictorial summary for the predicted functional roles of the two families.

Further, the authors mention some “KM” who analyzed the data (in “Authors” contributions”); yet, this person is not listed among the authors.

Authors’ response: We have removed this inadvertently included initial from the contributions list.

### Reviewer #3: Dr Santhanam Balaji, MRC Laboratory of Molecular Biology, United Kingdom

Burroughs et al. report computational discovery of two novel families belonging to PIWI modules, first family (pPIWI) is sporadic in phyletic distribution and restricted to bacterial superkingdom, while second one (MedPIWI) is found only in eukaryotes. pPIWI is prominently encoded by operon that also contain genes that encode restriction endonuclease-like enzyme and a DinG helicase. Based on these observations, the authors propose that pPIWI is likely to act on genomic parasites such as invasive phages and selfish replication elements. MedPIWI which is also found as core conserved module of Med13, part of CDK8 subcomplex. CDK8 subcomplex is a known negative regulator of transcription. Identification of PIWI family in it suggests possible mode of action of CDK8 by targeting small RNAs in the vicinity of mediator complex bound promoters of highly transcribed genes. Hence, the discovery of MedPIWI sheds light on mechanism of transcription modulation mediated by CDK8 subcomplex. There are also detailed mechanistic models proposed by the authors for each of the two families. This work reports important discoveries that have potential wide implications from genomic conflicts in bacterial systems to transcription in eukaryotes. Discovery of PIWI family in Med13 is particularly interesting, this probably triggers wider intriguing question: are there many more (yet to be identified) RNA binding family hidden in Mediator complex subunits or associated proteins? I fully support the publication of this manuscript in Biology Direct.

Authors’ response: We appreciate the positive evaluation and detailed review of the work presented here. It is increasingly becoming clear that ncRNA binding plays a role in Mediator function. In light of this it is quite possible more RNA-interacting domains will be identified in the coming years as new structural studies on Mediator are published. However, given that the majority of Mediator complex components are rife with regions of low complexity sequence at this point other obvious RNA-binding domains remain difficult to detect. The Med8C/18/20 submodule has been shown to contain a version of the CYTH domain (LM Iyer, L Aravind BMC genomics 3 (1), 33; PMID: 12456267) which is also found in the mRNA triphosphatase. Whether these CYTH domains might have a role in RNA interaction remains unclear.

My specific points/comments:

1. *Did authors find any more detail (in terms of functions or interactions) about the α-helical element following the three-stranded β-meander of the RNAse H fold in pPIWI-RE?*

Authors’ response: This is certainly an interesting feature of the pPIWI-RE family: mapping of this helical elemenet to existing structures reveals that is could be positioned reasonably close to the nucleotide binding/catalytic active site. At the same time, it lacks any strongly conserved residues outside of a well-conserved tryptophan residue immediately N-terminal to the helix; hence, we refrain from any detailed functional speculation.

2. *The observation of Zincin-like metalloprotease fused with pPIWI-RE is interesting although not in many instances. Is it possible that in these cases the metalloprotease domain could directly aid pPIWI-RE to target RNAs that are securely logged in ribonucleoprotein complexes?*

Authors’ response: Given the infrequency of the fusion we are not sure if it is a gene annotation artifact of some kind; hence, we have not speculated in the manuscript on any concrete functional role. If this fusion were to be recovered in the future more genome sequences a role as suggested by the referee is not impossible.

3. *It appears a bit ironic that the CDK8 subcomplex is a negative regulator of transcription but is found at mediator complex bound promoters that correspond to highly transcribed genes. Does this mean CDK8 has a direct role through MedPIWI in determining overall level of the transcripts emerging from these regions?*

Author’s response: Yes, this is generally the idea we hoped to express which is consistent with the prevailing view of the CDK8 subcomplex as more of a negative modulator of transcription and not an absolute repressor of transcription. We have updated several areas of the text to try and clarify our position on this point.

4. *There is genome wide binding data for CDK8 PMID: 16630888, it is may be useful to look at the data to propose some broader functional context for MedPIWI.*

Author’s response: We have examined the ChIP-chip data from *Saccharomyces cerevisiae* and compared it with promoter-mapping publicly-available small RNA data set in yeast. While we observe some interesting differences in the small RNA content which maps to promoter regions occupied by different components of the Mediator complex, at this point we are unable to conclusively identify any trends that might inform the relationship between the CDK8 subcomplex and small RNA derived from promoter regions. Several issues constrain the efficacy of this analysis, chief among these is 1) the multiple levels of regulation which appear to contribute to the decision of the loaded Mediator complex to move between RNA polII active and inactive states, many of which could influence small RNA content at the promoter (see Figure 3B) and 2) the medPIWI “switch” between activity and inactivity is likely to be subtle: instead of the binary presence/absence of small RNA at a promoter it is likely to be the presence of “enough” small RNA which triggers the switch. Additionally, the required concentration of small RNA could depend on several promoter-specific contextual factors including genome sequence, local DNA structure, or presence/absence of ancillary protein domains. Some of the following additional issues could bring clarity to such an analysis: 1) ChIP-chip does not identify the precise location of the binding of Mediator components on the genome sequence, to gauge the location of the Mediator complex (and thus the sites from which potential small RNA are generated) ChIP-seq experiments would be of considerable value. 2) Existing data extracts RNA for ChIP and small RNA-seq under different growth conditions and different time points; uniformity in such conditions would remove considerable noise. 3) Recent advances in sequencing technology would yield a deeper small RNA data set that what is currently available. This is particularly important given that the absolute number of small RNAs derived from any single promoter region tends to be quite low, particularly in relation to other classes of small RNA.

5. *Potential molecular mechanism models of pPIWI in the section “Functional implications of pPIWI-RE coding systems: A novel RNA-dependent restriction system” and the information in the last two paragraphs of “Partners and physical interactions of Med13: functional implications for the MedPIWI module in eukaryotic transcription regulation” could be synthesized in to schematic figures and this I believe would help the reading very much.*

Authors’ response: We have added a figure (Figure 3) summarizing the implications of these findings.

6. *In the “introduction” section there seems to be abrupt transition from last paragraph in the first page to first paragraph in the next page just above “results and discussion” i.e. from background information on pPIWI to reporting novel family of PIWI modules.*

Authors’ response: We have added a few additional lines to the introduction in an attempt to smoothen the transition.

## Competing interests

The authors declare that they have no competing interests.

## Authors’ contributions

AMB collected data; AMB, LMI and LA analyzed the data; AMB and LA wrote the manuscript that was read and approved by all authors.

## Supplementary Material

Additional file 1 **Provides access to: 1) comprehensive list of Genbank identifiers, architectures and operons of modules uncovered in this study.** 2) A comprehensive set of alignments of domains reported here in text format. Click here for file
