# Supplementary material for: Two novel PIWI families: roles in inter-genomic conflicts in bacteria and Mediator-dependent modulation of transcription in eukaryotes
Source: Biol Direct. 2013 Jun 8;8:13. doi: 10.1186/1745-6150-8-13 (PMC3702460; doi:10.1186/1745-6150-8-13)
Supplement: Additional file 1 — Provides access to: 1) comprehensive list of Genbank identifiers, architectures and operons of modules uncovered in this study. 2) A comprehensive set of alignments of domains reported here in text format. [file 1745-6150-8-13-S1.html]

Additional file 1 for the manuscript "Two novel PIWI families: roles in inter-genomic conflicts in bacteria and Mediator-dependent modulation of transcription in eukaryotes"


  
**Additional File 1  
Two novel PIWI families: roles in inter-genomic conflicts in bacteria and Mediator-dependent modulation of transcription in eukaryotes**  
  
A Maxwell Burroughs, Lakshminarayan M Iyer, and L. Aravind\*
  
\* *Address for correspondence: L. Aravind (aravind@mail.nih.gov)*  
  
 *National Center for Biotechnology Information, National Library of Medicine, National Institutes of Health, Bethesda, MD 20894, USA*
  

---

|  |
| --- |
|  |
| **Abstract****Background:** The PIWI module, found in the PIWI/AGO superfamily of proteins, is a critical component of several cellular pathways including germline maintenance, chromatin organization, regulation of splicing, RNA interference, and virus suppression. It binds a guide strand which helps it target complementary nucleic strands.  **Results**: Here we report the discovery of two divergent, novel families of PIWI modules, the first such to be described since the initial discovery of the PIWI/AGO superfamily over a decade ago. Both families display conservation patterns consistent with the binding of oligonucleotide guide strands. The first family is bacterial in its phyletic pattern and is typically encoded by a distinctive three-gene operon with alongside genes for a restriction endonuclease fold enzyme and a helicase of the DinG family. The second family is found only in eukaryotes. It is the core conserved module of the Med13 protein, a subunit of the CDK8 subcomplex of the transcription regulatory Mediator complex.  **Conclusions:** Based on the presence of the DinG family helicase, which specifically acts on R-loops, we infer that the first family of PIWI modules is part of a novel RNA-dependent restriction system likely to target invasive DNA from phages and/or plasmids. It is predicted to facilitate restriction of actively transcribed invading DNA by utilizing RNA guides. The PIWI family found in the eukaryotic Med13 proteins throws new light on the regulatory switch through which the CDK8 subcomplex modulates transcription at the Mediator-bound promoters of highly transcribed genes. We propose that this involves recognition of small RNAs by the PIWI module in Med13 resulting in a conformational switch that propagates through the mediator complex. |

|  |  |  |  |
| --- | --- | --- | --- |
|  | ---  **Contents**- **Findings relating to the pPIWI-RE family**   **- A. Gene names, gi numbers, phyletic distributions, and domain architectures**   **- B. Conserved gene neighborhoods**   - 1) standalone 'neigborhood' - 2) three-gene neighborhood**- C. Alignments**   - 1) X domain - 2) Y+zinc\_ribbon+REase - 3) Z+DinG-type\_helicase- **Findings relating to the MedPIWI family**     **- A. Gene names, gi numbers, phyletic distributions, and domain architectures**     **- B. Alignments**     - 1) Med13\_N - 2) linker region - 3) animal-specific CxC domain - 4) fungi-specific domain - 5) plant-specific domain1. **Organism abbreviations**      ---      **- A. Gene names, gi numbers, phyletic distributions, and domain architectures of the pPIWI-RE family**       ```     Proteins lists are grouped into shared domain architectures, which are provided at the top of each group following the '#;'.     GI              Gene name               Length  Class                                           Species                                                         Genbank defline     #;X+pPIWI_RE     219666713       Dhaf_0647               854     bacteria>firmicutes                             Desulfitobacterium hafniense DCB-2                              hypothetical protein Dhaf_0647 [Desulfitobacterium hafniense DCB-2].     49185448        BAS2440                 803     bacteria>firmicutes                             Bacillus anthracis str. Sterne                                  hypothetical protein BAS2440 [Bacillus anthracis str. Sterne].     49479681        BT9727_2402             803     bacteria>firmicutes                             Bacillus thuringiensis serovar konkukian str. 97-27             hypothetical protein BT9727_2402 [Bacillus thuringiensis serovar konkukian str. 97-27].     118478006       BALH_2354               803     bacteria>firmicutes                             Bacillus thuringiensis str. Al Hakam                            hypothetical protein BALH_2354 [Bacillus thuringiensis str. Al Hakam].     228927677       bthur0010_23850         803     bacteria>firmicutes                             Bacillus thuringiensis serovar pondicheriensis BGSC 4BA1        hypothetical protein bthur0010_23850 [Bacillus thuringiensis serovar pondicheriensis BGSC 4BA1].     228933907       bthur0009_23670         803     bacteria>firmicutes                             Bacillus thuringiensis serovar andalousiensis BGSC 4AW1         hypothetical protein bthur0009_23670 [Bacillus thuringiensis serovar andalousiensis BGSC 4AW1].     229030311       bcere0028_23860         803     bacteria>firmicutes                             Bacillus cereus AH1271                                          hypothetical protein bcere0028_23860 [Bacillus cereus AH1271].     229139270       bcere0013_23810         803     bacteria>firmicutes                             Bacillus cereus BDRD-ST26                                       hypothetical protein bcere0013_23810 [Bacillus cereus BDRD-ST26].     229196797       bcere0001_23530         803     bacteria>firmicutes                             Bacillus cereus m1293                                           hypothetical protein bcere0001_23530 [Bacillus cereus m1293].     118476564       BALH_0836               796     bacteria>firmicutes                             Bacillus thuringiensis str. Al Hakam                            hypothetical protein BALH_0836 [Bacillus thuringiensis str. Al Hakam].     229103229       bcere0019_23790         796     bacteria>firmicutes                             Bacillus cereus Rock3-28                                        hypothetical protein bcere0019_23790 [Bacillus cereus Rock3-28].     229116140       bcere0017_24290         796     bacteria>firmicutes                             Bacillus cereus Rock1-3                                         hypothetical protein bcere0017_24290 [Bacillus cereus Rock1-3].     42781704        BCE_2645                795     bacteria>firmicutes                             Bacillus cereus ATCC 10987                                      hypothetical protein BCE_2645 [Bacillus cereus ATCC 10987].     47568744        BCE_G9241_2572          795     bacteria>firmicutes                             Bacillus cereus G9241                                           conserved hypothetical protein protein [Bacillus cereus G9241].     52142875        BCZK2365                795     bacteria>firmicutes                             Bacillus cereus E33L                                            hypothetical protein BCZK2365 [Bacillus cereus E33L].     196040276       BC059799_2582           795     bacteria>firmicutes                             Bacillus cereus NVH0597-99                                      hypothetical protein BC059799_2582 [Bacillus cereus NVH0597-99].     206972467       BCAH1134_2627           795     bacteria>firmicutes                             Bacillus cereus AH1134                                          hypothetical protein BCAH1134_2627 [Bacillus cereus AH1134].     222096147       BCQ_2487                795     bacteria>firmicutes                             Bacillus cereus Q1                                              hypothetical protein BCQ_2487 [Bacillus cereus Q1].     228901173       bthur0014_23700         795     bacteria>firmicutes                             Bacillus thuringiensis IBL 4222                                 hypothetical protein bthur0014_23700 [Bacillus thuringiensis IBL 4222].     228952942       bthur0006_23330         795     bacteria>firmicutes                             Bacillus thuringiensis serovar kurstaki str. T03a001            hypothetical protein bthur0006_23330 [Bacillus thuringiensis serovar kurstaki str. T03a001].     228965554       bthur0004_23810         795     bacteria>firmicutes                             Bacillus thuringiensis serovar sotto str. T04001                hypothetical protein bthur0004_23810 [Bacillus thuringiensis serovar sotto str. T04001].     229044315       bcere0027_23450         795     bacteria>firmicutes                             Bacillus cereus AH676                                           hypothetical protein bcere0027_23450 [Bacillus cereus AH676].     229145200       bcere0012_23570         795     bacteria>firmicutes                             Bacillus cereus BDRD-ST24                                       hypothetical protein bcere0012_23570 [Bacillus cereus BDRD-ST24].     229161512       bcere0009_22980         795     bacteria>firmicutes                             Bacillus cereus R309803                                         hypothetical protein bcere0009_22980 [Bacillus cereus R309803].     229173281       bcere0006_23840         795     bacteria>firmicutes                             Bacillus cereus MM3                                             hypothetical protein bcere0006_23840 [Bacillus cereus MM3].     254743179       BantKB_010100019582     795     bacteria>firmicutes                             Bacillus anthracis str. Kruger B                                hypothetical protein BantKB_19582 [Bacillus anthracis str. Kruger B].     301054158       BACI_c25950             795     bacteria>firmicutes                             Bacillus cereus biovar anthracis str. CI                        hypothetical protein BACI_c25950 [Bacillus cereus biovar anthracis str. CI].     384180517       YBT020_13145            795     bacteria>firmicutes                             Bacillus thuringiensis serovar finitimus YBT-020                hypothetical protein YBT020_13145 [Bacillus thuringiensis serovar finitimus YBT-020].     401082495       IC1_02385               795     bacteria>firmicutes                             Bacillus cereus VD022                                           hypothetical protein IC1_02385 [Bacillus cereus VD022].     401098960       IC5_01579               795     bacteria>firmicutes                             Bacillus cereus AND1407                                         hypothetical protein IC5_01579 [Bacillus cereus AND1407].     401144408       IEI_02470               795     bacteria>firmicutes                             Bacillus cereus BAG5X2-1                                        hypothetical protein IEI_02470 [Bacillus cereus BAG5X2-1].     401187460       IGW_02253               795     bacteria>firmicutes                             Bacillus cereus ISP3191                                         hypothetical protein IGW_02253 [Bacillus cereus ISP3191].     401209057       II9_02953               795     bacteria>firmicutes                             Bacillus cereus MSX-D12                                         hypothetical protein II9_02953 [Bacillus cereus MSX-D12].     401229117       IIE_02377               795     bacteria>firmicutes                             Bacillus cereus VD045                                           hypothetical protein IIE_02377 [Bacillus cereus VD045].     401243031       IIK_02257               795     bacteria>firmicutes                             Bacillus cereus VD102                                           hypothetical protein IIK_02257 [Bacillus cereus VD102].     401248563       IIM_01825               795     bacteria>firmicutes                             Bacillus cereus VD107                                           hypothetical protein IIM_01825 [Bacillus cereus VD107].     401277315       IK9_02317               795     bacteria>firmicutes                             Bacillus cereus VD166                                           hypothetical protein IK9_02317 [Bacillus cereus VD166].     401284002       IKA_02291               795     bacteria>firmicutes                             Bacillus cereus VD169                                           hypothetical protein IKA_02291 [Bacillus cereus VD169].     401292968       IKG_02413               795     bacteria>firmicutes                             Bacillus cereus VD200                                           hypothetical protein IKG_02413 [Bacillus cereus VD200].     401650340       ICW_03146               795     bacteria>firmicutes                             Bacillus cereus BAG2X1-2                                        hypothetical protein ICW_03146 [Bacillus cereus BAG2X1-2].     402557164       BCK_21745               795     bacteria>firmicutes                             Bacillus cereus FRI-35                                          hypothetical protein BCK_21745 [Bacillus cereus FRI-35].     401256284       IIO_02425               794     bacteria>firmicutes                             Bacillus cereus VD115                                           hypothetical protein IIO_02425 [Bacillus cereus VD115].     401651349       ICU_02467               794     bacteria>firmicutes                             Bacillus cereus BAG2X1-1                                        hypothetical protein ICU_02467 [Bacillus cereus BAG2X1-1].     401658950       ICY_02309               794     bacteria>firmicutes                             Bacillus cereus BAG2X1-3                                        hypothetical protein ICY_02309 [Bacillus cereus BAG2X1-3].     407705060       MC28_1824               794     bacteria>firmicutes                             Bacillus thuringiensis MC28                                     Translation initiation inhibitor [Bacillus thuringiensis MC28].     163940471       BcerKBAB4_2522          793     bacteria>firmicutes                             Bacillus weihenstephanensis KBAB4                               hypothetical protein BcerKBAB4_2522 [Bacillus weihenstephanensis KBAB4].     229011883       bmyco0001_23290         793     bacteria>firmicutes                             Bacillus mycoides DSM 2048                                      hypothetical protein bmyco0001_23290 [Bacillus mycoides DSM 2048].     229017911       bcere0030_24490         793     bacteria>firmicutes                             Bacillus cereus AH1273                                          hypothetical protein bcere0030_24490 [Bacillus cereus AH1273].     229133448       bcere0014_23670         793     bacteria>firmicutes                             Bacillus cereus BDRD-ST196                                      hypothetical protein bcere0014_23670 [Bacillus cereus BDRD-ST196].     229167458       bcere0007_24210         793     bacteria>firmicutes                             Bacillus cereus AH621                                           hypothetical protein bcere0007_24210 [Bacillus cereus AH621].     401090269       IC3_00638               793     bacteria>firmicutes                             Bacillus cereus VD142                                           hypothetical protein IC3_00638 [Bacillus cereus VD142].     401105882       IE3_02748               793     bacteria>firmicutes                             Bacillus cereus BAG3X2-1                                        hypothetical protein IE3_02748 [Bacillus cereus BAG3X2-1].     401136851       IEE_02625               793     bacteria>firmicutes                             Bacillus cereus BAG5X1-1                                        hypothetical protein IEE_02625 [Bacillus cereus BAG5X1-1].     401143784       IEQ_02258               793     bacteria>firmicutes                             Bacillus cereus BAG6X1-2                                        hypothetical protein IEQ_02258 [Bacillus cereus BAG6X1-2].     401153241       IEW_02468               793     bacteria>firmicutes                             Bacillus cereus CER057                                          hypothetical protein IEW_02468 [Bacillus cereus CER057].     401163724       IG7_02522               793     bacteria>firmicutes                             Bacillus cereus HuA2-4                                          hypothetical protein IG7_02522 [Bacillus cereus HuA2-4].     401171654       IGC_02795               793     bacteria>firmicutes                             Bacillus cereus HuA4-10                                         hypothetical protein IGC_02795 [Bacillus cereus HuA4-10].     401188135       II3_04835               793     bacteria>firmicutes                             Bacillus cereus MC67                                            hypothetical protein II3_04835 [Bacillus cereus MC67].     401234658       III_02773               793     bacteria>firmicutes                             Bacillus cereus VD078                                           hypothetical protein III_02773 [Bacillus cereus VD078].     401298050       IKM_02828               793     bacteria>firmicutes                             Bacillus cereus VDM022                                          hypothetical protein IKM_02828 [Bacillus cereus VDM022].     401302369       IKO_02075               793     bacteria>firmicutes                             Bacillus cereus VDM034                                          hypothetical protein IKO_02075 [Bacillus cereus VDM034].     401636867       ICG_02882               793     bacteria>firmicutes                             Bacillus cereus BAG1X1-3                                        hypothetical protein ICG_02882 [Bacillus cereus BAG1X1-3].     402440352       IEM_00307               793     bacteria>firmicutes                             Bacillus cereus BAG6O-2                                         hypothetical protein IEM_00307 [Bacillus cereus BAG6O-2].     402453655       IG3_02199               793     bacteria>firmicutes                             Bacillus cereus HuA2-1                                          hypothetical protein IG3_02199 [Bacillus cereus HuA2-1].     229097149       bcere0020_23930         790     bacteria>firmicutes                             Bacillus cereus Rock3-29                                        hypothetical protein bcere0020_23930 [Bacillus cereus Rock3-29].     401257576       IK3_02862               790     bacteria>firmicutes                             Bacillus cereus VD148                                           hypothetical protein IK3_02862 [Bacillus cereus VD148].     402414458       IEA_02936               790     bacteria>firmicutes                             Bacillus cereus BAG4X2-1                                        hypothetical protein IEA_02936 [Bacillus cereus BAG4X2-1].     228921300       bthur0011_23050         788     bacteria>firmicutes                             Bacillus thuringiensis serovar huazhongensis BGSC 4BD1          hypothetical protein bthur0011_23050 [Bacillus thuringiensis serovar huazhongensis BGSC 4BD1].     401274498       IK7_03079               788     bacteria>firmicutes                             Bacillus cereus VD156                                           hypothetical protein IK7_03079 [Bacillus cereus VD156].     152975657       Bcer98_1891             781     bacteria>firmicutes                             Bacillus cytotoxicus NVH 391-98                                 hypothetical protein Bcer98_1891 [Bacillus cytotoxicus NVH 391-98].     254391263       SSCG_03447              1024    bacteria>actinobacteria                         Streptomyces clavuligerus ATCC 27064                            hypothetical protein SSCG_03447 [Streptomyces clavuligerus ATCC 27064].     406692971       SM8_00528               1015    bacteria>actinobacteria                         Streptomyces sp. SM8                                            hypothetical protein SM8_00528 [Streptomyces sp. SM8].     386383244       STSU_10299              1014    bacteria>actinobacteria                         Streptomyces tsukubaensis NRRL18488                             hypothetical protein STSU_10299 [Streptomyces tsukubaensis NRRL18488].     345850902       SZN_14191               1013    bacteria>actinobacteria                         Streptomyces zinciresistens K42                                 hypothetical protein SZN_14191 [Streptomyces zinciresistens K42].     269125748       Tcur_1501               965     bacteria>actinobacteria                         Thermomonospora curvata DSM 43183                               hypothetical protein Tcur_1501 [Thermomonospora curvata DSM 43183].     291439986       SSFG_05080              961     bacteria>actinobacteria                         Streptomyces ghanaensis ATCC 14672                              predicted protein [Streptomyces ghanaensis ATCC 14672].     392942503       FraQA3DRAFT_1301        960     bacteria>actinobacteria                         Frankia sp. QA3                                                 hypothetical protein FraQA3DRAFT_1301 [Frankia sp. QA3].     302867037       Micau_2559              959     bacteria>actinobacteria                         Micromonospora aurantiaca ATCC 27029                            hypothetical protein Micau_2559 [Micromonospora aurantiaca ATCC 27029].     357392957       KSE_60760               957     bacteria>actinobacteria                         Kitasatospora setae KM-6054                                     hypothetical protein KSE_60760 [Kitasatospora setae KM-6054].     375092908       SacmaDRAFT_0160         909     bacteria>actinobacteria                         Saccharomonospora marina XMU15                                  hypothetical protein SacmaDRAFT_0160 [Saccharomonospora marina XMU15].     302870454       Micau_6013              901     bacteria>actinobacteria                         Micromonospora aurantiaca ATCC 27029                            hypothetical protein Micau_6013 [Micromonospora aurantiaca ATCC 27029].     357393639       KSE_67660               901     bacteria>actinobacteria                         Kitasatospora setae KM-6054                                     hypothetical protein KSE_67660 [Kitasatospora setae KM-6054].     386357487       SCATT_38400             994     bacteria>actinobacteria                         Streptomyces cattleya NRRL 8057 = DSM 46488                     hypothetical protein SCATT_38400 [Streptomyces cattleya NRRL 8057 = DSM 46488].     297204964       SSEG_07535              934     bacteria>actinobacteria                         Streptomyces sviceus ATCC 29083                                 conserved hypothetical protein [Streptomyces sviceus ATCC 29083].     329936082       SGM_1373                934     bacteria>actinobacteria                         Streptomyces griseoaurantiacus M045                             hypothetical protein SGM_1373 [Streptomyces griseoaurantiacus M045].     302559389       SSRG_02904              876     bacteria>actinobacteria                         Streptomyces griseoflavus Tu4000                                conserved hypothetical protein [Streptomyces griseoflavus Tu4000].     295837642       SSBG_00887              866     bacteria>actinobacteria                         Streptomyces sp. SPB74                                          conserved hypothetical protein [Streptomyces sp. SPB74].     254390288       SSCG_02833              836     bacteria>actinobacteria                         Streptomyces clavuligerus ATCC 27064                            hypothetical protein SSCG_02833 [Streptomyces clavuligerus ATCC 27064].     407881735       BN6_20570               830     bacteria>actinobacteria                         Saccharothrix espanaensis DSM 44229                             hypothetical protein BN6_20570 [Saccharothrix espanaensis DSM 44229].     346056320       NCGM1179_1019           783     bacteria>proteobacteria>gammaproteobacteria     Pseudomonas aeruginosa NCMG1179                                 hypothetical protein NCGM1179_1019 [Pseudomonas aeruginosa NCMG1179].     386022140       PSTAA_3563              783     bacteria>proteobacteria>gammaproteobacteria     Pseudomonas stutzeri DSM 4166                                   hypothetical protein PSTAA_3563 [Pseudomonas stutzeri DSM 4166].     399519610       BN5_00852               783     bacteria>proteobacteria>gammaproteobacteria     Pseudomonas pseudoalcaligenes CECT 5344                         hypothetical protein BN5_00852 [Pseudomonas pseudoalcaligenes CECT 5344].     403248864       PACIG1_1802             783     bacteria>proteobacteria>gammaproteobacteria     Pseudomonas aeruginosa CIG1                                     hypothetical protein PACIG1_1802 [Pseudomonas aeruginosa CIG1].     404549289       PABE173_1560            783     bacteria>proteobacteria>gammaproteobacteria     Pseudomonas aeruginosa ATCC 25324                               hypothetical protein PABE173_1560 [Pseudomonas aeruginosa ATCC 25324].     398928563       PMI28_03164             777     bacteria>proteobacteria>gammaproteobacteria     Pseudomonas sp. GM48                                            hypothetical protein PMI28_03164 [Pseudomonas sp. GM48].     126655716       CY0110_18932            1074    bacteria>cyanobacteria                          Cyanothece sp. CCY0110                                          hypothetical protein CY0110_18932 [Cyanothece sp. CCY0110].     158336199       AM1_3061                1031    bacteria>cyanobacteria                          Acaryochloris marina MBIC11017                                  hypothetical protein AM1_3061 [Acaryochloris marina MBIC11017].     117920626       Shewana3_2182           923     bacteria>proteobacteria>gammaproteobacteria     Shewanella sp. ANA-3                                            hypothetical protein Shewana3_2182 [Shewanella sp. ANA-3].     210630742       COLSTE_00451            880     bacteria>actinobacteria                         Collinsella stercoris DSM 13279                                 hypothetical protein COLSTE_00451 [Collinsella stercoris DSM 13279].     257790573       Elen_0816               841     bacteria>actinobacteria                         Eggerthella lenta DSM 2243                                      hypothetical protein Elen_0816 [Eggerthella lenta DSM 2243].     295106506       GPA_14540               841     bacteria>actinobacteria                         Gordonibacter pamelaeae 7-10-1-b                                hypothetical protein [Gordonibacter pamelaeae 7-10-1-b].     126656944       CY0110_02159            882     bacteria>cyanobacteria                          Cyanothece sp. CCY0110                                          hypothetical protein CY0110_02159 [Cyanothece sp. CCY0110].     359458560       ACCM5_010100007527      608     bacteria>cyanobacteria                          Acaryochloris sp. CCMEE 5410                                    hypothetical protein ACCM5_07527 [Acaryochloris sp. CCMEE 5410].     260588610       BLAHAN_05684            875     bacteria>firmicutes                             Blautia hansenii DSM 20583                                      hypothetical protein BLAHAN_05684 [Blautia hansenii DSM 20583].     331082037       HMPREF0992_00089        875     bacteria>firmicutes                             Lachnospiraceae bacterium 6_1_63FAA                             hypothetical protein HMPREF0992_00089 [Lachnospiraceae bacterium 6_1_63FAA].     381168803       PHAMO_470060            873     bacteria>proteobacteria>alphaproteobacteria     Phaeospirillum molischianum DSM 120                             hypothetical protein PHAMO_470060 [Phaeospirillum molischianum DSM 120].     372487594       Dsui_0912               834     bacteria>proteobacteria>betaproteobacteria      Dechlorosoma suillum PS                                         hypothetical protein Dsui_0912 [Dechlorosoma suillum PS].     153817620       A5C_0038                1117    bacteria>proteobacteria>gammaproteobacteria     Vibrio cholerae NCTC 8457                                       hypothetical protein A5C_0038 [Vibrio cholerae NCTC 8457].     309791273       OSCT_1755               973     bacteria>chloroflexi                            Oscillochloris trichoides DG-6                                  hypothetical protein OSCT_1755 [Oscillochloris trichoides DG-6].     172039225       cce_4312                964     bacteria>cyanobacteria                          Cyanothece sp. ATCC 51142                                       hypothetical protein cce_4312 [Cyanothece sp. ATCC 51142].     186681799       Npun_F1340              919     bacteria>cyanobacteria                          Nostoc punctiforme PCC 73102                                    hypothetical protein Npun_F1340 [Nostoc punctiforme PCC 73102].     290956757       SCAB_22631              917     bacteria>actinobacteria                         Streptomyces scabiei 87.22                                      hypothetical protein SCAB_22631 [Streptomyces scabiei 87.22].     220930098       Ccel_2716               907     bacteria>firmicutes                             Clostridium cellulolyticum H10                                  hypothetical protein Ccel_2716 [Clostridium cellulolyticum H10].     392549087       PrubA2_010100022162     810     bacteria>proteobacteria>gammaproteobacteria     Pseudoalteromonas rubra ATCC 29570                              hypothetical protein PrubA2_22162 [Pseudoalteromonas rubra ATCC 29570].     403070934       ONdio_010100015231      784     bacteria>firmicutes                             Oceanobacillus sp. Ndiop                                        hypothetical protein ONdio_15231 [Oceanobacillus sp. Ndiop].     #;peptidase+HTH+X+PIWI_like     168216165       AC7_0986                1045    bacteria>firmicutes                             Clostridium perfringens NCTC 8239                               conserved hypothetical protein [Clostridium perfringens NCTC 8239].     #;pPIWI_RE(fragment)     228918309       bthur0012_54800         562     bacteria>firmicutes                             Bacillus thuringiensis serovar pulsiensis BGSC 4CC1             hypothetical protein bthur0012_54800 [Bacillus thuringiensis serovar pulsiensis BGSC 4CC1].     228946236       bthur0007_23840         562     bacteria>firmicutes                             Bacillus thuringiensis serovar monterrey BGSC 4AJ1              hypothetical protein bthur0007_23840 [Bacillus thuringiensis serovar monterrey BGSC 4AJ1].     228985726       bthur0001_24170         562     bacteria>firmicutes                             Bacillus thuringiensis serovar tochigiensis BGSC 4Y1            hypothetical protein bthur0001_24170 [Bacillus thuringiensis serovar tochigiensis BGSC 4Y1].     229076658       bcere0024_53290         562     bacteria>firmicutes                             Bacillus cereus Rock4-18                                        hypothetical protein bcere0024_53290 [Bacillus cereus Rock4-18].     229091630       bcere0021_24360         562     bacteria>firmicutes                             Bacillus cereus Rock3-42                                        hypothetical protein bcere0021_24360 [Bacillus cereus Rock3-42].     229122201       bcere0016_24970         562     bacteria>firmicutes                             Bacillus cereus 95/8201                                         hypothetical protein bcere0016_24970 [Bacillus cereus 95/8201].     229156215       bcere0010_24040         562     bacteria>firmicutes                             Bacillus cereus ATCC 4342                                       hypothetical protein bcere0010_24040 [Bacillus cereus ATCC 4342].     296503191       BMB171_C2359            560     bacteria>firmicutes                             Bacillus thuringiensis BMB171                                   hypothetical protein BMB171_C2359 [Bacillus thuringiensis BMB171].     365161000       HMPREF1014_02618        560     bacteria>firmicutes                             Bacillus sp. 7_6_55CFAA_CT2                                     hypothetical protein HMPREF1014_02618 [Bacillus sp. 7_6_55CFAA_CT2].     401100341       IE1_02918               560     bacteria>firmicutes                             Bacillus cereus BAG3O-2                                         hypothetical protein IE1_02918 [Bacillus cereus BAG3O-2].     401122728       IE9_02295               560     bacteria>firmicutes                             Bacillus cereus BAG4X12-1                                       hypothetical protein IE9_02295 [Bacillus cereus BAG4X12-1].     401199662       II5_02383               560     bacteria>firmicutes                             Bacillus cereus MSX-A1                                          hypothetical protein II5_02383 [Bacillus cereus MSX-A1].     401270990       IK5_00545               560     bacteria>firmicutes                             Bacillus cereus VD154                                           hypothetical protein IK5_00545 [Bacillus cereus VD154].     401640757       ICE_01894               560     bacteria>firmicutes                             Bacillus cereus BAG1X1-2                                        hypothetical protein ICE_01894 [Bacillus cereus BAG1X1-2].     402448903       IGE_03026               560     bacteria>firmicutes                             Bacillus cereus HuB1-1                                          hypothetical protein IGE_03026 [Bacillus cereus HuB1-1].     402417097       IEK_02860               556     bacteria>firmicutes                             Bacillus cereus BAG6O-1                                         hypothetical protein IEK_02860 [Bacillus cereus BAG6O-1].     401215665       IIA_02415               553     bacteria>firmicutes                             Bacillus cereus VD014                                           hypothetical protein IIA_02415 [Bacillus cereus VD014].     228908351       bthur0013_25120         533     bacteria>firmicutes                             Bacillus thuringiensis IBL 200                                  hypothetical protein bthur0013_25120 [Bacillus thuringiensis IBL 200].     228943767       bthur0008_63080         533     bacteria>firmicutes                             Bacillus thuringiensis serovar berliner ATCC 10792              hypothetical protein bthur0008_63080 [Bacillus thuringiensis serovar berliner ATCC 10792].     229110075       bcere0018_23310         533     bacteria>firmicutes                             Bacillus cereus Rock1-15                                        hypothetical protein bcere0018_23310 [Bacillus cereus Rock1-15].     229130921       bcere0015_53380         533     bacteria>firmicutes                             Bacillus cereus BDRD-Cer4                                       hypothetical protein bcere0015_53380 [Bacillus cereus BDRD-Cer4].     229150863       bcere0011_24130         533     bacteria>firmicutes                             Bacillus cereus m1550                                           hypothetical protein bcere0011_24130 [Bacillus cereus m1550].     229178898       bcere0005_22530         533     bacteria>firmicutes                             Bacillus cereus 172560W                                         hypothetical protein bcere0005_22530 [Bacillus cereus 172560W].     229190713       bcere0002_23800         533     bacteria>firmicutes                             Bacillus cereus ATCC 10876                                      hypothetical protein bcere0002_23800 [Bacillus cereus ATCC 10876].     30020729        BC2602                  514     bacteria>firmicutes                             Bacillus cereus ATCC 14579                                      hypothetical protein BC2602 [Bacillus cereus ATCC 14579].     75762950        RBTH_01086              514     bacteria>firmicutes                             Bacillus thuringiensis serovar israelensis ATCC 35646           hypothetical protein RBTH_01086 [Bacillus thuringiensis serovar israelensis ATCC 35646].     218231490       BCB4264_A2643           514     bacteria>firmicutes                             Bacillus cereus B4264                                           hypothetical protein BCB4264_A2643 [Bacillus cereus B4264].     218897627       BCG9842_B2680           514     bacteria>firmicutes                             Bacillus cereus G9842                                           hypothetical protein BCG9842_B2680 [Bacillus cereus G9842].     384186677       CT43_CH2604             514     bacteria>firmicutes                             Bacillus thuringiensis serovar chinensis CT-43                  hypothetical protein CT43_CH2604 [Bacillus thuringiensis serovar chinensis CT-43].     229024132       bcere0029_24560         511     bacteria>firmicutes                             Bacillus cereus AH1272                                          hypothetical protein bcere0029_24560 [Bacillus cereus AH1272].     229066888       bcere0026_58690         492     bacteria>firmicutes                             Bacillus cereus AH603                                           hypothetical protein bcere0026_58690 [Bacillus cereus AH603].     401131174       IEC_01786               417     bacteria>firmicutes                             Bacillus cereus BAG5O-1                                         hypothetical protein IEC_01786 [Bacillus cereus BAG5O-1].     401173249       IGK_01806               411     bacteria>firmicutes                             Bacillus cereus HuB4-10                                         hypothetical protein IGK_01806 [Bacillus cereus HuB4-10].     402462750       IGI_02851               411     bacteria>firmicutes                             Bacillus cereus HuB2-9                                          hypothetical protein IGI_02851 [Bacillus cereus HuB2-9].     65319924        Bant_01003271           351     bacteria>firmicutes                             Bacillus anthracis str. A2012                                   hypothetical protein Bant_01003271 [Bacillus anthracis str. A2012].     402462751       IGI_02852               345     bacteria>firmicutes                             Bacillus cereus HuB2-9                                          hypothetical protein IGI_02852 [Bacillus cereus HuB2-9].     229024133       bcere0029_24570         281     bacteria>firmicutes                             Bacillus cereus AH1272                                          hypothetical protein bcere0029_24570 [Bacillus cereus AH1272].     228915206       bthur0012_24260         241     bacteria>firmicutes                             Bacillus thuringiensis serovar pulsiensis BGSC 4CC1             hypothetical protein bthur0012_24260 [Bacillus thuringiensis serovar pulsiensis BGSC 4CC1].     229181835       bcere0005_51640         241     bacteria>firmicutes                             Bacillus cereus 172560W                                         hypothetical protein bcere0005_51640 [Bacillus cereus 172560W].     31415820        BC2555                  233                                                     Bacillus phage phBC6A52                                         hypothetical protein BC2555 [Bacillus phage phBC6A52].     218235423       BCB4264_A2588           233     bacteria>firmicutes                             Bacillus cereus B4264                                           hypothetical protein BCB4264_A2588 [Bacillus cereus B4264].     218897573       BCG9842_B2735           233     bacteria>firmicutes                             Bacillus cereus G9842                                           hypothetical protein BCG9842_B2735 [Bacillus cereus G9842].     228908343       bthur0013_25040         233     bacteria>firmicutes                             Bacillus thuringiensis IBL 200                                  hypothetical protein bthur0013_25040 [Bacillus thuringiensis IBL 200].     228939735       bthur0008_23890         233     bacteria>firmicutes                             Bacillus thuringiensis serovar berliner ATCC 10792              hypothetical protein bthur0008_23890 [Bacillus thuringiensis serovar berliner ATCC 10792].     228961936       bthur0005_53380         233     bacteria>firmicutes                             Bacillus thuringiensis serovar pakistani str. T13001            hypothetical protein bthur0005_53380 [Bacillus thuringiensis serovar pakistani str. T13001].     229060295       bcere0026_23960         233     bacteria>firmicutes                             Bacillus cereus AH603                                           hypothetical protein bcere0026_23960 [Bacillus cereus AH603].     229073695       bcere0025_58010         233     bacteria>firmicutes                             Bacillus cereus F65185                                          hypothetical protein bcere0025_58010 [Bacillus cereus F65185].     229079786       bcere0023_24390         233     bacteria>firmicutes                             Bacillus cereus Rock4-2                                         hypothetical protein bcere0023_24390 [Bacillus cereus Rock4-2].     229150813       bcere0011_23630         233     bacteria>firmicutes                             Bacillus cereus m1550                                           hypothetical protein bcere0011_23630 [Bacillus cereus m1550].     229190700       bcere0002_23670         233     bacteria>firmicutes                             Bacillus cereus ATCC 10876                                      hypothetical protein bcere0002_23670 [Bacillus cereus ATCC 10876].     296503147       BMB171_C2315            233     bacteria>firmicutes                             Bacillus thuringiensis BMB171                                   hypothetical protein BMB171_C2315 [Bacillus thuringiensis BMB171].     365160942       HMPREF1014_02561        233     bacteria>firmicutes                             Bacillus sp. 7_6_55CFAA_CT2                                     hypothetical protein HMPREF1014_02561 [Bacillus sp. 7_6_55CFAA_CT2].     401099996       IE1_02968               233     bacteria>firmicutes                             Bacillus cereus BAG3O-2                                         hypothetical protein IE1_02968 [Bacillus cereus BAG3O-2].     401123263       IE9_02242               233     bacteria>firmicutes                             Bacillus cereus BAG4X12-1                                       hypothetical protein IE9_02242 [Bacillus cereus BAG4X12-1].     401215612       IIA_02362               233     bacteria>firmicutes                             Bacillus cereus VD014                                           hypothetical protein IIA_02362 [Bacillus cereus VD014].     229075068       bcere0024_23820         232     bacteria>firmicutes                             Bacillus cereus Rock4-18                                        hypothetical protein bcere0024_23820 [Bacillus cereus Rock4-18].     228950417       bthur0007_64730         231     bacteria>firmicutes                             Bacillus thuringiensis serovar monterrey BGSC 4AJ1              hypothetical protein bthur0007_64730 [Bacillus thuringiensis serovar monterrey BGSC 4AJ1].     228988956       bthur0001_55850         223     bacteria>firmicutes                             Bacillus thuringiensis serovar tochigiensis BGSC 4Y1            hypothetical protein bthur0001_55850 [Bacillus thuringiensis serovar tochigiensis BGSC 4Y1].     229110028       bcere0018_22840         223     bacteria>firmicutes                             Bacillus cereus Rock1-15                                        hypothetical protein bcere0018_22840 [Bacillus cereus Rock1-15].     229159076       bcere0010_52450         223     bacteria>firmicutes                             Bacillus cereus ATCC 4342                                       hypothetical protein bcere0010_52450 [Bacillus cereus ATCC 4342].     65319922        Bant_01003269           218     bacteria>firmicutes                             Bacillus anthracis str. A2012                                   hypothetical protein Bant_01003269 [Bacillus anthracis str. A2012].     78033437        mgI394                  496     bacteria>proteobacteria>alphaproteobacteria     Magnetospirillum gryphiswaldense MSR-1                          hypothetical protein mgI394 [Magnetospirillum gryphiswaldense MSR-1].     17229461        all1969                 404     bacteria>cyanobacteria                          Nostoc sp. PCC 7120                                             hypothetical protein all1969 [Nostoc sp. PCC 7120].     126660228       CY0110_06704            370     bacteria>cyanobacteria                          Cyanothece sp. CCY0110                                          hypothetical protein CY0110_06704 [Cyanothece sp. CCY0110].     307153445       Cyan7822_3614           313     bacteria>cyanobacteria                          Cyanothece sp. PCC 7822                                         hypothetical protein Cyan7822_3614 [Cyanothece sp. PCC 7822].     17229462        all1970                 281     bacteria>cyanobacteria                          Nostoc sp. PCC 7120                                             hypothetical protein all1970 [Nostoc sp. PCC 7120].     17229465        all1973                 279     bacteria>cyanobacteria                          Nostoc sp. PCC 7120                                             hypothetical protein all1973 [Nostoc sp. PCC 7120].     254390295       SSCG_02840              176     bacteria>actinobacteria                         Streptomyces clavuligerus ATCC 27064                            hypothetical protein SSCG_02840 [Streptomyces clavuligerus ATCC 27064].     339488247       PPS_3348                171     bacteria>proteobacteria>gammaproteobacteria     Pseudomonas putida S16                                          hypothetical protein PPS_3348 [Pseudomonas putida S16].     359458561       ACCM5_010100007532      163     bacteria>cyanobacteria                          Acaryochloris sp. CCMEE 5410                                    hypothetical protein ACCM5_07532 [Acaryochloris sp. CCMEE 5410].     85813600        -                       147     bacteria>actinobacteria                         Streptomyces ribosidificus                                      hypothetical protein [Streptomyces ribosidificus].     307153444       Cyan7822_3613           132     bacteria>cyanobacteria                          Cyanothece sp. PCC 7822                                         hypothetical protein Cyan7822_3613 [Cyanothece sp. PCC 7822].     65319925        Bant_01003272           125     bacteria>firmicutes                             Bacillus anthracis str. A2012                                   hypothetical protein Bant_01003272 [Bacillus anthracis str. A2012].     401173248       IGK_01805               111     bacteria>firmicutes                             Bacillus cereus HuB4-10                                         hypothetical protein IGK_01805 [Bacillus cereus HuB4-10].     65319923        Bant_01003270           109     bacteria>firmicutes                             Bacillus anthracis str. A2012                                   hypothetical protein Bant_01003270 [Bacillus anthracis str. A2012].     226946971       Avin_49780              107     bacteria>proteobacteria>gammaproteobacteria     Azotobacter vinelandii DJ                                       hypothetical protein Avin_49780 [Azotobacter vinelandii DJ].     254384452       SSAG_04095              97      bacteria>actinobacteria                         Streptomyces sp. Mg1                                            hypothetical protein SSAG_04095 [Streptomyces sp. Mg1].     ```      Back to Contents       ---      **- B. Conserved gene neighborhoods for pPIWI-RE family**      **- 1) standalone 'neigborhood'**       ```     GI              Gene neigborhood                                                                        Class                                           Species     49185448        X+pPIWI_RE*->                                                                           bacteria>firmicutes                             Bacillus anthracis str. Sterne     49479681        X+pPIWI_RE*->                                                                           bacteria>firmicutes                             Bacillus thuringiensis serovar konkukian str. 97-27     118478006       X+pPIWI_RE*->                                                                           bacteria>firmicutes                             Bacillus thuringiensis str. Al Hakam     228927677       X+pPIWI_RE*->                                                                           bacteria>firmicutes                             Bacillus thuringiensis serovar pondicheriensis BGSC 4BA1     228933907       X+pPIWI_RE*->                                                                           bacteria>firmicutes                             Bacillus thuringiensis serovar andalousiensis BGSC 4AW1     229030311       X+pPIWI_RE*->                                                                           bacteria>firmicutes                             Bacillus cereus AH1271     229139270       X+pPIWI_RE*->                                                                           bacteria>firmicutes                             Bacillus cereus BDRD-ST26     229196797       X+pPIWI_RE*->                                                                           bacteria>firmicutes                             Bacillus cereus m1293     229103229       X+pPIWI_RE*->                                                                           bacteria>firmicutes                             Bacillus cereus Rock3-28     229116140       X+pPIWI_RE*->                                                                           bacteria>firmicutes                             Bacillus cereus Rock1-3     42781704        X+pPIWI_RE*->                                                                           bacteria>firmicutes                             Bacillus cereus ATCC 10987     47568744        X+pPIWI_RE*->                                                                           bacteria>firmicutes                             Bacillus cereus G9241     52142875        X+pPIWI_RE*->                                                                           bacteria>firmicutes                             Bacillus cereus E33L     196040276       <-X+pPIWI_RE*                                                                           bacteria>firmicutes                             Bacillus cereus NVH0597-99     206972467       <-X+pPIWI_RE*                                                                           bacteria>firmicutes                             Bacillus cereus AH1134     222096147       X+pPIWI_RE*->                                                                           bacteria>firmicutes                             Bacillus cereus Q1     228901173       X+pPIWI_RE*->                                                                           bacteria>firmicutes                             Bacillus thuringiensis IBL 4222     228952942       X+pPIWI_RE*->                                                                           bacteria>firmicutes                             Bacillus thuringiensis serovar kurstaki str. T03a001     228965554       X+pPIWI_RE*->                                                                           bacteria>firmicutes                             Bacillus thuringiensis serovar sotto str. T04001     229044315       X+pPIWI_RE*->                                                                           bacteria>firmicutes                             Bacillus cereus AH676     229145200       X+pPIWI_RE*->                                                                           bacteria>firmicutes                             Bacillus cereus BDRD-ST24     229161512       X+pPIWI_RE*->                                                                           bacteria>firmicutes                             Bacillus cereus R309803     229173281       X+pPIWI_RE*->                                                                           bacteria>firmicutes                             Bacillus cereus MM3     254743179       <-X+pPIWI_RE*                                                                           bacteria>firmicutes                             Bacillus anthracis str. Kruger B     301054158       X+pPIWI_RE*->                                                                           bacteria>firmicutes                             Bacillus cereus biovar anthracis str. CI     384180517       X+pPIWI_RE*->                                                                           bacteria>firmicutes                             Bacillus thuringiensis serovar finitimus YBT-020     401082495       <-X+pPIWI_RE*                                                                           bacteria>firmicutes                             Bacillus cereus VD022     401098960       X+pPIWI_RE*->                                                                           bacteria>firmicutes                             Bacillus cereus AND1407     401144408       <-X+pPIWI_RE*                                                                           bacteria>firmicutes                             Bacillus cereus BAG5X2-1     401187460       <-X+pPIWI_RE*                                                                           bacteria>firmicutes                             Bacillus cereus ISP3191     401209057       <-X+pPIWI_RE*                                                                           bacteria>firmicutes                             Bacillus cereus MSX-D12     401229117       <-X+pPIWI_RE*                                                                           bacteria>firmicutes                             Bacillus cereus VD045     401243031       <-X+pPIWI_RE*                                                                           bacteria>firmicutes                             Bacillus cereus VD102     401248563       <-X+pPIWI_RE*                                                                           bacteria>firmicutes                             Bacillus cereus VD107     401277315       <-X+pPIWI_RE*                                                                           bacteria>firmicutes                             Bacillus cereus VD166     401284002       X+pPIWI_RE*->                                                                           bacteria>firmicutes                             Bacillus cereus VD169     401292968       X+pPIWI_RE*->                                                                           bacteria>firmicutes                             Bacillus cereus VD200     401650340       <-X+pPIWI_RE*                                                                           bacteria>firmicutes                             Bacillus cereus BAG2X1-2     402557164       <-X+pPIWI_RE*                                                                           bacteria>firmicutes                             Bacillus cereus FRI-35     401256284       <-X+pPIWI_RE*                                                                           bacteria>firmicutes                             Bacillus cereus VD115     401651349       X+pPIWI_RE*->                                                                           bacteria>firmicutes                             Bacillus cereus BAG2X1-1     401658950       X+pPIWI_RE*->                                                                           bacteria>firmicutes                             Bacillus cereus BAG2X1-3     407705060       X+pPIWI_RE*->                                                                           bacteria>firmicutes                             Bacillus thuringiensis MC28     163940471       <-X+pPIWI_RE*                                                                           bacteria>firmicutes                             Bacillus weihenstephanensis KBAB4     229011883       X+pPIWI_RE*->                                                                           bacteria>firmicutes                             Bacillus mycoides DSM 2048     229017911       X+pPIWI_RE*->                                                                           bacteria>firmicutes                             Bacillus cereus AH1273     229133448       X+pPIWI_RE*->                                                                           bacteria>firmicutes                             Bacillus cereus BDRD-ST196     229167458       X+pPIWI_RE*->                                                                           bacteria>firmicutes                             Bacillus cereus AH621     401090269       X+pPIWI_RE*->                                                                           bacteria>firmicutes                             Bacillus cereus VD142     401105882       X+pPIWI_RE*->                                                                           bacteria>firmicutes                             Bacillus cereus BAG3X2-1     401136851       X+pPIWI_RE*->                                                                           bacteria>firmicutes                             Bacillus cereus BAG5X1-1     401143784       <-X+pPIWI_RE*                                                                           bacteria>firmicutes                             Bacillus cereus BAG6X1-2     401153241       <-X+pPIWI_RE*                                                                           bacteria>firmicutes                             Bacillus cereus CER057     401163724       <-X+pPIWI_RE*                                                                           bacteria>firmicutes                             Bacillus cereus HuA2-4     401171654       X+pPIWI_RE*->                                                                           bacteria>firmicutes                             Bacillus cereus HuA4-10     401188135       X+pPIWI_RE*->                                                                           bacteria>firmicutes                             Bacillus cereus MC67     401234658       X+pPIWI_RE*->                                                                           bacteria>firmicutes                             Bacillus cereus VD078     401298050       X+pPIWI_RE*->                                                                           bacteria>firmicutes                             Bacillus cereus VDM022     401302369       <-X+pPIWI_RE*                                                                           bacteria>firmicutes                             Bacillus cereus VDM034     401636867       X+pPIWI_RE*->                                                                           bacteria>firmicutes                             Bacillus cereus BAG1X1-3     402440352       <-X+pPIWI_RE*                                                                           bacteria>firmicutes                             Bacillus cereus BAG6O-2     402453655       <-X+pPIWI_RE*                                                                           bacteria>firmicutes                             Bacillus cereus HuA2-1     229097149       X+pPIWI_RE*->                                                                           bacteria>firmicutes                             Bacillus cereus Rock3-29     401257576       <-X+pPIWI_RE*                                                                           bacteria>firmicutes                             Bacillus cereus VD148     402414458       <-X+pPIWI_RE*                                                                           bacteria>firmicutes                             Bacillus cereus BAG4X2-1     228921300       X+pPIWI_RE*->                                                                           bacteria>firmicutes                             Bacillus thuringiensis serovar huazhongensis BGSC 4BD1     401274498       <-X+pPIWI_RE*                                                                           bacteria>firmicutes                             Bacillus cereus VD156     152975657       <-X+pPIWI_RE*                                                                           bacteria>firmicutes                             Bacillus cytotoxicus NVH 391-98     228918309       pPIWI_RE(fragment)*->                                                                   bacteria>firmicutes                             Bacillus thuringiensis serovar pulsiensis BGSC 4CC1     228946236       pPIWI_RE(fragment)*->                                                                   bacteria>firmicutes                             Bacillus thuringiensis serovar monterrey BGSC 4AJ1     228985726       pPIWI_RE(fragment)*->                                                                   bacteria>firmicutes                             Bacillus thuringiensis serovar tochigiensis BGSC 4Y1     229076658       pPIWI_RE(fragment)*->                                                                   bacteria>firmicutes                             Bacillus cereus Rock4-18     229091630       pPIWI_RE(fragment)*->                                                                   bacteria>firmicutes                             Bacillus cereus Rock3-42     229122201       pPIWI_RE(fragment)*->                                                                   bacteria>firmicutes                             Bacillus cereus 95/8201     229156215       pPIWI_RE(fragment)*->                                                                   bacteria>firmicutes                             Bacillus cereus ATCC 4342     296503191       pPIWI_RE(fragment)*->                                                                   bacteria>firmicutes                             Bacillus thuringiensis BMB171     365161000       pPIWI_RE(fragment)*->                                                                   bacteria>firmicutes                             Bacillus sp. 7_6_55CFAA_CT2     401100341       <-pPIWI_RE(fragment)*                                                                   bacteria>firmicutes                             Bacillus cereus BAG3O-2     401122728       pPIWI_RE(fragment)*->                                                                   bacteria>firmicutes                             Bacillus cereus BAG4X12-1     401199662       <-pPIWI_RE(fragment)*                                                                   bacteria>firmicutes                             Bacillus cereus MSX-A1     401270990       pPIWI_RE(fragment)*->                                                                   bacteria>firmicutes                             Bacillus cereus VD154     401640757       pPIWI_RE(fragment)*->                                                                   bacteria>firmicutes                             Bacillus cereus BAG1X1-2     402448903       <-pPIWI_RE(fragment)*                                                                   bacteria>firmicutes                             Bacillus cereus HuB1-1     402417097       <-pPIWI_RE(fragment)*                                                                   bacteria>firmicutes                             Bacillus cereus BAG6O-1     401215665       pPIWI_RE(fragment)*->                                                                   bacteria>firmicutes                             Bacillus cereus VD014     228908351       pPIWI_RE(fragment)*->                                                                   bacteria>firmicutes                             Bacillus thuringiensis IBL 200     228943767       pPIWI_RE(fragment)*->                                                                   bacteria>firmicutes                             Bacillus thuringiensis serovar berliner ATCC 10792     229110075       pPIWI_RE(fragment)*->                                                                   bacteria>firmicutes                             Bacillus cereus Rock1-15     229130921       <-pPIWI_RE(fragment)*                                                                   bacteria>firmicutes                             Bacillus cereus BDRD-Cer4     229150863       pPIWI_RE(fragment)*->                                                                   bacteria>firmicutes                             Bacillus cereus m1550     229178898       pPIWI_RE(fragment)*->                                                                   bacteria>firmicutes                             Bacillus cereus 172560W     229190713       pPIWI_RE(fragment)*->                                                                   bacteria>firmicutes                             Bacillus cereus ATCC 10876     30020729        pPIWI_RE(fragment)*->                                                                   bacteria>firmicutes                             Bacillus cereus ATCC 14579     75762950        <-pPIWI_RE(fragment)*                                                                   bacteria>firmicutes                             Bacillus thuringiensis serovar israelensis ATCC 35646     218231490       pPIWI_RE(fragment)*->                                                                   bacteria>firmicutes                             Bacillus cereus B4264     218897627       pPIWI_RE(fragment)*->                                                                   bacteria>firmicutes                             Bacillus cereus G9842     384186677       pPIWI_RE(fragment)*->                                                                   bacteria>firmicutes                             Bacillus thuringiensis serovar chinensis CT-43     229024132       pPIWI_RE(fragment)*->pPIWI_RE(fragment)->                                               bacteria>firmicutes                             Bacillus cereus AH1272     229066888       pPIWI_RE(fragment)*->                                                                   bacteria>firmicutes                             Bacillus cereus AH603     401131174       pPIWI_RE(fragment)*->                                                                   bacteria>firmicutes                             Bacillus cereus BAG5O-1     401173249       pPIWI_RE(fragment)->pPIWI_RE(fragment)*->                                               bacteria>firmicutes                             Bacillus cereus HuB4-10     402462750       <-pPIWI_RE(fragment)*<-pPIWI_RE(fragment)                                               bacteria>firmicutes                             Bacillus cereus HuB2-9     65319924        pPIWI_RE(fragment)->pPIWI_RE(fragment)->pPIWI_RE(fragment)*->pPIWI_RE(fragment)->       bacteria>firmicutes                             Bacillus anthracis str. A2012     228915206       pPIWI_RE(fragment)*->                                                                   bacteria>firmicutes                             Bacillus thuringiensis serovar pulsiensis BGSC 4CC1     229181835       pPIWI_RE(fragment)*->                                                                   bacteria>firmicutes                             Bacillus cereus 172560W     31415820        pPIWI_RE(fragment)*->                                                                   Bacillus phage phBC6A52                         hypothetical protein BC2555 [Bacillus phage phBC6A52].     218235423       pPIWI_RE(fragment)*->                                                                   bacteria>firmicutes                             Bacillus cereus B4264     218897573       pPIWI_RE(fragment)*->                                                                   bacteria>firmicutes                             Bacillus cereus G9842     228908343       pPIWI_RE(fragment)*->                                                                   bacteria>firmicutes                             Bacillus thuringiensis IBL 200     228939735       pPIWI_RE(fragment)*->                                                                   bacteria>firmicutes                             Bacillus thuringiensis serovar berliner ATCC 10792     228961936       <-pPIWI_RE(fragment)*                                                                   bacteria>firmicutes                             Bacillus thuringiensis serovar pakistani str. T13001     229060295       pPIWI_RE(fragment)*->                                                                   bacteria>firmicutes                             Bacillus cereus AH603     229073695       pPIWI_RE(fragment)*->                                                                   bacteria>firmicutes                             Bacillus cereus F65185     229079786       pPIWI_RE(fragment)*->                                                                   bacteria>firmicutes                             Bacillus cereus Rock4-2     229150813       pPIWI_RE(fragment)*->                                                                   bacteria>firmicutes                             Bacillus cereus m1550     229190700       pPIWI_RE(fragment)*->                                                                   bacteria>firmicutes                             Bacillus cereus ATCC 10876     296503147       pPIWI_RE(fragment)*->                                                                   bacteria>firmicutes                             Bacillus thuringiensis BMB171     365160942       pPIWI_RE(fragment)*->                                                                   bacteria>firmicutes                             Bacillus sp. 7_6_55CFAA_CT2     401099996       <-pPIWI_RE(fragment)*                                                                   bacteria>firmicutes                             Bacillus cereus BAG3O-2     401123263       pPIWI_RE(fragment)*->                                                                   bacteria>firmicutes                             Bacillus cereus BAG4X12-1     401215612       pPIWI_RE(fragment)*->                                                                   bacteria>firmicutes                             Bacillus cereus VD014     229075068       pPIWI_RE(fragment)*->                                                                   bacteria>firmicutes                             Bacillus cereus Rock4-18     228950417       pPIWI_RE(fragment)*->                                                                   bacteria>firmicutes                             Bacillus thuringiensis serovar monterrey BGSC 4AJ1     228988956       <-pPIWI_RE(fragment)*                                                                   bacteria>firmicutes                             Bacillus thuringiensis serovar tochigiensis BGSC 4Y1     229110028       pPIWI_RE(fragment)*->                                                                   bacteria>firmicutes                             Bacillus cereus Rock1-15     229159076       pPIWI_RE(fragment)*->                                                                   bacteria>firmicutes                             Bacillus cereus ATCC 4342     78033437        pPIWI_RE(fragment)*->                                                                   bacteria>proteobacteria>alphaproteobacteria     Magnetospirillum gryphiswaldense MSR-1     339488247       pPIWI_RE(fragment)*->                                                                   bacteria>proteobacteria>gammaproteobacteria     Pseudomonas putida S16     85813600        <-pPIWI_RE(fragment)*                                                                   bacteria>actinobacteria                         Streptomyces ribosidificus     226946971       pPIWI_RE(fragment)*->                                                                   bacteria>proteobacteria>gammaproteobacteria     Azotobacter vinelandii DJ     254384452       <-pPIWI_RE(fragment)*                                                                   bacteria>actinobacteria                         Streptomyces sp. Mg1     269125748       <-X+pPIWI_RE*                                                                           bacteria>actinobacteria                         Thermomonospora curvata DSM 43183     168216165       <-peptidase+HTH+X+PIWI_like*                                                            bacteria>firmicutes                             Clostridium perfringens NCTC 8239     ```      **- 2) three-gene neighborhood**       ```     Note the strict gene order in this neighborhood     GI              Gene neighborhood                                                                                               class                                        species     17229461        <-pPIWI_RE(fragment)*<-pPIWI_RE(fragment)<-?<-?<-pPIWI_RE(fragment)<-Z+DinG-type_helicase<-Y+zn_ribbon+RE-like  bacteria>cyanobacteria                       Nostoc sp. PCC 7120     307153445       <-pPIWI_RE(fragment)<-pPIWI_RE(fragment)*<-Z+DinG-type_helicase<-Y+zn_ribbon+RE-like                            bacteria>cyanobacteria                       Cyanothece sp. PCC 7822     126660228       <-pPIWI_RE(fragment)*<-?<-?<-?<-Y+zn_ribbon+RE-like                                                             bacteria>cyanobacteria                       Cyanothece sp. CCY0110     219666713       Y+zn_ribbon+RE-like->Z+DinG-type_helicase->X+pPIWI_RE*->                                                        bacteria>firmicutes                          Desulfitobacterium hafniense DCB-2     118476564       Y+zn_ribbon+RE-like->Z+DinG-type_helicase->X+pPIWI_RE*->                                                        bacteria>firmicutes                          Bacillus thuringiensis str. Al Hakam     254391263       <-X+pPIWI_RE*<-Z+DinG-type_helicase<-Y+zn_ribbon+RE-like                                                        bacteria>actinobacteria                      Streptomyces clavuligerus ATCC 27064     406692971       <-X+pPIWI_RE*<-Z+DinG-type_helicase<-Y+zn_ribbon+RE-like                                                        bacteria>actinobacteria                      Streptomyces sp. SM8     386383244       Y+zn_ribbon+RE-like->Z+DinG-type_helicase->X+pPIWI_RE*->                                                        bacteria>actinobacteria                      Streptomyces tsukubaensis NRRL18488     345850902       <-X+pPIWI_RE*<-Z+DinG-type_helicase<-Y+zn_ribbon+RE-like                                                        bacteria>actinobacteria                      Streptomyces zinciresistens K42     291439986       Y+zn_ribbon+RE-like->Z+DinG-type_helicase->X+pPIWI_RE*->                                                        bacteria>actinobacteria                      Streptomyces ghanaensis ATCC 14672     392942503       <-X+pPIWI_RE*<-Z+DinG-type_helicase<-Y+zn_ribbon+RE-like                                                        bacteria>actinobacteria                      Frankia sp. QA3     302867037       <-X+pPIWI_RE*<-Z+DinG-type_helicase<-Y+zn_ribbon+RE-like                                                        bacteria>actinobacteria                      Micromonospora aurantiaca ATCC 27029     357392957       Y+zn_ribbon+RE-like->Z+DinG-type_helicase->X+pPIWI_RE*->                                                        bacteria>actinobacteria                      Kitasatospora setae KM-6054     375092908       Y+zn_ribbon+RE-like->Z+DinG-type_helicase->X+pPIWI_RE*->                                                        bacteria>actinobacteria                      Saccharomonospora marina XMU15     302870454       Y+zn_ribbon+RE-like->Z+DinG-type_helicase->X+pPIWI_RE*->                                                        bacteria>actinobacteria                      Micromonospora aurantiaca ATCC 27029     357393639       Y+zn_ribbon+RE-like->Z+DinG-type_helicase->X+pPIWI_RE*->                                                        bacteria>actinobacteria                      Kitasatospora setae KM-6054     386357487       <-X+pPIWI_RE*<-Z+DinG-type_helicase<-Y+zn_ribbon+RE-like                                                        bacteria>actinobacteria                      Streptomyces cattleya NRRL 8057 = DSM 46488     297204964       <-X+pPIWI_RE*<-Z+DinG-type_helicase<-Y+zn_ribbon+RE-like                                                        bacteria>actinobacteria                      Streptomyces sviceus ATCC 29083     329936082       Y+zn_ribbon+RE-like->Z+DinG-type_helicase->X+pPIWI_RE*->                                                        bacteria>actinobacteria                      Streptomyces griseoaurantiacus M045     302559389       Y+zn_ribbon+RE-like->Z+DinG-type_helicase->X+pPIWI_RE*->                                                        bacteria>actinobacteria                      Streptomyces griseoflavus Tu4000     295837642       Y+zn_ribbon+RE-like->Z+DinG-type_helicase->X+pPIWI_RE*->                                                        bacteria>actinobacteria                      Streptomyces sp. SPB74     254390288       <-X+pPIWI_RE*<-Z+DinG-type_helicase<-Y+zn_ribbon+RE-like<-?||?-><-?<-?<-pPIWI_RE(fragment)                      bacteria>actinobacteria                      Streptomyces clavuligerus ATCC 27064     407881735       Y+zn_ribbon+RE-like->Z+DinG-type_helicase->X+pPIWI_RE*->                                                        bacteria>actinobacteria                      Saccharothrix espanaensis DSM 44229     346056320       <-X+pPIWI_RE*<-Z+DinG-type_helicase                                                                             bacteria>proteobacteria>gammaproteobacteria  Pseudomonas aeruginosa NCMG1179     386022140       Y+zn_ribbon+RE-like->Z+DinG-type_helicase->X+pPIWI_RE*->                                                        bacteria>proteobacteria>gammaproteobacteria  Pseudomonas stutzeri DSM 4166     399519610       <-X+pPIWI_RE*<-Z+DinG-type_helicase<-Y+zn_ribbon+RE-like                                                        bacteria>proteobacteria>gammaproteobacteria  Pseudomonas pseudoalcaligenes CECT 5344     403248864       <-X+pPIWI_RE*<-Z+DinG-type_helicase                                                                             bacteria>proteobacteria>gammaproteobacteria  Pseudomonas aeruginosa CIG1     404549289       Z+DinG-type_helicase->X+pPIWI_RE*->                                                                             bacteria>proteobacteria>gammaproteobacteria  Pseudomonas aeruginosa ATCC 25324     398928563       Y+zn_ribbon+RE-like->Z+DinG-type_helicase->X+pPIWI_RE*->                                                        bacteria>proteobacteria>gammaproteobacteria  Pseudomonas sp. GM48     126655716       <-X+pPIWI_RE*<-Z+DinG-type_helicase                                                                             bacteria>cyanobacteria                       Cyanothece sp. CCY0110     158336199       <-X+pPIWI_RE*<-Z+DinG-type_helicase<-MerR_HTH+Y+zinc_ribbon+RE-like                                             bacteria>cyanobacteria                       Acaryochloris marina MBIC11017     117920626       <-X+pPIWI_RE*<-Z+DinG-type_helicase<-Y+zn_ribbon+RE-like                                                        bacteria>proteobacteria>gammaproteobacteria  Shewanella sp. ANA-3     210630742       <-X+pPIWI_RE*<-Z+DinG-type_helicase<-Y+zn_ribbon+RE-like                                                        bacteria>actinobacteria                      Collinsella stercoris DSM 13279     257790573       <-X+pPIWI_RE*<-Z+DinG-type_helicase<-Y+zn_ribbon+RE-like                                                        bacteria>actinobacteria                      Eggerthella lenta DSM 2243     295106506       <-X+pPIWI_RE*||?-><-Y+zn_ribbon+RE-like                                                                         bacteria>actinobacteria                      Gordonibacter pamelaeae 7-10-1-b     126656944       Y+zn_ribbon+RE-like->Z+DinG-type_helicase->?->X+pPIWI_RE*->                                                     bacteria>cyanobacteria                       Cyanothece sp. CCY0110     359458560       <-X+pPIWI_RE*<-pPIWI_RE(fragment)<-Z+DinG-type_helicase<-Y+zn_ribbon+RE-like                                    bacteria>cyanobacteria                       Acaryochloris sp. CCMEE 5410     260588610       Y+zn_ribbon+RE-like->Z+DinG-type_helicase->X+pPIWI_RE*->                                                        bacteria>firmicutes                          Blautia hansenii DSM 20583     331082037       <-X+pPIWI_RE*<-Z+DinG-type_helicase<-Y+zn_ribbon+RE-like                                                        bacteria>firmicutes                          Lachnospiraceae bacterium 6_1_63FAA     381168803       <-X+pPIWI_RE*<-Z+DinG-type_helicase<-?<-LexA_HTH+Y+zinc_ribbon+RE-like                                          bacteria>proteobacteria>alphaproteobacteria  Phaeospirillum molischianum DSM 120     372487594       ?_HTH+Y+zinc_ribbon+RE-like->Z+DinG-type_helicase->X+pPIWI_RE*->                                                bacteria>proteobacteria>betaproteobacteria   Dechlorosoma suillum PS     153817620       <-X+pPIWI_RE*<-Z+DinG-type_helicase<-Y+zinc_ribbon+RE-like                                                      bacteria>proteobacteria>gammaproteobacteria  Vibrio cholerae NCTC 8457     309791273       LexA_HTH+Y+zinc_ribbon+RE-like->Z+DinG-type_helicase->X+pPIWI_RE*->                                             bacteria>chloroflexi                         Oscillochloris trichoides DG-6     172039225       Y+zn_ribbon+RE-like->Z+DinG-type_helicase->X+pPIWI_RE*->                                                        bacteria>cyanobacteria                       Cyanothece sp. ATCC 51142     186681799       TetR_HTH+Y+zinc_ribbon+RE-like->Z+DinG-type_helicase->X+pPIWI_RE*->                                             bacteria>cyanobacteria                       Nostoc punctiforme PCC 73102     290956757       Y+zn_ribbon+RE-like->Z+DinG-type_helicase->X+pPIWI_RE*->                                                        bacteria>actinobacteria                      Streptomyces scabiei 87.22     220930098       <-X+pPIWI_RE*<-Z+DinG-type_helicase<-?<-Y+zn_ribbon+RE-like                                                     bacteria>firmicutes                          Clostridium cellulolyticum H10     392549087       Y+zn_ribbon+RE-like->Z+DinG-type_helicase->X+pPIWI_RE*->                                                        bacteria>proteobacteria>gammaproteobacteria  Pseudoalteromonas rubra ATCC 29570     403070934       <-X+pPIWI_RE*<-Z+DinG-type_helicase<-Y+zn_ribbon+RE-like                                                        bacteria>firmicutes                          Oceanobacillus sp. Ndiop     ```      Back to Contents       ---      **- C. Alignments for the pPIWI-RE family**      Alignments are preceded by secondary structure predictions and are followed by amino acid consensus at various thresholds.     **- 1) X domain**       ```     N-terminally fused to MID, pPIWI_RE domains (see Fig. 2)     FINAL           -HHHHHHHHHHH--------------EEEEEE--HHHHHHHHHHHH---HHHHHHHHHH-------HHHHHHHHHH-----EEE---------------------EEEEE----------HHHHHHHHHHHHHH-----------E----HHHHHHH---HH--------------------------------HHHHHHHHHHHHHHH---------------------------EEEEE----------EEEEE----------------------------------EEEEEEEEEEEEE-------EEEEEEEEEEEEE---------------------EEEE----E------------------EEEEEEE------------------HHHHHHH-----HHHHHHHH-----HHHHHHHH--------------------EEEEE--------------------------------HHHHHHHHHHHHH--------------------------------------------------------------------HHHHHHHHHHHHHHHH--------------------HHHHHHHHH--HHHHHHHHHHHHHHHH     ALIGN           ----HHHHHHH---------------EEEEE----HHHHHHHHHH--------------------HHHHHHHHHHHH---------------------------EEEEE-----------HHHHHHHHHHHHHH-----------------HHHHHH---HH---------------------------------HHHHHHHHHHHHHH---------------------------EEEEE----------EEEE-----------------------------------HHHHHEEEEEEEE--------EEEEE---EEH----------------------EEE------------------------HHHHHHH----------------------HHH-----HHHHHHHHH-------HHHH---------------------EEEEEE-------------------------------HHHHHHHHHHHHH--------------------------------------------------------------------HHHHHHHHHHHH-HHHH-------------------HHHHHHHH------HHHHHHHHHHHHH     HMM             --HHHHHH------------------EEEEE--HHHHHHHHHHHHH---H-----------EEEEEEHHHHHHHHHHH---EEEE-------------------EEEEEE----------HHHHHHHHHHHHHHH--------------HHHHHHHH---HH----------EEEEE---------------HHHHHHHHHHHHHHHH----------------------------EEEEEEEEE------EEEEE-E--------------------------------EEEEEEEEEEEEEE------EEEEEEEEEEEEE--------------------EEEEE----EE-----------------EEEEEEE-----------------------HH-----HHHHHHHHHHH--HHHHHHHH---------------------EEEEE-------------------------------HHHHHHHHHHHHHHHH-----------------------------------------------------------HHH-----HHHHHHHHHHHHHH----------------------EEEEE----HHHHHHHHHHHHHHH-     FREQ            -HHHHHHHHHHH--E------------EEEEE--HHHHHHHHHHHH---HHHHHHHHHH-------HHHHHHHHHH----HHHHH--E-----EE----------EEEE-----------HHHHHHHHHHHHHH---------EEE------HHHHH---HH--------------------------EE-------HHHHHHHHHHHH--------------------------EEEE------------HEH-------------------------------------EEEEEEEEEEEE--------EEEEEEEEHHHHHH---------------H----EEEE---H------------------HHHHHHHHHHH--H-----------HHHHHHH-----H------------HHHHHHH--------------------EEEEE---------------------------------HHHHHHHHHHHH------------------H----------E-----------------------------------H---H-------HH-H--HH--------------------HHHHHHHHH-----HHHHHHHHHHHHH     PSSM            ---HHHH--------------------EEEEE--HHHHHHHHHHH---------------------HHHHHHHHHH----------------------------EEEEE-----------HHHHHHHHHHHHHH---------------HHHHHHHH---HHH-------------------------------HHHHHHHHHHHHHHH---------------------------EEEEEEE--------EEEEE-E--------------------------------EEEEEEEEEEEEE------EEEEEEEE-EEEE---------------------EEEEE-----------------------EEEEEEE-----------------------HH-----HHHHHHHHH-----HHHHHHH---------------------E-E---------------------------------HHHHHHHHHHHHH--------------------------------------------------------------------HHHHHHHHHH---------------------------HHHHHHHH--HHHHHHHHHHHHHHHH     386022140       MKALELRTSLFKFDATQLGQA---YRVVIGPQYLDAWQA-----LQ---GLVKKPHPG-----LPTT-GLEEMLAVLSRGPV----KVDLFPQKKG----GVSAILML-------YPLSV-DTINEVLHLWSMD----------VLRIWNEQLVGIE---GKLIVTDVV--PLDTSRLVTPG--------DISSLAYTVIPWLVGQALIQ------------------TPMQAARPIKLYQAAD-------SSLLAWD------------DPIVSENDV---------RYASALHAIEPTLVLLHGR-PQPYIQLRVKLTQVM-------------------PNLVGKKKHAWVKTGDL-----------IVKAKLKTKKTDEG------------WETTYEH-------------------PVEKLLTFMGVQSFPPMVDG---DIPVDSDVRPIYA--------------IPPSNPMIASGPGPLFLDQAGFHLLASLPG---------------------------------------------------------------TAPLLVKKAVA--------------------------SLREEKVVNTGEAANLNAMVLAAHADVM     399519610       MKALELRTSLFKFDATQLGQA---YRVVIGPQYLDAWQA-----LQ---GLVKKPHPG-----LPTT-GLEEMLAVLSRGPV----KVDLFPQK------AGGVSAILML-----YPLSV-DTINEVLHLWSMD----------VLRIWNEQLVGIE---GKLIVTDVV--PLDTSRLVTPG--------DISSLAYTVIPWLVGQALVQ------------------TPMQAARPIKLYQAAD-------SSLLAWD------------DPIVSENDV---------RYASALHAIEPSLVLLRGR-LQPYIQLHVKLTQVM-------------------PNLVGKKKHAWVKTGDL-----------IVKAKLKTKKTDEG------------WETTYEH-------------------PVEKLLTFMGVQSFPPMVDG---DIPVDSDVRPIYA--------------IPPSNPMIASGPGPLFLDQAGFHLLTSLPG---------------------------------------------------------------TAPLLVKKAVA--------------------------SLREEKVVTTGEVANLNVMVLAAHADVM     346056320       MKTLELRTSLFRFDPTQLGRA---YRVVIGEHYLDAWQA-----LK---GLAKKPHPG-----LPII-ALEEMLTVLSGGPV----KVNLSPQKDG----GVSAILLL-------KPLPI-DTINETLRLWSMD----------VLRIWNERLEGFE---GKLAVTDLV--PLETSLLVTPG--------DISSLAYTVIPWLVGQAMIR------------------NPMQASKPITLYQASD-------ATLLAWD------------DPVVSENDI---------RYASAVHAIEPKLVLLRGR-AEPYLQLRVKLSQVM-------------------PSLVGTKKHAWVKVGDQ-----------IVKAKLKTQPHEGG------------WKTTYEH-------------------PIEDLLGFMGVSSFPSIQEG---DIPIDSDFRPIYA--------------IPPSNPLIASGPGPLFLDQAGFHLLDNLPG---------------------------------------------------------------TSPLLARKAVG--------------------------SLREEKAQATGEVVTLPVMVLAAHSEVM     167631983       --MEKLKLLTFENIVEPLLNE-----SVSFIYFPIEWLDIVEIHYK---TFLLTSKLK----------RLNERLYDMFSDIL----FIQHNPYVL-----NENTPWIVSK-----EPIRK-EQLDYIFQSWYEI-----------IHDWKPN--------KLIESPKYEWHYDLISNL---------TVLHDKEVYSKWVPALISHIFCE---RPVQL-----------ENINEEDIYFSPLRT----QNICEAMSEP---------------IKDEK----------TQDYFAYVFRFEYITRGGE-NIPLLNVSIGIRRFYQEY-----NHPDISLLLRRKRGMILISTPEFASNNN----------KLRFVKLKVQQATNGI----------KWIKIF-------RNLKDDFHIGGE--VELEHILQYPKDYMLG--------TNLRVLLPYNER-------------IYKFQGTKIKPGIKVKEREYLFKAFQQKFAY---------------FTLIP-ECKKLETNNENE------------------------------IFPLIAPKGLE--------------------------TITLEIW--------SEKISLEVEQALF     398928563       -MNTVLRTNLFRFDPAQLGHA---YQAVLDRSFYDAWYA-----LK---AILPNADKN-----LPTR-GLEELLAALSHGPV----KVQARPSA------EGRVPAILLL-----NRIST-ERLNEAFEVWASE----------VLKEHQVRLPDLA---KRLVVQDV-------IDLDAAALFTEDTTLPSA---YIVVPWLAGQLLAA-----KPL-----------QAQADVPVKLTPAADYRHDRGGMTLLTWE------------NPIVAPN-----------QSAIAHHVLDLKLSLLHGR-HKPYLDLRVHVNRVM-------------------PSWVGQKKHAWVNTGSS-----------VVCCKVRTLPPVDGK-----------FVTEYVH-------------------PTSRLLGYLGAEPLPAIIEG---DIPISSKIRPIHA--------------STPSFAEIGAGAGPIFFDQASFHVAECIPG---------------------------------------------------------------AEPMLAEQTIRS-------------------------FIRQKTAPVEP--LSIKVMVLAASSNLL     196032101       --MEKLKLLTFENIVEPLLNE-----SVSFIYFPIEWLDIVEIHYK---TFLLTSKLK----------RLNERLYDMFSDIL----FIQHNPYVL-----NENTPWIVSK-----EPIRK-EQLDYIFQSWYEI-----------IHDWKPN--------KLIESPKYEWHYDLISNL---------TVLHDKEVYSKWVPALISHIFCE---RPVQL-----------ENINEEDIYFSPLRT----QNICEAMSEP---------------IKDEK----------TQDYFAYVFRFEYITRGGE-NIPLLNVSIGIRRFYQEY-----NHPDISLLLRRKRGMILISTPEFASNNN----------KLRFVKLKVQQATNGI----------KWIKIF-------RNLKDDFHIGGE--VELEHILQYPKDYMLG--------TNLRVLLPYNER-------------IYKVQGTKIKPGIKVKEREYLFKAFQQKFAY---------------FTLIP-ECKKLETNNENE------------------------------IFPLIAPKGLE--------------------------TITLEIW--------SEKISLEVEQALF     229011883       --MEKLQLLAFKNIVEPLYNE-----KVSYIYFPIEWLEIVEIHYR---TFLLTSKLK----------LVNERLYEMFSDIL----FIQHNPYIL-----KEDTPWIVAK-----EPMKQ-EQLDYIFQSWYEV-----------IHDWKPN--------KLIDPPHLEWQYDLISNL---------PALHDKKTFSKWVPALITHIFCE---QPLRM-----------ENKNEEEIYFSPLRS----QHVSEAMSEP---------------IKDEE----------THDYFSYVYRFEYVTRGGE-NIPLLKVSVGIRRFYQQY-----NHKDIPILLGRKRSQILISTPEFESNNK----------KQRFVKLKVQQAVKGI----------KWIKRF-------RNLKDDYRIGGE--VKLENILQCPKEYIRG--------TKIRVLLPYNEN-------------IYKVQGTKIKFGIKVREKEELFNEFQRICPY---------------FTLLP-ECENVLTNNENE------------------------------LLPLFAPKGLD--------------------------SITLEVW--------SDDIVIEIEQALL     228927677       HFMEKLKLLTFENIVEPLLNE-----SVSFIYFPIEWLDIVEIHYK---TFLLTSKLK----------RLNERLYDMFSDIL----FIQHNPYVL-----NENTPWIVSK-----EPIRK-EQLDYIFQSWYEI-----------IHDWKPN--------KLIESPKYEWHYDLISNL---------TVLHDKEVYSKWVPALISHIFCE---RPVQL-----------ENINEEDIYFSPLRT----QNICEAMSEP---------------IKDEK----------TQDYFAYVFRFEYITRGGE-NIPLLNVSIGIRRFYQEY-----NHPDISLLLRRKRGMILISTPEFASNNN----------KLRFVKLKVQQATNGI----------KWIKIF-------RNLKDDFHIGGE--VELEHILQYPKDYMLG--------TNLRVLLPYNER-------------IYKVQGTKIKPGIKVKEREYLFKAFQQKFAY---------------FTLIP-ECKKLETNNENE------------------------------IFPLIAPKGLE--------------------------TITLEIW--------SEKISLEVEQALF     163940471       --MEKLQLLAFKNIVEPLYNE-----KVSYIYFPIEWLDIVEIHYR---TFLLTSKLK----------LVNERLYEMFSDIL----FIQHNPYIL-----KEDTPWIVAK-----EPMKQ-EQLDYIFQSWYEV-----------IHDWKPN--------KLIDPPHLEWQYDLISNL---------PALHDKKTFSKWVPALITHIFCE---QPLRM-----------ENKNEEEIYFSPLRS----QHVSEAMSEP---------------IKDEE----------THDYFSYVYRFEYVTRGGE-NIPLLKVSVGIRRFYQQY-----NHKDIPILLGRKRSQILISTPEFESNNK----------KQRFVKLKVQQAVKGI----------KWIKRF-------RNLKDDYRIGGE--VKLENILQCPKEYIRG--------TKIRVLLPYNEN-------------IYKVQGTKIKFGIKVREKEELFNEFQRICPY---------------FTLLP-ECENVLTNNENE------------------------------LLPLFAPKGLD--------------------------SITLEVW--------SDDIVIEIEQALL     152975657       --METIQLLAFKDVIDPLFEQ-----TVFYVQWPKHWSSLLLEHNR---PYELIKKFK----------LLNKRLYIMFSDIL----FIEHDPYKL-----AEDSLWIICL-----KPLSS-EQLEYICRQWYAY-----------IHNWKPT--------EMPGEVLLEWQPSAISKL---------SLLHDKKTFYIWVPALISHLFCE---RALHL---------SIGNKQYEEVPFYPLRE----QNRCEAMSEP---------------IQDKK----------TKEYFSYIYRFELITRGIE-NMFLLKVSIGKRRFYQTA-----------NQYANYMIMKKRKGTVLVSNCH--------NEKDKFVTLKIEDARFGV----------NWSKAY-------RELQNVFMLKKQ--LDLVKIMQSPKKYIKG--------KRIRALVIYDEN-------------IFKVPGTKIKRGISRREKEVLLQAFHQRFPH---------------CTYIP-LCKNVTINQNDE------------------------------LFPLYIREEMK--------------------------EITLELW--------SFQRLTEIERILF     117920626       QDQNIIQDLAMAFSPPVYPQV--LNVQVTKAYWTPHAMALFADIYKLAEQHFIKQGAKDKNVTLPTS-ALRNLLHGSLPNLLSTNQYLGLGPHSLYRK--DISLCGLFKQ-----NIHTV-ERFYACLDEWIEHDMTGFIERYDIPDHFREEMYGLA---DNTSLVTFTDDSLCLYPWISPHVDTAPSLGERLTISANDIAVLLEGEVIF---------------------PELPPVRRVAGHG----GNRAELVTLP---------------INDH-----------QEGRFSLVCEISVETLPTD-NTPIININFKKRRWLESL-------------NNDYTRALTKSGYLIESEGT----------RAYHFCLEKNQTNNWK-----------WTPDSAF-----SALERRFDLT----FTEALMFQEPSDQSDF----------IGIVHDYENE---------------GDQKNEIGAGVPEKDRKDAFDLISQILSK-------------FGFTPFS-AFERVKSPIKAF-----------------------SNVTYMSKLVKDKPTDIKLEAADSL-------------------YLGQPLPPSLFEYDDIDALTAGTKVYLP     331082037       MKENKLQLHAMEVRREALKEH-----KIYIYRMPSHLKDFLGKVKP---IKEYDWTGT----------VIKKVVILNFPGVL----FAFGKVYQIS----GNDNIWFVSM-----EESNV-ELLKIRTIEWIKK-------------SYEDNFNE-----KFPYDLDGDWGKCECVSM--------DTLYRYEALMYGLLPKYYGYRLTQ---KPIRM------------DTLGCELNFTLVMT---DGTDTELITSP---IFLGRKNQEAFISENIEEIVTDEETYYTDQPFSYYLDIRLKKCIDE-EYYTLHTTLHTRIWTGYSIINKNTGKNYLSGKQSTKVYLCRDSKYYNHVQP----------VYFEIDVKRGKKEKAW-----------WCDCADS---------CFLELEQIDIQEILEQNQAVRNFEMN----------GHALIIT------------------KFLKNYVSRGAGLPERKEMWERITECFPE---------------LTPRQ-PLNEIHIIGKTI---------------------------------LTTAKEVEDEE-----------------------GISEELQFPNIAINKKGYLYNGNCNRII     381168803       ANIDTLQLSAWVPTGQVEQLV------LTRYKMPTSAHEILAQWCR---EKTNRESDPI----TVVLQGLSEILATLVPDVA----FMKHDYDRVE----RGKRLHLFFLGDRSGDEALR-SKVQAAFSMWLGI----------LYPNKPGDIRASI---AASVADRANWVSFEVSTKLKEH--PGACAVPEDGMLYDALTAHAARALAG---KRITF------------RSGETKLLVLETAQ-ASPYEGLELVAFP---------------PKRDPNK--------PDCFWSEVITVSAATFPER-PGIHILARPSIRNWGTIS---------RWSTNSDPTRRMDVFIPGQMGDDDMAPCYQHSSFEFRPRQNKTKPERSIV---------ADWNHKEDE-----RVLDLVRRLSGSNKVDGGDVVS-PVANDQG----------LWALPRLGNV--------------HGDRYLPGGSGVGWFDRMDMANSLDGAFAE-------------AGFQRVG-ALARLRRSLGVE------------------------KPFGDKGEHVDRRKALAKALARLGNP-----------------NNHLDLFVLHQLDGTPTAVVEEIIKFFG     403070934       MKKRNYNTDSIEVFAIPIKEE---SIEGYAVYFPKALRELLQVKKP---KHFYMSPVR----------SLHETIKIMFSDFL----FFNSNAEK------FLNQPWLFFR-----APFDI-EWLKEMIQSWYKV-------------HFNEELPA-----SILNDLSIQKE----KRIVDITINEAGNPIMKDFFTFQYLPKHYADKIKN----PLFV------------ESLGKSLNFIPIQS----DHKGELVSWP------------PERYERNE----------KKYYFSYKITFSLQTVPFS-ETPQMYLHIGLVRWKTDG--------NVVTRNRRLTVYIRGKFPWIVGNED----------KLPFVPAKIKRVKTEG------KYTYFWDQKI-------DQVIEGMNIPAR-ISSLTELEKDAESIFEGIND----SNRFSVSIPYSTQ---------------LFGYHPISDGAGFKERYEIAKAAANEIG----------------LTGNFLNYPRVAGASKVY----------------------------SKGLELSLPKNFTIYM-----------------------YYSSKEWLEKAEEAILQNLKVEK-----     260588610       MKENKLQLHAMEVRREALKEH-----KIYIYRMPSHLKDFLGKVKP---IKEYDWTGT----------VIKKVVILNFPGVL----FAFGKVYQIS----GNDNIWFVSM-----EEINV-ELLKIRTIEWIKK-------------SYEDNFNE-----KFPYDLDGDWGKCECVSM--------DTLYRYEALMYGLLPKYYGYRLTQ---KPIRM------------DTLGCELNFTLVMT---DGTDTELITSP---IFLGRKNQEAFISENIEEIVTDEETYYTDQPFSYYLDIRLKKCIDE-EYYTLHTTLHTRIWTGYSIINKNTGKNYLSGKQSTKVYLCRDSKYYNHVQP----------VYFEIDVKRGKKEKAW-----------WCDCADS---------CFLELEQIDIQEILEQNQAVRNFEMN----------GHALIIT------------------KFLKNYVSRGAGLPERKEMWERITECFPE---------------LTPRQ-PLNEIHIIGKTI---------------------------------LTTAKEVEDEE-----------------------GISEELQFPNIAINKKGYLYNGNCNRII     219666713       -MAHYLQLNAFEL-GENTIPQ----IPLYQMEFPHEWTVLLKGIMD---GYHSPVKLT----------DLRAKLQMLFPQIL----GGYDNLLTE-----GQAYPWLIAT-----EPIQE-VWLERLTRNWLKN-----------ELLKKQKYGAKA---AEISPVNLNWER---YNL---------PIQWDNCVRYQVIPGLFTHAFCQEI-RYLNL-----------PGGVDIPLKFVQVFY----GKKHECMSLP---------------FLN------------NQPQFSYIIHFDVTNRGGEADRLLLNVRIGMRRFHTAP--VLANENPYELLNKKGTLLLGMSNPFVLTDTS----------LTVLSTLKFRRAKDSS----------ALAQWVSAE----EDLFFDV-LKEN--LSIDQFLASPLSYWSE-------SGKVKGYLVHSSQ--------------VYQNEPDVKTGLGLPERTALLDLVSRTFNL---------------KARLD-FLEKVSCGGRLK------------------------------HLPIKAPERT---------------------------FINLELW------MSPDMYKECVELLAQ     126656944       PRYNYFQPIYFEYDETLIKDI-----TGYVMAFPN--LEKFKLHYE---GEHKNAPTD----------SLLDTLRLLLPQIRVINKLTRFNKITKEY---ESNPEAFLAD-----EPIDP-KVLELIFRKWVEV------WYPESSHDKVK---------ELCTLEQFEWKK-----------------RTPEQLEWWSPAWAMGKLLSE---QEYEL------------GDNKVKLLFSPSRK----SNTVELVSWP---------------PLT------------TPRQYKASIGVVISTQSDL-NHKRINIHFKMKRWVVKR----GDQDEIGLQRNTTHCYIRKLKSWSRELNF--------IEPNAFTFLEAKNFEKEGSEDKKK----EFERRWKD-----KKIRAILDKLGVNIPDIEDVLLKPLNYFES--------DDLDILVPAR-----------------ASQKVGVGTGVPISDIRRLLKQITNFLPK---------------SVNLSQPWQKISKIENNE------------------------------IIKQQFKQASELIKE----------------------QFCEQPKIQKPSKDKLPGITETLKDFLQ     372487594       --------------------------------------------MS---ADHAMAFSG-----LQAVLGWAAPSVDLIGIAA----SSPGAQKTL-----TMTAIDPAQS-----ADEMR-REIEVAISIWLGI----------VLPEKAAEITA-----MLNDGRSARGTPCSVQPIRSALSWNGACASPADFALFDLISLIAARALEG---RVLNR-----------DTPDEGKLVLSGAQQ-SLYFGKALLRHEP------------SRVDRKR-----------STGWWTEVFNVAAVSTPES-RNLRVAINVGIRNFGEIH---------EARLGRNRARYMDVFLPADSALAP---------RSGRIRCIELATTRRDW-----------WNAGRGQTEKESADRRVLKTILGMSGLEFDEATLGLTPLLGG---------SVGLYPRFGTV--------------HGDQWAPGGTGVPHPEREEYLAFLDKHLST-------------AGFTRVR--MERIAKRGPKE-----------------------KLVTVLDNKPSDLKTALSRRFG----------------------SNDATLALLQSRPTGAEVFDDAIRAVLG     186681799       -MTKIILPGAWELTATNPTYK------LSAIYVPITWRQVANNLAQ---QRARLQGKSYRA--VPVY-SLDPLVVGSFPKIIKT--LRNGWQ--------KSSLPWLLAT-----ETTDI-SDLGNLIKDWLIE-----------EFSTLEDVESQL---ANLNNADWQWSNSQTYELLHP------QNKVEISNLYQAIPDYLAEKFLQK--PTVYF-----------GLDEQYELTFYRVVS----LQGAELMSWP---------------PSEVTVTKGKDTT--ETAYISFVIEFVLQTFPWR-EKPIVYHHLSIRRWLTKP--------LNYVPYPGVKAHIGDNRRWLDGQRQ----------PFCFIPLTMKGYGREV----------KWP----------QAISNLFSLNDSQLPDANNFVSNPNHNWSGFNTI---PSGIQAAIAYTS----------------KLGEPPCFAGVSPQDLASLDQAIEARLPVQRVGEAERVTGNIFIFWPLEKPKQKIVPLLADE-----------DIQSPKPKKAKKSDNPNHSHTPMLRPKLAAPAVFREAE------------------NPLRTILILWETPQCRNALIAEICQLLY     309791273       ETIQSIQPVAFHLIETGLDLP------LVAMHFPNEWRAP-LDHLA---ALSSERGSERPSS-FRIQ-SLNAAITAFTPQL-----FVSPGQAN------RSDAPWLIAT-----EEIRS-DKILRIVRAWMAE----------HYSTRAELADAYDMAFEAIRHEDLMWEPFQLSPIQEPYPN---NTARIDSLGYAALPAFVANALVK---RDVRI----------PVGSQDRRLVRVPVAQ-----DRAELQTWP------------PVYYEAENG---------RRAAYSYVIGISLQTLTGV-PRPRIHLTYGVRRWRLDP----LRTPDKLFLPGKEGRTVYLRHPRALTGVP---------QSSHFTRAMLDGVFVGE--------NQRVPVWR-------DALAGIASRIGVALPVPDDLTLMPDRFLTP--------AQDDAVVAAIVE--------------KTPRSHPVGAGMGLEVREAITSAITGALGD--------------LIELVP-PLIRSTVTGTPQ-----------------------------RNQPMLEPDLREIPADVRLQALRDSIGP----------EVVVEIWYQNQ--ACRDQLVESLHAILT     220930098       SRTDSVKVNKFYMSPIKPENDVIRIEGIFVIKSPETLNSKIKSLME---KQYLDKSRKYYKDYRAFKPKWFTLTVALLSDPV----LYARDDFY------TCSGEWLYLY-----ERIDI-ELFKLKFKKWCRE-----NTDADFYEYIKDDIEGIT---ISQENIEMSGNPHNLQK-------------HENELMYKLLPAYLAYKISGEMDIQVPS-----------SKKGAVSYRFYKVQN---EDKSAELAAVP------------FRKYVDRDG---------KEFYWTHAVDLRVVSIPGE-SIPYLIIKLRTARFAYKK---------------PYASNKRLKALIYKNIGK----------GFEIYELGLKMDQAKW----------KWKYNIAD--------GINDNLIEKIFDWQDTIVAEPNKFMSN--------DDLGAGIIYGSH--------------IKGKGHKIRTGTTEKDREYISEGILKAIRN--------------HLNNTNIEVPEFITLDNIA---------------------KEIDTKAKTQKAEFRFEQIPAELVITA-------------------FYRNDAWLEYL----KDALVELFDLKIF     392549087       -MSFGLKTTTMFLPVDAYGSV---TIYTASNEFTQAWERLKQKCSL---KYLPYA-------------SLANMLRWHFGHFI----RFSPFGKEL-----NGGKVFIVSE-----SNLDL-DTLAMMFDSWELE----------VCKYMHEPVLGQLIRNGAFRQHRVRIEDHIVVKT--------NSCPTADDWVWDAAKWSVARQFISS------------------QLAVDNNVKLELVMD-----SEARLLTWD------------NCLKSEFSD---------MPVRAMHVITPRLITVPGF-DLPAMHFESSISRLASTW---------------KDSNLSPIKTAWIEIGSD---------KPVLQTAIDIEKGTWKK----------RWRDISIQ----------LLNKNGL------EFIREPEEYDLS----------QGGNVRARYQ--------------SAPIKFPIGKGVGNPFHHYVSNHMKQTFPN-------------GEALILRKSVNRLPKRAKLA---------------------------EVQKSESQLRDIVSSTG-----------------------KSTLELICVYSSYIVRKRLLQSFKSIVN     254390288       ----MLITLAYRIPRENLADL---LGTVTAYPLTKEFAAVWGDLPR---TGRRSRQPYS---------ALSTGLVTATGQPV----RLFGEEDLDDGERATGSRMLLLTT-----DNALD-YRLRVAVRAWER-------------HIRKGE--------GTAELADFLPEPDIERSFAEFMTFRPGMVPTAPNWVFRTAAWQITRQLAG---EPLRV------------DGRRSPLALRMDTD-------GSLLAWD-----------RRDLIVNRS----------GSAFSMARVSARLITRAGV-DDAVVCFDAHLSRVSPQG--------------------HWAKHVWIDRGDA----------GKTVLRLPLLRSLDKQ----------TERWRS-----------DLNPAIVKILEACQLLPLSIPDTLPD----------VPDLIRPHMA---------------GSRFHALGSGPGPRFMLRLHEHIVRMLPG---------------LVPLTYETDRRIKLLNPV-----------------------------KRYPVGGPPAAGVGSTG---------------------YERVTLV-----------CVYSTPEAR-     302559389       AAPTRLYTADFPLSPDLMGQV-------WLYPLAPEFEDLWEICER---QWDPEEGFRTPHG------ALRVALGTVTGRMIVF--TVARRREVRP----GEERSLIVAD-----GPLDP-RLTTRCFDVWQKL------------HFRGVRD-------GQFLSRHIDFERAVCRPL--------SDVLKRDEAGYVIGPWWWKDAAGWAVIQRLAA---------HPMVDKADPAQRATTFRVGLGHDAKRLVAWD------------YPYYRTIYQGRSNE----RTGWAMAYVSVTAGTQRGL-PDPVLRFDCHVTRVADHW---------------SNVKTVTLNHPQFDTLLR------------VPVRHVPQRDPDGE----------KLRDKN-------GRLIWKTEFRGHTAAIVQACGLTPVTLPDKA-------DGDLERVRAVFR---------------NKGSHLVGKGVGAYFTLRMATHIADVL----------------GVQPITYDKSRYTIPGATI---------------------TRGPIPAKKLPPALEAAGWK--------------------------SMRLVVVHAAA--TTPRRVMRQLRLDYG     407881735       ----MLISLAFRVPAKYLDEI---FGAVTAYPLTEEFSEAWSAMPR---TERGWGPPYA---------SLARGLTSATGRPVTLFDEYDLDETERA----SGNRMLLLTD-----DGAMD-YRMRIATNAWERY-----------VRGGA----------DVSTLAPLLPEPERARPFSEFIRLRDGEVPEAPNWVFRTGMWRIMRTLGE---HPLVI-------------DGREPLQLRMDTS-------GALVAWK-----------DDDLIANRK----------GTAFSMLRVTGRLVTRAGV-EDPVLCFDAHLSRISPKG--------------------HWSNNVWIEPVDA--------GLPILHLPMQYRKDKELG-----------------------DRRVTLHPATATIYEACKLDPLDIPEELPD----------RPGVLRPQRW---------------RSRFHSLGSGPGPRLLMRLQEHISAELPQ---------------LKPLEYPIDRSIKLAPRV------------------------KKYQKGGLPVAAVGASG--------------------------YKRVTIACVYRTTGARQRMLAELKELTG     210630742       GTQDTSSLHLMSLVPDSSFTA-----TYHLLDMSPELHDALIDLVG---DARMAHNISNR--------ALDDQLLQYLDQPV----RVKPVSRFRN----DRIPGWLTAN-----APIDL-PRLCAILTNWVAS------------VCPKSKFGDAD---YSRVVNLITPNELARHTRSESIALFDEDRRPAGNLTFTGFSLSMSDRIDG---KTCHL-----------SCGQDLNFERILSSD----GSPCELLSQI---------------LWH------------DGFAYAICLKLSTQTVPPR-QEARLNVKASIRRFAQ-----------------GTWTFGKGLEPYLQSDVN----ALVRWPGSRWCRVPYGYDPKGK--------SIDWNR---------ASARNLKDFAGVELPDVIDYLQHMNDWTQP-------GRELQILSPQAVA-------------ATWQTSHKVKAGLTINDRAELFEFVASCLEG-------------IATPSPDPVCERFMN-----------------------------------LIKLPDEKKLKT-------------------------DDGRAAWVLANRHRLAAATGSQRIELEF     158336199       GQIKKLRDVALAFTVPDDLPP------IHASGVTLVWTRQALKSFS---DIHKAAYGEEEKYKELPYQSLRGLLNVLVKEIDRIENHLCLKSTLIREIRSSNPQNYDFARLIDSDNDEELSKRLRLVLNNWITNDLRPFCEKKDIPEKLLEQIRELA---EKGNLIHSRPFEMTLLPWPWQQK--TGTTKADHGRAYTLVVDYIARLIAG---QEIFK--------------GCGPMRRVISSYGKFMSSTAELVTTP---------------ISIT-----------NKGRFSLVVKLQLVTFPSV-HQPVIEIDVSKRRWLDSL---------EAPDRYRNDIFGYAFSPDLPGRTF------------SFKVVCCKENKEWS-----------WKTAKDF-----GAIRNQLDLGMQAFSGQDIVLKKAD--YGG----------SQLALTHRN----------------GLEKHGIDVGVPETDKLEAYVNIEKLVRS-------------IGLVPFT-DYEVVKNKSSGR------------------------QKEKEIKLPTLLSAMLEAQESESVNFTP---------------DYVNKLSDNQLEKQMKEKFCLSLEAIIA     406692971       -MYKNIRRSAYHLAEEGTPWT----EDFHALPFPEHWHAGLLELHN---HGRDEEKRQPT---LPTR-RLDGVLQTLAPDVIVR--PRPRIPVEPGPQ--AAEDFWMYVPTSAP-GPLPG-RSMQQLLDAWLRT---LGPKDSAQDPRFRSLLLASS---TELKQKLPEWQPVSGVELLTTPTTRGGTAAPEPRQ-FQLATDALARRILT--LDPFPF--------------EGGELRFRALPR-GPRDQGAELMSQP------------LCRTVKR-----------KEWWFSVLLNISLHTTPFD-PRPRLHLHWGVRRWATHP--RATTKRLNLPYREATTVYLRPTIPWLPGAPA--------TERYALARLRRDRAADTF----------VWSENDT------AGILRGLSLAGN-FPDPEQLLTEPVSWIGE-------GRGVRAAVVHST----------------RMGKHEIGTGFMPNQRAQLTEWAEQALPE--------------TLARVP-DLTRGRGKGIGA-------------PQNRRPKPRTDEAKKAELLRETQARRVALAAQARIASHGPEQAG----------PPVVEARLLWQSPEVRGEAVEQFAKALG     172039225       MVYQTISALALKLKADLQSQT------YYRLQFNNSEEAIHYLKLL---TAQRNKQSVEKTT-IPSK-SLYQALR-LLPGLI----HIGQLSS-------YSGNYLAYSP-----QPIDK-TYIRAIINCWIDLEFPDNPTTKKKQGITAEERDNAK---SFFSENNLAWFEENITFNNKFKTHPNGTATLANDD-FILLSYTMAAELTK---PECTF------------IVDNQPLKFYRTTS--SKFQPIELISWN---------------PIKATIGE-------EIYYYSLVLTIKVETIPYQ-SYPEIHIKPSIRRWLSLP-------NSRLSHNHSSNAFILTDLQWGKSTNPNNFSKCFVSYLMKMYKQTIDGESQWI---------PNWKNRNRI-----TQLLTELNTLS---ATPQEILSNPVNYLQS-------NSKESIGVVFKEG---------------MTPQHQVGKGLPTLNVKELYEQINNIGFVK------------NHFETISYQRETYDVSKQSQQYFDLAKPKKSFDEPIVKRQPETEDQFEARKKKLEQKQQTKLKNCKKKKKESDEQYKDRKQQLIKKQEEDLEALKPKSEEDRKKEFTAELEAFKQ     375092908       PKYEDLKLAAFQL-RTDVPWE----RRLYLLRFPEPWKKPLTTLAK---ARRNGEEIRS----IPIT-LLNDVIAALVPDVI----TVATRATI------GENAPWIYSD-----VEVNT-ESLFAIIATWIRA-----------TATDPAKAEAIL---GRLNQSDLVWTPLD-VDFTTLTSDSADEIQSDE--LYRLLPHVIAAELSAPGLQHVHLMPKNPDADPADDDPADVEQVISQFHR-TPAEQGACVMNWP---------------PHRM-----------PHRPLSYMINITAQSRAFN-SQPLVHISVGTRRWAHKE--------AKLSFNQGHSVYMLPSLPWLPGLNQ---------SRSFLVASIESWKKDAPNTDQGFEYSARWSGGKI------GKILRELGLAEK-LPDPEVLKSKPTAYLGA---------PNAAALVFRNG--------------MYSFDHPVAPGTSLADKVPLVEWVTNTLAD--------------RLVPVE-NLRKGSQVFLDS--------------------KKLKESKFAASEAAQLRKAIADTVGE---------------------TLGVDIFYDTT--HTKNYARATLAKLLG     302867037       MRYDRTQTAAFVAAGDLKIPL-------HTLTFPISWRRP-ILDLR---TAGWREESRARAQQVPIG-RLNALMRTVAPDLV----STATWATL------DEDNPWLFAA-----KPFPP-AIMRTFINAWLYD-----------LPTTDDGKALVMPTIRKLDSGTLRWTQKT-VDLLAHELSDGNTAVPQPHL-FSLLPDYAAAKIAG------QS-----------YQHGSRSIRFRQVAA-NPANGYAELVSWP---------------PLEHH---TGRGSNIRTWYYSATIKIALRTKAFD-PTLRLHVDTGIRRWTSGE--------LHTKGRWGASTLLQSTSPFVVDAPT----------SQKFAVAHLIWDRDTR--------EMGWRNGGT------EQILNRIGAVDN-LPAAEQLAGKSDAWILG-------RDGVTAAVTYHTT--------------MRGPDHQVGTGVMPAERSRLIRWIGQCLQP--------------DFDLDQ-PLTRVLIGGKAS----------RSLSKLESVPKDKKDQTDAERTQLVERQEAANAANAEISKANALVRRQQLAAAVDG-RLSVVLLYQGDGKGMRDRLLAAVERLLD     357393639       -MYRTIRTVAYEPDPAHGAWQ----EPLRVLRLGDGLHAELLRRRP---PAHGEDRPAR----LPVR-HLNSLLRATAPGVL----ATGREAGV------DGRLPWLYAR-----QVVPP-EVLAPVLGTWAAG---LTGPDGEDDGTLEEELLADP---AAAAVSLPPWEREH-VDLTETVISAGGTAEPRQRL-YNLLPEAVAFRLAE---SPYRT--------------GGTSLRFRVVSS----GKGVELVSWP------------PQQYERRG----------RTWYYSARLTVTVQTVPFA-PRFRVYVATGVRRWATHL-------EAAPRALNGATVLLDAPLPWPENQDR--------GHRIAENALGFDRRAERL----------VWRRRSP------ALLLPELDIIRR-YPEPGELFESPEKWLTG-------HRGLAAGIVYHP----------------VLGPHEVGPGLMPRERSELDAWVEAGLRP--------------MLRRAG-DLTRVTRHNTPS--------------LLPRSVKRTEPDTREAQKAMLRRTALARALHGR--------------------PLDIEIVWQTP--RTRDALLSALPGVIG     302870454       -MYTLIRPTAYEPDPAAGPWL----EQYHVIRFAERWRTGLEEVYR---LGWKRRQNHVG---LPLR-QLNGLFRAVAPGVT----ATGRGAGS------DATVPWIYAR-----EPVPA-DVIRPVISSWVTG-----------FPIPDEHQQRLADVVETAHKSIPEWDVAQ-VDLTTVQPSPEGTADPSRQL-YALVPEILAARLAA---RPLRL------------PGVGRDLWFRVVDR----EQGAELVSWP------------PQEFVTNR----------RTWFYSAVLTITVQTVPFV-PTYRVHVSSGIRRWMTSG--------TDLSHNQTARVLLDVPLPWSDLTEK---------GTSRLIANRVGWSRDRR--------RIDWRDHSV------AGLLPDLDIVRT-YPRADEVVAQAPTWIKG-------RGGISAGIVHQN----------------RYGTHGVETGLLSGERSLIDGWVEEGLRP--------------LFRRAP-ELHRVREANKPT-----------LISKPAAKTNPAGHAEAVRKAVAARREALKTSLA----------------------GEPLGVDLLWQFPETRHALLSELAELLG     357392957       MKYSNVRLAAYQPHPGAAWNA-----EYHTLVLPRHYREALLALYR---RGLKRDPEQCRS--LPVG-RFNSLLQSLLPGIV----SVAKWVDV------EDEAPWLYAH-----GPVPP-EVFAAVFTAWVWD--------LRPDSVPRREVRQLI---AGLAPATLRWERGK-IPLLAGGANTVGTAQPDPRL-YQLLPDAIAQQALQ---LSPFK-----------HEKGSLSLSFRTVAR-RSFERGAELVSWP------------PDLHVDKR----------GEWFFSAVIGVTLQTVPFH-PLPRIHVRTGVRRWATGT----GRNGLFLPARRATSVFLKPSAPWLKGTSP------QLAGSFSVARMRYDRQAKAV----------AWEHGGP------TGMLSRLTLEGD-FPEPADLLSDPARWLRG-------VSGLEAAVVHST----------------AMGSHGVMPGLMPRDRVPLTEWFESAFPD--------------AIGRLP-DLERVTKHRPAP-----------VNRARSLPADEVGRSEEKARRARQRREGLARLNG----------------------GRTIDLDFLWNTSEIRIAMVKGLEELLG     269125748       PAYTRIRTASWLPDSPDASVT----ARYQALPFPEQWREVLLELCNAGRPTDAEPYRT-----VPTR-RMEQVLQTFAPDYL-----VLPRPR-------DGRGHWLLVPEGV--ERLPD-QVFRALYNAWLSD----LRPDMAKDPHYRELLAKAR---AMLDDAPPRWEPVE-LELLRCPVTEGGTAAPLAHQ-YPLTTDWFARKILA-----LEP-----------YDYGSGTLRFHAVPR-GPRDQGAELVSEP-------------LRFDKDG----------QRWWYSITLNVTLHTVPFE-PLPRIHLHTGIRRWATRV---GAAGRLYLPRRRRTTVLLRPRVPWLPGASR--------SDRFAVARLERRWDREAR----------DWVTGWVEG----GPAGMLHRLSLSSFPDAEAIVTAPEEWLAD---------DMSAAVVYST----------------AMGSHGVLPGLMPHQRSELVAWAEQAFAP--------------ELRPEP-ERVRTRLGRSTP----------------------------LNTPPKTKTSEYDRRRAAKVRAAAAHAMRALGAVEDD--RAVLEAWLLWQTPQMRDEAVEAFIGLLG     126655716       QKYIQLGDAKTTATIPLVFTVPNDLSPVTVDGWTISWTSEALANFS---KILEDIKQIKNLPYA----SLRGLLEVRLSNVTRIESDMGLSKSAINSN--KYRNPFAYLEGGD--KEQIL-KKLKPVLNDWLENYFASYIKKEGVDENIIEQLRELQ---EDNLLLSIEPFQSQIFPWLQYK--GSGTTKPKDKYSYPALADYLARLIAE---HDIFT-----------ELGGIKRIITSKSGN------SVQLVTNP---------------IKLE-----------NKGLFSLFVDLEIVTFPSL-PQPLITVDIGKKRWLSSL--------------KENSFDFNAINGYIFSKNH--------SDRIFNFKLNRRQNKETN--------QYSWQPDSSF-----AALQRELNLPLN-ISNGQQIVQNSASTKD-----------CQVLLTYRNG--------------IQEKNHDIKAGVPEKDKLEAFEAITQILAT-------------VGIKPFK-DYSKVKFRKGMAHSNNIAGSRNINTPTSVNTIFESLESKDVSDSEKKSPDEMSHQEINNLLKEYFDF------------ELSEKGIKSLKLNSNKKNQTKELKQLLS     345850902       -MYKNIRRSAYHLAEEGTPWT----EDFHALPFPEHWHAGLLELHN---HGRDEEKRQLT---LPTR-RLDGVLQTLAPDVIVR--PRPRIPVEPGPR--AAEDFWMYVPASAP-DPLPG-RSMQQLLDAWLRT---LGPKDSAQDPRFRSLLLASS---TELKQNLPEWQPVTGVELLTTPTTGGGTAAPEPRQ-FQLATDALARRILT--LDPFPF--------------EGGELRFRALPR-GPRDQGAELMSQP------------LCRTVKR-----------KEWWFSVLLNISLHTTPFD-PRPRLHLHWGVRRWATHP--RTTTKRLNLPYREATTVYLRPTIPWLPGAPA--------TERYALARLRRDRAADTF----------VWSENDT------AGILRGLSLAGN-FPDPEQLLTEPASWIGE-------GRGVRAAVVHST----------------RMGKHEIGTGFMPNQRAQLTEWAEQALPE--------------TLARVP-DLTRGRGKGIGA-------------PENRRPKPKTDEAKKTELLRETRARRVALAAQARIASHGPEQAG----------PPMVEARLLWQSPEVRSEAVEQFAKALG     257790573       MSDSKLHLMSFAPVPDTKTTV-------HVVGLPDSLRDALFTLMP---PRKEGGYLNTK--------VLKDDLRCWLDRAV----ELNPVRPN------VHTDSWLIAL-----APIDL-AKLCNVIAVWISS--------RKDIDTQSPAYRKVM---GMLHPETFE-EAVRGEEI----CLFSGDGRPAGGLTFPAFSAQVADVIAG-----IPL-----------ELANGMVENFSRVSR--GNGNVYELISDI---------------HWH------------KEDPWAFALRFHVETLPVG-RKARLNMDVAVRRFIGKP-------------WQDDPFLKHDVNAYVRTEGG----------TLRVVPYGYDKQKRDL----------AWDP---------AALANYEFASGTGLPAVREYLEDMGRYARD-------GSQPQILSPYAMT-------------ASWASKPSVASGASVIDKAMFFEAVAARLKD--------------IAEPVG-ALDSLQLTHLKA--------------------------------SIEEPRQADWDKDPVSA------------------RARQEAWGRAN--RARLARCTGRDRAVF     295106506       MSDSKLHLMSFAPVPDTKTTV-------HVVGLPDSLRDALFTLMP---PRKEGGYLNTK--------VLKDDLRCWLDRAV----ELNPVRPN------VHTDSWLIAL-----APIDL-AKLCNVIAVWISS--------RKDIDTQSPAYRKVM---GMLHPETFE-EAVRGEEI----CLFSGDGRPTGRLTFPAFSAQVADAIAD-----IPL-----------ELANGMVENFSRVSR--GNGNVYELISDI---------------HWH------------KEDPWAFALRFHVETLPVG-RKARLNMDVAVRRFIGKP-------------WQDDPFLKHDVNAYVRTEGG----------TLRVVPYGYDKQKRDL----------AWDP---------AALANYEFASGTGLPAVREYLEDMGRYARD-------GSQPQILSPYAMT-------------ASWASKPSVASGASVIDKAMFFEAVAARLKD--------------IAEPVG-ALDSLQLTHLKA--------------------------------SIEEPRQADWDKDPVSA------------------RARQETWGRAN--RARLAHCTGRDRAVF     392942503       ERYTAIRRAAYEPAGDQHGLT----VGFHTLEFPEQVATRVLDLYN---LTRKGRARAL----VPPTSRLDSLLQALDLRLG-----VLPRAAAAATA--AEPRVWLYCPVDQPGGPLPV-PVLLRVLDYWIEE-----------LQPEPEHAKAVAQVCDALRVHAPVWRRQD-VPLLGCDLSSGGTAQPTD-VQYLLTTQHFARRIQE-----LTL-----------YDSGEHALRFRSVAR-RARSQGAELMSQP---------------LHHEGKKG-------LRWWFSVVVNVTLQTMPFH-PRPRVHVHFSVRRWATHP--DPKTGLLRLGFRRGSAVYLRPLQPWLPGVPA----------SDRYSVAHVVKRGDEH----------MWRTGDP------ALLMKRLASSNRLFPELASLLSDPQRWFGG-------DQEVDALVVHNT----------------HMGEHGVGVGYMSHERSRLVEWAEQAFAP--------DLVRVGDLTRSPLPAAKPTNARQGG----------------------SSERTAAAKIALQRSRRASLALLCTEWSG----------------APEFAARLLWRSEQVRDAVVAALATVLG     290956757       PRYDHIITPGFVARRGATLPI-----TVHTAKFPPSLLARLERAWN---SNPKARSPY-----LPTY-ALRELIEQVEPAVL----AVEGR---------LGDGPWLHAMR----MPHNE-LPLQLALEFWITT-------------HVAPHQNDVDWPTMVKRALPLEWAQAE-VDLLAHGNAANGTATPHSST-FSLLATYIAGCWVD---EKLSL-----------PGHTAKGFSVLGQLT---DRGERSIYSWP------------PRELDDDG----------AYGLWTHRTALRVVSMPHD-GRLILRAVPHIARFGGTQ---PAYIPRRGDTPATATILLYLAKGALRDVER---------PMLLRAPVTVSGKKEDM----------RWQWQPGI-----ARILPSLPASNR-YPNPEDVRTDPRRAAGVNRSAEEDKEPTALLLHATGYTYLTRTDLADVQPEFSTHGHPAESGLQPIDHLTLFEHLKEPLAK-------------LNFDPLP-ALDRTPQR----------------------------------RAPRLTPTREDA-------------------------HYHFELWHCAP--KTYQAIHLALTKLLN     consensus/100%  ..................................................................................................................................................................................................................................................................................................................................................................................................................................................................................................G........h...h......................................................................................................................................     consensus/95%   .....................................................................h............................................................W........................................................................h...h.............................................h..........................................................h........h..........................................................................a..................................................................................h..G........h...h...h..................................................................................................................................     consensus/90%   ..........h.....................h....................................h...h................................hh...............h......W.....................................................................s..h...h............................................phhs....................................h...h...h...........l..p....bh..........................................................................W..............................h...................................................h..G.....b..h...h...h........................................................................p.........................................................     consensus/85%   .....h....h.....................h...h................................l...l...............................hhh...............h..hh..W..............................................b......................s..hs..h............................h.b....p........phhs....................................h...hp.ph.p.........l.hp...pbh..............................b......................h......p.............W..............................h...s.p...s..............h..........................l..G.....b..h...h...h..........................p.............................................p.h...............................p.............h.......h.     consensus/80%   .....l....h.....................hs..h................................lp..l....s..h.......................hhh...............h..hhp.W..............................................h..................a.hhs..hs..h............................h.h....p........phhsbs..................................h...hphph.s.........lphp...pbh.............................sbh.s.s.................h......p.............W..............................h...s.p.h.s............h.h..........................l.sGh....b..h...h...h.................h........p.............................................p.h...............................p.............h...h...l.     consensus/75%   ...p.lb...h................h..h.hs..h................................Lp..l..h.s.hl....................p..hhh.........h.....hp.hhp.Wh.............................................h..................a.hhs..hu..h.......h....................l.h....p.......splhsbs.................p.............p..hs.hhphph.o.s......blphp..hpRa.............................sbh.s.s...............h.hp.pb..p.............W...................h.....s....h...s.pbh.s...........phhl..........................l.sGh...pc.bh.p.h.p.hs................h......hpp.........................................b...p.h.............................p.ph............h...hp.hl.     consensus/70%   ...p.lb..sa................h..h.hs..h...h....p.......................Lp..l..hhs.hl.....h..............s..hlhs.......sh...p.lp.hhp.Wh................b..p..............ph.........h.........s.....p..a.hhs.hhup.h.......h....................l.h....p.....p.scLhobs.................p............pp..ashhlphpl.o.s....b.blphph.lpRa.......................h.....shh.s.s..............bh.hp.pb..p.............Wp............h...b.h.....s..ppl...s.phh.s...........phhl..........................l.sGh...p+.bh.p.hpp.hs................h..h...hpc.........................................b...p.h............................bpbchh........bp.h...hpphl.     ```      **- 2) Y+zinc\_ribbon+REase**       ```                        Y domain------------->                                                                                                                                                                               |zinc ribbon--------------->                                                                                                                                             | RE-like domain------------>     FINAL           --HHHHHHHHH-HHHHHH----------------HHHHHHHHHHHHHHHHHHHH---------------HHHHHHHHH-----------------------------EEE---------HHHHHHHHHHHH-----------------HHHHHHHHHHHHHHHHH-----H-----H---HHHHHHHHHHH--H------HHHHHHH------H----HHHHHH----------HHHHHH------------------EEEE----------------------------EEE-------------------------------------------------------------EEEEE---EEEEE---HHHHHHHHHHHHH---------EEEE-----EEEEEEE-----E-----EEEEEE---HHHHHHHHH----------------EEEEE----E---------HHHHHHHHHH---------EEEEHHHHHHHHHHHHHH-------     ALIGN           ---HHHHHHHH-HHHHHH-----------------HHHHHHHHHHHHHHHHHHH---------------HHHHHHHHH----------------------------EEEE---------HHHHHHHHHHHH-------------------HHHHHHHHHHHHHHH-----H---------HHHHHHHHHEE--E------HHHHHHH------H------HHHH----------HHHH--------------------EEEE-------------------------------------------------------------------------------------------HHHHH----EEEEE---HHHHHHHHHHHHH---------EEEE--------EEE------E-----EEEEE-----HHHHHHH-----------------EEEEE---------------HHHHHHHHHH---------EEEHHHHHHHHHHHHH--------     HMM             --HHHHHHHHH-HHHHHHH---------------HHHHHHHHHHHHHHHHHHHH---------------HHHHHHHHHH---------------------------EEEE------E-HHHHHHHHHHHHH------------------HHHHHHHHHHHHHHHH-----H-----H---HHHHHHHHHEH--H------HHHHHHH------HH----HHHHH----------HHHHHHHH----------------EEEE------EEEE------------------EEE------EEEE----------------EE---------------------------EE----EEEE---EEEEEE--HHHHHHHHHHHHHH---------EEEEEE---EEEEEEE-----E-----EEEEEEEHHHHHHHHHHH---------------EEEEEEE-EEEEEEEE----HHHHHHHHHHH--------EEEEEHHHHHHHHHHHHHH------     FREQ            --HHHHHHHHH-HHHHH------------------HHHHHHHHHHHHHHHHHH-----------------HHHHHHHH-----------------------------EE----------HHHHHHHHHHHH-----------------HHHHHHHHHHHHHHHHH-----HH--------HHHHHHHHHEE-----------HHHH------------HHHHHH----------HHHHH---------------------E---------------------------------------------------------------------------------------------HHHHH----EEE-----HHHHHHHHHHHHH--------EEEE-------EEEE------E-----EEEE------HHHHHHHH----------------EEEEE----H--HHHH---HHHHHHHHHH---------HEEEHHHHHHHHHHHHHHH------     PSSM            --HHHHHHHHH-HHHHH-----------------HHHHHHHHHHHHHHHHHHH----------------HHHHHHHH-------------------------------E----------HHHHHHHHHHHH------------------HHHHHHHHHHHHHHHH-----H-----H---HHHHHHHHHHH--H------HHHHHHH------HH----HHHHH----------HHHHH-------------------------------E-----------------------------------------------------------------------------------EEEEE----EEEE---HHHHHHHHHHHHH---------EEEE-----EEEEEEE-----E-----EEEEEE--HHHHHHHHH----------------EEEEEEE-------------HHHHHHHHHH----------EE--HHHHHHHHHHHH--------     260588608       -MDEMSLLYLL-KQSTEIKQRK----------DVREQVKKAMSALQALTRYLYENGIQE------IPSNLCEYYQFLEERPLCDYIT-----CIKSVEP---FYDAGIVNKEVEELFQEENLEEIQQNAIV-----------GNGIRFFREKLQKLEEEKSEDTW-----IYIKECE---EEYVRFRLFLS--ETEFRDSISMEYDI------ANLNVPAEIVQ----------ILKKSFSCKKHYGK----------DVEV-CPVCGKPIME--------------KRENICDNEVCNWYIQK----------EKLEPKT---------------------------IKLQGKAMELLPGIYRFCLSPGIAERKIYQKLKDLYG-----ADHVFLYPHVDRYDIDVQ-KNGKA-----VYLDIKDVKNPLRLFKTLTETSDLSKLFYSKENPVYLVIPEHRISIYTQTNRNLSYIEQLRSYLT--EAGMDIRVIREARLPVMLNSEWRCQDEPLS     331082039       -MDEMSLLYLL-KQSTEIKQRK----------DVREQVKKAMSALQALTRYLYENGIQE------IPSNLCEYYQFLEERPLCDYIT-----CIKSVEP---FYDAGIVNKEVEELFQEENLEEIQQNAIV-----------GNGIRFFREKLQKLEEEKSEDTW-----IYIKECE---EEYVRFRLFLL--ETEFRDSISMEYDI------ANLNVPAEIVQ----------ILKKSFSCKKHYGK----------DVEV-CPVCGKPIME--------------KRENICDNEVCNWYIQK----------EKLEPKT---------------------------IKLQGKAMELLPGIYRFCLSPGIAERKIYQKLKDLYG-----ADHVFLYPHVDRYDIDVQ-KNGKA-----VYLDIKDVKNPLRLFKTLTETSDLSKLFYSKENPVYLVIPEHRISIYTQTNRNLSYIEQLRSYLT--EAGMDIRVIREARLPVMLNSEWRCQDEPLS     158336201       DAVVLLAQGIL-KQYTRVLNQE----IVRQNDSMPSELYRAMCKLGR--LYIEEGTPDK------AACVHTVLDRARHSFRSEDWRL---KVFSQNDFR---FSDVVLIDPD--LYVPTSDCTEVA----------------QANGGWGEDNVVEYRLYQRLKTY-----TEQLGTRRQHLGYTALRELAV--RHSLIGEKALWDYLE----DRQLLPLQER------------IVDTFFDQIPESWL-------IKGKANR-CAYCGTLMHP--------HPNDQRFKEGRCPIRQCNSRYT-----------PKVSEAL---------------------------DPEQHRLLVAKPQILTYWTGPGIDELAIFDTAKQQ-------GLRAELYPESDLCDVSL---NDYE-----IGIDAKSYISPVSLALRLNQSIGG----LVYYQRRIIAISDQLIA------DNPSYLSTLQSTLS-----KKGDPATVEVLPVSAVIKLLQEVAYAS     117920628       LTARMLAKGVL-KQYLLVEHGE-----LVDGTQIPIELRQAMCLLGK--LRINNGKIDN------CASVHEVLSRCIKPFGDDEWGI---AEFSSNSFL---HRTVSLIDPD--HRCPTFDCIELA------------------DIRSDADLREKYSFEEFSQLC-----TSFGAMR--HQVYSQIRRFVA--ENPIVESSVLLDFLK----ENRIQKADRF------------LRQACYRSLQEADL-------INRRLHV-CKSCGVPIKM------------ERHGVGRCSIKQCSQFSVD----------QPLSKN----------------------------VELTSSLLILKPHLLIYWFGPAIDELKIFYQAEST-------GLSCSLFPNSDQCDISI---DGYD-----VGIDVKSYQSPHFLAQKLSGSIGG----LSEYHHKIIAVNDNLIH------HQREYLEILARYYR---GNVKLEFMSVAEVVRWLEDM---------     210630744       QLRNQFLETVF-LIAAGLRKREELFAANPRRYPYCNELRLGIQRFAA--LHAQHAEDNQALRNLLDGLSESGFIRTWCTRDLSTWIEQWTPAAREELGNLECLELGPFAEVGGSFFNITGDCLEFLRL-------------------NGNGNTDDFHENRVYSML-----RDAGQAA-----YVFGRKFLI--LHPTLSWEERTAAAHANLAVLEVDQLDQGEVAHIDGAWVQELVDTAYERAPLGLK-------------V-CPVCGWTMSL-------------HGLRPMCLDARCTVRVDA----------DEYENL----------------------------PDVPPNSYRLRRGVMRFIAKPGILELQIAHKVQAF-------GLPFELWPLLDTCDILITLPGGHT-----IAVDAKDHHDIDRLVRSIESDDMREA--MAASEAIY-VVPADTPP----------PRRRAANRRL--AHKPGYSIKTLRELTQHLKTEMGDA-----     257790575       DIANRFETAVF-LIAYGIAEKDGLYEADPHRYPYSQAFRHGMNILAA--LCAECSDDAE---ELLPTFNESDFIRNSAASDVREWTARWRDECREAVEGCRSIEIGPLASVDGDYFAATSECYEVLRFA-------------------ENDLLGGHQERRVYEFL-----RAGTQEQ-----YVYGRRMLI--RHPLLTWNEYVRIKT-GLALGDPDPLDQGEADTIDPVWLQEFVSMAYEPVPGAAK-------------V-CPNCGWTMTM-------------RGKQPHCSSATCAKAVT-----------GDFDKL----------------------------DSVAHDAFRLSRGVMHYISSPGKLELAIAEAAAGL-------GLKYEMWPLKDTCDILIHLPDGRQ-----LAVDAKAYGRAERLAREIEDDTGIAQ--MCADEVAY-VVPDQVER------DHPGYRALCNAVLR---GKTGYSCETLRNFSKRLDAAAKGGTR---     295106508       DIANRFETAVF-LIAYGIAEKDGLYEADPHRYPYSQAFRHGMNILAA--LCAECSDDAE---ELLPTFNESDFIRNSAASDVREWTARWRDECREAVEGCRSIEIGPLASVDGDYFAATSECYEVLRFA-------------------ENDLLGGHQERRVYEFL-----RAGTQEQ-----YVYGRRMLI--RHPLLTWNEYVRIKT-GLALGDPDPLDQGEADTIDPVWLQEFVSMAYEPVPGAAK-------------V-CPNCGWTMTM-------------RGKQPHCSSATCAKAVT-----------GDFDKL----------------------------DSVAHDAFRLSRGVMHYISSPGKLELAIAEAAAGL-------GLKYEMWPLKDTCDILIHLPDGRQ-----LAVDAKAYGRAERLAREIEDDTGIAQ--MCVDEVAY-VVPDQVER------DHPGYRALCNAVLR---GKTGYSCETLRNFSKRLDAAAKGGTR---     186681797       LSVTDTLRYLA-TGVVQWEKLE----LEKRS-QVPEALRIGMSRMYL--TSLLR-GEQA-------PENLPQFFD-WAETSITSWKP---AQEIKYLSP-----DVSVIE----DGVVSDFAREWQVS-----------------GNDVVAQVQESVLKDVLEYC-----RGNQLED----AYRAFRLLII--SKPTLHYSEYRQQ------LRLPELKPLRE-----------LLSRLEIYVDMDKL------AEGSVYHL-CPRCTYLQRQ------------RPDGTYTCRNPWCERLCATSNF-------QPLPPIS---------------------------KEKAADWKAVTPGVHLYGTLPGIWEIQLQEELTKL-------GLRVTLWPFVDEFDLLVEFPPKVR-----WAIDFKDWASVDEERLRKVQYR------RDATETFV-VFPDERED--------YLRIKVVREQLE--PELEGVRLRLFSEIITQAQAILGKKK----     381168806       TLVAMFASALL-KVQAQIDE------KGFLSSPYPAELRRAFDLGAA--RNITAKTASW-------PTDLDRLMA-LAKEPLFRWVP--DMSWDKADEF----FAARLVE----DGEISTDCRKLAVAS----------------GNPEREIEENIGYEMLMGIC-----RDRVDGE---NLYRIWRRTVI--ENPVVAGYATI--------IANPGLADVERVDE--------IVEAFYQRIPDAVA-------VNGQLPI-CTISGTILRR------VGEDSGVTRFHTECRDPEAIRRAN-----------AGVHDT----------------------------RRYRPGMMQLRRAFRTYWCLPGRAELELERRFREK-------GWQTTLWPNLDRVDLVATSASGRH-----LAIDVKDYLSPNTLAARFSGFKE-----FTADHECYLVIPDYIPE------VDARFEAKFEAFRA-SLGKTTVALQTVSGLIDELEAA---------     372487592       DMVNLFAAGLR-RLQEDIDHQG------HIQAPYPEPLSRAFNLAAA--FNIERRTSYP--------VDLASLIR-AAARPIFEWCS-AYGECEHGEAF----FASRLID----DGEITPDCISMA--------------------GLTETDAEQAFYQMLMSAC------AELDEDLGQRFYSEWRRAVI--ENPVAESHAIFIAKY-----DVLRSNTILTKK---------LVDFFYERIPEVHA-------VDGKLAL-CPLTGTRLKK-------------HANQWATEMRDPVAAQHL----------RDYGPRW---------------------------IEHTSATIELKRPVRVFWTLPGWHELQLHREVQAR-------GCTSILWPDFDSVDLLVSQPSGRR-----FAVDVKDHISPISLSRTSGQHNR-----RRRYKRVI-VIPDYLED------RLPQYKTLFCKAAL-SALKEPEQIMTASEFLELLGDKA--------     17229467        MEISEFNSLVI-GLAEFVRRDG--------KYPYPELLRHGLNK------LALELTAVP------YPRTLNGLLV-LLEKPVKTWYP---RRFIPKEFD----SDFGLLY----EGGLSEEANRYFYEELLERTKLSESATATTQQIAIENLQFQRILARLQELY-----NNDSNPESVQREYVLLRRFLI--ENRYTTKAHIRKVF------LKTKYIHIQ------------EVGELYDECEIEEP----------SWN--CDRCGPLFKK-------------YGKLRGIKPSVCNDH-------------RQSLPYVKK-------------------------ITWQHGLCRLKFGIHLRICLPGIPEIRLFEILAELQSKFPQQLCAIHLYPGIDRYDLQLCFCDQTT-----WAVDIKDYQSPYKLAPKLTPLFSEGD--LRYHESFY-VIPIERLQ------QTQNYLEILREQAA--KLPSSIHLLSDAVFEKRVINKIESLLEGNS     220930101       KRLNFYAYSLT-EIFESDSNKK-----------LLDNWKKASLLLQA--ITIETKSEVI------FPLTINEQID-IITKPICDWGIAHFDKFDGILEG-------LIWNNS-GEWHLTDVARSLAGDY----------------NPGEGAQFYRELQIHVR--------SNEIADS----YYTTLRELAV--EKPLLDEKEYLVEEEKLEE-KASSRDKLIFKLFLNAEQPSHLLKKIYEEIPPDVI-------YDKRIYI-CPYCGYTLSK----KIKCNTMGKEYEEFYCISKKCFERKG-----------KSEVKCIH--------------------------INGQMKYYRPRAEIMHSVVIPGRSEILLRDKLTELIRDK---SFTVELYPNADETDIKIEFSDGEI-----WIADVKDWDIPFKLASHLNIRGFAKDKGISYNKAFL-VLPDEVQN---------PYINVLRNNWI---DAKLYQIIRVKEFIELVKERADDSEAGV-     403070936       DSLSILARSII-DMDKAFKDEN--------LEGFPKLMIKATGDLHS--IGLEL-GLDF---KDIYPLTLDEQIV-WWEKPLHLWPY---PSFDEWFFVEEDELEQAVLY----DYDVTEFCISIAQGG--------------------HDPIHEQDTKEFISIR-----NLLNNDS---HDYSNIRKFLI--ENPILNLDSPTYIR------KIAHMPPNIQEK---------LVNYLYEAVPKHYY-------NEGQVYV-CPRCGWTLSI------------TDDGDANCDTNKCKK--------------LSSGWYFKSN------------------------KLNSHKLLRVRRGIRRYIVDPGLLELNLFKRIEKLPR-----LKDVILYPNNDEYDLELIFESGTS-----WVVDVKDWVNPYMLNHNLKSFSTN----VIREKAFI-VVPSFRGS---------TYVKTLRSICK-----KGYEVLDDKQFVKLVKKEGAQFESQI-     392549085       MMAQEALAMSA-KAATLWQKLN-----DKDP-LVSAHLFMGHISHTI--AYINSISNKP-------ITSVEEFIK-NLRQPICEWLP------VPDAFT--------LIN----RFGVTEFAEDIVLES---------------SGNLIDELNQKQIKDAKDRLA-----SMPGGDL----HYTRFRKGLI--ENPVAQPKNIH---------SFLNITGCN-------------FRDLYEPLPSNIL-----LEGTSDYFP-CPRCKWPMQI------------KKGEIVRCASNQCVNEGAHYSLSKGKL--SPLGGLPAPA------------------------SEDGTSSYRLVRGIWRYTVLSGLSELDLANKLEQLP------DVTVTMWPFLDDFDLEVTYDSGDSMAKHTWRVDVKDWSLESRLAANIQGH-------SHYQELCY-VIPDYRRQ------QLKYLEEQFKSSPM-------ITFHTSSKFIQMVKKYIKRLSNEPF     290956755       MTLELLAAGLAGIQANQQKALWSSAQPSVDPTGFPPAWKAGVTRLLW--RALED-GCRE-------VTSHADVFR-WCRVPLGQWPL---ALCVAQ-------SDSELLLIE--NGRPSAFAEQ------------------AARLLLSKDPEAELVENRCYELMIAVADRNGETEQEVQNNYVQLRGFLT--KHAVASDIDLLGLLRAFPAKDTNGQPWVKH-----------WFSQCYRAQPASGA---------VVLSF-CDGCENPVSG----------------QRGCGTPGCTG--------------GPVART----------------------------VDIMAEYFVQNRAVRRFFHDPGLAEQRMFAALEPEF------RQRLRPWWGMDAVDVAIDFGTGKMGVGEWWAADVKDHASATLLGRSFRWNP------RAASQRRILVIAQHRFE-------QPDYVDDLTTSLQ--GRVQGIEIMSENAFVAAALAHDGRRR----     172039223       ETVQRIAKGIC-QYHQNINRQK--------LSQYPANLQLAVDKLII--KCLLT-KTEP-------LQGVPDFLNRWAKQLLQDWDL--DINCPEDWHS------KSLIEEQ----KPSNFCVETAEEY-----------------LEEYGNFQRKVVEKLRL-------KAFCDCD----LYTKFRQYII--EHPIITKGELDTT-------AILEFSSLKE-----------LLIDCYELAPESYK-------KDGNFYY-CGHCGGLMYL------------KSDGDLRCENRHCLQYKK-----------KPIPFK----------------------------ADSNDLVLWLKQDLRYFMHRPGRPEVRLFNKLKKLD------LKEVILFPNLDIYDIHLVFPDDTV-----WAIDLKFWESAYNLAKKVDKPIPRWKE-QPYNECFF-IFPDEIKH------YSKEYIQEFRSYCT--VPLKKSQVMFEGAFMQKVKRKLGKQS----     126660232       ETIQKIAKGIC-QYHQNISRQK--------LSQYPANLQLAVDKLII--NCLLT-GIEP-------LQGVPDFLNRLAKQPLQDWDL--DINCPEDWHS------KSLVEEQ----KPSNFCVETAEEY-----------------LEEYGNFQRKVVEKLRLKS-----LTSKDRN----LYTKFRQYII--EHPVVTKGELDTN-------AILEFSSLKE-----------LLIDCYEPAPESYK-------KDDNFYC-CGHCDSLMYL------------KSDGDLRCENRHCLQYKK-----------KPIPFK----------------------------ADSNDLVLWLKQDLRYFMHRPGKPEVRLFNKLKKLD------LKEVILFPDLDKYDIHLVFPDDTV-----WAVDLKFWESAYNLAKKVDKPIPRWKE-KPYNECFF-IFPDEIKH------YGKEYIQEFRSYCT--VPLKKSQVMFEGAFMQKVKRKWSKQS----     126656941       ILFIKLCKGLV-EICDRINRGEPAYLVNDYQ-PFPQPLHEAFEKLSI--KWILRDGKIR-------HPSILNMVQ-AARESIEAVDP----EFCQWVDF----PDEPLIEDM---TKPSEECEDYAIEY---------------SLSLEIDNNQSYIPRLMEEIQ-----KNDLPYS----TYTEFRRFIA--ENPFPSELDKALLID-----EHPEIERVKD-----------LLEEAYQDVPLQSN----------DKQL-CKKCGGYLDC----------------AAREIEGCC----------------ESLEDKVDK-------------------------APLPDTV-------RCLI-RPSLIELRLEKKIKEM-------GLEVELWPELDKADLKVTFPDGKS-----WAVDAKDWVNATKLARELNEDGIPE---IGQCQSFF-VVPDYRLK-------KLQNQAILKSKYQ-----GNIPVISESELIKRIKKELK-------     359458563       ILFIKLCKGLV-TVCDRINHGQPAYIVDAAQ-PFPQPLYEAFQSLSL--KWLLQ-GDEP-KGRKMKHPSILCLIE-AARKSTEAIDP----DFAELVDF----PGEPLIEEL---SRPSEECEAWASEY---------------ALNLERDQNQSYILDLMNEIR-----RLSLPDS----VYVSIRRFIA--EHPFPSEFEIARFRS-----DNPEIESVQD-----------FLMQAYREAPPQSS----------TLPL-CKTCGGYLDC-----------------AAAELGYC----------------KPLTEEVSR-------------------------APIEDTV-------ICLI-RPSLLELRLAKTLQDM-------GLTVELWPQLDQADLKVTFPNGEI-----WAVDAKDWGSATKLVHTLGQDEIPD---IGQSTSFF-IVPDYRWN-------NLAYQATIEAQYV-----GEPPILSESELVKQAKRALL-------     307153447       ILFIKLCKGLV-QICDRINRGEPAYLVDASQ-PFPKPLYEAFQELSL--KWILRDGKMR-------HPSILCMIE-AARDKVEAVEP----EFIECVDF----PDEPLIENM---TRPSEECEAWASKY---------------AINLERDQNQSYIPRLIQEIE-----RLSLPYS----TYPLFRKFIA--ENQFPSDFDLTRFGS-----DNPNIRPVQE-----------LVRQAYREAPPQSV----------SMPL-CKTCGGYLDC----------------AARDIEDCC----------------EPLDRRVDR-------------------------VPTEDTV-------ICLI-RPSFLELRLARILEEM-------GLQVELWPDLDKADLRVKFPTGEV-----WALDAKDWGSATLLAIELNQDTIPD---LGQSQSFF-VVPDYRWQ-------KLAYQAAFESRYK-----NDISVLSESELIKRARGYLNER-----     309791271       LIVPLLAAGLT-RLSVRVEAGE----ALRAP--YDEAWERGARMLTL--ECLTR-GVAA-------PAHLGEVINLWCRIAPVDWPL----PLGPAGRL----YAEPLLDEG--DEL-TSLCRELAQNL-------------VSGDAE-L-ELSESLMAGVRSQA-----QARRNQD----GYVSFRRYLI--EHPVSTQDDLSDAG------ARREISPFAA-----------TLHEMYERIPSTAI-------HNGQLLL-CGHCGWTLER-------------ADNRLRCGGPRCRTLTDN----------FSRGTSTRP-------------------------YDSADPPLRARRAIRQYVIAPGVYEINLARRIEAM-------GLSCQLWPFYDRYDLRVVFENGEV-----WAIDVKDWKHPHLLGRRLTTFTDVA---PDWQRAFF-AIPDSRVR------ENRSYLATLRNLVR----SDDFAIVTISDLLHAIGRRKEAGHA---     302870452       TILPQLARGLI-DL-GDAARRG----SLDLP--YPPSIQRVLDRIVL--ACLRE-GRTP-------PSSVPELIAWCTAPLGARWPS----SALPGFLS----AEVTLLDLR--RRLPTRSCAELAAYG-------------SG-----N-SAEQSVVRKLDEFA-----QAASTPA----LFTQCRDFLI--DRVAVTNDDTRG--------TSWKPQIWK------------LVQDLYRP-PVSAY-------AHGGLTT-CPTCGLLATV------------EHGRIAWCEGEICER--------------GPTPGP----------------------------DYKKGKVRVLDFPLRLFLSLPGRTERAVMRRLAER-------GVTGTLLPG-ELGAYQLSEPVLGR-----SNVQVFDRVEPLLLAAQLS----------GRSDLLA-VIPDHLAR------ARPALLKSVRDALP---PELDVVVTTQTAASEGAPQHA--------     357393637       --------------------------------------------MVL--RCLDL-GEEP-------PLSVPGLLEWCRRRPAGDRVF----GVPPGLLD----AGARLVHPV--GVTPTRTCLELASHE----------------RRGGIEQEARALLADLARRC-----RNTE-------QYRRSRRFLV--RHVAVHQKDRFE--------RGWDKEVWV------------RVRELYQPVPEFLV-------VAGKFLR-CGTCRLPALL-GGRRAPEHGAPVAGPQTWCEGERC----------------PAGERMEL--------------------------VRDPERVLLLRRALRMFLALPSAVEHAGLETLAAH-------GLVHEAVPE-ELGSYR--LPGLGA-----YTVHFYDRVQPVLLADRFT----------DLVDRLP-GTPVLVLP--RRSAGSTEFRRALAAALP-DELRARTLISAPQELARRIHQHHTGRRKGAH     375092906       MVISLAASGLV-ELSQSLERQG----AFPVP--YPAALQRALDRLAA--MGVIA-GAEV-------PRSVMDLAAWAH-RPFVQWPF----RPAVEGLN----DDESLLTD----GKPTKACLEWAVV--------------SGDVEG-E-IRERLLIHGVLDVC-----KANDDPD----AYVRFRRLLV--EKPAMSERDLLVT------MAQPGFAILAQ-----------HLRGAYRAAPAEAL-------ATGEAVV-CGGCGHLWTL------------QADGVRRCSEWDC----------------PAPTTIKKR-------------------------LPASEGVVWLSRELRMFVSGPGRAELRIAKTLERA-------GVTAQLWPDFDACDV---LPVEVP-----WAADVKSWTNPVRLAWRLTERPFVLP--RGAERGFI-VIAKEQTT------GRPEYLRALRNHCTWLKGQSRIEAVSEKTFIDRVVREFRKAQA---     392942505       GLLGAVASALV-AI-DELQNLT----MFQMP--YPSSVQRALDKIVL--HCLYQ-GALP-------PGSVAALVRWGYERPLGTWPL----MLDTGIYP----VDGFLIDEE--SGSPTELCHEIALCA-------------ETDNPLWE-ASHRMATLAALATE-----RGQDS------TFGTMRSVLA--KHPMLT-NELFNAVR-----FANNLGALEK-----------RLGEFYQKIGPEYE-------VDGAVFP-CAHCGTPLLP------------TDAGSWWCEREECSAGGPV----------SPDDPV----------------------------DWDAKVSIQTDRRHRQFVSGPGRAVLRIASSLALP-------RVTVRLWPAQSPGDLRVKVADVLK-----WTAAIVDWHNPALLGRAIAATTAW----SGTDTAIW-VVAQYRVD------ADPAYLQLVRKYGT--TAAGSPRVCSEEEFTSMVRRHSEESGRA--     357392955       AALQDVATALV-ALHDVENPGT-------FQLPYPAEAQRALDRLAL--TCLWL-RCPL-------PESVPGLIEWGATRILDDWPL----HLPEGTAH----PDGRLLDED--TRLPTQLCLEWAADA----------------ERHPANRFDRQVLAFAADRC-----RAEDDQE----TYLAFRRQLA--KEPVTTKNKLSTMLG----RTDMDADLVRD-----------VLNEVYRPAPPGSV-------EKGRAVL-CGRCGILLVR------------TRRSEWRCEREECRRRGV-----------REGKEL----------------------------DARDGGLRQLTRPVRQWITAPGRVALKLEQRLRAW--------CEVELWPDFGAFDLRLVLPGGVV-----WAVDVHDWVDPGLLGRLREQPRPD----LAAHRRLW-VVPRESVT------AVSGYRTRYREVRP-----IGPELLSDAELVSEVKAFVENGPRGRE     291439984       ALLRQIATALA-CLSRLRTVPNP---------VYPAKVQNAYNRLVL--HCLRR-GEEP-------PGSVPEMVRWAGERPLTEWPL----KLPAEEFP----GASPLVDPE--TRLPHQLCLEWEVSA----------------ADPAAELFENELIREAVARC-----RAAKSPQ----AYTAFRALLT--SRPVLTGAELALLG------GDPDCGMLLHD----------VIKRCYEPVPASYQ-------RHGTYRQ-CARCRCLMVP------------LLHGGHRCELDRCRRDGT-----------SDEGPRL---------------------------RAGKGGLLQLSRPLRMFITSPGLAETDLQAALLKKF------GITAEMWPHYDVYDLRIPLPGGRH-----WAVDVKDRVNPVLLARSLTPFRNE----PPYDRAFL-VVPRYRFR------DNEAYGRQFRRNLP-EDLADRITLLDDRAFLRLVADEIPSPRDKAG     302867039       TLLRMVATALV-DLSRHDRSPDP---------IYSDKVQKAYNHLVL--QCLLR-EMEP-------PASVAGMARWAATRPIGQWPF----DLPAEAEN----LDEYLVDAQ--TGVPTQRCYEWEVSG----------------PDAAAELFENEIIRTVFAQC-----ETARSPQ----SYTAFRRLLI--DKPVLTGIDKATLIG-----EHPELGTVLE-----------TVNRCYEPAPAAYR------GTDGRFAT-CARCHCLLKP-------------DGRSWRCELDRCRREA------------HATPKEFID-------------------------DRSTGGLYLLRAPLRMFITGPGLAEVELEQKLIGK-------GLVPEMWPGCDAYDLRITLPNRQV-----WAIDVKDRANPALLGHSATRFRPE----PPYNRAFL-VVPQYRFD------DREDYKIVFEHFCP-PEVKEQVTLLSDKAFLRMLTTTLTRLRKNGS     345850904       VLLRDVATAVL-HLAAVDRLDS-------FTLPYPAGAQRALDALVL--QCLRN-GAKP-------PAGVPEMMRWARARPLGSWPL---DRLPTDLFD----TADRLIDED--SGEPSQLCHELTVQG---------------HGDSTGRQYDRLVIHEALRAC-----RAMSSPE----SYTAFRRLLV--NQPVLTEADWAEVS------TDLFLDPVRF-----------LVEEIYAPVPLGFR-------RDGAYLC-CHRCLTLLHP------------VSDTEWWCERDQCRHQGP-----------PPHGRELV--------------------------AFEVGELRQLRKPLRQFVTGPGRAEVFLEGELRAL-------GLTVEMWPGFDAYDLRITFPDGHV-----WAVDVKDWAHPAFLGRAATAVRPE----PPYDEACW-VVPEFRVR------ARRDYLAMYAKERG--AGAGGLRLLTDDQLKRAARLRLSGERGPDA     406692973       VLLRDVATAVL-HLAAVDRLGS-------FTLPYPAGAQRALDALVL--QCLRN-GAKP-------PAGVPEMMRWARARPLGSWPL---DRLPTDLFD----TADRLIEED--SGEPSQLCHELAVQG---------------YGDSTGRQYDRLVIHEALRAC-----RAMSSPE----SYTAFRRLLV--DRPVLTEADWAEVS------TDLFLDPVRF-----------LVEEIYAPVPLGFR-------RDGAYLC-CHRCLTLLHP------------VSDTEWWCERDQCRHQGP-----------PPRGRELV--------------------------ASEVGELRQLRKPLRQFVTGPGRAEVFLEDELRAL-------GLTVEMWPGFDAYDLRITFPDGHV-----WAVDVKDWAHPAFLGRAATAVRPE----PPYDEACW-VVPAFRVR------ARPDYLAMYAKERG--AGAGGLRLLTDDQLKRTARLRLSGERGPDA     254391265       ALLRMLAGAVV-ALEDVTGLDS-------FRLPYPSDAQRALDHTAL--ACLRR-RAEP-------PRSIPELLEWCRNRPLTDWPV----DLPADAVA----PEDRFLDEE--SGLPTELTREWWVQP----------------GDTPARLHHRDVVRWALRTC-----RQHGPEP-----YAAFRRLLV--RQPVLTHTEWFSVI------TDPLLTDVRE-----------LLGSIYQEVPPGLV-------RPGRGCAECLHCGMLLTP------------VGEADWWCERDSCRSLGPE----------PEIGRIIGP-------------------------EQCNG-LVQLDRPLRQFVSRPGRTALDLEAGLTRL-------GATVRMWPLTDAYPMWVGFPDGHV-----WALDIKDWAHPGLLGRTAATVRAD----PPYDEAFW-VVPGYRVT------ARPDYLAVFHRHRP--PGAGGLTLLPEDEVLRRAELRLRAANGLPE     386383242       ELVELLARAVV-ALEDITELSS-------FRLPYPHVVHQALNRTAL--VCLLR-RAEP-------PRSVPELLSWCRDRPLANWLL----DLPADAAG----PADRLLDEH--TGLPTELVREWWAHQ----------------ADVPARLHHQDVLRWALQRC-----RSLGPES-----YGAFRRLLV--EHPVLTHTEWFKVI------SNPALMGLPE-----------LLENIYQPVPSCFV-------RPGRGCAQCRRCGTLLTP------------VGDADWWCERDYCRSESGE----------PPIDRIIPP-------------------------EQCKG-LVQLDRPLRQFVTWPGRIARGLRKRLTRL-------GATVTMWPTTEAYIMRVGLPDGRS-----WAVDIKDRAHPALLGRSAGPAPAG----PPHHETLW-VVPGHRVR------ARPDYLTVFHRNHP--RPEGGISLLPEGELLLRAERHIRESGGGPA     118476562       LQIREIKEMIY-FIAKGLEQWE-----EKGWTEIPETLHKGHLLFMK--NTLQ--SNMP-------PNNIFDLIK-LLHKPVEKWGMAGIEKLLPGDST-------FLVE----GFGLSIDIEDFLEEY------------------TSPEEFDQSVMKEILQYC-----RKYGLDE----EYRKIRSIVANPNNAVLTYVRLLSIL------SLIEDDKLKE-----------CLKKCYEEVPHSVD----------NYKK-CPNCGWTVSF-------------VKGSWRCNKEQICGHF------------QSFERLESYK------------------------FSEGESVYRVRPGIQRYVLLTGIIEEKICKRLK---------GYDAVLYPNVDEFDIRINVNDTL------IDLDVKDYRSPRMLANAFNNKNSGYL--EKYHKNAYVIIPNYRLK------MNPNYKELVCSSLS-KESSVYIQIMTERELFKCLKEETLCSAN---     219666711       DLAQKSLLNII-QGLRGWSRDW---------KRIPKEMYLGHLQFSQ--VALNE-GIIP-------PGNLYDLLA-MLQKPCQEWGI---PAFEASFDL----DASILKK----YGGLSYEAEEFLEEH------------------VSVEESEARVMLEILSHCREKVKEANNYQE----LYSKIREFISSPEHAVLPSGEVYDFA------HSLQDKKLSD-----------LFFSLYEPSPLPYE----------EYYY-CPYCGWTLTT-------------QRGKNRCTNRSCHLNG------------EFRNPVKMP-------------------------NASRDVLWRLTPGIQRYVLVPGIAELKLYKRLETK-------KYDVQLYPEIDRYDIRVKLENSI------VDIDVKEYYSIRSLADHINANW------QKYHENSYIVIPGHVLR------EKPYFIERVANYLS-PEAYRHINIIPESKVLSVLERGVDGQ-----     398928561       SLLWLLANIAM---------------------TGSRDRAQTLDLLCQ--ACAGIHRLEP-----TLPVLTADRLEASLQVPVEEWLP---DTIRGELTG------ALLVSS----VVPSRICREMHLEY----------------EAREQMDRTQALILRIRDFC-----KVLPDGQ---TLYRTFRRFLI--QSPTTQRRPA----------RLIFQPLDV------------SLDDMYRAPKRHEL-------LGTYLYL-CPCCGWAMNV-------------TQPEVACTSQWCEQAGSLFD--------RQHDQVVRRSNGAVLA------------------GVAAEDILVLQDTFWKFTLLPGLLELQLAEGLQNL-------GFTPVLWPDFDESDVRIEL-DGEY-----LDLDAKVWRYPDVLASHLSA--------LPRSKLRWIVIPDYQAT----------SLPFLREKTG-------LQIHTYSSCLKELKKRA--------     399519612       HLLQLLAQGAS---------------------LSSKNRALAFPLLQR--ACALLWRLEP----VGYPMSAIELER-KLSVPLEDWLS---SAIRADYPG------PLLYSN-----IATQTCNEMLLEL----------------DVRELWEQVQASVNRVKQAC-----RLRADGE---THYRNFRLFLI--EHGVIVPFQA----------QESFVPLNL------------SLSEFYEPIPPHLH-------HNGLLYL-CPECKWPMNA-------------QRHQVSCDSAWCQDKKSLFV--------RDGSRLINRVDNSILL------------------GHPVEDRLMLKPPLWKFTLQPGLLEVALASALVAK-------GLEVQLWPDVDRTDLRIRLGHAY------QDIDAKVWISSYELAKHIES--------IPSSKPRWIVIPDYQMQ----------NIPLLRQRCP-----AGVAVFTQSQCVREAQKHAAPF-----     386022138       -------------------------------------------------------------------------------------------------------------------------------------------------------------------------------------------MFLI--EHGVIVPFQA----------QESFVPLNL------------SLSEFYEPIPPHLH-------HNGLLYL-CPECKWPMNA-------------QRHQVSCDSAWCQDKKSLFV--------RDGSRLINRVDNSILL------------------GHPVEDRLMLKPPLWKFTLQPGLLEVALASALVAK-------GLEVQLWPDVDRTDLRIRLGHAY------QDIDAKVWISSYELAKHIES--------IPSSKPRWIVIPDYQMQ----------NIPLLRQRCP-----AGVAVFTQSQCVREAQKHAAPF-----     302559387       KLVGDLTTAGL-RAAQAWTVRR----------EQPEAWREVSRMHGVF-LSLLSPGCGP--------ATPRDLIK-FLHRPMREWVPLDWDSLPDEVGSFKILGADDLLTGDAVEYG-SDYSEALFEDHEAGI---------DWVPRWARQTFER-VERAIYTVL-----SSAGQEE-----YVATRQMLI--EVPAGTADRVS---------DELHARGA-------------LHTEAYEPLPPDRQ---FVVGSESWYVP-CPTCKWPMHV-------------QGASLACRYPPHFGRFQVTDERDGSGVPRVRGPVVAS-------------------------ALSAADVVCVHEAVWRYITVPGVTEVALMRWLADQAGIG---GDAVTKWPHKDRWDITVRAGATI------FEVDLKDTRSPSKITAR-----------PPRARHV--VVPDYRAW----------QVAQLRRSLP-----AGYEVRTVRAFKTAIRSALKEAR----     295837640       ---MEIVKAFA-VCEHAVAAGE---------LTGPRRLDRLMEAHGI--LMAAC-GPEH------HIAFDELMGR-LTGERAGS-----LERLLPEWINAEELAGVRLLESD---GVATEDGFDFRHEAQRVMRAAEKVGKFSGQVTKPK-LDDEYSQESIFSAI-----KGP--------FYERHRRTLV--ENPTVPRKDL----------ATLSLPS--------------RANDFYRPIAQYAQ-------YSGWWWP-CPACQWPMKV-----TTDRSTGRIRGQVRCLYPWHDETGASYEFVVTTR--RKTAPELHPTFECRVPSGRFAPLWTGAVPLMPE-ARAVEETVVLVRPVWRYTVVPGLPELALHRAVSASLEGT---PWTSHLWPNGDQCDLWMTHADNDEPQ---FMADFKDYTWSNHLVSKLDLDG------GDRGGAEYLVVPDHRRE----------QVDQLNAVCR---RHGMKAAMTATEYLEMVINHAKGGQA---     386357489       -----MAAAGP---------------------GHRVRFTDLLERLTG-----------------------------AEAGSMTG--------LMPEWADPGSLDGLRFLDVD---GVAPGDAFDFRLEAERVIRAAKKVGRFAGRVTFTA-LDDEHSQESVFSAI-----KGP--------YYERHRTNLI--EHPVVLEKETATAVSDRPALADLELPT--------------RAIDFYRPVPQHAR-------YRGWWFP-CPACRWPMKV-----SLARADGREQGRAWCLYPWHEQTGATYLFQPS----EAEPPVLHPVFECHVPQGRQGELWTGCVPEVPQAQPVEG-HKALVRSVWRSTCVPGLPELRLHRVLNHHLKGT---GWRAELWVDGDRIDLHVANDNGPRRKTA-FPADFKDYTFVNHLVDKLDMDA------GDKGDAKWLVVPDHRDE----------QVQTLDPVCR----RYGMRAITASGFVDEVLASVREGRA---     297204966       AGLSDAAVRHM-VMAAAAMAAKALTDTEQSAEGRLGTLMDAYGRVQA------ARGPAA-------PLSFGRFRA-LLRGDLAR--------LLPAAVPAEEMDGVRLIDPD---GDFDEDFFDLEIEQRVLMRALAKTTH-GGRPASTRTLEAEMDQDRVFTAL-----RKRMDQD----AYVHGRSSLI--RMPAGSDAQL----------RRLNLPS--------------SVAEFYRPVCFDAM-------WDRWWFA-CPVCHWPMKV----TVHGSRAGTRTGGVRCFHRPHATWGAAYSFKLPD---AGRPPVLRPQSRPAAPSGAQAVLFPDLTGQVPE-PVPVEGHKALTRGVWRWTTVPGLVEIALFDALRER-------GLSVALWPELDAYDLLVTAGQGASRAE--FRLDVKDYTSALLLAKKIQADG------GDRGGAEWLVVPDYRES----------SLELVGSVAG----EFALKAATASGIGELICKKAGAAWQ---     329936080       MVMAAAAMAAK-ALTDTEQSAE----------GRLGTLMDAHGRVQA------ARGPAA-------PLSFGRFRA-LLRGDLAR--------LLPAAVPAEEMDGVRLIDPD---GDFDEDFFDLEIEQRVLMRALAKTTH-GGRPASTRTLEAEMDQDRVFTAL-----RKRMDQD----AYVHGRSSLI--RMPAGSDAQL----------RRLNLPS--------------SVAEFYRPVCFDAT-------WDRWWFA-CPVCRWPMKV----TVHGGRAGTRTGSVRCFHRPHATWGAAYSFKLPD---AGRPPVLRPQSRPAAPSGAQAVLFPDLTGQVPEPVPVEG-HKALTRGVWRWTTVPGLVEIALFDALRER-------GLSVALWPELDAYDLLVTAGQSAGRAE--FRIDVKDYTSALLLAKKIQADG------GDRGGAEWLVVPDYRES----------SLELVGSVAG----EFGLKAATASGIGELICKKAGVAWQ---     254390290       ERWRRVVAAAL-RAAYAWSVRR----------RYPTALREVAMMTGV--VMEAHGPCRG-------PASPSALVD-RLRAPLGELLA-----FAELGETEDSVVADAVLLDS--RDQLTPDVHDLVCEYALPLAGTFEAE--VWLPTWTR-MNADHIRHQAFASL-----IETRSQG----DYVVSRKFLI--DHPAGSREELA---------ELVSTTGAR------------VVSRGYTEIPAERR-YHAGPDTA-WWWP-CPVCAWPMEV-------------TGATVRCRYRPHASVFRIVPGRAH----RSRPGLIALDEGPRVARPE---------------ARPVNDAVCLDAGVWRFVVVPGASELRLSRALEKQ-------GAQVRMWPELDSYDLHVSAGPHE------FRIDVKEYRSVHRLIADLRTK-------PPQARVLL---PQTHEH----------QWDAVRAALP------FLAVTTETRFRAEVRRALRKGRTA--     407881733       ----RVVAAAL-RAAYAWSVRD----------VQRNSAMLEIARMTG--VVMEAHGPGK------GPVTPAELVD-RLRLPLRG--------LLAGVREQRAELDDVVLLDD--EGRLTENAYDLVCEHVVPLKDAAGA---QSWLPTWTTMHSDQIRQRVFTAM-----ITSTKQD----EYVASRRFLI--EHPAGGEQELVELR------DRVGGVR--------------LAKGGYRAIPEDQV--YRGLDAQGWWWP-CLECKWPMAV------------TSTGKVRCRYRPHSAVFDLVPGTRPTLRRRDSARRVSPPA-----------------------GKAVEGAKCVDAGVWRFVVVPGASELRIAAAAERA-------GAKVLLWPFRDRFDLLVEAGDEK------FSVDVKECLSLSSLIERLRTR-------PPSARVLL---PKSCEW----------QLETLRDALP------GLAVTTETKFLGQVRTAVRKAR----     consensus/100%  .............................................................................................................................................................................................h.................................................b.......................C..s.....................................................................................................su.........h................h.......h................h.h........b.........................s...................h....................h...h............     consensus/95%   ...........................................................................................................hh.........p...............................................................a...R..l...p.........................................h...a.......................C..C...h.....................s.....................................................................b.b...Pu.....l...h...............haP..-..sh.h..............hchb.......L.....................h...s...................h..............h.....h...h............     consensus/90%   .....................................h..................................b...................h..............lh.........s..h.ph.............................p...........................Y...R..lh..ppsh......................................h..hYp......................C..C...h.....................s....p...............................................................hb.h...Pu..E..l...h...............haP..-..Dl.l...s........h.hDhK....s..L...h..............p..h.hhs...................h..............h.....h...h............     consensus/85%   .....h...h...........................h..s..........h................s...h........h..........h..............lhp........s..h.ph.............................p.....h.....................Y...R..lh..cpsh....p.................................h..hYp.hs...............h...C..C...h.....................C....p...........................................................h...lb.a...PGb.E..l.p.l............ps.haP..D..Dl.l...s........h.lDhKs..ps..Ls.ph...........s..p..h.llP....p...........b..h..............h.s...h...h............     consensus/80%   ..h..hh..hh..........................h..s..........h................s...hh......sh.ph.......h..............llp........o..h.ch.............................p.....hb........p...........Y...Rphlh..cpsh.s..ph................................l.phYp.hs...............h...C..C...h.....................C....s...........................................................lp.slb.a...PGb.El.l.p.l..b.......shps.haP..D.hDl.l..sss.......h.lDhKsb.ps..Ls.php..........s..p..h.llPp...p..........bb..hpp............h.s.p.h.p.h...h........     consensus/75%   ..h.bhh.slh......................hs..hbbuh..h......h...s..........s.s...hhp.h.p.sh.pa.......h..............Llp........op.hb-h.............................p..bp.hbp.h.....p.....p.....Ys..Rphll..cpsh.s..ph................................lpphYp.hP...............h...Cs.C...hp.................p..C....C.................s.s....................................hb.lp.slbbah..PGb.El.l.p.lp.b.......uhpsphaP..D.hDl.l.hsss.......hslDhK-a.ps.bLupphp..........s.pcshh.VlPp.b.p..........bb..hpp.h........hpl.s.p.h.pbhp..h........     consensus/70%   ..h.bhh.ulh..h....p..p...........hs..hbbuhpbh.h....lbp.s..........s.shsphhp.h.pbsl.pW.......h...h.s........Llp........op.hb-hh.p.......................b.pp..bpbhbp.h.....p.....p.....Ys.hRphLl..cpPh.s..ch...........p.p.................blpphYc.hP...............h...Cs.C...hp.................p.bCp...C.................s.s....................................hh.Lp.slbbah..PGb.El.l.p.Lp.b.......GhpspLWP..D.hDlblphsss.......hslDhKDa.ps.bLupplp..........sbpcshh.VlPcbb.p..........hb..hpp.h........hplho.sph.pblppph........     ```      **- 3) Z+DinG-type\_helicase**       ```     Fe-binding cysteine rich and helical region are denoted above alignment                              Z domain----------------------------->                                                                                                                                                                                                        | DinG-type helicase ----------->                                       Walker A motif                                                                                                                   cysteine-rich region---------------------------------------------------------------------------------------------|                                                      Walker B motif                                                               large helical insert------------------------------------------------------------------------->     FINAL           ---------HHHHHHHHHHHHHHHH--------------------HHHHHHHHH----HHHHH-----HHHHHHHHHHHHHHHHHHHHHHHHHHHHHHHHH-----------------------------------------------HHHHHHHHHHH------------------------EEEEEE------------EEEEEE-----------------------------------------HH-HHHHHHHHHHHHH-----------------HHHHHHHHHHHHHHHHH----HHHHHHHHHH-HHH----EEEEE-----HHH-HHHHHHHHHHH---------------EEEEEE--HHHHHHHHHH----HHHHH--HHH----------EEEEEE-----HHHHHHHHHHHHH---H-----HHH-HHHH-HHHH---------------------------------------------------------------------------------------------------------------HHHHHHHHH----EEEE-HHHHH-------------------------HHHHHHH----EEEEHHHHHHHHHHHHHHHHHH----HHH------HHHHHHHHHHHHHHHHH-------------HHHHHHHHHHHHHHHHHHHHHHHHHH------------------HH--HHH---------HHHHHHHHHHHH---HHHHHHH---H-HHH--------------------------------HHHHHHHHHH---HHHH---------------------HHHHHHHHHHHHHHHHHHHHHHHHHHHHHHHH-HHHHHH---------------------------------------HHHH--------------HHHHHHHHHHHHHHHHHHHHHHHHHHHHHH-----HHHHHHH----HHHHHHHH-------HHHH-------------EE----------E-HHHHHEEEEEEEE---HHHHHHHHHHHHHH---------EEEEE------------------------------------EEE-----EEE---------------EEEEEEEE-----------------EEEEE-----HHHHHHHHHHHHHHH---------HHHHHHHHHHH---------------------EEEEEE--HHHHHHHHHHHHH------------EEEEEEE--------------------------------HHHHH----------EEEEE-HHHH---EEEE-----------------------EEEEEEEEEE--------HHHHHHHHHHHHHHH------------------------HHHHHHHHHHHHHHHHHHHHH---HHHHH-HH---HH--H----HHHHHHHHHH-------HHHHHHHH-----------EEEEEE-------HH------------------------------------HHHHHHHHHH-------------------HHHHHHHHHHHHHHHHHHH--     ALIGN           ---------HHHHHHHHHHHHHHHH--------------------HHHHHHHHH---------------HHHHHHHHHHHHHH-H---HHHHHHHHHHHHH-----------------------------------------------HHHHHHHHHHH------------------------EEEEEE-------------EEEEE----------------------------------------HHH-HHHHHHHHH-HHH---------------H---HHHHHHHHHHHH------------HHHHH-HHH-----EEEEE-----HH-HHHHHHHHHHH---------------EEEEEE--HHHHHHHHHH----HHHHH----------------EEEE------HHHHHHHHHHHHH---HH--------------------HHHHHH----------------------------------------------------------------------------------------------------------HHHHHH--EEEEE-----------------------------HHHHHHHHHH---EEEEE-HHHHHHHHH-----HHHH------------HHHHHHHHHHH------------------HHHHHHHHHHHHHHHHHHHHHHHHHH------------------HH--HHH------HHHHHHHHHHHHHHH----------------HH--------------------------------HHHHHHHHH-------H---------------------HHHHHHHHHHH-------HHHHHHHHHHHHHH-HHHHHH-----------------------------------------------------------HHHHHHHHHHHHHHHHHHHHHHHHHHHH-----HHHHHHH--------------------------------EEEEEE-----------------EEEEEEE----HEEEHH-----------------EEEEEE------------------------------------EE-----HEEH--------------EEHEE--------------------EEEE-----HHHHHHHHHHHHHHH------------HHHHHHHH----------------------EEEEEE--HHHHHHHHHHHHH------------EEEEEE------------------------------------------------EEEEEHHHHH----EEE-----------------------EEEEEEEEEE---------HHHHHHHHHHHHHHH------------------------HHHHHHHHHHHHHHHHHHHH---H--------------H----HHHHHHHHHH-------HHHHHHHH-----------EEEEEEH------HH------------------------------------HHHHHHHHHH-------------------HHHHHHHHH-HHHHHHHH---     HMM             ---------HHHHHHHHHHHHHHHHH-------------------HHHHHHHHH--HHHHHHHH----HHHHHHHHHHHHHHHHH---HHHHHHHHHHHHH-----------------EE---------------------------HHHHHHHHHHHHH------------EE---------EEEEEE------------EEEEEEE-------------------------------------EE-HH-HHHHHHHHHHHH------------------HHHHHHHHHHHHEHE------------HHHH-HHHHHH-HHEEEE---HHHH-HHHHHHHHHHH---------------EEEEEE--HHHHHHHHHH----HHHHH--HHH----------EEEEEE-----HHHHHHHHHHHHH---HH-----------H-HHHHHHHH--H-----------------------------------------------------------------------------------------------EEEEEE-----HHHHHHHHH--HEEHHHHHHH-------EE---------------HHHHHHHHHHHHHHEHHHHHHHHHHHHHH----EEE----H------HHHHHHHHHH------H-------------HHHHHHHHHHHHHHHHHHHHHHHHHH------------------HH--HH----------HHHHHHHHHHHH-----------------H--------------------------------HHHHHHHHHH----HHH---------------------HHHHHHHHHH-------EEEE--HHHHHHHHH-HHHHHH------------------------------------------------------------HHHHHHHHHHHHHHHHHHHHHHHHHHH-----HHHHHHH--H----HHHHHH---------E--------EEEEEEE----------E------EEEEEEEE--HHHHHHHHHHHHHHH---------EEEEEE----------------------------------HHHE---HHHEEH---------E---EEEEEEEEEE----------------EEEEE----HHHHHHHHHHHHHHHH---------HHHHHHHHHHH------------------HHEEEEEEE--HHHHHHHHHHHHH--------HH--EEEEEE---------------------------------HHHHHHH--------EEEEEEHHHH---EEEE-------------------E---E-E-EEEEEE--------HHHHHHHHHHHHHHH-------------------------HHHHHHHHHHHHHHHHHHHH---H--HH-HH---HH--H----HHHHHHHHHH-------HHHHHHHHHHH--------EEEEEEHHHHH--HH------------------------------------HHHHHHHHH--------------------HHHHHHHHHHHHHHHHHHH--     FREQ            ---------HHHHHHHHHHHHHHHH--------------------HHHHHHHH-----HHHHHH----HHHHHHHHHHHHHHHHHHHHHHHHHHHHHHHHH------------------------------------------------HHHHHHHHH------HH------------------EEEE---------------EEE------------------------------------------HH-HHHHHHHHH-HHH---------------HHHHHHHHH-------HHH----HHHHHHHHHH-HH-----EEEEE-----HHH-HHHHHHHHHHH---------------EEEEE---HHHHHHHH-------HHHH--HHHH----------EEE-------HHHHHHHHHHHHH---H----HHHH-HHHH-H-------EEEEEE----------------------------------------------------------------------------------------------------HHHHHHHHHH---EEEEEE-EEEHH-HHHE---------------------HHE-----HHHHHHHHH----HHHHHHHH-----HHHH------HHHHHHHHHHHHHHHHH-------------HHHHHHHHHHHHHHHHHHHHHHHHHH------------------HH--HH----------HHHHHHHHHHHH---HHHHHHHH-HH-HHH--------------------------------HHHHHHHHHHHHHHHHH---------------------HHHHHHHHHHHHHHHHHHHHHHHHHHHHH-------EEEE------------------------------EEE---HHHHHH--------------HHHHHHH----EEEEEHHHHH---HHHHHH-----HHHHHHH--HHHHHHHH------HHHHHHH-------------HH----------HHHHHHHHHHHHHHH----EEE-HHHHHHHHHHHH-------EEEE-----------------------------------EEEE------------------------EEEE--------------------EEEE--------HHHHHHHHHHHHH-----------HHHHHHHHH---------------------HHHEEE--HHHHHHHHHHHHH------------EEEEEEE------------------------------------------------HEE--HHHHH----EE-------------------H---H-E-EEEEEE---------HHHHHHHHHHHHHH-------------------------HHHHHHHHHHHHHHHHHHHH------HH-H--------H----HHHHHHHHHH-------HHHHHH-H-----------EEEEEE--------H------------------------------------HHHHHHHHH--------------------HHHHHHHHHHHHHHHHHHH--     PSSM            ------------HHHHHHHHHHHHH--------------------HHHHHHHH-------HH------HHHHHHHHHHHHHHHHHHH-HHHHHHHHHHHHH-----------------------------------------------HHHHHHHHHHH------------------------EEEEE-------------EEEEEE------------------------------------------H-HHHHHHHHHHHH-------------------HHHHHHHHHHHHHHHH----HHHHHHHHHH-HHH----EEEE------HHH-HHHHHHHHHHH---------------EEEEEE--HHHHHHHHHH----HHHHH----------------EEEEE------HHHHHHHHHHH---------------HHH-HHHHH----------------------------------------------------------------------------------------------------------------HHHHHHHH---EEEE--HH-------H------------------------------EEEEE--HHHHHHHHHH---HHE-----H------HHHHHHHHHHHHHH--H--------------HHHHHHHHHHHHHHHHHHHHHHHHH------------------HH--HHH-HH------HHHHHHHHHHHH-----------------H--------------------------------HHHHHHHHHH----HHH---------------------HHHHHHHHH-HH--HHHHHHHHHHHHHHHHHH-HHHHHHH---------------------------------------------------------HHHHHHHHHHHHHHHHHHHHHHHHHHHHH-----HHHHHHH-----HHHHHHH------------------------EE----------------EEEEEEEEE---HHHHH-------------------EEEE-----------------------------H-----HH-------EEE---------------EEEEE--------------------EEEEE------HHHHHHHHHHHHH------------HHHHHHHHH---------------------EEEEEE--HHHHHHHHHHHH-------------EEEEEEE--------------------------------H-H-H----------EEEEE--------EEEE---------------------------EEEEEE--------HHHHHHHHHHHHHHHH-----------------------HHHHHHHHHHHHHHHHHHHHH---H----------HH--H----HHHHHHHHHH-------HHHHHHH------------EEEEEE--------H------------------------------------HHHHHHHHHH-------------------HHHHHHHHHHHHHHHHHHH--     392549086       ---MNRFRKTALHHAAISILYLCNEYET--NP---V---------LGDALLLTLGRLNHLPYWGSIPTEIQNHILETIRLSQISYLSKSRALLLLLEVVEEEGNPVVLTTPGANE---LDIATL----------------AFSCMPSKSYSQVIEEGLLFLRALYAK--KPHTFKQSKQ----STITFEP---QT------GLQFVVP---TELV------------PDELSPIRVETQPELEEICISVP-ELIEHARRLSNLT---------------NEPNYFKNTVLFLESLKSG----KTNANSDTLW-FSTGEL-QLVIAPTGSGKSI-LTRVMSTFLLSK-----N-------ISIAIVVPVVKDAINEYKK----LSSFI--ELLGLNS------QAGLLFSHR--KLGTETCDELAEA---LSSDEASRD-QSEW-VFRHLAYFCQLSAYE-------------AEEKTFEYGDEP----CTQ---------------------------------------------------------SGFSCPFMAQCGKFNHQRNSAKSQLLFINHHSYLVGRLKVPMVDRH--------ENPINTIQQLIAHRAQLVLIDEVDLLQKNLIGLDVGSLSLTKNKG------PTVAGLLSRELEDSGKW-------------PNNFGIIEQLNTLELSAKLLREAINS------------------NH--ISW-PKQFGYLDTPALMTVSLEKM---IHFRFKELAPN-YDD--------------------------------NMLGLLFDNSPISDNPQ---------------------WENLRVALEYWNHTGAYVGMSETVRRKEVSQA-LDTLLVLYQPEEKP-----------------------LPAEIVDKRKKL--------------EASVKDRLIGMLVLHRMFNHLQERLRDITY-----QIGSTDL--RASDYASEFYSQLIGYKPFSISPLGPLGGRYYGFQL----------DGKEKRQQLSVISLSGDPHQTIAHLGNFASRSICGI---PRTIIGLSATAWFP--------------------GAT-----SCDVSGSLAFYIA---------DETKKLHLYLSEVF------------EQEKVIKVSGT--TKLRMSNLYRLAQGLW---------CQTLESKIQQL----KQSK---------PERARVMLVTGSHSEAEEVAKALTEKMGEE---TALKRIRFLVSNS--------YDSSKNPK-----------AIKRNELANFA---HSDADILISCFSSVARGHNIL---QP-----------GSAE---SAISAIYVLVRPVPHFIDPSLALAHVSYNSLKTE-------------------YAEPASHEILKREQKFAHISLRQYY---NEIGP-VKLLTPE--L----KMEYFCNVLV-------ELLQLAGRGRR----GGTDIAIYLVDGAFHDQEC------------------------------------GWAVLIKDTLDI------------WKESGHYEQMKILHGALINALERFII-------------------------------------     398928562       -------MHETAEISCVLFLALAAKYVS--AS---P--------VIADAAALAAGQVIHWAGL-KALTRKELTTLGVALRYIHPQLTEPREFLSAAQSLLLRD----GAL--------FELTTD---EPLIQLSYRHQYRLVSVVEIDDLIQEYLARLCA---FTQP--ERKRIPAKGA----FTLGAKV---EG-----NYKLFEILEEDRAAL------------PPQGQTMPIPNVPPLARASWNIQ-ALHTLAEQLDAA--------------SSVHNSHLKSLRNIICKEVDV----Q----------ADAGNF-YRVNAPTGSGKTV-LMVMMAVDAARR-----G-------EQVAIGVPGLLEVEAMTAE----IRRSL--DIVAPTL------TVAPLHAES--RMAEMATRQFGEN---NLAD--------------PYRYVCLLSAYA-------------SDGQPVARGSEP----CFRTRLVENQG------------------------------------------AGAERSSKIDHCPFLFKCGKTAMLAGALQATIVVVNHDALLSASTNIPLEDGN-------RIRGRRSYQELLLRTRSIFLVDEIDGLLSRAIQSCTTELELGSKLQ--STALARLHSDVRQRSEVAGIK-------S-----TKLNNIKWALTFTAQTPDEILDLHAE------------------KH--FQW-PRHG---MRWPQSEDSSLQA---------ALKVD-GGEL-------------------------------RSLYQLNDRSL--PQH----------------------LHALQDNLSHWSIKTG--LQSPEDVLRQLTNI-LHALLTADRLKTGT---------------------------------------------------IITAELKGALVLRGALTLIQSQVRLLHR-----HIQELIW--ADIPYAREVQNALEGHPPISLNRNGPLQQPVCGFKH---------KIGERGGSTLHIETLKGDPHATLQLLPSLTSLAFAGH---ERIFIGLSATAHFS--------------------GAS-----RYDLPAIDLIDVP---------DASGQITFRDLPT-----------------TTRVSGTQ-IQARKQQVRQLAKELA----------LCLPGMLAAL----HDND---------PTRARIILVTNSDADAEVLAIALRNALNNDPLVNVTGIIQNTTSGHGNLGKGVLWLRGKEAANRLSDFPVAQ-AMLHHDLPMLLTGEHRDACILVSALSPIARGHNIV---HE-----------NG-R---SAVGSIIVCVRPLPPSDHPADTHAHICYENCKKL-------------------QSYATPGEAMEASRALAYHNLNAIR---NALPS-FSHQPEN--V----RHYTIMNILV-------DIAQLAGRGRR----GETSITCFFADAAFFEGTS------------------------------------TWATLLDASVCL------------MRKDGEWDDFRREHAGIATAMENYILLSRKES-------------------------------     403248865       -------MRPLSDIATTLFLAIAVRYVT--NE---P--------TLADAAAIIAGRTRKWSTW-GSLTEAQQTLIAQSLRLRPSLLAQPKRFMLSAEGILK------GKLSS------FELVDSSNLNTE--LHEQDRYRLSSHLDIDQVIRENFEALKNG--RTAP--AHGQLPNEGD----VVLKHIT---GD-----HVRVFAIPENERHVL------------NALDQHVLIPPSTALPEISWEHA-RLMKLAKQMDAS--------------PDLHSQHAASLVNIWGDEPVR----K----------ADSGNF-YRVNAPTGSGKSV-AMVMMAIDAAER-----G-------HRVAIAVPTLVEVENTVRI----IKQSA--AAVGKEL------KVAPLHSAS--RIYERAEMQFEQG---KTDH--------------PYDYACLLDVYS-------------ADLMAVEPGNEP----CFNIRISACGD------------------------------------------DGEESSKRLKHCPFLFRCGQTRMLSQAMEADIVVINHHALLSGTTRIPLSDAE-------QFPGPRSLVEILLRTTPVFLVDEIDGLLKSAIDSSVIELRLGNVGD--NSPLLRLFNHVVGKSHIPGID-------R-----SSLYRVNWALMYCTLSVSQLMNLQQE------------------RY--FEW-PIKE---TTWSDADDTFITQ---------ALGIT---------------------------------------RETLEYLF--SND-LGQLPHH--------------LQSLSRNLANWRSNDG-------EHRLETLAITLGNLIADLSESGRL-------------------------------PERL--------------KEHDRIRLKASLILRGTLLVIETHLRNLQV-----ELPAFVN--AEIPYAYEVRRSIAGPEPLSPSPNGPLQRAVFGFKR---------KDSSDQDSTLHVVAMRGDPHSTLLSLPDISALGYAGV---KRLFIGFSATAYFP--------------------GAS-----AFDLKAQDFIDVP---------DAKGQVTFE-----------------NVNQTTAISGGP-FAQRKFLVTKLAKELW----------PWLKARLEKL--ANDPET---------RDRARLLLVTNSDADAEALAMTLAKMADGP------GESVGWVRGR--------QSEYKPSSLEAQQ------MLVYDDLAEFTSGKHKHKTLLVSALGPMARGHNIV---NA-----------DG-L---SAIGGVVICVRPLPASDSPNNNLAHICYETGNTV-------------------LPRSSPGEVMTQERKLSNALLQTIR---TARPA-FSQQPAN--I----RHYTIMNILV-------SLTQLIGRGRR----GGTPVTCYFADAAFLKG------------------------------------LKPWSEMLNESVNR------------LKEDGDWEQFERHHAGIASAVQQYILRSRKESV------------------------------     346056321       -------MRPLSDIATTLFLAIAVRYVT--NE---P--------TLADAAAIIAGRTRKWSTW-GSLTEAQQTLIAQSLRLRPSLLAQPKRFMLSAEGILK------GKLSS------FELVDSSNLNTE--LHEQDRYRLSSHLDIDQVIRENFEALKNG--RTAP--AHGQLPNEGD----VVLKHIT---GD-----HVRVFAIPENERHVL------------NALDQHVLIPPSTALPEISWEHA-RLMKLAKQMDAS--------------PDLHSQHAASLVNIWGDEPVR----K----------ADSGNF-YRVNAPTGSGKSV-AMVMMAIDAAER-----G-------HRVAIAVPTLVEVENTVRI----IKQSA--AAVGKEL------KVAPLHSAS--RIYERAEMQFEQG---KTDH--------------PYDYACLLDAYS-------------ADLMAVEPGNEP----CFNIRISACGD------------------------------------------DGEESSKRLKHCPFLFRCGQTRMLSQAMEADIVVINHHALLSGTTRIPLSDAE-------QFPGPRSLVEILLRTTPVFLVDEIDGLLKSAIDSSVIELRLGNVGD--NSPLLRLFNHVVGKSHIPGID-------R-----SSLYRVNWALMYCTLSVSQLMNLQQE------------------RY--FEW-PIKE---TTWSDADDTFITQ---------ALGIT---------------------------------------RETLEYLF--SND-LGQLPHH--------------LQSLSRNLANWRSNDG-------EHRLETLAITLGNLIADLSESGRL-------------------------------PERL--------------KEHDRIRLKASLILRGTLLVIETHLRNLQV-----ELPAFVN--AEIPYAYEVRRSIAGPEPLSPSPNGPLQRAVFGFKR---------KDSSDQDSTLHVVAMRGDPHSTLLSLPDISALGYAGV---KRLFIGFSATAYFP--------------------GAS-----AFDLKAQDFIDVP---------DAKGQVTFE-----------------NVNQTTAISGGP-FAQRKFLVTKLAKELW----------PWLKARLEKL--ANDPET---------RDRARLLLVTNSDADAEALAMTLAKMADGP------GESVGWVRGR--------QSEYKPSSLEAQQ------MLVYDDLAEFTSGKHKHKTLLVSALGPMARGHNIV---NA-----------DG-L---SAIGGVVICVRPLPASDSPNNNLAHICYETGNTV-------------------LPRSSPGEVMTQERKLSNALLQTIR---TARPA-FSQQPAN--I----RHYTIMNILV-------SLTQLIGRGRR----GGTPVTCYFADAAFLKG------------------------------------LKPWSEMLNESVNR------------LKEDGDWEQFERHHAGIASAVQQYILRSRKESV------------------------------     386022139       -------MQPLSDISTTLFLALAARYVG--SE---P--------MLADAAALCAGRTRGWSTW-CALQDADQLLIAESLRLRPSLVAQPKRFLMSAEAIIKGERSPFELIDA------SKLSDE--------LHEQDCYRISPHLNADQLIREYFEALKYG--RATP--VYAQLPESGD----VVLKHIA---GD-----QVRVFVVPESERSVL------------EVADCHIHIPSSPAVQEIKWELG-SLRELAQQLDAT--------------PNLHSQHEASLTNIWGSEPRR----A----------ADSGNF-YRVNAPTGTGKSV-AMVMMSIDAARR-----G-------HRVVIAVPTLVELENTVRI----LKQSL--AVAAADL------KVAPLHSAT--RVYERAQIQFAQS---NTAS--------------AYDYACLLDAYA-------------SDLLDVEPGKEP----CFNVRVSTQEE------------------------------------------GRLEHSKRLNHCPFLFKCGRTRMLSEALEADIVVINHHALLSGTTRIPLSDAD-------QFPGPRSLIELLLRTAPVFLVDEIDGLLKSAIDSSVIELKLGNQGD--NSPLLRLFNTVVGRSNILG-----IDR-------SSLYRVNWALTYCTLSVSQLMNLQQE------------------KY--FEW-PKKE---TTWSDADDTFITE---------KLGIN-R----------------------------------EELEQLFNNTN--RIP-PH-------------------LERLSHHLACWRSNDG-------EHKLETLAVNLGHLIKALSDSGLL-------------------------------PAPL--------------KEHDQIRLKASLILRGTLLVIETHLRNLQV-----ELPAFVN--AEIPNAYEVRRSIAGPEPLSPTPNGPLQRAVFGFKR---------KDSSDNDSTLNVVAMRGDPHSTLLSLPDVSALGYAGV---KRLFIGFSATAYFP--------------------GAS-----AYDLRAKDFIDVP---------DAAGQITFE-----------------NVNQTTAISGAT-FAERKFLVSKFAKEIW----------PWAKARLQSL--ANDPKT---------QERARLLLVTNSDTDAEVLAMTLAKMQGGP------GQLVGWVRGR--------QSEYKPSSLEAQQ------TLVYDDLAEFTSGRHKDKTLLVSALGPMARGHNIV---NA-----------DG-L---SAIGAVVICVRPLPSSDSPNNNLAHICYETGKAV-------------------AFYSSPGLLMMQERKHSNALLQSIR---TARPA-FSQQPDN--I----RHYTIMNILV-------SLTQLIGRGRR----GGTPVTCYFADAAFLNG------------------------------------LKPWHEMLNESVHQ------------LKKDGDWDQFERHHAGVASALLKYINESRKDAR------------------------------     399519611       -------MQPLSDISTTLFLALAARYVG--SE---P--------MLADAAALCAGRTRGWSTW-CALQDADQLLIAESLRLRPSLVAQPKRFLMSAEAIIKGERSPFELIDA------SKLSDE--------LHEQDCYRISPHLNADQLIREYFEALKYG--RATP--VYAQLPESGD----VVLKHIA---GD-----QVRVFVVPESERSVL------------EVADCHIHIPSSPAVQEIEWELG-SLRELAQQLDAT--------------PNLHSQHEASLTNIWGSEPRR----A----------ADSGNF-YRVNAPTGTGKSV-AMVMMSIDAARR-----G-------HRVVIAVPTLVELENTVRI----LKQSL--AVAAADL------KVAPLHSAT--RVYERAQIQFAQS---NTAS--------------AYDYACLLDAYA-------------SDLLDVEPGKEP----CFNVRVSTQEE------------------------------------------GRLEHSKRLNHCPFLFKCGRTRMLSEALEADIVVINHHSLLSGTTRIPLSDAD-------QFPGPRSLIELLLRTAPVFLVDEIDGLLKSAIDSSVIELKLGNQGD--NSPLLRLFNTVVGRSNILGID-------R-----SSLYRVNWALTYCTLSVSQLMNLQQE------------------KY--FEW-PKKE---TTWSDADDTFITE---------KLGIN-R----------------------------------EELEQLFNNTN--RVP-PH-------------------LERLSHHLACWRSNDG-------EHKLETLAVNLGHLIKALSDSGLL-------------------------------PAPL--------------KEHDQIRLKASLILRGTLLVIETHLRNLQI-----ELPAFVN--AEIPNAYEVRRSIAGPEPLSPTPNGPLQRAVFGFKR---------KDSSDNDSTLNVVAMRGDPHSTLLSLPDVSALGYAGV---KRLFIGFSATAYFP--------------------GAS-----AYDLRAKDFIDVP---------DAAGQITFE-----------------NVNQTTAISGAA-FAERKFLVSKFAKEIW----------PWAKARLQSL--ANDPKT---------QERARLLLVTNSDTDAEVLAMTLAKMRDGP------GQLVGWVRGR--------QSEYKPSSLEAQQ------TLVYDDLAEFTSGRHKDKTLLVSALGPMARGHNIV---NA-----------DG-L---SAIGAVVICVRPLPSSDSPNNNLAHICYETGKAV-------------------AFYSSPGLLMMQERKHSNALLQSIR---TARPA-FSQQPAN--I----RHYTIMNILV-------SLTQLIGRGRR----GGTPVTCYFADAAFLNG------------------------------------LKPWHEMLNESVHQ------------LKKDGDWDQFERHHAGVASALLKYINESRKDAR------------------------------     329936081       MAVTRSRRLSKTAGAVGAALALAVHYFPREGAAGEP-----PVASFRDAAFLLSGQLDKWARW-RDLSHDEKRRIGAVVALAPQELASVTVFAARARELLNSAPGEPAALP-------FRPAGP---SAA--RAE-----AAVALVGGDLLEYVDRMLQRLH-RDHPRRRPDEPAGPGIWLPRTQYAAGS---GL-----IQGRTVIPVFADGDDDEA---------ADRAALPEVRTVPYQEEVALPAG-ELLELARLIDGRY-------------PAADRYVHTVLANLFAQLQTT----DAVPAQELIR-LAAGPM-DILNAPTGTGKSV-LVRVAASWYAVN-----G-------LTVTLVLPTVEATLSAAWD----ISRDL--EQLGRTE------TCTPLMSPS--RLHERAMKVAARI---EGSLLEQPD-KTLW-KLDQLSYGCALSHHT-------------QSSHPYPPGQEP----CRGLSPLPPAT-------------------------------------------------DRAACPWTATCGKYAQHRQACTASVVVTNHHNFMTGHFPLGIRLDG-------RAVADVSVAEFVLRRSHTVMIDEVDQFQSTALDLCSSELVLDSRRQP-TVPLRELDEDKAKLSPEAVKA----LCPT-----ISHARYLSEFLLASICEDVLHLRHYE----------------GHGP--SR--ERPGVNSTGWHLAGSRDRRL---ITLLFPDEGIT-SEQEIP-----------------------------ARLFDELNALR--PASPAGGGGTDTASAGDPSLAPH--LAAVRRLLGDLLAPRG-------EDLLASVQLELNEVLLDVVKDPHD-----------------------------------------------------RSEAIELLIVRMWLAELDDTLALLRN-----KTGQLLS--LGMRSARTLAQRLETGVAAHILPYGMLGKAITGYRV-------TGLDNPHKNAELTAQSISGDPHTYTAQLGSIVSLALAGV---ERPVLGLSATAYFP--------------------QAV-----REHVHADVKWWMT--DAAPDSIRAKRRMITDSVTQR----------------AIQISGIP-QQHKREALIKLGDRLY---------DTVIHDELARI----ARTD---------PDRAHAAVVVNSYEHCRHLALGIHSAGQYT------GGLCVAVPADRH------WRAKLPPLPPGIT------ELTPDEFEDFP----RKGTILVVPMARIARGLNIV----I-----------GT-K---SAITPVYLCTRPLALLTDPAEMYASVNAAGIGSLPF-----------------TPTSTPVGDLREARAAAWARMGMIM---RAAPG-FVSTAHV--L----QEEIVAGMVV-------DMIQLAGRARR----GGTDMTLHLVDYAFHEDSWQSDLA---------------------------GILRRMHGRWSP--------------------EVRRQMNAIYREALAAFLAYAGIEPDDT-------------------------------     297204965       MAVTRSRRLSKTAGAIGAALALAAHYFPREGEAGDP-----PVASFRDAAFLLSGQLDKWTRW-RDLQHDEKRRIGAVVALAPQELASVTIFAARARELLDYAPGEPAALP-------FRPAGP---SAA--RAE-----AAIALVGGDLLEYVDRMLQRLH-RDHPRRRPDEPAGPGIWLPRTQYAAGS---GL-----IQGRTVIPVFADGEDEA----------ADRAGLPEVRTVPYQEEVALPAG-ELLELARLIDERY-------------PAADRYVHTVLANLFAQLQTT----DAVPAQELIR-LAAGPM-DILNAPTGTGKSV-LVRVAASWYAVN-----G-------LTVTLVLPTVEATLSAAWD----ISRDL--EQLGRTE------TCTPLMSPS--RLHERAMKVAARI---EGSLLEQPD-KTLW-KLDQLSYGCALSHHT-------------QSSHPYPPGQEP----CRGLSPLPPAT-------------------------------------------------DRAACPWTATCGKYAQHRQACTASVVVTNHHNFMTGHFPLGIRLDG-------RAVADVSVAEFVLRRSHTVMIDEVDQFQSTALDLCSSELVLDSRRQP-TVPLRELDEDKAKLSPEAVKA----LCPT-----ISHARYLSEFLLASICEDLLHLRHYE----------------GHGP--SR--ERPGVNSTGWHLAGSRDRRL---ITLLFPDEGIT-SEQEIP-----------------------------ARLFDELNALR--PASPAGGGGADTASAGDPALAPH--LAAVRRLLGDLLAPRG-------EDLLASVQLELNEVLLDVVKDPHD-----------------------------------------------------RSEAIELLIVRMWLAELDDTLALLRN-----KTGQLRS--LGMRSARTLAQRLETGVAAHILPYGMLGKAITGYRV-------TGLDNPHKNAELTAQSISGDPHTYTAQLGSIVSLAMAGV---ERPVLGLSATAYFP--------------------QAV-----REHVHADVKWWMT--DAAPDSIRAKRRMITDSVTQR----------------AIQISGIP-QQHKREALIKLGDRLY---------DTVIHDELARI----ARTD---------PDRAHAAVVVNSYEHCRHLALGIHSAGQYT------GGLCVAVPADRH------WRAKLPPLPPGIT------ELTPDEFEDFP----RKGTILVVPMARIARGLNIV----I-----------GT-K---SAITPVYLCTRPLALLTDPAEMYASVNAAGIGSLPF-----------------TPTSTPVGDLREARAAAWARMGMIM---RAAPG-FVSTAHV--L----QEEIVAGMVV-------DMIQLAGRARR----GGTDMTLHLVDYAFHEDSWQSDLA---------------------------GILRRMHGRWSP--------------------EVRRQMNAIYREALAAFLAYAGIEPDDT-------------------------------     357401427       MNGELTGAEAGLIGALALASHCMRRKDA-DGNVVAS---------FRDAAYFAARKPQAWDQW-PMLTLAERRVVRQVGQLMPAEWCNPKAFKSAVLAFVGKLA---PEGDG------YDRSGG---TVTLVGGDPFAAEAALLKIGGDFLGHAERLTGRFFTPGKRP-KAAEFAGPGVWKTRTTYLGQE---HG----VTSREIEFPATPLFEKA-----------PGHEALPRLVTAPACERISAPVE-ELLEVAQWLSER--------------DESVAYLHTVLKKFLSDLKSS----ASGELSELDL---LSGLLQVLNAPTGSGKTV-LVRVMASWAVLH-----G-------YRLAIALTDVRATLNMAWD----INNDL--GWLYETGKLDKLATCVPLMSAA--SMHKRAMDYAALT---PSAKITEWNLRGQR-DIAHLAYGCAQRVLM-------------DPPDLYPPGEEN----CLTLIAPAADS------------------------------------------------AKRHACPYLPVCDKFRPVYEAAEADIVVTNHANLLDGALRVGAVIDGHEWRGQARGTAGVSALELALRSFDALIVDEVDAFQKTAIGRCTSSVVLASRKR--DSALREIDQDAKRLPSENQEQ---ILS-P-----VSHARLMAEFLLLWLCSRAL--KLNP----------------GNEA--EGW-GAAGRDNDGWRLTHSRDREI---LQLLFPDLAPS-ADAVP------------------------------PQLFRFLEDIM--PER-WNAPEPADSTDHPASAD----WGALQRALAALTSPRS-------ENFLSLVQQALHGLLSEAVPVAND-----------------------------------------------------RAAVVNLLATRTILRDLDVSLDNLRR-----QASALSH--LDLASVRKILDGLHKSTVTVLYPLSTLGRSISGYQI-------RGLEAKESEAELLSRCFEGDPHTFVSELGGLTALMVAGV---QRPVLGLSATAYFP--------------------QAV-----QEHVHAPVKWWLP--------DTRPRSIVTRATPVIDPNSG-------DEREALRVGGIW-ADKKPAVLRDLGRLLY---------EQHLDRRLRRLEERNERRD---------EDRARVILTANSYEQCAYLAHGLAQAEGLR------HRICLLVKDFHKRD----YEQHLPSHVR---------RMVREELEAFP----KHGEILIAPLAVIARGLNIV----V-----------GT-R---SAVSEIFLCVRPVLSIEDTEWIHGSVNAAGINALPS-----------------GGCADPLLAIEKARDASWRQLRRIL---HSPSR-FSQMAHE--L----QEELIAGMLV-------QLVQLAGRARR----GETDMTLHIVDHSLHDTKFSSDLA---------------------------SIIQRIHGNWTR--------------------EQKRIMNELYGQALQAFLSYAGVPDADL-------------------------------     295837641       --------MTGTEAGLAGALALAAHYFPRKDDEGHV------LAGFEEATFFAHRKPQAWSEW-ANLPTTERNLIRQVMTLMAQDWTDPKKLRTSVLAMLGVL----SSEPD------IADRSGGSLRLP--PGDPHRADAAIPRMGGDFL-GYVQRVTGPFFTFGKRPKAQAFAGPGKLKTRTAYLGPE---HG----TTSREIVIPATPDFREA-----------PGHEFLPQVHTRPPRDRVAPTVE-QLLEVAKTLSGQ--------------DKSVRYLHKVLKRFFKSMKTT-----TGALTELD--LTAGDL-QLLNAPTGSGKTV-LVRVLASWAALN-----D-------RRIALGVTDVRATLEMAWD----INNDL--AYLHKIKRLIEPTHCTPLMSPS--SMHRRAMDYAALG---TSTQIDEWDRRART-DIGLLSTGCAQRALM-------------DPPTLYPYGEEN----CTSLTSEATGS-------------------------------------------------NRLTCPFAPTCGKFQQFYRATNAAVIVTNHANLLEGRTRIGVVLDGQEFRGQARGTRGMSVLEMTLRACDALVIDEIDAFQTAAISRCTSEITLASRKR--TTALREIDSDAKNLPVVHEMG---LVS-P-----VSHARLMAEMLLLWLCSGRGLKLNPG-----------------NEM--DGP-DGAGRDNTGWRLAHSRDREI---LQILFPEATAA-EDIP-------------------------------PELFAFLNEIM--PARWYGSEPDEEEFDLPEGAD----WDAVQTALMTLTAQRG-------QDHLTDTRDEMRKLLLGLVPDAHR-----------------------------------------------------QAAVINLLVTRTVLRELDGALDELRA-----QAQTLRY--LDLGSVRKILETMRSSTVATLYPLAMLGRSIHGYQV-------KGMDNKEKEAELLSRSFGGDPHTFVSELGGLTSLLTAGV---QRPVMGLSATAYLP--------------------QAV-----QEHIHAPVRWWLP--------DTRPESIVALATPVR---S--------GAGDAMRIGGLP-PELKPNALRDLGRGLY---------EQQLARRLAYL----EKRE---------PERARAILTVNSYEQAAHLASGVAQADGLN------HRVCLLVKKAEKQN----YEEHLPPHVD---------RMVREELKDFP----ERGEILIAPLAVIGRGLNIV----V-----------GT-R---SAVRDIYLCVRPVLSIEDTDWLHASVNAAGVNTLPV-----------------GGSDTPLADLRRAGEASWKQLSKIL---RSPAR-FSNMDHD--L----RKELVAGMLV-------LLIQLAGRARR----GETDMTLHIVDHAIHDQKFSSDLA---------------------------TIIKQIYADWNP--------------------EQRAIMNELYGQALQAFLTYAGLDREML-------------------------------     302559388       -------------------------MFP--NRSEEP---FRHASAIRDAHFLLSGHTHLWNRY-GELSCEDTRRVTRLLWHRPARLAILAAFRRAVEDLFDSP----DLPEG------FAALDD----EH--YLV---------TVQQRLASLVDQVLEELD-------GRRAAADEGR----SRDLALQ---GH-----FRIGTQIPDPSRPGNGVIQAHYVLDLPQIQAPLPDFGKPKECLRLQVPID-ALRAIADAQDRAF---------------GQSHRRKSLERLFKHIRRA----DGSLLADGFLILDAGLI-RVLSACTGSGKSV-LAKLLAAWGVKN-----N-------LVTGIVVPRNDSVLSFTRAIREEL-HAL--GLQDA--------VVVPLISPN--SMQEEAEKVARSL---IEEGRAQEADE----AYGEFAYGCAMQAKS----------TNTQDVDLWQPGHER----CSDFEGTDPES----------------------------------------------GETRRHRCPWFAHCGKHRHQLNLDKADVIITNHINLMSGRLHIPVLAEG-------DVRNHMTVEEALLRLTHVLLIDEADAFQATGFAKSAHHVTLARYGSRQPSALQELHDQFSIRAGALMYELERYIHPK-----ITQARFLAESYVSNAAHGRIAQRGPN----------------ERNR--QQL-ARRR------LIIPRRWDAWCTARLWRAPDGKSP-TREQI------------------------------DTFRKLFNPKE--EAQLTGARVPVVPGVGESTREH---LTQLRTELLDATALR--------EGTDPVFDNVHARIAAAARPLLDE---------------------------------HL--------------ADDDRELLVDLLVRRAYLEQIRAQIEYVTR-----SGTSLKA--SGITAANELTEMLEDNNQWHAAPYGPLGAPVFGFTA-------IHDIEDKHRTELALTSFAGDPHAYTAQLGDTTALALCGQ---RRIVLGLSATAHMP--------------------GGT-----MHHLITPPTWYVP--------DGITGSLVLRSLTLR---D--------ERNNPIRISGTS-DIHRDQAHQQMGRALW---------SQHIVQHLKKL--ASRPAT---------AHRARILVACTSYVGAAQLAEGMISAGADP------EMIAVATRAS--------DENTVPAHYRRRPAWY---EIPSDALERFP--HHPTAKILIAPLAIAERGLNMV---DH-----------SG-R---SLVGEVILAVRPIPLMDEPAQLLALISSRAYSSV-------------------TRSADPAETLRHLAITSSSVHEELF---RSHHF-FQSLPNK--V----RLSIVAEMLI-------GIIQLGGRVRR----GGDRGVLYLADYAFHNPSSGSDLP---------------------------RLIRELREGWSRS-------------------GELELLQNIYGSTLRAIFDFADERTSN--------------------------------     254390289       -SVSDAPGTRSFPKLMGAAIGLAASFFP--VD---VGTGHTRTTTRADALFVLDGHIADWSGW-SALSAADARRFTKLLRRRPNQLANAEAAGRVLDRLLAG-----GEIPG------CRAADD---YTGDGNSR---LLVEPGMPLPAFV---DGFLRDLDVLYAGRQRSLPSAEAGPHTTTTMIPGLD---GS-----SQKYVDFRYEIRPHHV-----------ARTVSAPTLMDAPALLDCWAPFS-ELRDIAARIDHGL-----------DGTGGITFRQKAVDQFAEQIRSR-----EGTSVTGLR-LTAGTL-NQLLAYTGFGKSVVLVETFACWAVTQ-----G-------IVVAFVLPTNADVVKAAYA----VERAL--RLLDEDMDK----SVIPLMSPR--ALIKVAEASSARA---SAQGPDADW------IWRRFGYGCALAAVA----------EASGGTDNWQPGREP----CAKLRSPRPRG-----------------------------------------------RDRKVACPWRKTCDRARQAREACTADIVVTTHANLLLGRVQTPVNDGH-------GESDRLTVEELVLRRCQIVVIDEVDLFQQFAIDRAARGLVLDQAGRT-NTPLRNFDRDFGEAFGRLREETDASVRDA-----FVNLRYLSEIYVSHLTYARLGATGRP------------------AR--RRP-RGPGRF---WMVPRRWDNWL---AGRLFGVEPDD-VSA--------------------------------DQMNVFCSLFP--DAPNLAPDAPRGFAEARRQ------LTLVVTPGAGGPAVA--------TARAALERL-------TTAVPEGD-----------------------------------------------------RAQAVNRILRRAILERIRISLHSLMA-----NNAQLVD--VGVESAQEIAEALGSYNRWQLTPTGPLGRLVFAFTE-------YFDDTGADPAQLMTTAFGGDPHSYVVGLGDTTALAHSGV---RRIVLGLSATSYFP--------------------GAP-----HHHVHTRPNWWVA--------DDTPGMVRILPAPIL---D--------EEIRPRRISGLE-GSARDEAITTIAGSLW---------GTYLRAELERL----RAED---------RDRARVLLATTSYLAARQVAEGLFSAGVDA------SRICLAVRPSEDEPGA--FMGMGNGRWR---------ELPADRLETFP--QLEGADILIAPLGRVQRGVNII---GR-----------DD-R---SALGSVWLIVRPIPLIDEPPELLAHIQAKALAEHS------------------GPSSDPLALLAERRRTAGTYLDEIV---RRPPY-FQAQPDE--V----KLGVVAEIIN-------GAVQLIGRARR----GGTPAVLHLVDGAFHDGHGGTDFA---------------------------TLILQLREKWRR-------------------DGVLNDMRAYYGTTLEAFLTYAEEQSSGASSC----------------------------     407881734       ----MTTKGKVLPRIITAAIGLAARHFP--ED---P-DGGYAIVTRDHVLYVLDGHITDWDRWRRRLSDMDAMRFARLLRKRTNQLANADMAERVLDELLKG-----GEIPG------CEPADDYDDMKN--PRI----IVSPGMPLTDFI---DQALTELDELYVANRKPLQPATAGTYTTTTRIIGAD---GT-----DQTYVNFRYRIEAHHE-----------EPSRPLTRIVDAPARSEVVVPFD-ELEVIATRLDKLL-----------DDVASDGYRATSVRRFEQQVRDR----AGGEIKELD--LSAGEL-NELLAYTGFGKSVVLIETFACWAVEN-----K-------VSVAFVLPTNADVVKATYQ----IGRAI--KALGNETA-----SVVPLVSPR--SLIKVAETAAARA---SDGGPSADW------IWERFGYGCALAAVA----------SSDKSVDGWVPGREP----CAALRQPVPHR-----------------------------------------------KDKTFSCPWRTTCGRYRAAREACTADVIVTSHVNLQLGVLQTPVDDGL-------RRNDRVTVEELLLRRCQVMVVDEVDAFQRSMIEQAGRGLVLDHGGRT-NTPLRRLDQDFGAAFGMLSDEVDASVRDA-----YFTLRYLAETYVSHLRYERLGASRKN-----------------KNR--TRR-PGPG-RD--WIVPQRWDNEL------ANQLFGQP-LDTEPTDGQM-------------------------RLYRSIFPDQA--EPE-PGDPPKYQEMRDV--------LRKVVTSGLAGQAIV--------AARVRLAEF-FDLVEDEGQALVAN-----------------------------------------------------------CVLRRTILERIRWSLHSLMA-----SNAQLVD--IGVESAQDIADALGTYGRWRVMPTGPLGRLVFAFTE-------YYDDSGNDPAQLSTAAFGGDPHLYAISLGDITALAQAGT---RRIVLGMSATSYFP--------------------HAP-----HSHLHVMPKWWVANVGKKDDGDTDEKKVTIVAEKVV---D--------KAGNVIKVSGLD-GKDRADALVRLAKLLW----------PRLDEELTGL----AVTD---------WDRARVLLATTSYDGAKQVAQGLSEAGVPA------SRICLATRPR--------GEGGAGSAILTGQWW----ELPADRLESFP--SIEGADVLIAPLARVQRGVNII---GT-----------GD-R---SALGSVWLLVRPIPLIDEPAELLAHIQARALTCHR------------------GPTGDPLGLLEERRKKAGTYFEQIV---NRPPY-FRCQPKE--V----KLGVTAEIIV-------GAIQLIGRARR----GGTSAVLHLVDGAFTDGSAGTSFA---------------------------SLITALRDEWRAS-------------------GELGEMERYYGRTLRAFLEFADRPDTGDQPC----------------------------     117920627       -----MVPKWKGPNVINWLCVFMSHYLG--TS---C---------LKMAPLLLSGY----RSVASTPQMSGHEEALVNIRRICFHLSNISAIRKCIIRYNEHHAS--NQTMGSVDRRLFKINEK---------------TLEFEFAFDHPPVEFNDAVQHLAMTYQPSAHSVQFISPGN----AKHFIQI---KDTNTSFTYEIKGLP-------------------KQVAPLHHDLSPPKKDPIKIPVS-ELRELARQMDDVD----------KERGYRLGNWENRLKNIICEVPQV----D-GKFLVTEV-ITLDEM-KHLIGLPGSGKTT-LLVCITKWLSEK-----G-------FRTVMFFPSIEVCRQYMTT----L-QRY--DV-----------KASLLMGRSRNTRKRHAHQLAESL---ASGDHLNGFASTLP-GAKYFATSCPLPAYT----------TA------FDAGLVPEEVFCEDVLEKNEDS----------------------------------------SSRSSAAKMQSRLCPLWSQCGYQLAPRELVEANVWLGHIASA-DTEVPKHTLTQK------------MRYFELIARRSDVVIFDEADKVQSHLDEQGIATLSLTGDA---NSFNADLLNR-HRELAAGHNY---TLG-D-----PQIFSFYVSTLDFSRCSHLLVNAIQS----------------LDRL--QMA-RYEGSLLTPARILGELISGN---NSKKVKVASDR-DSLF-------------------------------RKKDALSQLWE--SAA-VHAFINRLGLSEEVIGKNLD-VAFIASSLFISKEDV--------KERHNVLVRAFRIWLSEEHDAGLD------------------------DALTEILDTIN--------------PYIPEDRVVQKIQQVRLLTAVSFTILGYRK--LEPHANALIE---QGFIEPLNIDQRCSADLLKHTNDNILGG-LSGVRFFSSEGATSGNRAGSKNIRLQYVLFAGSPRAYMYNLHK--FKMNAGS---NPKVLLTSATSFLE--------------------QSP-----AFHVTKEPDYIVR----GLEKKKAVTQSRFVFLPIKASA---------QGKEYLRYSGETLEKKRKDNLERMVFHLL--------NNRVVEDAINTF-----DSG---------LKKRKAAFVVNSFAQCEQLKMFIDMRFPEW---R--SKTVALTKDV--------EKHSGKTG-----------YITASQVEAIG--DDDSIELLIFPMGAIGRGVNIV---FT-----------NGERPRDAAIGCLFFLTRPHPSVDDLSLLVSIAGRRSEAFNQR------------NFSSVASLEEVAECLKISRKETYFQIGRLL---RNPLM-ASRLGKL-------LEPFTANIAV-------ELLQTIGRGMR----NGCPVQCFFVDAAWAQMSCHGEKD-----------------------TPVSSMLVQLIDILTECIN-------------HPDPKHAAIYTRLYRSFLDPLVETENLLYSRQSDYEDPTVQENYQPGCFFQEGLE--------     158336200       --MRVKPNFWTGPNQICWLCVLMESFVG--SE---S---------LEHAPAVMSGM----HSILNASALIEARQAIFNMRQMSLAYVTQRSVNHAIAIYNNHHYL--KGTQGS-----YQIDAQ---NLT--FER-------TNPLEDSLITQAKEILRAPL-SYQP--NTTKVAEVRQ----AMSVALG---TD------STSTRIPIPPIEA-------------KIAQRHYHPLNRQLQDNIRIPLE-DLKVLAVKMDDRE---------ANYPERRPGRWASRLERFMLTVPTA----D--GLQPTDT-LTLAGI-KHLIGLPGSGKTT-ILILIAMWLAQE-----N-------YKAMFVFPSIEVSRQYMDV----L-QFH--AV-----------KVGMLVGQSDETRRRHADNLAEAI---AAAYPNRGFSHTLG-VADIFSLNCVLPAFS------------NADTSMWGFGYAP----CNAVLQSDSKG-----------------------------------------------ELKPCLCPLWTMCGRNKSVRELTTSNIWVGHIRSM-DTPVSSHAVQVR------------IRYFELIARTFDVVVFDEADMVQSNLDAYGAATLSISGSE---KSIHRTIQEQIHSRFAGKDNH---RLA-D-----PDIATFSWHLSDFGNHNSALVTTVQN---------------LNESY--IGE-KYENQLLTVLKIVSEIVNEN-------GQNRLNP-EEKKPPTDEGRRLKV--------------------NRSRALTDFWE--TAA-YNAFYNRTSSNRDE-------WKNLSFNAATLRISET----ELEEKRDTLIQH-FQRYLAEGLAQRRD------------------------EISQEISQFYL--------------SICFPDHSSTGKESDAIKLLIVVTFVILGYQRIIPGTRALVA---EGLIRDPIIQSTASRSLRRMIPSSLLGS-LSGVKYTFSKA--QTTRTNARNVEISYVAFVGAPRMLMYRFHQLLSPDNNHR---GPATLLTSATSFLE--------------------ASP-----AYNVNIKPDYLLK--PLEPLYKTEPSRYEFKWQADK---E--------WRSQPLRYSGA--GESRERNLKKMIEELV---KGGTRNEDARQSEIYKSINNFDVRG---------GQKRKAALIVNSYEQARMVKTFLNRYYPET---G--RRTKAVVRFL--------KEGEKSDD-----------YVTTGQCESLG--DDDSCDIIVFPMLAIGRGVNIV---FT-----------KGPRMLDAAIGSIYFLTRPHPTSDDMQLLYSLAGQATQDFDSR-------------TFGEEDVDAIANSWQQARKDLWRTANQLL---REPIM-ASRLSPE--L----FKAFTANQMV-------AILQTIGRGMR----NGCPVAVYFVDAAWAMNSALGKPD-----------------------SGRDSMLVQMRSILEDCIN-------------HPDPTDRSIYQELYGAFLEPLREIAGVKYPKEPQHLSDETPETDDFDSYSHFYEQ--------     126655717       MRAKPKPDFWRGNDRICWLCVLMEEFIG--VQ---S---------LEYVPVIVSGM----ESVFNAPVLEGARQAIFNIRSLSLNYVTRNSVKYAIALYNDLHYQ--QGTQGV-----YEIDSQ---TLE--FKR-------TDFLEDPKITQAKEILSAPM-AYKF--HGKTTANPQQ----QMVIALG---DR------VTSTRLPISPITA-------------PLASRKTHNINRQPQGNISIPLS-ELHQIAEEMDRSD---------REHPERRPGNWTKRLEHFDLLVPEP----D-RGLKTEIR-IELSNI-KHLIGLPGAGKTT-LLMVSAVWLGKK-----G-------YKVMLVFPSIEVARRYMAD----L-KFY--GV-----------KVGMLVGQSSVTLKTHAERIAETI---AASGGQGGFAYTLE-GADSFACNCLLPAFS----------TVETS--QWEYGKAP----CSSILQS-----------------------------------------------SSQGKMKTHLCPVWTMCGRNKAPRDLINADIWVGHIRSM-DTTVPYQAIDEE------------IRYFELMARTFDLVIFDEADMVQSSLDSYGVAELNLSGAE---ESIHRTILEQVHNPLARTENY---RLA-D-----RNIDLYSRNMAEFGNHNTSLVSLLHN---------------IHPRL--KK--RFQGQLLTVSRIITEILEGF---DQNKFYSLRDT-SADVEITKGF-------------------------KKSHALTDFWS--TAA-YTAFYDRTGTETVKKSY----EKDLCSRTLSIDQKK------LDANWHKLLKL-LRRYLAENLIKKRD------------------------EIVDDITILFL--------------DICFPNREPPHLTREVIPLLVAITFVILGYQRTVPETKTMIA---EGLIKDPILNPPLSHQLRCLTPENLIGR-LSGVKYKEQNA--HTNRNNSSNISLSYIIFVGAPRMLMHRFDR-LLAADGRKD--APAVLMTSATSYVE--------------------ASP-----AYHIDIYPNYLLK--PKVNQHSAESSKYYFQYFLDK---E--------RRNEPLRYSGA--GELRDTNLRKMIDELV-----KNGEKSEVRKAIRNF----DVRN---------GIHRKAAFVVNGYQQVRDIKKYLDRNYPDL---G--KRTKAVVRHL--------EEGEKPSD-----------YVTTSQCEALG--DDEACDLIIFPMLAIGRGVNIV---FT-----------KGVRERDAAIGSIYFLTRPHPTTDDTQLLYSLAGRATQAFDGH------------TFSSEDQLADINLAWERRRKDIYRDTRRLL---QEPLM-ASRLGAE--L----FKPFTANQMV-------AILQTIGRGMR----NGCPVSVYFIDAAWAPQSARGQPD-----------------------SPRDSMLVQIRVILEECLK-------------HPDSVIRQIYQELYGTFLEPLRKIDKVICPPDLFQSEDSVYEDDDYEDFDPLLDM--------     153817615       -----KELRITQILQIEAALQFCVQFLPHEGG-------------DTQLVPILSENLPRLVDWRETGGASRLRSLL-----LLLPKMSNQVWRNNLLAYQKLP----AAHRC------FDIFEN---GSK--FSP-----LELGDYNRK---MFLTSLFDTKIEAEP--AEVHFLEAEKDGIPNHSVRVR---YSRSGQQLECSYRIPQALFRKASVV---------TLPKPRQRKMPRDHRTDITITKS-ELVIRGELLDEID----------RNQKRNSHQIGRRCANLELTGVGK-----------ADNQITLGQQPEHIVGKPSAGKST-LVRSIVPELAAR-----G-------YRMAVIVNSTAQAQIQSES----F-CAH--GV-----------KATAWCGWR--SRQDHAMRRY------LSQEQRIPIRDGFP-DAGNLAGGCLLRACQQGNEVDLSQISSPQRLPPPGASDSI----CRALHDPRNPD------------------------------------------------SKPYDCPFHTICPSFAQERATLDADVVIITAQALNNMRPSPFYYPKC------------NTVIEWITEYIDIVIVDEVDGIQQLLDESQSLEQQMYAGEAY-GNILAEMTSRHSRLRADGGAHQQLEACLNASTILERYIRWGANLVADASTRDLGASVFKR-------------GYNEHTV--LTT-AALELYLNNKALLESGLSSAYKSISNILELTTLI-EREYER-----------------------------RSIQDIECSFYQELPPWLAEAHDRFPTKDLSAGWS---LARLAYYIQNHIRQDE-------RPEQRAVEY-CTKLWNESTEEGSLR-------------GGLKLFSHWVEAALTEGEQST--------------REHQRQQLEKDLQILLVIAVMTRLAMRTYERLSYPAQQLASE--DLAMPAAIGKAARLERLYRTLLPSTLIEG-SSKFEY---------RKEENDQHSIIFRRLLAPGRSLLYHLPY-LRESEGIS---GPHLLVLSGTSYGGRNMRYPVDHPYAKRAYEPSLASP-----DFDVDLPVSVILE--QPKSERDAITERSVFEPLMLK---D--------ENGKPIRVSGS--GSLREENATLSAKALL--APAIGEASSMVEAHFADAQQRWGDEF---------TGRRRAMLVASSYQMVEELMEELPARLPPN---R--WTLVGVRRDE--------FKVRDKVNLLENCS-----WLSAADVEDFG--NFPENSVLIAPLSVISRGHNILCDSPT-----------LG-R-KVAAISHIYFLNRPHPTPTD-QSALRGLHNRKINALSL---------SSFQQQA-KPGENTVKLISRLRKEAKTRYQKAL---NQQLP-IRQMNEE--S----RLRILYRPLV-------QLWQTVCRGVR----GGVPVYAGFTDMAFHPLSSQGKED-----------------------NTSTSLLLGIDELLSRLLD-------------EKWNPDAKIAERLFDTPRIAFAKLRGDLVALSESTKDDLIENESSNIDELIDDGYEGDNEYSI     210630743       -DPKEGCRIARTITDVEQLLVLSRRYDG--PH---R---------ADKANLLLCNY-----HLLQPESFGEREHALQMARRHLRHLSSGYSWANKLEAYMEDET---RAFRM------FDMVDG----NI--VRRQTAFGEGPDEGERRED-LYMVCVLEPI-PCKP--RGPQVKAGVR----HTYWAKP---EREMRDNPRSASDTPPRSPRSISIPPAMLEMAKAAESRKVNDIPHKGVRPAITFTLD-ELISAAREMGELT---GQTHLALVLETALERGLFKRLEGATASAAEG---------------ITIERA-INMVGIVGSGKSV-FANVLTYACAKR-----G-------LRVATVHNSISDVIESFEL----F-ESL--GI-----------EASPLISKN--RRLEHLDEL-------ASKTGGMLLDNA---VARYLEAPCLLDGMA----------EHADA--PTGYGDCP----CFGLKDP---------------------------------------------------RGNRRACPFFDICPAQSMARAALSSRVVITTTAGFALTTVSA----ER------------QPFFEHALADFDLVIFDEADRVQSQLDALFAPSESF-------GAYIRESADAVARALKRPPRE---KVD-D-----PNLEYL-HDLRGSSDSIAKALSAEAR----------------KPAI--AEWKELKGHTFTTLSLLEMLKG-----------TDEAP-EDQRLP-----------------------------DSLIEDLQHCI--DTR--GDYRREDEDMGDVY------LRSAIHAVRDGANDA--------HFRQEFDSY-LKQRGVELSPTLRE-----------------------------------------------------------RLAFALKAVSFDGHLHDLDH---ASDMLAFKDESIEMLYDFIHASTS---RQAPYLPASPVGN-LCGFKI-------------TDEHDIELYRQFGTGRALMTALPWLDTDEQGNPC--GPHALMLSGSSYEP--------------------GCL-----QFHINQPVDYLLD--AQPEFAEFLTRSTVRDLEP------------------GVAVSGS--GPQRGENLRRLLKSVV----------DTLVFEMDD------------------PHSHKALVIVNSYEQAAMARDTLQGELRRR---GRSERVCRLVPH---------REGAMPDPFSLENDD----SLPRAEVHRFA---HHEARILVAPAMAIERGFNIV---DN-----------LG-H---SAIDTLIFAVRPMGIPQDLVVRFKRMVGLICEQSR------------------HLDSRPPSFEREIREGAWRCWVTLERDEALRLSDHAALDDY--L----TRDIIATLMV-------LLVQIFGRLARVRDPERRPPHIYFADSAFTGGQDTTRAS------------------------------FRTLELLISYMNEL-----------IGGSGQPVVAQALYGPFFSALTKGIQP------------------------------------     257790574       ---EDSDLSLDEFLNVESLLALAVRVCGPGAD---P----------RKADRLLSNY-----QLIRPETIGADGHLMQMARRHLFSLASSTAWRRLLGLYERAEY---ERYRF------FDIANG---GMA--LRE------HPLTGIDRMPIYIDRLLGDVKLSH----KNVVARPKGR----YSYTCKPEATGDTTVTGSIRWVTIPDAVP---------------PQTGKIDDIPRKSRRPPIDITLD-ELISTADEVGEKT---------------GKTHYAAVLRRVKDQGLLK---RARGGSTSVADGLRLDEV-VSLVGLVGAGKSV-LANMLIVCLAKR-----G-------LRAVSLLNSVSDVMESVVL----L-REA--GI-----------SASPLVSRG--RRIERLDEF-------FDHDDSMLLDHS---ASKYLETACIMDGLS----------SSDPE--ACGYGNTP----CRGLRSK---------------------------------------------------KGGANSCPYWDVCPSQAMARESLTSDVVVTTPTGFATMIVGR----ER------------KAFFEEALQQFDVVLFDEADRVQAQLDGCFAPSMSF-------QELIRNAADPTAVAVKRRPDD---KMR-D-----FNEELFYDLRQKSEPVAKALLKSVRD-----------------DRV--AKWRIVKDEAFTSLSLLNDLL------------EQGLP-KQVY-------------------------------EDADKLINPYR--FEI-AKKSLDGGGAALNK-------LSQAVATSCEGIDDD--------THSYSLNEY-LAACGCSELPDELR----------------------------------------------------------TRFSFALKVIRFDSYLRELAS--AQDLLSFKDD--SVDELYNFLKFSY--TRQQHYLPNSLIGN-IFGMKL--------------DGNDLRLFRQFAFGRAFMCSLPWLDTDPAGAAL--GPHVLLLSGSSWEP--------------------GCL-----QYHVNRPVDYLLE--AEPWKAAKLSTSTV----------R--------DLGIEQNVSGSA-AEMRSGNLGIVLSQTM----------ATLRDELDA------------------EGAGKALVIVNSYREAEDARDRIEQEFRRK---GQAIKVAALVRNN--------HDHREH-------------FVPRSEVYKFC---DHPAKVLVAPAMAIERGFNIV---DR-----------GG-H---AVFTSLIFSVRPMGTPHDLGGRYRKLNGLIEREVG------------------DYPANPGEFATEVRASAWRTWKTMERDENLPMGAWRTMGRQFLV----DDAIS-TLMV-------TIIQIFGRLARLADKERPAPHVYFADAAFRGGDGK--------------------------------LSFRTLEELGAYMERL-----------MHDSDQPEVAKALYGPFYESFRKGIGNVGL---------------------------------     260588609       -----PFYKRTSQIAIEVCLYVIHSIGE--DI---E---------AKEGWSVFNKY---YLRLSEKRVTAEHQRWLYMVRKYWHSYGSRFYWNRDLDDYMTHM----ERERI------FTIEEG----HI--RIN-----PSCQVSVERFL-LFRNFLNSLPDEEEI--KKEVYCE--------KIFYRN---QE------AFELNVE-------------------SSSDELATLPAYREKKIYTIRQDKDWNMILAEMGGI---------------------FSHRPSIQLEILDG----DE---------LKLDGL-RHVVGTLGSGKST-LKQALIYDAVKN-----EH------LKIAVVENSISKLLELWML----L-KQC--GI-----------QAIPFISTS--SEEIYLREYLA-----ACRGIREIKNSD---EIAILSGNCILKAVA-----------------GDETEKYP----CNRLLNE---------------------------------------------------QSVPVVCPFFGACGHQFRIRELIDADIILITPQAIARSKVEKPIDNYN------------RSMYELLYDLMDLIIVDEADDIQQDFERSLMVTEHINREQ---ESILVEIE-KLYKKVEGCSVI-------K-----NDLYNFKQDFERMKSHLTLMERIFIK---------------YHDII--EHY-NNKNILIN--VLRDKVLKGF---SYEKVLIDGKE-TSF--------------------------------EKLLLEYVKLT--EGM-YGITEEQLSNEIKT-------MLDFLFDFHWTGSYA--------EKKIEENSLALLKRYGVTYASDIK----------------------------------------------------NKELQFQRYILLMLLVPFDVMLKRLVR--LYSSVYFEME--GYSKSVRMFAGIR--EQMRHLVTEPCFGM-LLGYKL----------EFKDHLLYIDALRYVGVGREMLLNWSG-AKEELGKK---GPGILCLSGTSLAP--------------------KSA-----HYHIAKKPDAILY--GKPE------GKIYMKFKPLA------------DGKSYIRISGTN-ERQRKENLKKMIDKLA----------DTLRACLSKS------------------QYRKIMIICGSYQEAEEVCAKLRYH----------NFNAYFVNED--------GAEKKNY------------MVYRKDIERFP-VLSNYADILVVPLIVISRGFNIL---DE-----------DG-N---SYFGTEFYLARPYMVPGNYANEIQMIHYQLDKIIDA---------------VKKENKDYQNRMLAFRKACFAKFIHVT---NITY--WKSLNEE--D----REIMTWFILI-------QMKQAIGRLQR----NGNESFVFLCDSAFCDGLQKQGTE----------------------LSPSTSTIHSMEFLLESIM-------------------EDKATKILYQNFYEAICEMNREAERIYCNLQEEDE-----------------------     331082038       -----PFYKRTSQIAIEVCLYVIHSIGE--DI---E---------AKEGWSVFNKY---YLRLSEKRVTAEHQRWLYMVRKYWHSYGSRFYWNRDLDDYMTHM----ERERI------FTIEEG----HI--RIN-----PSCQVSVERFL-LFRNFLNSLPDEEEI--KKEVYCE--------KIFYRN---QE------AFELNVE-------------------SSSDELATLPAYREKKIYTIRQDKDWNMILAEMGGI---------------------FSHRPSIQLEILDG----DE---------LKLDGL-RHVVGTLGSGKST-LKQALIYDAVKN-----EH------LKIAVVENSISKLLELWML----L-KQC--GI-----------QAIPFISTS--SEEIYLREYLA-----ACRGIREIKNSD---EIAILSGNCILKAVA-----------------GDETEKYP----CNRLLNE---------------------------------------------------QSVPVVCSFFGACGHQFRIRELIDADIILITPQAIARSKVEKPIDNYN------------RSMYELLYDLMDLIIVDEADDIQQDFERSLMVTEHINREQ---ESILVEIE-KLYKKVEGCSVI-------K-----NDLYNFKQDFERMKSHLTLMERIFIK---------------YHDII--EHY-NNKNILIN--VLRDKVLKGF---SYEKVLIDGKE-TSF--------------------------------EKLLLEYVKLT--EGM-YGITEEQLSNEIKT-------MLDFLFDFHWTGSYA--------EKKIEENSLALLKRYGVTYASDIK----------------------------------------------------NKELQFQRYILLMLLVPFDVMLKRLVR--LYSSVYFEME--GYSKSVRMFAGIR--EQMRHLVTEPCFGM-LLGYKL----------EFKDHLLYIDALRYVGVGREMLLNWSG-AKEELGKK---GPGILCLSGTSLAP--------------------KSA-----HYHIAKKPDAILY--GKPE------GKIYMKFKPL----A--------DGKSYIRISGTN-ERQRKENLKKMIDKLA----------DTLRACLSKS------------------QYRKIMIICGSYQEAEAVCAKLRYH----------NFNAYFVNED--------GAEKKNY------------MVYRKDIERFP-VLSNYADILVVPLIVISRGFNIL---DE-----------DG-N---SYFGTEFYLARPYMVPGNYANEIQMIHYQLDKIIDA---------------VKKENKDYQNRMLAFRKACFAKFIHVT---NITY--WKSLNEE--D----REIMTWFILI-------QMKQAIGRLQR----NGNESFVFLCDSAFCDGLQKQGTE----------------------LSPSTSTIHSMEFLLESIM-------------------EDKATKILYQNFYEAICEMNREAERIYCNLQEEDE-----------------------     219666712       -NRSNFRYITDLIIRVELMLTGFNLATA--HN---K---------AEEAWSLIPGY----DFW--KIAEPANFGIIGRMRILFGFYRAESLWIKDLEEYGKLD----AAYRL------YCLMDN----GL--YKE-----VPPRYVSDRKS-EYYNILMNPI-PHKR--HSLPFITAGV--FNYQVFIDE---KR------NVQGNIP-------------------VIQTEQVVFPKQRSKKSLTCDFNRDWLGLPEEMEHI-----------------QKGWLKRANSFELASLKQ-----------SMC-LNYRGV-NHLAGGLGTGKST-FRVMETYRLVKK-----HQ------AKVGMLEGTVAEVVKRVKE----L-NSL--GI-----------NAVPLIGPS--RRKKHLDTYLFSHAK-EVDDLSGWINEEHR-ALSHLSGICFIQALS-------------EN--FEDDKSFP----CQKIVQD----------------------------------------------------NKPAQCSFAASCGIYADFRKAIEADVWVTTSAAVLKTRLPGMLDPYE------------RTVFEVMYDLLDVVFVDEADYVQEQFDRTFLEEYELFGAP---NHLLEQLESELTAKINGRYSD---VAG-N-----PIILNFRRQLKEMIDIVWQIFELLNN----------------SPRL--RKH-LAYRKVFHIYALAHDLTDKL---------AKGSE-SS---------------------------------DKIWAELRGYI--SDP-FAEGK----------------FTAVANQLLALNRT---------KFKEELLEPFVASFFEGVKSKLDR------------------------------------------------------SLLSLQLELFLYLGRFEDHLKSILQ--YADIVFEELN--ISSSLGKVFNMRK---EFSPFMKEGMTGM-MVGYRY---------FAKESSLGTFKFFEYSGVGRLLLKEWSDLYDQTDGIQ---GPGVVFLSGTSVAP--------------------GSR-----HYELNSPVDWLMK-------TRTTMGKIEQFYHPLADP----------ETGELIFVSGTN-PEKRGEHLEKEVRLLK----------PLIKREIEHW-----------------YKERKILLVVNSYDDLERVAGVL-SLDPEW---K--DNFKILSRGD--------QAEEEAEGLSF--------YFPLANIEQFV---HESAEILVAPLLAMNRGHNIL---DD-----------RQ-G---ALFGTVFFLIRPYPVPDNLGYMIQALHAQLPNILET---------------IEQKSLVGGKAIKELRKNSILLLETMV---RKPDF-WSILSPK--E----RKALAWFILV-------PVWQMIGRLLR----GGRDARVFYIDAKFGMEGTTGTGT-------------------------IPSLLSYWRTMLEAH-------------------QGDKVIQSLYGSFLDSLPRITK-------------------------------------     118476563       -EFNVEKEEALQIIKTELLIYGGKTADH--QV---P---------IEEAWALLVGYNEP------VLEKYTDKSIVMNLRLLLAEFSNERIWRDNLASYLQIE----KKYRL------FHEENG----EI--IQ------VIPQIVTYRTK-VYAKLILDVL-EQEE--KDYEFAVEGD----FSYSRTV---ET---MLHKYKGSIP-------------------KIKVKYPMLPKYREKIKCSSDLNTNWVNTATEMELI----------------SKKSYVDRAKRIKFQSLHV--------YQQNT--FDYHET-QHIAGGLAAGKST-WMMLETYHQVKE----KG-------AKVGFIENSVFQVLDRVQE----L-RDL--GI-----------KAVPIIGKG--SRANHEKRFLESYVD-PSKDVSQFLTQTFH-SLASISDSCTLKALA-------------ND--FERNNYYP----CKSIKQG----------------------------------------------------DKTVLCPLANTCGVYKEWTELIDADVWVATTASLITSSIPAVIDPLE------------RTIYEAMYDLLDIIFVDEADAVQKQFDEQFTVEIDAFGNN---NSFFEKSLYAMSNNITGRYT----EFAND-----KLIQRWQLNSRELEKAVWSLYAKLSH----------------TPKI--RN--SLKNKLLFPNKLANELSKKV---------TKDEE-EQ---------------------------------RKIEKQLRKFA--VDP-RK-------------------TVQIKRKLDQLLAKE--------PNKNQILQE-VAKLFKMDNQITRD--------------------------------------------------------AVDLLEFYLYFSYLDYNIKFLLS--YYPAVQSRLG--IGYDVAPLLTKAK---RYKPFLLDAMTGK-LFGYRY--------EQTEDDKFGNFKILEYSGIGRKFLYDWSNMYEIAFKKK---GPAVVLLSGTSLAP--------------------GSD-----HYDIAMKPKWLIQ-------SELPPSKIKQSYIPTFDE----------KDGELLCISGKR-EQKRAENLSKLAASLE----------DRLLVELETL-------Q---------VENRRILLVVNSYEDAKTVANTLEMIPRLS------KQYRVLTREN--------DKLKN--------------AFPRSQIEMFR---KAEQKILIVPLMSVGRGFNIL---DG-----------GT-G---ALFGSVFFLVRPYPVPNDLNYMIQVLHAAFPVFMNQ---------------IESRGLHYGKAIKQLRKLSMGRFESMY---KRADF-WAVLTSK--E----REVLSWFIFI-------PVWQMIGRLLR----GGRNARVYYCDGSFHNKN-----------------------------SNVPSLLEFWRMKMQKY-------------------KDDETFMALYGPFVTSIENMWKVED----------------------------------     126656942       ---ENYVSQPTNFIRTELMLYALHTYFP--LH---P---------IKKAHLLMQGYREP-------GISDYQWQIIKHLRHLASEFRSSISWEIALRNYDELTKNN-NSILG------FKIDYD---HNH--IR------LTNDMFTNRYE-SYKAVLTEKNLEFDT--KNYNPAPKGN----YQFKINS---EE------TRLVKIDEAIANIGI-----------QYNNNIPSINLSNNRESIRVKIK-ELVKKGQELKQV----------------LKYDAGEIIEKSRYWDVEN--------NKETDE-IYIDGE-SHILGPTGSGKST-LIESLITILIEQ-----N-------KRIAIATNSVGEVQDWLE-----FAQKT--NI-----------KAVPIIGNS--ERHKHLSRLNQAI---MFGNKDQSFTHP---GFKWLSQCCPLFALA---------NPSSPQSTTNKRNKKP----CFNQLEDINDT-----------------------------------------------KNKKYDCPLVGVCPQHITAKELEEAQLIVGTLPGFIHKKVSSHTLKEN------------ITILEYLALTTDLFVVDEVDLAQPKLDELFYPIVTLASFEQMQDTWSRNEFYQHINSVLEGEVVVLKKFRDS-----YLEESEGWKFLACKAIADMMYSLRDI---AAIFQGKQPTQEIETLL--TQC-ATEGRLFGAWSLFDSLAEHLSGKIKIRLGEKIRK-PTANKYEKSY-------------------------ERYREIFKRIQ--DKI-SDPSLVGLENKDSKIVTK---LTYIAGILLNTPSSK--------IPHPKCVEFIKETKWDTELNKLES----------------------------------------------------DEEKFINNLAMLLQLSIYAAQGLGALG--KHISTRKLSN--SELQSNLPLIPPV---DFEKLLPASPVGA-VTSAQY--------------QDGHLKIHRGICIGRSLLSQWQE-IFTVDGLT---PSHLLVTSATSYSG--------------------KEK--QSYGFHVQQKPSLLIE--TPKEKIEKVTQKSEFFFCPVV---D--------NSNIPVRISGFY-GEQRHDNIGKMVSGLC----RSFTQGEPLIDRFQTY--LKDKIG---------EDRKNILLITNSYAEAKTFYTCLKPPYQ--------EKASFVVRDGDSVL----WSPDI--------------NVPRSKMTEFP---SQGKELLIAPIGAISRAVNLM---HP---------DNKE-E---PYFGGMVILVRQHPRPDDNQIIISAVNKNAVDNMGS-----------------KPVTTIQREARNTRDV-------FL---AVPQI-FSNLPDE-IQGVAMRNPLVWTLAV-------NLTQLIGRSTR----GGRNTVV---------------------------------------------------------------------------------------------------------------------------------------     307153446       ---DSVPMEFSSFARIELMLYALQAYFP--EQ---P---------IRQAHLLMQGYREP-------NITDFQWGILQHLHHLTPEFRSSISWEIALRAYDDIANDGASSCLG------FEINYA----TN--QIT-----LTHTMVRERYE-IYESLLIKEPLKFVK--QKYKPAPVGE----YEFNINP---EQ------VRLIRIE---PEIANIGI--------QYASRIPSINLSKNRQAIRVSLA-DLIQKGRELEPV----------------LGYDAGAVLEQSNYWNIQE----D----QQATE-IIIDGE-SEILGPTGSGKST-KVECLVTLLTSQ-----E-------KRVAIATNSVGEVQDWLE-----FAQKV--GI-----------KAVPIIGDS--ERHQHLSRLNQAV---MFSNRQQPFTHP---GFRWLSQSCPLYALS---------EPNIPQSGDGQHHKPP----CFGKLRNINDT------------------------------------------------DKAYDCPLAPICPRHIQADELEKAQLIVGTLPGFIHKKIARHNLEEN------------ITVFEYLALTTDLFIIDEVDLAQPKLDEIFYPIVTLESFNLTQDTWTRTESYQHVHGILKGSVVVPGKYS-D-----PYLEQSEDQRHLANRAIEILMYLLRNIGATLKGKKSSKQQIIEKIL--KDY-SREGRLFSAWTLFDNLAEQL---SGEAHLKNEKK-NTITEESREQIKRSY--------------------ERYREIFKRIQ--NNP-VRPNYTGLNDTDTRIVER---LALLSGAVLAGDLLTT-------VPHPDCKRFITETQWDIQFEQLES----------------------------------------------------DVERFSKNLAILLQLAICTAQALGALG--KHVSARGRTS--VDLDSSLPLIPPV---DFDRLLPKCPVGS-VTSTQF--------------LDGQLKIFRGIAIGRSLLSQWRS-IFSVDGLT---PSNLLVTSATSYSG--------------------DTE--QSYTFHVQLMPTLLIE--PPPEKAQAVAHDSEFFYCPVI---D--------DTNEPIFISGSQ-GDQRKENIYRMVSGLC----RSSRHGRALLDQFQDY--LAENVG---------IERKNLLLVTNSYVEAEVLYNSLQFPYK--------DKASFVVRDGQ-------WKSDILDS-----------KVARSKITEFP---TKNKELLIAPMGAISRAVNLM---NP----------TTG-E---PYFGGIVIVVRQHPSPDDNQLVTSGVNKETIDLIGQ-----------------HSVETIQEHARHVRDV-------FL---AVPQI-FSKLPEQKIAGVAFKKPLVWTLAV-------NLTQLIGRSTR----GGRKTVIWFVDAAFMPETAKGNPS---------------------ADSEQNSLLIAVRKLLREAIN--------------KGGVSGRIIQTLYGPVYYPLTRLTHFIDGRNL------------------------------     359458562       ---GIGLPKVSVFIRTELLLYALLTYFP--HQ---P---------IEKAHWLLQGYREP-------GITNDQWHILTHLRHLASEFQSSISWELALRAYDELAESPISSWLG------FDIDYD----AN--TIT-----PTRNRVLERYT-TYERVLTEDPLHFLT--QEYKPAPVGE----YTFNLNP---KL------QRNIRIP---ATLASIGA--------SYVSSVPNIDTSSSCDSIQVELT-DLFAQGTALQSQ----------------IGYDAGAVLQQSVYRDIQN----D----EIATA-ICINGQ-AHILGPTGSGKST-LIDCLVALLIER-----G-------KRVAIATNSVGEVQDWLE-----FAQKI--GI-----------RAVPVIGES--ERHKHLSRLNQAV---MFSNSQQPFTHP---GFRWLSQACPLYALA---------DPSIPQPAEGQRSRPP----CFQKLQDKAEK--------------------------------------------------KFDCPLVPICPRHINADELKEAQLIVGTLPGFIHKRVASHDLAEN------------LTMLEYLALTTDLFIIDEVDLAQPKLDEIFYPTVALASFKTV-DTWTRTEAHQHVTGPLEGEVVVPGVLN-D-----PYLEYSEDQRHLANRSIGALMYLIRN--IAESLKGKSSKQAIERLL--QAY-TREGRIFSAWTLFDKLAKHL---SGLAHVERSVH-KVRQQTVNKRERSY---------------------ERYRELFKRVQ--DNL-VQPDLTGLESADRRLVES---LARVSGVLLAGDLLTA-------VPHPYCEDFILTTRWDTDLATLEP-------------------------------------------------TSTDSDCFIINLATLLQLAIYSAHVLGALG--KHISARQRAD--LSLESTLPITPPR---DFNGLLPNSPVGS-VTSAQF--------------TQGHLNIFRGIAVGRALLSQWYS-IFNVDGMT---PANLLLTSATSYSG--------------------TQA--QSYPFHVQLPPTLLIE--PPSRKRKAVAQDSEFFYCPVS---D--------DAGNPVFISGSQ-GERRTENIGHMVSGLC----RSPKHSKALIDLFQDY--LETSFG---------ADRKNLLLVTNSYAEAETFYNVLKHPYQ--------DKASYVVADGQ-------WKSHD--------------QVARSKLTEFP---DGGKELLIAPMGAISRAVNLM---HP----------NSG-E---PYFGGIVIVVRQHPRPDDNQVVVSAVNKETIDIMGR-----------------RAVEVTQRRARQVRDV-------VL---EVPQI-FSNLPDS-IADIALKDPLVWTLAV-------NLTQLIGRSTR----GGRPTVIWFTDAAFMPNTAKGDPA---------------------SDTEKNSVIRAVRTLLGDAIS--------------KGGESSPIIETLYGPIYHPLNRLTHFVTGVKL------------------------------     17229466        --EGLERIQAELLLQVELCFVLMERLGL-DDE---P---------VTAPWAVLSGM--PLRHPRLQNLDEMARKAIANARQIA-PFSARFAWLAALRSYLKIP----LDWRN------YGDFTP----QN--WDIYIIHAAKNLRHPANQD-VYERCLIANL-NFRL--RKVQQVELDV----AYQFEAK---TG------EQTVIVPIKFTQQQVRN---------AQIQHLPWFATSRSRSSFSLRIS-DLEQDAAWIDERE---EALTRQYGWDETAKGHWVNRFRKINFHRVQE----NATLLQQEERILELDGF-TNIAGMVASGKTT-FSQLLTVNIVRH----HGD------RRITLVVSDVQSAIKLANQINWWFCDDP--ENDEP--------IAVPILGRS--KRDAHLRSFSTS----KDYLEHQQRGQPHW-GERWLGTACPLQGQI---NKRSFQEFLNNK--PLKPGTEP----CYSLQKMPASD---------------------------------VYDGLRLRARRSKSRGSFYLCPFFAKCPSQQVYHDMPNARVWITTPGAMAMAGLPRHLELRP------------MKIGELVYEQSDFVVFDEVETVIKWFDDTYAEEVVLTDGGK--NGVFDDIGVKTEQFSTTNRVM-------P-----PLTQRWTGAERDAQKAITATLTLLDK-------------HFGHQIL--RDW-IKRG-YFTPNTLLFKFARRL---AGLEEFESPET-PEAHSQANARLI-----------------------KPIVRYFDALLNEEDP-LRMDSPENPSQNPVYR-----LAALMQEINSTGESA-----LDDKIYIACRAW-ILEFFPNTQRRLER----------------LRTELENRQHSSPQSSNEN--------------EVDTIETLAYRLQFALTIALLDRHTRIVFY--EWQNRPQSIT------DDSPHQRMP--TAMLNILPLPLTGR-QFGTYYSKDNES-DKKSKNRSNNALTLFAYTNIGRCYILNFHHLLTDFNGQR---GPNVLALSGTSYLP--------------------HST-----SFHVGNPQGILMP----EQEAIKAIAQSYFKFLPQF---N--------QKNQPLRISGSA-ERKKMGLFQEIARSLI-----GSNGTGHLGQELKELRHLGDDAN------YHWRDRDRILLLVNSYDQARWVAKEIGNCWSSM---Q--DSVYHLVPDNTDT-----YTENDFDEIDRLLQPTDKGALNRADIETFG---QTNGRILVAPMSAMGRGFNIL---NG-----------NG-K---AAFGSVYFLTRPYPHPHDTQAIAQEMNRRALDWVDK---------DAFTAW--LQGDGVVQRAEKVRQLAARYWRSV----EQRSY-YKTLRDD-------KELLAYPRFDLAATTAGLVIQAVGRLLR----GGVPFRAYFVDAAWAPKSAARIAN----------------PELNENDTEQTSLLVAMILRICDYAS-----------------EENSVGNALYQPLAEAMETINDLYF----------------------------------     220930099       ------------------------------------------------------------------------------------------------MDYLD------KSIKK----------------------------------------IYLKV------------QYIEYVKSKE-----------------------RSINID----------------------------VTPKKCEKIVIRKD-ELDRAAKDMDEQD-----------KKKGKSYNWEKRVRDLVFTIPQQ-----NLTIKDSDE-LTVDGI-KHMVGALSVGKST-FIKICSYLLAKK-----G-------KRVTLFINTITEVLQTVDY----F-NDI--GI-----------RAVPLLSPN--QIKDHSNQYLTTL-----KSRHELFDKKP--SFRYLSDSCLLVNSN----------MTIEG--VMQDREKP----CTSLMSGE----------------------------------------------------KKVYCPFIYSCPRYNNVKDLNDAQIVITTINSAVQSYLPAPFCDKK------------ITILEYLIRTCDLAFIDESDRVQANLDTLFSTTIHLYGSE---DLFYEKIMERQMDYFKKGTLPDSPLLI-DFIKDTISLENYITGIIEFFKNTDRPGKLFSK-------------DIFGKVI--KSSMMWEELFFEAIGVSTKDLKDL---------PSGDP-LKQQVTKAR--------------------------ESFRESYWEFQ--SEH-IKGLMEYSSKSQMNYL-----ARAITSNIVDEAKLR--------TDIQRFTKLPLKNFFKHKRSDIEK---------------------YDKLEKLKSNLDRP--------------LSDVVHRAEEKVRFILQIYMLEYKLKCLLQ--SWTAVKNIDT---DFIGQDNKLPGILKEEFAGIVPALPVDV-NYGFRI----------KEDNNSFAIDYYYYEGVGRWILLNFDK-LYRDLDGV---GINCILLSGTSNLE--------------------YSP-----KYHVDIPVSCLLR------KKDANRPRLEMSFPAHI----------------AVKVSGTY-RENRIDALQKAAYNIS-AKEINPNGTSFLDSIIETL-------E---------PGRKRILFTLGSYENCKEFSKALNSFG---------YSARSLVRPG--------SADANDKN-----------VLERSNIEDVA---KLNIKHLSIPLG-IGRGYNII---TESLEMIDESLAVQG-KKTVAAMGAVFFIARPYYMPDDANTLLSWLNSVYVSEIKQ-----------FKG---SRKNTFEGFIRQLIKRLNATQRSY----ENMFG-YTSLDSW--Q----RNRLLGDTLV-------DVYQLCCRLIR----GDVNAKIIFLDGSFAPNTFSCGKK---------------------SDSPETSMLIGWRELLKKMIQS------------ESSIADKEINKELYSILLDGLSELKLVEGDVINV-----------------------------     290956756       AEAIKPFLSAREFFRVELGLFFLSEYAP--GQ---P---------ATLMRKLLDGY---------ALPGAGTETVVRNVRRRMGPTARGGQWRLRLNDYCKVP----AHLRL------FELDDG---AGTRVVHGSVLRRRGSSTLPEREQ-VYRDAMSVPVAYKVE--AGHDPASPGM---RVRLKRAD---GS------IVRFRIPDWLEE--------------AAPERRLEPKTHRIRRPFPQLTEDDFHQAAKEMDQLL---------RTSERYRTENFTGRVERMVFSRTDS----EAGCLTNGLGIFTIDGL-AQVVGLMNSGKTT-FNDVLVKICTDR-----G-------LRVGYLVSSVGNALEKVRF----F-RSL--GI-----------DAVPLIGHR--NRPAHVARYWDDLLYVPDNGQAPPLPDGNDEVAAFATDICLLDSLL---------EPSGPIAAPLSVDERP----CRDKLRVDTSG----------------------------------------------KRAPIVDCPLLAVCPVQAAVRRIPTAQVWVATPAALLASRAEPASYKTQ--------------WVIPAQHELDLLVADEADQVMTQFDKAFMHHEPLTSP----DGWSSRIALAWHEGLARTWYR---PMA-D-----RQGRRYQQYATYHAEALAGLLPLLMR----PSAEGQEEARGSGSEL--LEE-VTADGPFSGHTLLMQLARAL---HGITSRVEEQQ-QSHLWERAE--------------------------EYFHTHFARLV--EDP-FGSPPAG--------------LKPLLDTMTSGYDTEM-------SAEEVAQDWLTHHIPEDMPDPGDR-----------------------------------------------------LGELARVLVAGCWSARVTTTVFELSH--MQESLRALMA--VEDTNNLLAHQPR--PELLAVVPEQPMGN-MMALQW--------TPSPKEGRGSLDLLWLRGVGRWLLYHLHD-LLACEGVE---GPHVLLSSATSYNP--------------------LSA-----RYHIDVLPTLILH---EPPDCAAAIRESRFYTRPRRRP----------GHERGIYVSGAGGRGARQAAVRAMTDAVC--TPDPGAALSLLEQVLQGC-------D---------PERRRALFVVLSTEDAATSAHYMNTRTA--------VRAVHVVPDR-----------RAPGLY----------GLNHRRISAFP---RTDAQVMAAAEGSAGRGHNML---ND-----------SG-V---AAIQAIFYLARLHPPPTDLSFPLAILNAQAMQRLLK------------PVLCDTPGTDAAGEMRKLAYGARATWGTMM---GRPLN-FRAMKDD-YL----RHAFIADQSA-------SLYQTTGRGLR----GNVPVQVYLLDAAFAPRAADPRDT--------------------APDTERTSVLIACRELTRRMLADPG----------PTADSHVRLNHQIYTATWGLLGHLLDTIDWG--------------------------------     372487593       ------------------------------MI---P--------------SLLQGH-------LAATLPDEMSRALFVLRQYAGNLCSRLAWRKALSSHAEAR----SRTA-------YIVFAD---DEV--QQR-------EAVAPDLVT-LLRDSLKSPL-PYSQ--RELRFAEPGH----AKVELRQ---GQ-----EVRTFKIPHFQ----------------VPPPNRNSLPKRSVNPPISIKWE-RLLSIAREVDARE------------AAPGFPEWMSRL-NLHSRLSKVQIESLSSDFFQNGT-IILDGT-SHVVGMLSSGKST-LLQALIFALASP---DYG-------KRVVVLFPDTASASQLVAR----L-HAH--GYE----------QATVISSPR--NRDEHLSMAHWNA---QGYPSDAMLEATAA-MTRSLGVACPLEGFQ----APPQFSDGTSSRTVLRLSEKP----CHRLKQLGKNR-----------------------------------------------TEVDRSCPLIGECPLHEQQSKLGTAKVIAMTPQALLHMTADKAFVAEE------------MSFPELFQFIADVVLIDEADSVQATFDSECTQEKDLLSPNES-AFMISNMRTVAKSLSDKTGRQ---YLA-------ASNVRWHRELNRLQDSISAIYHLLLK----------------RGEN--LSW-FTNCKTFTAASILADLVPSGESTGKDRESSARER-SKTLEQIA---------------------------IIAGMLYGKAAASDDKEAGDDLTTGTLSVNMQAAHAF-LNRLLPAIIDAVIEDDPEGVIH-QIAEAIDSGPLSVFAYRGPALSKQ------------------------RRSDSPFAPPI--------------LPTDSLSRAYAIALALLTNICLSSFAYLVR--NQAAVEDDFG-ISDEDAFREARRLL--RHYGNLIPRPLFGT-VFGLMF-------SPAGQSAQGGTLKLVNHLGVGRYLLTQFHR-LLAHEGQA---GPHVMLMSGTSWAG--------------------GCSTTASPTFDVQQPVSAILT--QPPGELAAL-SHSRYEFVSL--------------GPESIVVSGSA-PDERRENLRRVAHLLG----RSSATGTRLMNKWHDL------DTLWPEERERTMHRRRALLVTNNYGDAKIVANELARVAGQA------HSVYCLVSDQLARA----GELEQDATRSGEEFHRNIVPLPRSRVEDFG--QSPPFSILVAPLKPISRGHNIV---TD-----------SG-Y---AAISTIYFLHRPHPRPDDHSSVIGMLNRLAMSVLQE--------DGVFP----KSGRSLDSVNRRFVSLAHRALNEGF---AMRVA-YSVMSDE--A----RTQYSWDLMT-------SLWQTIGRGIR----GGVPIYVGFIDKKFAPGIFGNPPK---------------------RDTANSSCLKQCQETLQLAIN---------------DDVNKVIAERLYRPFLDALDRLFGDEQGAEI------------------------------     381168804       ---------------------------------------------------------------------------MKAIARQVDEKEAQRDWPSS---------------------------------------------LPPLHLAGRLE------------------KTLKLEGLAP------------------------------------------------------------------------------------------------------------------------------GFFDGET-LTLEGA-NHLVGMLSSGKST-LVMGLIFALAKG-----GTG-----KRIAIIVTDTIQGATLAAR----L-RKH--DV-----------KATVVSSLY--NRERHLNSIHWQQ---GLSSTGWALSSLGD-ISQNFGVACPLDGLQNSDPQVIRGGAGDVG--FPSFKEKQ----CHRIYQKVPDD---------------------------------EVEGGDGATSELVDDGRARSCPLWAACPAQDQQRAAVDAQVLIMTPQAFVHMTPDKWTTDHH------------LTIPELLQYVADLVIIDEADAVQKSLDDAFAPRSQIMGDER--DVYAPSISTRSSEKLREKSGL---QFS-K-----ATNARWQNNFYTFFRLVGDIYAMLQN----------------EQAS--LSK-VYQNAPFTAASLLYELWRKK-------IDESGAS-RTDEEVETEFLQLIRVASA----------------ISKFSPMSGVA--EDE-EGSRGERLEFDDPRFLTAADALQELARQVLFADYYA--------DILPMAETMLTERLEPFCLLSEED------------------------IRLLSGNGADK--------------ATRDVVLVKRRSNTLVVLLAVVAELALAHYNWLVKAQPAVADDFGIGDAELLTQANNLIRHYRSLLPSNPAGS-IFGLLYDEP----SKERANLLGGKLTMINHLGVGRYLLTHLHD-LLKGEGQA---GPNVLMLSGTSWAGGSARRYDPEVKRPIDS----ASP-----TFDVQVPVKGVLV---QPEAELEAIKKSKFALVNIR---N--------GDGNQVVISGAN-EKERRQNLAFIAEKFA----ARQDDSNLFERQWRRLEQAWGADD--------LADRRRAMLVVNSYADAAGVADALMQALETNGFAD--WKVFCLVRDRGDDANQ--TVGTGPRLAR---------PLPRSLVEKFG--EEGEKTILVAPMQIVARGHNIL---NS-----------HG-K---AAISAIYFLHRPHPRPDDLGPIIGRLNRFALERF-D--------KGLKPV---DRPETLGARARRMRYAATNIVRYSL---DFRGG-YKNLPGE--F----KAQFAWDMLT-------PLWQTIGRGXR----GGCPVFIGFVDYKFAPLSFDWKDN-------------------APIDNGKTSALVQAIHQLKLAMDP------------ESNPNEHRVARLLYEPFYRALCQTEGLKHG---------------------------------     403070935       -NDDESEVYAKLIVEVEVAFYVSTQLLPSDNK---Q---------RSQLEYLYSGTHLPQFQQ----FTPTQMELWMKVRVLLPAWKARTIASSKIEDYIEIP----VPSNL------CHLSFE---ENT--IEW-----KALIQYPERTN-LYKDLLSMPI-PYLE--NKKGFASPKS----EVSYFDK---EL----FKSEYYTIPEHMKPF-------------PTFNEGVFLTPSKKGKDMTISKS-DLLQSAERMDMI----------------INQDWYERIKELNFHEVVG------ETLIKSER-LNISGT-QHWVGTLSAGKST-IMDVIAFHQSQQ-----K-------KVTVLVVGDVATALQKVDL----F-HSL--GV-----------KTVPILSHR--QRKDHIKQHFQSI----NENVKSLSTLKKR-SFRYLSDTCIIKTSQ--------------E--KLTDTAPP----CFSLKE----------------------------------------------------KGKTKVCPYFKQCSYHNAYHDLPNAQIIVATIQGLVLSELPPILLGIR------------MRMLEYVSKSSDLILVDESDRVQSQLDSIFAPSVEMAGTE---DSWLEKLQNKAFQSFVRSKV----PLS-K-----RRVERWFIALQNTVTASYIAFGQLQD----------------QDIL--K---MIGKQYFTGYKLIKMFLLNAFGVTPENEEKSLKS-KEFQKLS----------------------------EQLESFIHSYR--KDE-YAHEQNNASNEELKQK-----LSGVIDSITQDNDDI---------------RFLLDEIISITQPIAKF-------------------------------------------------PDHKRSLLQKQLKFSLSILFIEKNLFTLIQ--LLPSVRDELKGLVEDEVMSIFTGTP--EDYDGLIPISPTGI-WMGFRYE------SDQQSNAQNGKLNFMRYVGVGRYILTNLDH-LFEGIGHYK--GAPVALLSGTSSAP--------------------YSS-----KYNVQLPVSYLLE------KEDEDLPNINMSFRPLM------------KRDEFIIISGKH-GAERNIALREATRILF--------QQNILQNSLKRS-------S---------SGRERVLIVVGSYKEAKIVGEYLMDIWPDP------NEVYHLVNDE--------TKFGTKG------------EWTRQKISSFP---KVRGSILVVPLLALERGHNII---TT-----------ID-EQVVAAFETAIFLVRPYPKPFDVDRVVNRLNEFSINMLSK------QY---------NGNYDVKEGVLSLRNRAYVIQKELL---ANQVW-FRHLDEK--E----RYHLVMDMFI-------SVWQLIGRLIR----GGVSANVLLCDGSFAPETIKGNSD-----------------------SYETSMILSWKKVLSEVLD---------------TPKTSTIASKLYSPIINGIQNVEGVTTYEKKKL----------------------------     186681798       VQQRELKRLAQLIADVELGLTLLKEVAP--EE---P---------ATSVEALLKGYRFPVEQL----QSDRNWQLIQNARFYLIK-RKGRQWLRVLQEYINLP----EIIRI------YCLEEA---RNV--PQP-----IPSSTYPTRLENIYLPTLLKTP-QHRQ--RKVKLADEGR----WYAKISQ---KG------NSPVEVPIDIPKEVANIA--------PSSVVSLCRTRTKQNPQCSVTRE-ELILAAQEMDDKL-----------SQFGQQENYRNRLENILFQLYDA----ESDNFQQGNQ-LTLEGL-IHIVGLLNVGKST-LLEIMIYFFAKQ-----G-------DRCGLMVNDVATAVRLASL----FCHKL--GI-----------PAAPVLGSK--RQEQLAKVYEPIL---KSEGKEITKGGMHP-AWRWFSRVCPLLALV-----------QSED--KWEFGNEP----CHKLYQKVLVA-----------------------------------DNDDSDDEDWQESDEKYTCPCYYKCPRHHLEDDIATASVWIFTPASFIHTRVPRQGFEQD------------LTFAEAVYRECKYLFVDEADRVQIQFDEEFAPDEVLVDGSG--NSFLNKLGLNLATIYNSDRGD---MAG-------DRFLGWTSAHYHTQNATNRIYHLLLT----------------HSKL--VAW-LGPL-PFTGRSLFARIIRDLVDPPEIPVSAKPKL-THQQIMEERRKRIIEADLAPTEQRRRR--------KQMMDQLDGFL--QHP-LNPRRGGE-------------LSGLALTILTAEDDR--------QALAEIAPW--CKRWIEIHNLSLP-------------------------------------------------DETQFEKLTRNLQFAILVTILDNRLGFIVD--NLSDLGRVIN--LHDSSQDLLHRPP--DDFLPVLPESPVGN-ILGFLY-------KQERSAKQAGKLDYFRYVGVGRALLLNFPK-LFAVDDWQ---GPHTVLISGTSYAP--------------------GSP-----AYHINIKPTILLQ----SRTGEAGIAESQFFFSPKQ---N--------PQANYIALSGLP-PAKRKLAAKEMVEAMC---YSVRGGESFLDGVFEDLEQRKQQQT------EWWSDRDRILIVVGSYDESEWVTSILQSR----------YRFDVNINDDGIATL---RRDNAPTHLH---------GILRSEIRNLQ---HLPTQIVVAPLMALERGHNIL---NA-----------QG-K---AAFGAVLFLNRPMPVPDNWQSTVQQLNAWALKHEKD------STLYEEAQSI-SGNLTLTQIADIFYQNAVAEMVNLN---YTAWA-FKQLTQG--E----RSVLCWTQLV-------SIWQIIGRLVR----GGVPAVVHFIDVKFAPNSAIGEQD-----------------------SEITSLLVAIIKVLEPYVE-----------------GEDVLARSLYGAFLNALKQMRERNLNYD-------------------------------     172039224       ----------TGLLNVELGLYLLESLLP--NA---T---------AKSLWVLLTGY--DYSFTEKQNWTQEQRQMLSIARHLLAQYASPKLWSDTLDRYQEYP----EETRG------YEITEL---GTF--ERR-----TNITVANNRFE-VYERSLNTPVVLSQR--KEVSWAREGQ----YKCEVEK---RMDTVTITSELANFP-------------------RPPSHNLN--NNSQRETLNIPWR-DLHSTAEWMDEQ-----------RIEKGLNPIWINCFERMKLEVFN-----DSEELVEADY-LRLDCI-KHLGGIPSAGKSV-LMKVLTVYAYRR-----G-------LKVTLIVADVLQIFDLIKT----F-TEV--NIN----------DVAPILGNS--NKASHLSRLHKAV---YNANPNEPYNQNHP-GFKYLSSACLLTPYI---------TPRLEK--AFEIGKQP----CFSLEPIESEE--------------------------------------------SEEFTNNKYCPAYGVCPSHQKERDLVKASIWIATPGSLIYSKVPRTINREN------------IIYFELMARRSDLVIIDEVDQHQAYLDSAFSPNQTLRRPTR--DAWIDELHDLVETKLKYTQSK---LLH-K-----DFIADWWDALQEARQIADTIYGLLTE----------------ENRINLGKW-RSYKRYFTDWLLLNEVATLLTVRNKQEPQPEEIN-NRQ--------------------------------WFMQYVFQPYI--NSK-LEPDVKAEELDQR--------DKQLLLQLTLIANKK--------KKKLAIRKW-INEVATIQLSKEEK------------------------------------------------------NKVIATLEFALLVCTLQRNLYLVTS--RWQFVRGILN--LNMSDSMWFEFPP--LDFNPLIPNMPMGN-QLAFQY--------LKSYKEPLGSLQVFRCTGVGRWLFTNFAN-LFQGDNFK---PPYLLMMSGTSWAG--------------------ESS-----AFHVDVPVSGIIS--PREEKE----IKIDSKFLPFY---D--------DQQKPISVSGT--GDKKDYNLKKIVSKLV--------EKNYLEEKLQQL------------------NGRKILLLVNSYLQVDTVYEHLKDL--GW---E--DRVIPLSRDDENINE---WEDIKDK------------SLQRGQSKEFA---TGKEEILIAPLKSIERGHNIV---NV-----------DG-K---AAIGAAYFLVLPHPSPDDLSYAIHSINRWAIDNYKK-----------------VSGETLDELGDNFRKNAYAKWRHWL---QLSIR-LRTLPQD--EQYSDRNAVYWDIIV-------CLWQVIGRLIR----GNANAEVFWCDAKFAPNTAKNGDE---------------------SDNVATSTLVGIAHLLHPYFQEDS----------DIPQQEKLIVQRLYKPFYKAIIQTKGVSGLPIVNI----------------------------     309791272       -AYGRIPMPAADLIETELILTLLTEYLP--GE---D---------PEQAWAIMNGYPFP----ARAALSDAARKAITRSRLYLRAPLGRSTWTRWLAQYRQLP----APYPI------YSLHNG-----A--IVT-----QLTTLAPERQA-LLQAALGTPP-PWEV--ERPRFAPAGV----YRFMVNR---EP-------YEVEVDGRSAAL-------------AWRYVANKLTPQRERAPINVTLN-ELVATSRWMDRSL---------------RSDRWERIMREIQLRLVTA----D--GFAETDT-LCLDGL-HHMIGMLGSGKST-LLTVLSTHLARK-----G-------LRVVLVYGDVATLLRELDT----Y-ERL--RRDDDKV------VAVPLIGRS--TRLAHLNRLHAAE---RDRNEIGLQLAHP--GFTALSTLCPLDGLR-------------QDARPIPTGQEP----CTTLAEPPAGE--------------------------------------------EGSEPRRYDCPFLPVCPVHQTSQALLDAKIWLATPASLLASSPQRPFIQER------------MRFIELVMWSADVVLVDEADMVQVQFDDHFAQTEVLIGRS---DSWLDRLYTQVARQVYRPGRP---LVGRS-----RDFDQWLTAQNNAQRAADCLLRLVRD----------------DDRL--RHW-LRTT-YFNGRRLLQRIAYYL---------EQTHS-CDT--------------------------------ARFLEATAQIA--DRP-MGSARRGQQAPLSAA------WVEIIQLELLGSERE--------EVLDLVIDS-LCTLVPETRRLNRQ----------------------------------------------------VRDTIATQLLTALMVTVLDQSLHSLIA--AWPSAEEVLD--LDKGGGGLFYPPS--ESVTRLIPEAPTGA-ILGFQY--------YDPQDTGQGELRFFRLRGLGRALLYHMHDAFGESDGVV---GPHVLLTSGTSWAP--------------------GSW-----RYHLDKAPDYALL--PGPPDQTGQ-TRCLFNFIPDPDP-R--------AGGKFLHVSGKPAPQDRINALIAMVRALAKRSTISGVARNQFDIEFANL-------P---------AHRRRALLVVGSYEEAEYVEHALADALCVDAG----EAVVALIPDTDGD-----LQLRRPHA-----------KLRRSNLARLP--EMEGIQFLIAPLQAIERGHNIL----V-----------GQ-E---AAIGSIYFLTRPMPVPGDLNVAIQKLNAWAMRAAPT-----------------CEAATIGEAGVWLRNEADKRWRDASPANDRQGT-YRELDDV--E----RSGLLWTQLV-------LVWQCIGRLLR----GGVPARVHFVDAKWAEVRTGLMPG--------------------IEETEASSMLVGFARLLRTAMA-------------DPDPAQAAVAQALYGSFAQALDLLLKS------------------------------------     375092907       ---TLESRTALRLVDIELVLHLVEQVAP--DQ---S---------VLDAWALLGGY--PFLTAGRGLVASEHESMIGVARHLVLWFKDREDWRAALEEYAELP----ETIRG------YEVDLD---SGT--TSR-----RVPTVLTDRFD-YYEDVLSNPP-PDLT--SKITVAEAGR------YFNQA---DM------GTSVTIPDWFPI--------------HPGGRGHDVLARCDRDAIELEWS-ELVTAARWGDNRE-------AELGWPEHLRSNWVDRLKRVTLEVRQG-----NNSFSRSEK-LTIAGI-MHMIGMVGAGKST-LVEVLLLWCHLN-----N-------KRVCVVADNVTSVLRKTVH----L-QAL--GV-----------RAAPVLGQS--NRVQHLTRLHRLT---NPRNGRIAPAGDR--MFDLTSTSCSLDGLR----------DHSAK--PWELRHSP----CAGLIPEETDD-----------------------------------------------KPKAHTCPAWHTCQRHEPSRELVDATVWVATTASLIHTPIPKQLNEER------------LRYLELAWRRSDLIIVDEADQVQAQLDSVFSPEQQLIGEDY--DAWLDEVIDRTKRTIRQAGRE---PMH-E-----PLVRHWLVNLDNANIAVDIMYSALSR-----------DGARPQPALS-RRW-LDKA-YFTEWTLSQQLAQSW---AGFGPSKTNTH-RPVDGWDDDPVY-----------------------RRLRRAFDGLI--DDP-LGAKDTDDELTQR--------MVTLTAQILRDANES--------VRLRRVGEW-LESTAKELAEQGLT------------------------------------------------LTIDDPERQAVRLEFTLAVAVLAKSLDHAMD--MFRAVEMELG--LEGTSSALFHRAP--ADYQPVVPDSPMGN-VLGFQYLDDDH--DQRRGRSKMGRLRFFRCSGIGRWVLLHLHE-LFLTDSDR---GPNVLLLSGTSWAG--------------------TSA-----RYHIDIPVTAILT--PPKKELAAV-RASRFEFFPLFAG----------DNTAPIKVSGKF-GEQRELALRQMIAELA----NPGAGKSILEQHRDSL-------P---------PSRQRLLLLVGSYAEARMVAQHLVSLRSSW---Q--DQVLHLVADDEEFTDS--WDGAN--------------GLRRGDVHTLA---RTPAWILVAPLLAVERGHNIL---ND-----------DS-Q---AAIGAAYFLIRPHPRPDDLSYVIQRVNRWATHQIRN----------KFPVVEDSDREALGRCATAFRSAGHRQWRWLL---NLKLA-YSNLPVR--E----REALAWTQLV-------TIWQVIGRLVR----GGVPAEVYFCDAAFAPNAARRSEN--------------------SSDDASLSLLIGLREVLAPYFAD------------DCADPDTYLVETLYGCLYEALAQLEGL------------------------------------     392942504       ---TRLGVPLEVLCPVELGLTLLYRLDA--GQ---P---------PTSMWTLLGGH--PFAQAAGLADRPEQCWMITCVRHRLWPLRRRESWIQALRHYREVD----SSFRI------FRVSEN---LES--FEW-----TGTTILTRRFE-IYDRALETLP-HLVD--RYLAPAGAGR----YHYFDRD---ER-------VEVEIPDDVADLVDRPG--------ASLRHDLTAHTATNGAPLCVPWA-ELEETAAWMDDL-----------GARCGGRTDWGATIHSTGLLVRDS----HGHDFVEGDKVLRLDGL-LHAVGMVGVGKST-LMKVLAVWAVRR-----DEA-----LRVTLVVSDVAEQLRTAEE----LRRFL--DDH----------VVAPVIGSS--TREQHVESLHRRL---AAAGQPKLAGHHAEDGFGDLSTACPLDALR-------------ATDSPVRLVDAP----CHRLYSVDGQS--------------------------------------------ERRRPRPSGCPLWHECPRHSAARDQVDAGVWIANMASLVMSPVSAELSGVS------------LRQLELACLRSDIIIVDEADRVMMNLDMMFAPTATLVTRGP--RSWLDTLHMHNIRELAQEGRL---QLS-D-----RNVRNWEVALSVVTGATNLLYSMLIA----------------DKQL--RDW-VGIN-YFNSWTLQAKIISELFPPDESVSATAPED-SVSVVDAPERPR-----------------------SQVEEVFDQFR--DDP-LGDNGPYNTMTDN--------LAAVTHDLLHTLNPE--------AAARRLNAV-LDELRAIATDAEPD--------------------------PATRGSGHT--------------PTAENEPLVRKLEFTLLLSALHHRLDRLTY--LWPQVEDAMR--LDSTDNELVRRPP--MDYMPLVPESPMGN-VLGFQYDVDE---QDVGGDRVTGTLRFFRCTGIGRELLFSLPSLGADTATGRK--GPHVLLLSGTSWAG--------------------IST-----RAHIVIPVGAVLR--PTPKDEEKV-LKTVFTTRFFY---D--------GTGEPISISGQP-RKVRSSMLRALVDKLG-KPGPSRMSPSPLREEILAV------RD---------PDRQRALLLVGSYDDARAVADQLNEM-PEW---S--GHVRALVADNADLTEA--VTGGADDLDRAG-------VVRRGEVARFA--TDSMAQVLVAPMLAVERGHNIL---NE-----------RD-E---AAIGVALMLTRPHPVPTDVSLAVFAVNDWASRYTRGLYRSKGSEPASLTELV-AAYPSLDAAAREFRALARSRWGRLM---TRRYA-YSALKDD--E----LKSFAWDQLV-------LLWQVIGRLVR----GGVAARVVFVDAQFAPGRAAKKAP-------------EARPSPPRCDHAQSSLLYGIREVLRPYFAPRPGDE-------VFDPADAHLARLLYEPVYLALCAMLDQLDLDGAVVDLGKM-----------------------     345850903       ---EHAGTRPALLCQVELALRLMETVAP--GH---A---------ADGAWTLLGGYGLAFARASKLPNGTTEQVALAAARHLLWPMRRGRMWQQSLDAYLELP----ERLRA------YRVPAA---GEP--AHR-----VPLKVAADRFT-TYDDALANLP-GFAT--KVLPPAEAGE----HRFMDRR---HR------RTSVTVPADLVRD-------------PFPGHPLTAERPATAGPLDVPLD-ELADVARWMDTEE----------QRRGLKAGNWEQRLAQLDLDTRTP----DGTDFEEASA-LPLDRL-THLVGMVGAGKST-LMTLLAVWAYHN-----G-------LRITLVVGDVAEQLTLTEL----F-RTL--GL-----------SASLVQGGT--TRPQHTQRLHRRL---AARGEHSLLAHTGP-VFAHLSTACPLDALR-----------ALDTSEPLRYTDAP----CGALHPARRQE-----------TAEEPAAERAVRELERARGAVDGEDATDDTDSDEEDLGTPHACPLWSVCPRHSAARDLVDALIWVANPASLVQTAVPRQLNAER------------LRYLELACLRSDIVVVDEADRVQMQLDQMFAPSATLVTKGVS-DSWLDQLQTHEIAELARQGRL---QLS-D-----QDVERWSAALDVVGSAANRLYAMLID----------------DGAM--RDW-AQID-YFSAWTLQEKLLHAWYPLTRSQAAGWPDSTNEGDVEDESALYEDEEGLGADGDYHIPDAPWAHRRTEITGFFDTFR--DDP-LGGRGPYGTPADE--------LTALAHDVLHTLDEK--------RTRRRVRAL-LDSFLVGAPGPEQR-----------------PMPAAKRGKEPAPEDVPL--------------TEEWRELNARRLEFTLILAALHQRLDRVTF--LWPQVEAALR--LDSAAHELTRRPP--LDYAPLLPEAPMGN-VLGFQYLVDERAAARNKDGHRTGTLRFFRCAGVGRELLLGLPQLGADPGLGKA--GPHVLLMSGTSWAG--------------------TST-----RAHVLAPVRAVLK--PQPKALDAI-RKTVFRTEFLY---D--------TAGQPIRLSGQD-PDQREDVLRLMIDRLA--RSRSDGNSSPLQSELARI------PD---------QRRKRALLLVGSYAEAKVAATALNEI-PRW---R--GRVRVLAADDAELEAA--VDGTAPTGSQAPGTGSGAGAVRRGDLASFA--DDPDAELLVAPLLAVERGHNILTAPQR-----------PG-EEKVAAFGTVFFLVRPHPRPDDLSLAVFAINDWATRFVRG--QLELPDEGTFSDMV-TQAGDLNAAGSAFRTTARGVWRHVL---SRPYI-YSSLSDD--E----KKSFVWDQLV-------TIWQVIGRLVR----GGVPARVVFVDAAFAPQLAAAQAP-------------FTGQGRRPRRAGDPGLLVRLRDVLAPYFAHTDKVTGTVEYGARTDPADAALVKLLYRPLYEALCAMGTSPGPRPID-----------------------------     406692972       ---EHAGTRPALLCQVELALRLMETVAP--GH---A---------AGGAWTLLGGYGLAFARASKLPTGTTEQVALAAARHLLWPMRRGRMWEQSLGAYLELP----ERLRA------YRVPAA---GEP--AHR-----VPLKVAADRFT-TYDDTLANLP-GFAT--KVLPPAEAGE----HRFMDRR---HR------RTSVTVPADLVRD-------------PFPGHPLTAERPATAGPLDVPLD-ELAAIARWMDAEE----------RRRGLKAGNWEQRLAQLDLDTRTP----DGTVFEAASA-LPLDRL-THLVGMVGAGKST-LMTLLAVWAYHK-----G-------LRITLVVGDVAEQLTLTEL----F-RTL--GL-----------SAALVQGGT--TRPQHTQRLHRRL---AARGEHSLLAHTGP-VFAHLSTACPLDALR-----------ALDTSEPLRYTDAP----CGGLHPARRQE-----------TTEEPAAERAVRELERARGAVDREGATDDTDTDEEDLGTPHACPLWSACPRHSAARDLVDALIWVANPASLVQTAVPRQLNAER------------LRYLELACLRSDIVVVDEADRVQMQLDQMFAPSATLVTKGVS-DSWLDQLQTHEIAELARQGRL---QLS-D-----QDVERWSAALDVVGSAANRLYAMLID----------------DGAM--RDW-AQID-YFSAWTLQEKLLHAWYPLTRGQAAGRPDNMNEEDVEDESTLYEDEEGLGADGDSPVPDVPWVQRRTAITGFFDTFR--DDP-LGGRGPYGTPADE--------LTALAHDVLHTLDEK--------RTRRRVRAL-LDSFLVGAPGPEQR-----------------PMPAAKRGKEPAPEDVPL--------------TEEWRELNARRLEFTLVLAALHQRLDRVTF--LWPQVEAALR--LDSAAHELTRRPP--LDYAPLLPEAPMGN-VLGFQYLVDERAAARNKDGHRTGTLRFFRCAGVGRELLLGLPQLGADPGLGKA--GPHVLLMSGTSWAG--------------------TST-----RAHVLAPVRAVLK--PQPKALDAI-RKTVFRTEFLY---D--------AAGQPIRLSGQD-PDQREDVLRLMIDRLA--RSRSDGNSSPLQSELARI------PD---------QRRKRALLLVGSYAEAKVAATALNEI-PRW---R--GRVRVLAADDAELEAA--VDGTASARSQAPATESGAGAVRRGDLASFA--DDPDAELLVAPLLAVERGHNILTAPQR-----------PG-EEKVAAFGTVFFLVRPHPRPDDLSLAVFAINDWATRFVRG--QLKLPDEGTFCDMV-TQAGDLNAAGSAFRTTARGVWRHVL---SRPYI-YSSLSDD--E----KKSFVWDQLV-------TIWQVIGRLVR----GGVPARVVFVDAAFAPRLAAAQAP-------------FTGQNRRPRRAGDPGLLVRLRDVLAPYFAHTDTDTGTVEYGARTDPADAALVKLLYRPLYEALCAMGMSPGPRPVD-----------------------------     386383243       VREEQPDVKPVQLAQVELALRLLQRLDP--SR---P---------ARDAWVLLGGY--PFARAAGIPVGEQEEIMLTAARHRLWTLRRHRLWEQYLEIYRQLP----QRLRG------YRVTDG---NGA--FRQ-----VVPAVAPWRFQ-HYDAALDGVP-GFST--RSLKLAEAGR-----HSFVDR---GR------VGSVNLP---PELIGG----------PVPGHPLSGSDRRASGALPVPRH-ELAATALWMDQL----------------IDGDWSSRLADLELAVRDG----D--GFRDGGE-LCLDEL-VHLVGMVGAGKST-LMILIAVWAATRDRWNPGAPI----LRTTLVVGDVAEQLRLVDT----F-RSL--GL-----------RAVPLLGPT--TRETHIQRLHRRL---ASQGEHRLLLHDAA-GFDDLSTVCVVDALR------------DEQAQPLRYADGP----CTGLYPKGPDP----GADDGAGEPGRSGEDPGRAPSYRPTGRRGRNESLPDDRPETALLGTPAGCPLWSGCPRHGVARDQVEAEIWVANPWSLVSSSVPAHLNEER------------IRQLELTCLRSDIIVVDEADRVQMIFDQIFAPSATLVTQGLA-ESWLDQVQTKKIDELAQQGRL---QLT-D-----RDIAGWDAALSIVAAATNRIYALLIA----------------DPAL--RTW-VDID-YFNTWTLQEKLLSEWFPTWRSADDPDGTP-DERLLYEEDEQAEEDQEVSTG---LPGEEPWAERRDEVQTLLDRVR--DDP-LGNNGSYEGDIRL--------LLECVNDLLHGLDER--------RARARVLKL-LDRLLQGSPAVDGS------------------AGRPHPGDTGDPADVLG--------------SDAWRKRSVRRLSFTLLLYALERRLDRLTR--LWPQVEAALH--LDATGNELSRKPP--LDYAPLIPEAPMGN-VLGFQYLVDKLDQSPDKDGLLSGTLRFFRCAGIGRELLLGLPR-LGNGGGPPQPDGPRVLLMSGTSWAG--------------------TST-----RAHVLAPVQVVLK--PNVKAREAI-RQTVFRTGFLFEP----------ETRKPLTLSGQR-PENRSTALAQMIRRLA--ERPRPGMTSPLEDELKEI------TD---------PQRKRALLLVGSYRDATAAAEQLHAV-KRW---Q--GRVRVLTADDAELDEP--PPATADDSTTVT-------SVRRGDLASFA--EDPEAQLLVAPLLAVERGHNIL---NK-----------QG-K---AAFGTVLFLSRPHPRPDDLYLSVFAINDWATRFVRD---RPDAHGGTFSGLV-ADSAGLDEAGQEFRRKARGEWRRLV---SRRYV-YSRLEEH--E----KESFAWDQLV-------TLWQVIGRLVR----GGVPARVVFVDAAFAPDLAAEQAG----------------GGAPRRPRRDPGLLVRLRRVLEPYFT-------------EHKGPDAELVRTLYEPLYKALVELNVTPAPPP-------------------------------     294816303       VLIDQPHVKPVQLAQIELALRLLQRLDP--RR---P---------ARDAWVLLGGY--PFARAGGITIGPREETMLAAARHRLWRLRRHRVWEQYLEIYRQLP----KRLRG------YRIEEG---TGA--FRQ-----VVPAVAPWRFE-KYDAALEEIP-GFSV--HRLTLAPAGR-----SVLVDR---GS------ATTVTIPAELIGE-------------RAPGHALTDATGRYRGPLTIPRE-QLAATARWMDER----------------IDGNWLERWDNLELAVRDK------DGFREGAD-LRLDSL-LHLVGMVGAGKST-LMILIAVWAATR-----GEPEGSQALRTTLVVGDVAEQLRLLDT----F-RSL--GL-----------RAVPVLGPT--TRETHVQRLHRRL---ASQGEHHLLRHDAT-GFDDLSTVCVVDALR----------DGSAE--PLRYAEGP----CSGLHPLGPEAEQGDGHGAGGADGRTGPQEDAPPSYRRKARNERRATAAEGQRPESALRREAHGCPLWSACPRHGAAREQVEAEIWIANPWSLVSTSVSAHLNTER------------LRQLELACLRSDIVVVDEADQVQMTFDRIFAPSATLVTQGLS-DSWLDQVQTKKIDELAQQGRL---QLT-D-----RDIAGWDAALSTVVDTTNRIYALLIA----------------DPGL--RAW-VDID-YFNTWTLQEKLLSEWFPTARAADDPDGTP-DERLLYEDDAPADDGEDDEAFPRFLPGEEPWAERRDEVRSLLDRVR--DDP-LGNDGSYGGDARL--------LLECVNDLLHGLNDK--------QVHERVLEL-LDRLLQGSPCLDGR---------------VPPPRPRAPADPPKPADLPG--------------SAEWHRRAARRLSFTLLLYALERRLDRLTR--LWPQVEAALH--LDATGNELSRKPP--LDYAPLIPESPMGN-VLGFQYLVDTFDHAPDKDEPLSGTLRFFRCAGIGRELLLGLPR-LGNGGGPPRPDGPRVLLMSGTSWAG--------------------TST-----RAHVLAPVGAILK--PNKEAQAAI-RRTVFRTGFLYEP-E--------SSGKALSLSGKR-LEDRPAALAQMVRRLA-DSGRPGLV-SPLEEELKQI------TD---------PQRNRALLLVGSYRDAAAVAEQLHAM-KRW---R--GRVRVLTADDAELDEP--PPAAAEDGTTVT-------SVRRGDLASFA--QDPEAQLLVAPLLAVERGHNIL---NR-----------AG-K---AAFGTVFFLARPHPRADDLYLSVYAINDWACRFVRD---RPDVHGGTFTRLV-AEAAGLDEAGQEFRRRARAEWRRLV---SRRYV-YSRLEEH--E----KVSFAWDQLV-------TLWQVIGRLVR----GGVPARVVFVDAAFAPALADEQAERTARQNRSATGQNEPAPRRARRKPGEPGLLPRLRLVLEPYFT-------------ERTGTEAELVRTLYEPLYEALASIRTDPPPRP-------------------------------     254391264       ----------------------MQRLDP--RR---P---------ARDAWVLLGGY--PFARAGGITIGPREETMLAAARHRLWRLRRHRVWEQYLEIYRQLP----KRLRG------YRIEEG---TGA--FRQ-----VVPAVAPWRFE-KYDAALEEIP-GFSV--HRLTLAPAGR-----SVLVDR---GS------ATTVTIPAELIGE-------------RAPGHALTDATGRYRGPLTIPRE-QLAATARWMDER----------------IDGNWLERWDNLELAVRDK------DGFREGAD-LRLDSL-LHLVGMVGAGKST-LMILIAVWAATR-----GEPEGSQALRTTLVVGDVAEQLRLLDT----F-RSL--GL-----------RAVPVLGPT--TRETHVQRLHRRL---ASQGEHHLLRHDAT-GFDDLSTVCVVDALR----------DGSAE--PLRYAEGP----CSGLHPLGPEAEQGDGHGAGGADGRTGPQEDAPPSYRRKARNERRATAAEGQRPESALRREAHGCPLWSACPRHGAAREQVEAEIWIANPWSLVSTSVSAHLNTER------------LRQLELACLRSDIVVVDEADQVQMTFDRIFAPSATLVTQGLS-DSWLDQVQTKKIDELAQQGRL---QLT-D-----RDIAGWDAALSTVVDTTNRIYALLIA----------------DPGL--RAW-VDID-YFNTWTLQEKLLSEWFPTARAADDPDGTP-DERLLYEDDAPADDGEDDEAFPRFLPGEEPWAERRDEVRSLLDRVR--DDP-LGNDGSYGGDARL--------LLECVNDLLHGLNDK--------QVHERVLEL-LDRLLQGSPCLDGR---------------VPPPRPRAPADPPKPADLPG--------------SAEWHRRAARRLSFTLLLYALERRLDRLTR--LWPQVEAALH--LDATGNELSRKPP--LDYAPLIPESPMGN-VLGFQYLVDTFDHAPDKDEPLSGTLRFFRCAGIGRELLLGLPR-LGNGGGPPRPDGPRVLLMSGTSWAG--------------------TST-----RAHVLAPVGAILK--PNKEAQAAI-RRTVFRTGFLYEP-E--------SSGKALSLSGKR-LEDRPAALAQMVRRLA-DSGRPGLV-SPLEEELKQI------TD---------PQRNRALLLVGSYRDAAAVAEQLHAM-KRW---R--GRVRVLTADDAELDEP--PPAAAEDGTTVT-------SVRRGDLASFA--QDPEAQLLVAPLLAVERGHNIL---NR-----------AG-K---AAFGTVFFLARPHPRADDLYLSVYAINDWACRFVRD---RPDVHGGTFTRLV-AEAAGLDEAGQEFRRRARAEWRRLV---SRRYV-YSRLEEH--E----KVSFAWDQLV-------TLWQVIGRLVR----GGVPARVVFVDAAFAPALADEQAERTARQNRSATGQNEPAPRRARRKPGEPGLLPRLRLVLEPYFT-------------ERTGTEAELVRTLYEPLYEALASIRTDPPPRP-------------------------------     357392956       --GEESPPRARQLCRVEIGLYALDRLCP--GQ---P---------AKAGWTLFSGY--PYALV--GADSAERERALRILRHLGWSMRRPYAWDACLEKYRDYP----ERVRG------FELGSL---SEP--PKR-----LDPAAANHRWK-VYESVLVDTPKRHLR--PGLKQAEEGR----TYRFPLR---NG------TGHVRIPEKFPAY-------------SAQGYDLEAIDRRRPGPVEVVRG-ELLETADWMDREL------------GPSRGKTWVERVESINFDVLTQ----DGQEYVERQW-LTIDGM-FHLAGMVGAGKST-FRDVLTVRLAKQ-----R-------RRVTLVVGDIAEQLKLVAL----F-TDL--GI-----------KAAPVLGAS--TRVRNTQRMHRRL---ATAKAPTLLAHDPT-RFTYLSTACPLDALR---------SMEGSE--PLLFSEAP----CSHLRAVETKE----------------------PPVPVFGRAGRAAAAQP--RGDEEETGPDRSCPLWAKCPRHQAAHSLVDADVWVANPASLVYSLVPRQQNRER------------IRLLELAAARSDLIVVDEADQVQMQLDALFAPSATLVGRAP--DSWLDDLYSHTIDELARRARL---QLS-K-----STVDDWTSALNTVSAAADRLYARLLR----------------HSDL--RAW-VQAD-YFSTWTLQQKLVNNWFGNPGGADPESEEE-SEESEEPADAVEEEPAEAAGEGAPESAGKTAGSARERLFDLFEQFR--EDP-LGANTTNDPQLAE--------LVGITQDLLHGLAES--------AVHHRITPL-LRTLSGRPSLEEIT------------------ADLQAAGRRLDAAEERR--------------LDEELKPEIRRFEFTLLLAALQERLDAVTD--QWVTVEDELN--LESASNHLAKRPP--IDYGPVVPESPMGN-ILGFQFLPE----DLDDAGSGSGALRFFRCTGVGRILLSELIE-SAAPIDGP---CPNVLLMSGTSWAG--------------------TAP-----GAHVRAPVRAILR--TPKREHANV-LTSVFRTHFVH---G--------LDGKALHLSGTR-PEERPKMLELMLRQLA--APVEGRRTSVFDEEIRLI------GD---------HQRKRLLLLVGSYDEARRAAALIHKM-PQW---Q--GKVCQLIADDAELEP---WSYGGPPEQDSDEDHGPA-VLRRGEVATFG---ETGAQILVAPLMSVERGHNIL---ND-----------AG-K---AAIGSVFFLARPHPRPHDLNLAVQKINRWVEEQLDI---GGTTSGSEFARLV-GASADLDEAGRAFRRLGRIEWRTLL---TRQAA-WSRLDDE--E----KAAFTWDRMV-------IMWQVIGRLVR----GAVPARVVFVDSKFAEREAAGRGR----------------------DTHRTGLLASMLHVLAPYFDRTS----------GIARRDRQLVEALYGPLHKALAEMLDNGTTAAGPAAVRRQGGDQRW-----------------     302867038       ---APAGFGIGDLLDVELAIYLGTRVMP--TR---P---------VRDIWTLLSGY--PYEEVFGDVRTSEARLMIRRARHFLWDRSRRYTWHNSLHMYLDVD----ITLRG------YTFATI---DDL--PKA-----LEPTRAHDRRE-IYERLLTQPP-PLER--RELPVAGAGD-----QLFYVR---DQ------RHSVNIPVELIGLK------------PWIGHDIGAAPAGRGEPITVTMT-ELEAAADAMDALEVAANAADLAENRTPRKLNDWKKRLRRVELLLRDE----AAGAFQTHPR-LRIDRL-MNLVGMVGAGKST-IRDILAFQLATRERPADR-------RRTTIVVGDVAEVLTITEQ----F-RRL--GV-----------HAAPILGKS--TLERNIQRLHRR----SESARQSMLNHDNP-GFAHLSNACPLDALR-----------GFEARRALAITAAP----CTALYNADDVE------------------------KAEIEHDDPDSASTRFRRRRKPARPKRHGCPLWTACPRHSAARDLVDAQIWVATPASLVHSRVPDHLNDEN------------MRYLELACRRSDLIIVDEADRVQMQLDTAFAPAATLVARGA--QSWLDEVGKHKLTELAAQGRL---QLS-S-----DEIDDWNNALNTVITATDRIYSMLIN----------------DEPL--RRW-ITQD-YFSAMTLHQWLINEWF--PELRRRNRDDD-ENVVAPDEDKLASQRAEL-----------------QRVDTVLGSFR--DSP-LTPLRGSGPEDTTTDLVNA--LVAMATELLHAPRGVF----VREFSRERLKKL-LRQLVPNNASVAGD-----------------------------------------------------LDANLHRFEFTLVLAVLHDRLDRMTI--LWPRVEAALN--LDSTSNVLSRRPP--KDYMPVVPESPMGN-VLGFQFQAG----DRNEDGDQSGELRFFRCNGVGRELLLELAD-IPAVDNRP---GPHVLLMSATSWAG--------------------TSS-----RYHLHAQVDAVLR--PHEHEVAAI-KKSNFRKLFLYPP----------GVTTALRLSGTD-PRQRPAQLEQMLHQLAVPDRSLTGATSLLQQELDDI------DD---------PQRRRILLLVGSYAEAHRAGHYLNNL-PEW---T--GNVTVLVSDDTDLDHS--WSN-LPADTRQL-------HLRRGELAKFP---DIGGQLLIAPLLAVERGHNIV---VP-----------GG-K---AAIGSVYFLARPHHRPDDISLATQAINDWAVRQVRG--------PGRRFTQTALAAGSPDDAGLRFRAEAARKWHRFL---TRRLS-WTSLPPD--E----KSAFTWDQLV-------VIWQVIGRLVR----GGVAARVYFVDGAFSPREAGFTGD----------------------DTPATSLLASMLHELAPYFDPES----------AQTTIDRSLVQALYDPLYRALRDMN--------------------------------------     291439985       WPTNLGSFRPGDLLDVELGLYLLQDVTP--AR---T---------AADVWPLLGGY--PYSEVFGDVRTDEQRLRVLRARHYLWDMRRRHAWSEALADYLKVP----QHLRG------YDIDGP---GSV--PRR-----RETSRAARRFE-IFEELLSTAP-RFAT--RSIPFAEEGE----HTFQVQD---RT-------HSVVFDEELLTDT------------EPRAHALDAPPAGHGEPVDVTWE-ELEKAADEMDAAE---------STAPKGPRNDWAGRLRRVKLLVRDA----ELGRFTEQGR-LRIDRL-LHLVGMVGAGKST-LRDILTYHLVTR-----TP------RRVTLVVGDVAETLAVVEQ----F-DRL--GV-----------RAAPVLGHS--TRERNINRLHRRT---ATAGAATMLGHDHA-GFTYLSSACPVDVLR-----------GLEARRPLGVREAP----CITLYTVPEQE--------------------------ATDGDPLLDDENSTQRDARGPKPQRRLCPLWNRCPRHRGARDLVDAQVWVATPAGLVHSSVPLHQHEEQ------------LRYLELACRLSDLVIVDEADRVQMQLDTVFAPASTLVGKAP--NSWFDEVAVHKFQQMARDGRL---RLS-A-----RDVDDWTNAVNTVSAATDRLFALLVK----------------DDKL--RRW-ISVD-YFSAFTLHNLLLDSW---------FPGRK-EGERPPEAA--------------------------RTLDGALGRFR--DDP-LHEQQSTGNDDEVTPLVNT--FVHLTLELLHAPQGA--------RTRERLRDT-LTDIVGDGTTVLAD-----------------------------------------------------ADLQARRFEFTLILSALHSRLDFMTT--LWPRVEAALN--LESASNVLSRRPP--KDYEAVIPESPMGN-VLGFQFRHD----DCERDGDRSGELRFFHCNGVGRELLMRLGD-LCAVDGRP---GPHVLLMSATSWAG--------------------TSS-----RYHVHVEVGAVLR--PHDEEVEAV-LGSEFRKEFLYWPGN--------DRPKPLRLSGCD-PEERPQALLHMLHQLAVPDRSLPDAESMLDAELKEI------DD---------PDRRRILLLVGSYDEARRAAEYLNQI-PEW---N--GRVTRLVSDDADSDTA--WTR-LPEDAAVR-------TLPRGDVRAFP---SVGGDILVAPLLAVERGHNVV---LR-----------GG-K---AAIGTVYFLARPHPRPDDIALAVQSINDWAVRQLRD-------LDGSFRQNA-LAAATPDQAAVAFRSRARRQWNRFL---TRRLA-WSSLRDE--E----KAAFTWDQLV-------VMWQVIGRLVR----GGVPARVVFVDAAFSPREAGFQNA----------------------DTPDTSLLASMREVLAPYFEDGNVTER------DPAPIDRSLVRELYEPLYRALVDMG--------------------------------------     357393638       ---RLRGPAAEYLCQIELGLYLQQRLTP--DA---P---------AAHAWVFFSGY--GFAKAYLADPPEDVERVLRIARHSLWTLGRNRAWRDALEHYRRVD----PALRG------YEVPDP---GRP--PVR-----CETGIGRERWQ-VYDGLLRTAP-PLAG--SRQRVAGAGR----HGFPVSR---AM-------AVVELPEVPEG--------------PPASHDLDLGPAGGGEGLTFRMD-DLRSTAAEMDAIH---------ARTGSGKAPEWERRLRNFELSNAQI------GTFQPADE-FTVDGI-QHLLGIVGAGKST-LRDVIAVHLARL-----G-------KRTTVVVPDVAEVLKLVES----YNLYT--EG-----------AAAPVLGAG--GRERHAQSLHRRL---AGRGEQRLLAHDDP-AFAYLGTSCLLNTML-----------GGPSGEPLAFGEAP----CSRLRPPAPSA------------------------------------RGAGRSLRSEWQKQSLACPYWSVCPRHHGARALVGALIWVATMPSLLDSSPPRPQNGER------------IRYLELACRRSDLVIVDEADRVQMNLDRAFAPAVLLAADEQ--RGFIDRLNKHKIRELTAGGRT---QLS-D-----RDVAVFCGALNTVVAATDRLHAMLVS----------------QYRL--RRW-TRFG-FFSAWTLQLALLDER-------YPPGGDD-GAARSDRTPR-------------------------EALGDLFDAFR--DNP-FGDRTRHTEEDFAD-------LTALLNELLHTGNPE--------KTRESLLEVMEDRFRLDERFRTRQRQQYEERVAQWQEESAERGHGNGRRGKAVDGPPRT--------------PEQWFEDLADRLEFTLILSALERRLALVNT--MWPPVEAALG----LGFNDMYRRPF---DYGPMVPEAPMGN-VLGFQFRVT----GEDGEGVRGGDLVFFRCSGVGRELLRAMPA-LSSVDCRP---GAHVLLMSGSSWAG--------------------RSS-----RYHVAVPVGVIIE--PVPAVTERIAVESEMRFEFV----D--------DGDERMRISGTD-PEDRPAKLRRLIGRLG-AGADEAENGGPLEEELLSL-------P---------PGRDQILLLVGSYEEAAAVADTLHNLNPRW---R--DRVLRLVSDDQEID----EDAEAPSSHRAR-------VLRRGDVEHLK---DLNADVLVAPLLAVERGHNIL---ND-----------DA-V---AAIGTVYFLARPNPHPDDLFLAVHAVNDWIVRAQQD---------GDFARWV-ASAETIEAGAEEVRRRARSRWYRLL---RRSTA-WSRLGDD-------REQITWDVLV-------LMWQVIGRLVR----GGVPARVVFVDAAFAPGRAAEPPV---------------------ADSPESSLLHSILAVLDPYFEDG-----------ARPADEQFIARALYAPLRSMLGRCLARPLPAA-------------------------------     302870453       QTGELRSRELVRLCEVELGLRLQQHLAP--ES---P---------ATDAWVLFSGY--PFAHARGLVADQQGEQMLRVARYSLWTLRRKTAWEEALRSYDQTD----PSLRG------YDVPDI---SRP--ATE-----RLSAFAPDRWD-TYDLLLRCAP-PFRR--TEMPVAGDGP----AEFPVGR---HS-VTIRLSGTGNMP-------------------APTAHDLGLLPAGGGAPICLRWR-DLLETAAEMDEV----------------LSQNWVDRLSRTHLFTSSG------GAFAPDEE-FSVAGV-QHLLGIVGAGKST-LRDVITVYLVNR----HG-------FCITVVVGDVAESLKLVSL----YNTYLTAGR-----------TAAPIIGAS--GRERHAQRLHRRL---ASRGEQNILSHTDP-SFTYLSTSCAINALL----------PEDDD--ILPYGAAP----CARLQGRSRRE---------------------------------------RREEESGRSGKRHACPYWSGCPRHHGARELVDAHVWVATPPSLIDAAVPRVQNAEH------------LHYLELACRRSDLVIVDEADRVQMQLDRMFAPATKLVAGVGD-RSLLDELNAHKIRELADGERL---QLS-N-----RDVEVWNAAVNTITAATDRLYAMLVG----------------DHAL--RTW-VRIG-YFNAWTLQLQLLDER-------YPEAGEP-DR---------------------------------VELEQRLDEFR--DNP-LGDRRPELRRG----------LVDLVTELLHTSYQE--------HTRLRLVAF-VVDLFNLPPFLADK--------------QCVFEKASEAPRRKRAKPPQS--------------PDDWLSEWTRRFEFTLLLAALESKLSLMNA--MWPRVEATLK----LGFNEMYRSPL---SYGPVIPEAPMGN-VLGFQFLVN----GPDSGGVRSGELRYFRCNGIGRELLHAMPD-IPLADGRP---PTNVLLMSGSSWAG--------------------KSS-----RYHLRFPVGVLLK--PDPEEFEALVAGTEIRLELLG------------EDAPQLRVSGAR-PEARTQILTQMALRLG---ETQDGEASMLEKELLRL-------P---------EARRHLLLLVGSYDEAGAVADTLHTLGNRW---R--GRVLRLVADDFDEEFA--TDPFDDDDQHAG-------ILRRGDVDTLR---NTSAEVLVAPLLAVERGHNIL---NS-----------DG-Q---AAIGTVYFLARPNPRPDDIGLAVHAVNDWMIRSIDS---------GEFDDWV-RSAATLSAGAREVRRQARQTWYRVL---RRSLA-WSRLKDD-------RPAVTWDMLV-------LIWQVIGRLVR----GGVHARVVFVDAAFMPNLGDGTKL---------------------PDTAETSLLYSIAEVLAPYFLTDS----------EVSPLDRDIVQSLYRPLWDALDRFLPPTEEKNETCTR--------------------------     395777309       -----------------------MGACP------------------ETSWPL---------------------------------------------------------------------------------------------------------------------------------------------------------------------------------------------RESLSFAWD-DLKATAARMDSI----------------RFENWTGRLDAIRLFVRRP------AGFKSAKA-FEVAGI-QHLLGIVGAGKST-LRDVLTVNLAER-----N-------RRVTVVVGDVVELLKLVQM----YNTYL--PG-----------TAAPVLGAA--GREQHAERLHRRL---AGRGERNLLAHDDR-GFGYLSTSCALNALR--------HAQGQPADHLLAFGEAP----CTRLQRPNPGG-----------------------------------------YSEDLWSRRAVACPLWTACPRHRSARELVDAIIWVATPPSLVDSKVPHPQNREH------------IRYFELACRRSDLVIVDEADRVQMQLDRMFAPGIPLLGGGPDTRSFLDEVNQHRIRELAGAGRI---QLS-D-----RDVENWSAAVNTTQTAADRLVAMLVS----------------DSDL--RKW-VRTG-YFSAGRLQQWLLEERYPLPEDQDGIEPEN-SGSANEQLHRKRR----------------------RELSEVLDAFR--DNP-FGDRRTATGIGTE--------LTGLTGELLHTNYRA--------RTRERLREV-ICSLLDLDGLLEPTEKGRERYAELRDLAEGDQGQSTTRQPSDRATPKRRETKRRKTKSRLPEDPEVWVKLLMIRFEFTLLLSALEPRLALINA--MWPRVSSALN----LGFNEMYRRPP---DYGPMVPEAPMGN-VIGFQFLLEG---GPDQGGVRTGELRFFRCSGVGRELLRAMPG-LPTVDGHP---GTNVLLMSGSSWAG--------------------KSS-----RYHIPVDIGVIIE--PDPRELDQIAEQSEFRFEFIRYRCDCRAACECHERDDAQRLSGEP-LDQRADILRRMVTVLG----DNATGTSRLQEELGLL-------P---------ESRRHILLLVGSYEEAKVVADTLHTLNKRW---Q--DRVYRLVSDDDPDA----GTGTDDDEYRAP-------VLRRGDVENLA---NTRAEILVAPLLAVERGHNIL---DV-----------DR-RH--AAIGSIYFLARPNPRPDDLGLAVHAINDWIVRARGS---------GDFDGWV-RSEPSLALGARKVRDLARAKWFRVL---ARSMA-WKGLGED-------RESVTWDLLV-------LIWQVIGRSVR----GAVPTRVAFVDAAFAPKLAVGQPT-------------------DEPDSPDTSLLHSIHAVLKPYF--------------------------TQGT-----------------------------------------------     consensus/100%  ..................................................................................................................................................................................................................................................................................................................................h.u..s.GKo..................................s...............h....................s..h.u...........................................C...................................C...............................................................Csh...C...........u.lhh.p..sh.............................b........hhhDE.-..b............h............p...............................................................................................................................................................................................h................................................................................................................h.............................................................................h......s.s+.....h..................h..Suou.................................pl.......h...........................................huG......+............................b........................phhh...s..........h...................................................h.......h..........h..s...h.Ru.Nhh.......................shh...hh..b......p.......h.............................................................................................h.Q..sR..R..........h.......................................................................................................................................     consensus/95%   ...................................................h.......................................h........................................................................................................................................................h.......h............................................................h........h.u..usGKos.h...........................shh.ss..............h.p..................s..h.u.....b.................................h...C.h.s...............................C...............................................................CPh...Cs.........pu.lhl.p..sh.............................E.h.....hhhhDEhD.hb..h.........h............p...............................................................................................................................................................................................h.......................................................................................................h........h...h..h................................................uh....................l......u.s+.....h.................hh..Suou........................s........cl.....h.h...........................................hSG......+...h..h...h.............h...h........................phhh.ssS...s..h...l..................h................................h...ph..h........plll.sh..h.Ru.Nlh.......................shh..hhhh.R......p.......h..................................................h..........h..b..........b....h..h.........h.Q.hGR..R....s.....h.h.D..h..............................................h...............................a......h..........................................     consensus/90%   ...................................................hh.....................h................h...h..h..........................................................h................................................h.....................................h.h....ph...u..h...........................h.p.......................h........h.u..usGKos.h...h...h..p..............p.shh.ss.....p........h.p..................ss.l.u....pb.................................hs.sC.l.s..........................s....C...............................................................CPh...Cs.....p...pA.lhl.s..sh..........................ph.Ehh....phhhhDEhD.hb..h.........h............p.................................h.............h............................................................................................................................................h..h.......................................................................................................h.....h..h...h..h......................................s.s.....h.uhb...................l.h....u.s+..h..h........s........hl..Suouh.s.....................s........cl...s.h.l..............p............................lSG......+...l..h...l.............h...h......................p.+hhlhssS..ps..h...l..................hs...............................h...ph..h........plLlssh..h.RG.Nll.................s.....uhhsshhhhsRP.....s.......hp..............................s........b.........h..........hp.b..........b....h.bhh........h.Q.hGR..R....s.....h.hhD..a..........................................h...h............................p..a.....sh..........................................     consensus/85%   ...................h....ph.s.......................lh.....................h...h......b.....h...h..h.p.................hp............................p....h...l................................................h.....................................h.h....pL...u.bhs..........................h.ph.....p................h.hs.h..pl.u..usGKos.h..hh...hhpp..............p.shh.ssl.p.bp........h.p.h...............psssl.u.p..pb.pphppb..........................hs.sC.l.sh.......................p.P....C..l........................................................p...CPhh..Cs.....pph.pAplhl.s..uhh.s.........p............bph.Ehhh...slhllDEhD.hb.ph.........l.......ps...ph..p.............................h.............h.......................................s...l..p.........................................................h...h..h............................h..h...h...........................pb......................................................................h.....l..hp.pl..h................................p...h.P.s.hG..h.uhbh.................plph..h.G.s+.hh..h........s........hl..SuTuahs.....................us......hcl...s.hhl..............p..b.........................lSG......R...l..hhp.l.............l..ph..h..................p+.+hhllssSa.ps..h...l..............ph..hs.s.............................l..spl.phs.......plLlsPh.sh.RG.NIl.................s.....uhhuslhhhsRP.s..ps....h..ls....p.........................s........R..s...h..h..........ap.b..p.......b..h.h.bhl........h.QhhGR..R....Gsss..h.hhD..a.......................................l..h...h.......................p...hp.ha.shh.sh..h.......................................     consensus/80%   ...................h.h..ph.s.......s............h..lh.s...................h...hb.b...h.p...h...h..h.p.................hp............................pb...h...L...........p....s..s............................ls....................................hph....cL...A.bhc..........................h.ph.....p................hphs.h..pl.u..usGKSs.h..hhs..hhpp.....s.......b+hslhhssl.p.bp.h......h.p.h..sh...........pssPlhu.s..pb.pchpph........s..............h..hu.sC.l.uh......................sp.P....C..l........................................................p...CPhh..Csp....pph.pAplhl.s..uhh.s.........p............bphhEhhhb..clhllDEhD.hQ.ph.p..s.p..L.s.....pshh.pl..p...............h.............h...b.....s.s.h..bb.p...................h..b..........ss..l..p....b....................................................h...hp.h...ps.......................h..l...l.........................h.phh........p............................................................h.b...l..hp.pl..h................................p...hhP.s.hG..l.Ghbh................spLphh.h.G.s+.hh..h.p....s.s........ll.hSuTuahs.....................us......hcl...s.hhl..............p..h.....................p.h.lSG....p.R...l.bhhp.Lh..........s.l..ph.ph..................p+.+hhllssSY.pu..h...L..............ph..hs.s.............................l..spl.phs...p...plLlsPh.sl.RG.NIl.................s.b...uAhuslhhhsRP.s.ss-....h..ls....p.........................s.....p..Rp.u...h.ph....p.....apphs.p.......+..h.hsbhl........lhQhhGR..R....Gusss.l.hhDsuF.......................................l..h...h.......................p...hp.lY.shhpuh.ph.......................................     consensus/75%   .................h.l.hh.ph.s...p...s.........h.ph..lhsGb..............p.b.hh..hR.b...h.p...a...l..h.p.................aph...........................cb...h..hL...........p....s..G.......h..................h.lP..........................p.........lsh.bp.-L...Apbh-.....................ph.ppl.ph.....p................lphs.h..clhu.sGuGKSs.lh.hhsh.hhpp.....s.......b+lslhhssl.p.bp.h......h.p.h..sh...........pssPlhu.s..pb.p+hpph........s.s............h..hu.sC.Lpuh...................h..sp.P....C..lb.......................................................p...CPhhs.Cspp...cphhpAplhlss..uhhpsph.......p............bphhEhhhbpsDllllDEhD.lQ.ph.p.hs.p.pL.s.....sshhppl.pp..pb...........h..p......p..pa...h..h..s.s.lh.hb.p...................h..bpb....s..hsshpl.ppbhp.b...........s...pp.................................pph...hp.h...ps..h....................h..l...l...............p.b.ph.ph.h.phh........p...........................................................pl.h.hhl..lp.pl..l.......ph...hp.......s.b...s....ph..hhPpushGp.lhGabh..........p...p.upLphhph.Gss+.hl.phsp....s.s.....s..ll.hSuTSahs.....................us......hcl...s.hhl..............p..hb....................psl.lSG....ppR...l.bhhp.Lh..........s.lp.cl.ph.......s..........pR.+hlllssSY.pAc.lhp.L.p............ph..hs.sp.........p..................l.bscl.pFs...p...plLluPh.sl.RG.NIl................ss.b...uAhGslhhlsRP.P.scD....l..ls.bs.p.h.....................s.s....hpphRp.u...h.phb...p.....apphspp.......+p.h.hsbhV........lhQhlGR..R....GGsss.lhhhDuuF.s...................................uhl..h...Lp..h...................p...hp.lY.shhpul.ph.......................................     consensus/70%   ...............hphhl.hh.ph.s..sp...s.........hppu..lhsGb......h.......p.b.hh..hR.b...h.p.p.a..slp.abp........b........acl..s.......................pch...hpphL.p.........p....s..Gp......h..................hplP......................sp..ph.s......lsh.hp.-L...ApbhD.....................ph.pplpph.hp..p................lplssh..+lhG.sGuGKSs.lhphhsh.hhpp.....s.......b+lslllsslspshpbh......h.ppl..sl...........pssPlhu.s..pb.p+hpph........sps....b.p.....h..hu.sC.Lpuh...............p..sh..sp.P....C..lb.......................................................p...CPhhs.Cspp..h+chhpAplhlss..uhlpspls..h..pp............hphhEhhhbpsDllllDEhD.lQ.ph-p.hssphpLss.....sshlppl.pp..pbh..........h..p......ph.pa...h..h..sssblh.hh.p...................h..bph....s..hsshpL.pphhpph...........s.p.pp.................................pphbphhs.hb..ss..hs...................l..l..plhp....p........p.b.ph.ph.lpphh........p...........................................................pl.h.hhl..lp.pL..l.......ph..hhp......ss.bh.ps....ph.sllPpushGp.lhGabh..........p...ppupLphhph.GssR.hl.phsp....s.u.....ss.lLhhSuTSahs.....................us.....paHlph.sphll..............p..hb...................spslplSG....ppR..sLpbhhc.Lh..........s.Lppclppl.......s..........pRp+hLLlssSY.cAc.lup.L.ph...........ph..ll.sp.........p...ss.............l.+scl.pFs...p..splLluPl.sl.RGaNIl...p............su.c...uAhGslhhlsRP.P.scD.s.hl..ls.bs.p.h.....................s.s.s..hpphRp.u...h.phh...p.....apphspp.......+p.hshsblV........lhQlIGR..R....GGsssplhahDuAF.s...................................uhL..h..hLp..h...................p..hhp.LY.shhpAl.ph.......................................     ```      Back to Contents       ---      **- A. Gene names, gi numbers, phyletic distributions, and domain architectures of the MedPIWI family**       ```     Proteins lists are grouped into shared domain architectures, which are provided at the top of each group following the '#;'.     GI              Gene name               Length  Class                                                   Species                                                         GenBank defline     #;Med13_N+ZNF+linker+MID+MedPIWI     296478636       BOS_16685               2674    eukaryota>metazoa>chordata>vertebrata                   Bos taurus                                                      mediator complex subunit 13-like [Bos taurus].     344295372       MED13L                  2413    eukaryota>metazoa>chordata>vertebrata                   Loxodonta africana                                              PREDICTED: mediator of RNA polymerase II transcription subunit 13 [Loxodonta africana].     194675789       MED13                   2332    eukaryota>metazoa>chordata>vertebrata                   Bos taurus                                                      PREDICTED: mediator of RNA polymerase II transcription subunit 13 [Bos taurus].     326930078       MED13L                  2272    eukaryota>metazoa>chordata>vertebrata                   Meleagris gallopavo                                             PREDICTED: mediator of RNA polymerase II transcription subunit 13-like [Meleagris gallopavo].     403281842       MED13L                  2268    eukaryota>metazoa>chordata>vertebrata                   Saimiri boliviensis boliviensis                                 PREDICTED: mediator of RNA polymerase II transcription subunit 13-like [Saimiri boliviensis boliviensis].     345805272       MED13                   2262    eukaryota>metazoa>chordata>vertebrata                   Canis lupus familiaris                                          PREDICTED: mediator of RNA polymerase II transcription subunit 13 [Canis lupus familiaris].     47210173        GSTEN:00004927:G:001    2253    eukaryota>metazoa>chordata>vertebrata>actinopterygii    Tetraodon nigroviridis                                          unnamed protein product [Tetraodon nigroviridis].     403275344       MED13                   2233    eukaryota>metazoa>chordata>vertebrata                   Saimiri boliviensis boliviensis                                 PREDICTED: mediator of RNA polymerase II transcription subunit 13 [Saimiri boliviensis boliviensis].     332840500       MED13L                  2230    eukaryota>metazoa>chordata>vertebrata                   Pan troglodytes                                                 PREDICTED: mediator complex subunit 13-like, partial [Pan troglodytes].     348567424       LOC100729262            2213    eukaryota>metazoa>chordata>vertebrata                   Cavia porcellus                                                 PREDICTED: LOW QUALITY PROTEIN: mediator of RNA polymerase II transcription subunit 13-like [Cavia porcellus].     44771211        MED13L                  2210    eukaryota>metazoa>chordata>vertebrata                   Homo sapiens                                                    mediator of RNA polymerase II transcription subunit 13-like [Homo sapiens].     387849173       MED13L                  2210    eukaryota>metazoa>chordata>vertebrata                   Macaca mulatta                                                  mediator of RNA polymerase II transcription subunit 13-like [Macaca mulatta].     395833974       MED13L                  2210    eukaryota>metazoa>chordata>vertebrata                   Otolemur garnettii                                              PREDICTED: mediator of RNA polymerase II transcription subunit 13-like [Otolemur garnettii].     402887785       MED13L                  2210    eukaryota>metazoa>chordata>vertebrata                   Papio anubis                                                    PREDICTED: mediator of RNA polymerase II transcription subunit 13-like [Papio anubis].     410221240       MED13L                  2210    eukaryota>metazoa>chordata>vertebrata                   Pan troglodytes                                                 mediator complex subunit 13-like [Pan troglodytes].     300794669       MED13L                  2209    eukaryota>metazoa>chordata>vertebrata                   Bos taurus                                                      mediator of RNA polymerase II transcription subunit 13-like [Bos taurus].     301770725       MED13L                  2208    eukaryota>metazoa>chordata>vertebrata                   Ailuropoda melanoleuca                                          PREDICTED: mediator of RNA polymerase II transcription subunit 13-like [Ailuropoda melanoleuca].     83305883        -                       2207    eukaryota>metazoa>chordata>vertebrata                   Mus musculus                                                    RecName: Full=Mediator of RNA polymerase II transcription subunit 13-like; AltName: Full=Mediator complex subunit 13-like; AltName: Full=Thyroid hormone receptor-associated protein 2; AltName: Full=Thyroid hormone receptor-associated protein complex 240 kDa component-like.     146134449       Med13l                  2207    eukaryota>metazoa>chordata>vertebrata                   Mus musculus                                                    mediator of RNA polymerase II transcription subunit 13-like [Mus musculus].     291042494       -                       2207    eukaryota>metazoa>chordata>vertebrata                   Rattus norvegicus                                               thyroid hormone receptor-associated 240-like protein [Rattus norvegicus].     224071919       LOC100226614            2206    eukaryota>metazoa>chordata>vertebrata                   Taeniopygia guttata                                             PREDICTED: mediator complex subunit 13-like [Taeniopygia guttata].     38036201        -                       2203    eukaryota>metazoa>chordata>vertebrata                   Mus musculus                                                    thyroid hormone receptor-associated protein complex 240 kDa component-like [Mus musculus].     126324384       MED13L                  2201    eukaryota>metazoa>chordata>vertebrata                   Monodelphis domestica                                           PREDICTED: mediator complex subunit 13-like isoform 1 [Monodelphis domestica].     397525010       MED13L                  2200    eukaryota>metazoa>chordata>vertebrata                   Pan paniscus                                                    PREDICTED: mediator of RNA polymerase II transcription subunit 13-like [Pan paniscus].     332250746       MED13L                  2198    eukaryota>metazoa>chordata>vertebrata                   Nomascus leucogenys                                             PREDICTED: mediator of RNA polymerase II transcription subunit 13 [Nomascus leucogenys].     395744919       MED13L                  2198    eukaryota>metazoa>chordata>vertebrata                   Pongo abelii                                                    PREDICTED: mediator complex subunit 13-like [Pongo abelii].     301619721       LOC100491622            2197    eukaryota>metazoa>chordata>vertebrata                   Xenopus (Silurana) tropicalis                                   PREDICTED: mediator of RNA polymerase II transcription subunit 13-like [Xenopus (Silurana) tropicalis].     326931507       LOC100551484            2197    eukaryota>metazoa>chordata>vertebrata                   Meleagris gallopavo                                             PREDICTED: mediator of RNA polymerase II transcription subunit 13-like [Meleagris gallopavo].     395531834       MED13                   2197    eukaryota>metazoa>chordata>vertebrata                   Sarcophilus harrisii                                            PREDICTED: mediator of RNA polymerase II transcription subunit 13 [Sarcophilus harrisii].     291405670       LOC100337925            2196    eukaryota>metazoa>chordata>vertebrata                   Oryctolagus cuniculus                                           PREDICTED: mediator complex subunit 13 [Oryctolagus cuniculus].     392332681       Med13l                  2196    eukaryota>metazoa>chordata>vertebrata                   Rattus norvegicus                                               PREDICTED: mediator complex subunit 13-like [Rattus norvegicus].     118098868       MED13L                  2195    eukaryota>metazoa>chordata>vertebrata                   Gallus gallus                                                   PREDICTED: mediator complex subunit 13-like [Gallus gallus].     345483019       LOC100120623            2194    eukaryota>metazoa>hexapoda                              Nasonia vitripennis                                             PREDICTED: mediator of RNA polymerase II transcription subunit 13-like isoform 1 [Nasonia vitripennis].     345791178       MED13L                  2194    eukaryota>metazoa>chordata>vertebrata                   Canis lupus familiaris                                          PREDICTED: mediator complex subunit 13-like [Canis lupus familiaris].     296213025       MED13L                  2191    eukaryota>metazoa>chordata>vertebrata                   Callithrix jacchus                                              PREDICTED: mediator of RNA polymerase II transcription subunit 13-like [Callithrix jacchus].     149720623       MED13L                  2190    eukaryota>metazoa>chordata>vertebrata                   Equus caballus                                                  PREDICTED: mediator complex subunit 13-like [Equus caballus].     291407023       LOC100342832            2190    eukaryota>metazoa>chordata>vertebrata                   Oryctolagus cuniculus                                           PREDICTED: mediator complex subunit 13-like [Oryctolagus cuniculus].     348584428       LOC100728565            2190    eukaryota>metazoa>chordata>vertebrata                   Cavia porcellus                                                 PREDICTED: mediator of RNA polymerase II transcription subunit 13-like [Cavia porcellus].     355564716       EGK_04229               2190    eukaryota>metazoa>chordata>vertebrata                   Macaca mulatta                                                  hypothetical protein EGK_04229, partial [Macaca mulatta].     355786565       EGM_03797               2190    eukaryota>metazoa>chordata>vertebrata                   Macaca fascicularis                                             hypothetical protein EGM_03797, partial [Macaca fascicularis].     114669769       MED13                   2188    eukaryota>metazoa>chordata>vertebrata                   Pan troglodytes                                                 PREDICTED: mediator of RNA polymerase II transcription subunit 13 isoform 1 [Pan troglodytes].     354467014       Med13l                  2188    eukaryota>metazoa>chordata>vertebrata                   Cricetulus griseus                                              PREDICTED: mediator of RNA polymerase II transcription subunit 13 [Cricetulus griseus].     327276156       LOC100563224            2186    eukaryota>metazoa>chordata>vertebrata                   Anolis carolinensis                                             PREDICTED: LOW QUALITY PROTEIN: mediator of RNA polymerase II transcription subunit 13-like [Anolis carolinensis].     363741191       MED13                   2181    eukaryota>metazoa>chordata>vertebrata                   Gallus gallus                                                   PREDICTED: mediator of RNA polymerase II transcription subunit 13 [Gallus gallus].     224076629       LOC100223296            2179    eukaryota>metazoa>chordata>vertebrata                   Taeniopygia guttata                                             PREDICTED: mediator complex subunit 13 [Taeniopygia guttata].     4530437         -                       2174    eukaryota>metazoa>chordata>vertebrata                   Homo sapiens                                                    thyroid hormone receptor-associated protein complex component TRAP240 [Homo sapiens].     102468717       MED13                   2174    eukaryota>metazoa>chordata>vertebrata                   Homo sapiens                                                    mediator of RNA polymerase II transcription subunit 13 [Homo sapiens].     296201893       MED13                   2174    eukaryota>metazoa>chordata>vertebrata                   Callithrix jacchus                                              PREDICTED: mediator of RNA polymerase II transcription subunit 13 [Callithrix jacchus].     380786745       MED13                   2174    eukaryota>metazoa>chordata>vertebrata                   Macaca mulatta                                                  mediator of RNA polymerase II transcription subunit 13 [Macaca mulatta].     383423397       MED13                   2174    eukaryota>metazoa>chordata>vertebrata                   Macaca mulatta                                                  mediator of RNA polymerase II transcription subunit 13 [Macaca mulatta].     397486826       MED13                   2174    eukaryota>metazoa>chordata>vertebrata                   Pan paniscus                                                    PREDICTED: mediator of RNA polymerase II transcription subunit 13 [Pan paniscus].     402899848       MED13                   2174    eukaryota>metazoa>chordata>vertebrata                   Papio anubis                                                    PREDICTED: mediator of RNA polymerase II transcription subunit 13 [Papio anubis].     410216582       MED13                   2174    eukaryota>metazoa>chordata>vertebrata                   Pan troglodytes                                                 mediator complex subunit 13 [Pan troglodytes].     126307412       LOC100010182            2172    eukaryota>metazoa>chordata>vertebrata                   Monodelphis domestica                                           PREDICTED: mediator of RNA polymerase II transcription subunit 13-like [Monodelphis domestica].     124286862       Med13                   2171    eukaryota>metazoa>chordata>vertebrata                   Mus musculus                                                    mediator of RNA polymerase II transcription subunit 13 [Mus musculus].     301777948       LOC100464926            2171    eukaryota>metazoa>chordata>vertebrata                   Ailuropoda melanoleuca                                          PREDICTED: mediator of RNA polymerase II transcription subunit 13-like [Ailuropoda melanoleuca].     345324850       MED13                   2169    eukaryota>metazoa>chordata>vertebrata                   Ornithorhynchus anatinus                                        PREDICTED: mediator of RNA polymerase II transcription subunit 13, partial [Ornithorhynchus anatinus].     410914654       LOC101070072            2166    eukaryota>metazoa>chordata>vertebrata>actinopterygii    Takifugu rubripes                                               PREDICTED: mediator of RNA polymerase II transcription subunit 13-like [Takifugu rubripes].     194217212       MED13                   2165    eukaryota>metazoa>chordata>vertebrata                   Equus caballus                                                  PREDICTED: mediator of RNA polymerase II transcription subunit 13 [Equus caballus].     297486414       MED13                   2157    eukaryota>metazoa>chordata>vertebrata                   Bos taurus                                                      PREDICTED: mediator of RNA polymerase II transcription subunit 13 [Bos taurus].     332258930       MED13                   2157    eukaryota>metazoa>chordata>vertebrata                   Nomascus leucogenys                                             PREDICTED: mediator of RNA polymerase II transcription subunit 13, partial [Nomascus leucogenys].     354497406       Med13                   2155    eukaryota>metazoa>chordata>vertebrata                   Cricetulus griseus                                              PREDICTED: mediator of RNA polymerase II transcription subunit 13 [Cricetulus griseus].     355568592       EGK_08602               2155    eukaryota>metazoa>chordata>vertebrata                   Macaca mulatta                                                  hypothetical protein EGK_08602, partial [Macaca mulatta].     355754062       EGM_07789               2155    eukaryota>metazoa>chordata>vertebrata                   Macaca fascicularis                                             hypothetical protein EGM_07789, partial [Macaca fascicularis].     395845983       MED13                   2155    eukaryota>metazoa>chordata>vertebrata                   Otolemur garnettii                                              PREDICTED: mediator of RNA polymerase II transcription subunit 13 [Otolemur garnettii].     351714843       GW7_08306               2153    eukaryota>metazoa>chordata>vertebrata                   Heterocephalus glaber                                           Mediator of RNA polymerase II transcription subunit 13, partial [Heterocephalus glaber].     348535272       LOC100702662            2122    eukaryota>metazoa>chordata>vertebrata>actinopterygii    Oreochromis niloticus                                           PREDICTED: mediator of RNA polymerase II transcription subunit 13-like [Oreochromis niloticus].     328720163       LOC100163445            2121    eukaryota>metazoa>hexapoda                              Acyrthosiphon pisum                                             PREDICTED: mediator of RNA polymerase II transcription subunit 13-like [Acyrthosiphon pisum].     321460432       DAPPUDRAFT_60138        2118    eukaryota>metazoa>crustacea                             Daphnia pulex                                                   hypothetical protein DAPPUDRAFT_60138 [Daphnia pulex].     242008648       Phum_PHUM174820         2117    eukaryota>metazoa>hexapoda                              Pediculus humanus corporis                                      Thyroid hormone receptor-associated protein, putative [Pediculus humanus corporis].     344285341       MED13                   2117    eukaryota>metazoa>chordata>vertebrata                   Loxodonta africana                                              PREDICTED: mediator of RNA polymerase II transcription subunit 13 [Loxodonta africana].     297715614       MED13                   2115    eukaryota>metazoa>chordata>vertebrata                   Pongo abelii                                                    PREDICTED: mediator of RNA polymerase II transcription subunit 13 [Pongo abelii].     348536266       LOC100700595            2114    eukaryota>metazoa>chordata>vertebrata>actinopterygii    Oreochromis niloticus                                           PREDICTED: mediator of RNA polymerase II transcription subunit 13-like [Oreochromis niloticus].     351694755       GW7_10169               2106    eukaryota>metazoa>chordata>vertebrata                   Heterocephalus glaber                                           Mediator of RNA polymerase II transcription subunit 13-like protein, partial [Heterocephalus glaber].     410910158       LOC101071213            2106    eukaryota>metazoa>chordata>vertebrata>actinopterygii    Takifugu rubripes                                               PREDICTED: mediator of RNA polymerase II transcription subunit 13-like [Takifugu rubripes].     139948785       med13b                  2102    eukaryota>metazoa>chordata>vertebrata>actinopterygii    Danio rerio                                                     mediator of RNA polymerase II transcription subunit 13-like [Danio rerio].     301608632       LOC100490132            2101    eukaryota>metazoa>chordata>vertebrata                   Xenopus (Silurana) tropicalis                                   PREDICTED: mediator of RNA polymerase II transcription subunit 13-like [Xenopus (Silurana) tropicalis].     350592501       MED13L                  2075    eukaryota>metazoa>chordata>vertebrata                   Sus scrofa                                                      PREDICTED: mediator complex subunit 13-like [Sus scrofa].     345483021       LOC100120623            2070    eukaryota>metazoa>hexapoda                              Nasonia vitripennis                                             PREDICTED: mediator of RNA polymerase II transcription subunit 13-like isoform 2 [Nasonia vitripennis].     51476392        DKFZp781D0112           2069    eukaryota>metazoa>chordata>vertebrata                   Homo sapiens                                                    hypothetical protein, partial [Homo sapiens].     395513911       MED13L                  2063    eukaryota>metazoa>chordata>vertebrata                   Sarcophilus harrisii                                            PREDICTED: mediator of RNA polymerase II transcription subunit 13-like [Sarcophilus harrisii].     148687845       mCG_124529              2050    eukaryota>metazoa>chordata>vertebrata                   Mus musculus                                                    thyroid hormone receptor associated protein 2 [Mus musculus].     157820255       Med13                   2040    eukaryota>metazoa>chordata>vertebrata                   Rattus norvegicus                                               mediator of RNA polymerase II transcription subunit 13 [Rattus norvegicus].     307209243       EAI_15618               2020    eukaryota>metazoa>hexapoda                              Harpegnathos saltator                                           Mediator of RNA polymerase II transcription subunit 13, partial [Harpegnathos saltator].     410904265       LOC101064404            2018    eukaryota>metazoa>chordata>vertebrata>actinopterygii    Takifugu rubripes                                               PREDICTED: mediator of RNA polymerase II transcription subunit 13-like, partial [Takifugu rubripes].     405976314       CGI_10026509            2015    eukaryota>metazoa>mollusca                              Crassostrea gigas                                               Mediator of RNA polymerase II transcription subunit 13 [Crassostrea gigas].     340717957       LOC100642571            1999    eukaryota>metazoa>hexapoda                              Bombus terrestris                                               PREDICTED: mediator of RNA polymerase II transcription subunit 13-like isoform 2 [Bombus terrestris].     350400120       LOC100742855            1999    eukaryota>metazoa>hexapoda                              Bombus impatiens                                                PREDICTED: mediator of RNA polymerase II transcription subunit 13-like isoform 2 [Bombus impatiens].     380019945       LOC100870386            1995    eukaryota>metazoa>hexapoda                              Apis florea                                                     PREDICTED: mediator of RNA polymerase II transcription subunit 13-like [Apis florea].     328790302       LOC410159               1994    eukaryota>metazoa>hexapoda                              Apis mellifera                                                  PREDICTED: mediator of RNA polymerase II transcription subunit 13-like [Apis mellifera].     383854209       LOC100880003            1994    eukaryota>metazoa>hexapoda                              Megachile rotundata                                             PREDICTED: mediator of RNA polymerase II transcription subunit 13-like [Megachile rotundata].     47223584        GSTEN:00017185:G:001    1986    eukaryota>metazoa>chordata>vertebrata>actinopterygii    Tetraodon nigroviridis                                          unnamed protein product, partial [Tetraodon nigroviridis].     410980596       MED13                   1974    eukaryota>metazoa>chordata>vertebrata                   Felis catus                                                     PREDICTED: mediator of RNA polymerase II transcription subunit 13 [Felis catus].     340717955       LOC100642571            1938    eukaryota>metazoa>hexapoda                              Bombus terrestris                                               PREDICTED: mediator of RNA polymerase II transcription subunit 13-like isoform 1 [Bombus terrestris].     350400114       LOC100742855            1938    eukaryota>metazoa>hexapoda                              Bombus impatiens                                                PREDICTED: mediator of RNA polymerase II transcription subunit 13-like isoform 1 [Bombus impatiens].     410976768       MED13L                  1924    eukaryota>metazoa>chordata>vertebrata                   Felis catus                                                     PREDICTED: mediator of RNA polymerase II transcription subunit 13-like [Felis catus].     20521734        KIAA1025                1917    eukaryota>metazoa>chordata>vertebrata                   Homo sapiens                                                    KIAA1025 protein, partial [Homo sapiens].     332017084       G5I_14070               1903    eukaryota>metazoa>hexapoda                              Acromyrmex echinatior                                           Mediator of RNA polymerase II transcription subunit 13, partial [Acromyrmex echinatior].     357625948       KGM_17578               1884    eukaryota>metazoa>hexapoda                              Danaus plexippus                                                hypothetical protein KGM_17578 [Danaus plexippus].     49257394        Med13l                  1879    eukaryota>metazoa>chordata>vertebrata                   Mus musculus                                                    Med13l protein, partial [Mus musculus].     391342315       LOC100901356            1872    eukaryota>metazoa                                       Metaseiulus occidentalis                                        PREDICTED: mediator of RNA polymerase II transcription subunit 13-like [Metaseiulus occidentalis].     198415621       LOC100179760            1817    eukaryota>metazoa>chordata                              Ciona intestinalis                                              PREDICTED: similar to mediator complex subunit 13, partial [Ciona intestinalis].     350590574       MED13                   1806    eukaryota>metazoa>chordata>vertebrata                   Sus scrofa                                                      PREDICTED: mediator of RNA polymerase II transcription subunit 13 [Sus scrofa].     307189226       EAG_08471               1799    eukaryota>metazoa>hexapoda                              Camponotus floridanus                                           Mediator of RNA polymerase II transcription subunit 13, partial [Camponotus floridanus].     322784983       SINV_12591              1778    eukaryota>metazoa>hexapoda                              Solenopsis invicta                                              hypothetical protein SINV_12591, partial [Solenopsis invicta].     28972570        mKIAA1025               1658    eukaryota>metazoa>chordata>vertebrata                   Mus musculus                                                    mKIAA1025 protein, partial [Mus musculus].     291231370       LOC100378573            1501    eukaryota>metazoa>hemichordata                          Saccoglossus kowalevskii                                        PREDICTED: skuld-like [Saccoglossus kowalevskii].     260820746       BRAFLDRAFT_279958       1251    eukaryota>metazoa>chordata                              Branchiostoma floridae                                          hypothetical protein BRAFLDRAFT_279958 [Branchiostoma floridae].     260820706       BRAFLDRAFT_264574       1245    eukaryota>metazoa>chordata                              Branchiostoma floridae                                          hypothetical protein BRAFLDRAFT_264574 [Branchiostoma floridae].     390361438       med13l                  1129    eukaryota>metazoa>echinodermata                         Strongylocentrotus purpuratus                                   PREDICTED: mediator of RNA polymerase II transcription subunit 13 [Strongylocentrotus purpuratus].     10435895        -                       1111    eukaryota>metazoa>chordata>vertebrata                   Homo sapiens                                                    unnamed protein product [Homo sapiens].     170595964       Bm1_55650               910     eukaryota>metazoa>nematoda                              Brugia malayi                                                   Thyroid hormone receptor-associated protein complex 240 kDa component [Brugia malayi].     355701728       -                       816     eukaryota>metazoa>chordata>vertebrata                   Mustela putorius furo                                           mediator complex subunit 13-like protein, partial [Mustela putorius furo].     37360000        mKIAA0593               731     eukaryota>metazoa>chordata>vertebrata                   Mus musculus                                                    mKIAA0593 protein, partial [Mus musculus].     74206709        -                       525     eukaryota>metazoa>chordata>vertebrata                   Mus musculus                                                    unnamed protein product, partial [Mus musculus].     402592247       WUBG_02916              407     eukaryota>metazoa>nematoda                              Wuchereria bancrofti                                            hypothetical protein WUBG_02916, partial [Wuchereria bancrofti].     355701713       -                       373     eukaryota>metazoa>chordata>vertebrata                   Mustela putorius furo                                           mediator complex subunit 13, partial [Mustela putorius furo].     47195883        GSTEN:00002683:G:001    278     eukaryota>metazoa>chordata>vertebrata>actinopterygii    Tetraodon nigroviridis                                          unnamed protein product, partial [Tetraodon nigroviridis].     341881130       CAEBREN_25284           3087    eukaryota>metazoa>nematoda                              Caenorhabditis brenneri                                         CBN-LET-19 protein [Caenorhabditis brenneri].     341879129       CAEBREN_23274           3047    eukaryota>metazoa>nematoda                              Caenorhabditis brenneri                                         hypothetical protein CAEBREN_23274 [Caenorhabditis brenneri].     269849618       -                       2974    eukaryota>metazoa>nematoda                              Caenorhabditis briggsae                                         RecName: Full=Mediator of RNA polymerase II transcription subunit 13; AltName: Full=Lethal protein 19; AltName: Full=Mediator complex subunit 13.     308510662       CRE_01872               2923    eukaryota>metazoa>nematoda                              Caenorhabditis remanei                                          CRE-LET-19 protein [Caenorhabditis remanei].     268532428       CBG03168                2898    eukaryota>metazoa>nematoda                              Caenorhabditis briggsae                                         C. briggsae CBR-LET-19 protein, partial [Caenorhabditis briggsae].     71992166        CELE_K08F8.6            2862    eukaryota>metazoa>nematoda                              Caenorhabditis elegans                                          Protein LET-19 [Caenorhabditis elegans].     324499641       -                       2659    eukaryota>metazoa>nematoda                              Ascaris suum                                                    Mediator of RNA polymerase II transcription subunit 13 [Ascaris suum].     312065604       LOAG_00283              2646    eukaryota>metazoa>nematoda                              Loa loa                                                         hypothetical protein LOAG_00283 [Loa loa].     256079566       Smp_147560              2353    eukaryota>metazoa                                       Schistosoma mansoni                                             hypothetical protein [Schistosoma mansoni].     358339524       CLF_100529              2165    eukaryota>metazoa                                       Clonorchis sinensis                                             mediator of RNA polymerase II transcription subunit 13-like [Clonorchis sinensis].     189240457       LOC662491               2011    eukaryota>metazoa>hexapoda                              Tribolium castaneum                                             PREDICTED: similar to conserved hypothetical protein [Tribolium castaneum].     270011427       TcasGA2_TC005449        1973    eukaryota>metazoa>hexapoda                              Tribolium castaneum                                             hypothetical protein TcasGA2_TC005449 [Tribolium castaneum].     402583804       WUBG_11343              463     eukaryota>metazoa>nematoda                              Wuchereria bancrofti                                            hypothetical protein WUBG_11343 [Wuchereria bancrofti].     339244989       Tsp_06327               1918    eukaryota>metazoa>nematoda                              Trichinella spiralis                                            TRAP240 family protein [Trichinella spiralis].     221129420       LOC100203963            1014    eukaryota>metazoa>cnidaria                              Hydra magnipapillata                                            PREDICTED: similar to mediator complex subunit 13-like [Hydra magnipapillata].     156338015       NEMVEDRAFT_v1g223651    510     eukaryota>metazoa>cnidaria                              Nematostella vectensis                                          hypothetical protein NEMVEDRAFT_v1g223651, partial [Nematostella vectensis].     47216840        GSTEN:00021909:G:001    2163    eukaryota>metazoa>chordata>vertebrata>actinopterygii    Tetraodon nigroviridis                                          unnamed protein product, partial [Tetraodon nigroviridis].     345321772       LOC100074327            1547    eukaryota>metazoa>chordata>vertebrata                   Ornithorhynchus anatinus                                        PREDICTED: mediator of RNA polymerase II transcription subunit 13-like, partial [Ornithorhynchus anatinus].     355701710       -                       386     eukaryota>metazoa>chordata>vertebrata                   Mustela putorius furo                                           mediator complex subunit 13, partial [Mustela putorius furo].     355701725       -                       204     eukaryota>metazoa>chordata>vertebrata                   Mustela putorius furo                                           mediator complex subunit 13-like protein, partial [Mustela putorius furo].     344242806       I79_019144              1336    eukaryota>metazoa>chordata>vertebrata                   Cricetulus griseus                                              Mediator of RNA polymerase II transcription subunit 13 [Cricetulus griseus].     327285202       LOC100567482            1450    eukaryota>metazoa>chordata>vertebrata                   Anolis carolinensis                                             PREDICTED: mediator of RNA polymerase II transcription subunit 13-like [Anolis carolinensis].     313239429       GSOID_T00007359001      790     eukaryota>metazoa>chordata                              Oikopleura dioica                                               unnamed protein product [Oikopleura dioica].     241713105       IscW_ISCW022893         2155    eukaryota>metazoa                                       Ixodes scapularis                                               hypothetical protein IscW_ISCW022893, partial [Ixodes scapularis].     340376395       LOC100634857            435     eukaryota>metazoa                                       Amphimedon queenslandica                                        PREDICTED: mediator of RNA polymerase II transcription subunit 13-like [Amphimedon queenslandica].     #;Med13_N+fungi-specific+linker+MID+MedPIWI     400597912       BBA_05505               1843    eukaryota>fungi>ascomycota                              Beauveria bassiana ARSEF 2860                                   Subunit of the RNA polymerase II mediator complex [Beauveria bassiana ARSEF 2860].     407918219       MPH_11380               1764    eukaryota>fungi>ascomycota                              Macrophomina phaseolina MS6                                     Mediator complex subunit Med13 [Macrophomina phaseolina MS6].     189201233       PTRG_06620              1675    eukaryota>fungi>ascomycota                              Pyrenophora tritici-repentis Pt-1C-BFP                          conserved hypothetical protein [Pyrenophora tritici-repentis Pt-1C-BFP].     336466369       NEUTE1DRAFT_148840      1670    eukaryota>fungi>ascomycota                              Neurospora tetrasperma FGSC 2508                                hypothetical protein NEUTE1DRAFT_148840 [Neurospora tetrasperma FGSC 2508].     330945209       PTT_19672               1644    eukaryota>fungi>ascomycota                              Pyrenophora teres f. teres 0-1                                  hypothetical protein PTT_19672 [Pyrenophora teres f. teres 0-1].     398398996       MYCGRDRAFT_109073       1631    eukaryota>fungi>ascomycota                              Zymoseptoria tritici IPO323                                     hypothetical protein MYCGRDRAFT_109073 [Zymoseptoria tritici IPO323].     396475444       LEMA_P112280.1          1618    eukaryota>fungi>ascomycota                              Leptosphaeria maculans JN3                                      hypothetical protein LEMA_P112280.1 [Leptosphaeria maculans JN3].     380089044       SMAC_06131              1553    eukaryota>fungi>ascomycota                              Sordaria macrospora k-hell                                      unnamed protein product [Sordaria macrospora k-hell].     408394961       FPSE_05654              1534    eukaryota>fungi>ascomycota                              Fusarium pseudograminearum CS3096                               hypothetical protein FPSE_05654 [Fusarium pseudograminearum CS3096].     46128121        FG08438.1               1533    eukaryota>fungi>ascomycota                              Gibberella zeae PH-1                                            hypothetical protein FG08438.1 [Gibberella zeae PH-1].     225681214       PABG_01817              1532    eukaryota>fungi>ascomycota                              Paracoccidioides brasiliensis Pb03                              conserved hypothetical protein [Paracoccidioides brasiliensis Pb03].     367029541       MYCTH_69563             1532    eukaryota>fungi>ascomycota                              Myceliophthora thermophila ATCC 42464                           hypothetical protein MYCTH_69563 [Myceliophthora thermophila ATCC 42464].     327348676       BDDG_00470              1531    eukaryota>fungi>ascomycota                              Ajellomyces dermatitidis ATCC 18188                             hypothetical protein BDDG_00470 [Ajellomyces dermatitidis ATCC 18188].     347839995       BofuT4_P126150.1        1531    eukaryota>fungi>ascomycota                              Botryotinia fuckeliana                                          hypothetical protein [Botryotinia fuckeliana].     336260101       SMAC_06131              1528    eukaryota>fungi>ascomycota                              Sordaria macrospora k-hell                                      hypothetical protein SMAC_06131 [Sordaria macrospora k-hell].     156060191       SS1G_02234              1527    eukaryota>fungi>ascomycota                              Sclerotinia sclerotiorum 1980 UF-70                             hypothetical protein SS1G_02234 [Sclerotinia sclerotiorum 1980 UF-70].     225557414       HCBG_05964              1527    eukaryota>fungi>ascomycota                              Ajellomyces capsulatus G186AR                                   conserved hypothetical protein [Ajellomyces capsulatus G186AR].     240278051       HCDG_04205              1527    eukaryota>fungi>ascomycota                              Ajellomyces capsulatus H143                                     conserved hypothetical protein [Ajellomyces capsulatus H143].     325096116       HCEG_08641              1527    eukaryota>fungi>ascomycota                              Ajellomyces capsulatus H88                                      conserved hypothetical protein [Ajellomyces capsulatus H88].     389638856       MGG_06499               1523    eukaryota>fungi>ascomycota                              Magnaporthe oryzae 70-15                                        hypothetical protein MGG_06499 [Magnaporthe oryzae 70-15].     226292071       PADG_03575              1521    eukaryota>fungi>ascomycota                              Paracoccidioides brasiliensis Pb18                              conserved hypothetical protein [Paracoccidioides brasiliensis Pb18].     402085989       GGTG_00880              1518    eukaryota>fungi>ascomycota                              Gaeumannomyces graminis var. tritici R3-111a-1                  hypothetical protein GGTG_00880 [Gaeumannomyces graminis var. tritici R3-111a-1].     212529140       PMAA_030290             1516    eukaryota>fungi>ascomycota                              Penicillium marneffei ATCC 18224                                conserved hypothetical protein [Penicillium marneffei ATCC 18224].     310794822       GLRG_05427              1515    eukaryota>fungi>ascomycota                              Glomerella graminicola M1.001                                   hypothetical protein GLRG_05427 [Glomerella graminicola M1.001].     320588584       CMQ_5994                1515    eukaryota>fungi>ascomycota                              Grosmannia clavigera kw1407                                     hypothetical protein CMQ_5994 [Grosmannia clavigera kw1407].     406862209       MBM_06476               1513    eukaryota>fungi>ascomycota                              Marssonina brunnea f. sp. 'multigermtubi' MB_m1                 putative Mediator of RNA polymerase II transcription subunit 13 [Marssonina brunnea f. sp. 'multigermtubi' MB_m1].     322698707       MAC_03469               1507    eukaryota>fungi>ascomycota                              Metarhizium acridum CQMa 102                                    hypothetical protein MAC_03469 [Metarhizium acridum CQMa 102].     322711079       MAA_02235               1507    eukaryota>fungi>ascomycota                              Metarhizium anisopliae ARSEF 23                                 hypothetical protein MAA_02235 [Metarhizium anisopliae ARSEF 23].     367040017       THITE_135495            1502    eukaryota>fungi>ascomycota                              Thielavia terrestris NRRL 8126                                  hypothetical protein THITE_135495 [Thielavia terrestris NRRL 8126].     116202613       CHGG_09191              1500    eukaryota>fungi>ascomycota                              Chaetomium globosum CBS 148.51                                  hypothetical protein CHGG_09191 [Chaetomium globosum CBS 148.51].     242764366       TSTA_067970             1498    eukaryota>fungi>ascomycota                              Talaromyces stipitatus ATCC 10500                               conserved hypothetical protein [Talaromyces stipitatus ATCC 10500].     119480151       NFIA_081530             1497    eukaryota>fungi>ascomycota                              Neosartorya fischeri NRRL 181                                   hypothetical protein NFIA_081530 [Neosartorya fischeri NRRL 181].     119193684       CIMG_01219              1495    eukaryota>fungi>ascomycota                              Coccidioides immitis RS                                         hypothetical protein CIMG_01219 [Coccidioides immitis RS].     303311871       CPC735_051720           1495    eukaryota>fungi>ascomycota                              Coccidioides posadasii C735 delta SOWgp                         hypothetical protein CPC735_051720 [Coccidioides posadasii C735 delta SOWgp].     317143452       AOR_1_482154            1495    eukaryota>fungi>ascomycota                              Aspergillus oryzae RIB40                                        hypothetical protein AOR_1_482154 [Aspergillus oryzae RIB40].     342879481       FOXB_08768              1495    eukaryota>fungi>ascomycota                              Fusarium oxysporum Fo5176                                       hypothetical protein FOXB_08768 [Fusarium oxysporum Fo5176].     391864049       Ao3042_10678            1495    eukaryota>fungi>ascomycota                              Aspergillus oryzae 3.042                                        hypothetical protein Ao3042_10678 [Aspergillus oryzae 3.042].     346970926       VDAG_05542              1489    eukaryota>fungi>ascomycota                              Verticillium dahliae VdLs.17                                    hypothetical protein VDAG_05542 [Verticillium dahliae VdLs.17].     255932893       Pc12g10970              1488    eukaryota>fungi>ascomycota                              Penicillium chrysogenum Wisconsin 54-1255                       Pc12g10970 [Penicillium chrysogenum Wisconsin 54-1255].     346323556       CCM_04526               1488    eukaryota>fungi>ascomycota                              Cordyceps militaris CM01                                        hypothetical protein CCM_04526 [Cordyceps militaris CM01].     340517015       TRIREDRAFT_22783        1486    eukaryota>fungi>ascomycota                              Trichoderma reesei QM6a                                         predicted protein [Trichoderma reesei QM6a].     115399220       ATEG_06021              1485    eukaryota>fungi>ascomycota                              Aspergillus terreus NIH2624                                     conserved hypothetical protein [Aspergillus terreus NIH2624].     340939292       CTHT_0044070            1485    eukaryota>fungi>ascomycota                              Chaetomium thermophilum var. thermophilum DSM 1495              hypothetical protein CTHT_0044070 [Chaetomium thermophilum var. thermophilum DSM 1495].     295673690       PAAG_01250              1482    eukaryota>fungi>ascomycota                              Paracoccidioides sp. 'lutzii' Pb01                              conserved hypothetical protein [Paracoccidioides sp. 'lutzii' Pb01].     317032822       ANI_1_1956094           1479    eukaryota>fungi>ascomycota                              Aspergillus niger CBS 513.88                                    hypothetical protein ANI_1_1956094 [Aspergillus niger CBS 513.88].     358367197       AKAW_01931              1479    eukaryota>fungi>ascomycota                              Aspergillus kawachii IFO 4308                                   hypothetical protein AKAW_01931 [Aspergillus kawachii IFO 4308].     320039898       CPSG_01989              1478    eukaryota>fungi>ascomycota                              Coccidioides posadasii str. Silveira                            conserved hypothetical protein [Coccidioides posadasii str. Silveira].     327302560       TERG_03025              1469    eukaryota>fungi>ascomycota                              Trichophyton rubrum CBS 118892                                  hypothetical protein TERG_03025 [Trichophyton rubrum CBS 118892].     315040840       MGYG_07965              1468    eukaryota>fungi>ascomycota                              Arthroderma gypseum CBS 118893                                  hypothetical protein MGYG_07965 [Arthroderma gypseum CBS 118893].     83767347        AO090003000283          1467    eukaryota>fungi>ascomycota                              Aspergillus oryzae RIB40                                        unnamed protein product [Aspergillus oryzae RIB40].     67523243        AN2078.2                1458    eukaryota>fungi>ascomycota                              Aspergillus nidulans FGSC A4                                    hypothetical protein AN2078.2 [Aspergillus nidulans FGSC A4].     85085914        NCU03962                1458    eukaryota>fungi>ascomycota                              Neurospora crassa OR74A                                         hypothetical protein NCU03962 [Neurospora crassa OR74A].     326471082       TESG_02585              1458    eukaryota>fungi>ascomycota                              Trichophyton tonsurans CBS 112818                               hypothetical protein TESG_02585, partial [Trichophyton tonsurans CBS 112818].     134079124       An11g04480              1457    eukaryota>fungi>ascomycota                              Aspergillus niger                                               unnamed protein product [Aspergillus niger].     302510044       ARB_05276               1443    eukaryota>fungi>ascomycota                              Arthroderma benhamiae CBS 112371                                conserved hypothetical protein [Arthroderma benhamiae CBS 112371].     302663861       TRV_02315               1439    eukaryota>fungi>ascomycota                              Trichophyton verrucosum HKI 0517                                conserved hypothetical protein [Trichophyton verrucosum HKI 0517].     296813681       MCYG_04915              1435    eukaryota>fungi>ascomycota                              Arthroderma otae CBS 113480                                     conserved hypothetical protein [Arthroderma otae CBS 113480].     358386223       TRIVIDRAFT_112408       1434    eukaryota>fungi>ascomycota                              Trichoderma virens Gv29-8                                       hypothetical protein TRIVIDRAFT_112408, partial [Trichoderma virens Gv29-8].     121710210       ACLA_089870             1431    eukaryota>fungi>ascomycota                              Aspergillus clavatus NRRL 1                                     conserved hypothetical protein [Aspergillus clavatus NRRL 1].     70989521        AFUA_2G04790            1429    eukaryota>fungi>ascomycota                              Aspergillus fumigatus Af293                                     conserved hypothetical protein [Aspergillus fumigatus Af293].     159129017       AFUB_021830             1429    eukaryota>fungi>ascomycota                              Aspergillus fumigatus A1163                                     conserved hypothetical protein [Aspergillus fumigatus A1163].     302918258       NECHADRAFT_59159        1426    eukaryota>fungi>ascomycota                              Nectria haematococca mpVI 77-13-4                               hypothetical protein NECHADRAFT_59159 [Nectria haematococca mpVI 77-13-4].     239611315       BDCG_03422              1423    eukaryota>fungi>ascomycota                              Ajellomyces dermatitidis ER-3                                   conserved hypothetical protein [Ajellomyces dermatitidis ER-3].     261205472       BDBG_02144              1423    eukaryota>fungi>ascomycota                              Ajellomyces dermatitidis SLH14081                               conserved hypothetical protein [Ajellomyces dermatitidis SLH14081].     358394853       TRIATDRAFT_223394       1399    eukaryota>fungi>ascomycota                              Trichoderma atroviride IMI 206040                               hypothetical protein TRIATDRAFT_223394 [Trichoderma atroviride IMI 206040].     154274722       HCAG_05817              1396    eukaryota>fungi>ascomycota                              Ajellomyces capsulatus NAm1                                     predicted protein [Ajellomyces capsulatus NAm1].     378733479       HMPREF1120_07914        1380    eukaryota>fungi>ascomycota                              Exophiala dermatitidis NIH/UT8656                               hypothetical protein HMPREF1120_07914 [Exophiala dermatitidis NIH/UT8656].     350631249       ASPNIDRAFT_118893       1377    eukaryota>fungi>ascomycota                              Aspergillus niger ATCC 1015                                     hypothetical protein ASPNIDRAFT_118893, partial [Aspergillus niger ATCC 1015].     169618607       SNOG_12496              1375    eukaryota>fungi>ascomycota                              Phaeosphaeria nodorum SN15                                      hypothetical protein SNOG_12496 [Phaeosphaeria nodorum SN15].     326479766       TEQG_02810              1348    eukaryota>fungi>ascomycota                              Trichophyton equinum CBS 127.97                                 hypothetical protein TEQG_02810 [Trichophyton equinum CBS 127.97].     171682458       PODANSg3200             1333    eukaryota>fungi>ascomycota                              Podospora anserina S mat+                                       hypothetical protein, partial [Podospora anserina S mat+].     258574905       UREG_01150              1231    eukaryota>fungi>ascomycota                              Uncinocarpus reesii 1704                                        predicted protein [Uncinocarpus reesii 1704].     238487616       AFLA_035360             1073    eukaryota>fungi>ascomycota                              Aspergillus flavus NRRL3357                                     conserved hypothetical protein [Aspergillus flavus NRRL3357].     380480996       CH063_12197             918     eukaryota>fungi>ascomycota                              Colletotrichum higginsianum                                     hypothetical protein CH063_12197 [Colletotrichum higginsianum].     154315531       BC1G_04338              677     eukaryota>fungi>ascomycota                              Botryotinia fuckeliana B05.10                                   hypothetical protein BC1G_04338 [Botryotinia fuckeliana B05.10].     390600750       PUNSTDRAFT_142256       603     eukaryota>fungi>basidiomycota                           Punctularia strigosozonata HHB-11173 SS5                        hypothetical protein PUNSTDRAFT_142256 [Punctularia strigosozonata HHB-11173 SS5].     367001186       TPHA_0D02570            1487    eukaryota>fungi>ascomycota                              Tetrapisispora phaffii CBS 4417                                 hypothetical protein TPHA_0D02570 [Tetrapisispora phaffii CBS 4417].     403214916       KNAG_0C03050            1449    eukaryota>fungi>ascomycota                              Kazachstania naganishii CBS 8797                                hypothetical protein KNAG_0C03050 [Kazachstania naganishii CBS 8797].     401837777       SKUD_189403             1424    eukaryota>fungi>ascomycota                              Saccharomyces kudriavzevii IFO 1802                             SSN2-like protein [Saccharomyces kudriavzevii IFO 1802].     495490          SCA1                    1420    eukaryota>fungi>ascomycota                              Saccharomyces cerevisiae                                        Sca1p [Saccharomyces cerevisiae].     151942409       SCY_1324                1420    eukaryota>fungi>ascomycota                              Saccharomyces cerevisiae YJM789                                 mediator complex subunit [Saccharomyces cerevisiae YJM789].     256270662       C1Q_03776               1420    eukaryota>fungi>ascomycota                              Saccharomyces cerevisiae JAY291                                 Ssn2p [Saccharomyces cerevisiae JAY291].     398366601       YDR443C                 1420    eukaryota>fungi>ascomycota                              Saccharomyces cerevisiae S288c                                  Ssn2p [Saccharomyces cerevisiae S288c].     190404629       SCRG_00095              1418    eukaryota>fungi>ascomycota                              Saccharomyces cerevisiae RM11-1a                                suppressor of RNA polymerase B SSN2 [Saccharomyces cerevisiae RM11-1a].     349577488       SYK7_016661             1418    eukaryota>fungi>ascomycota                              Saccharomyces cerevisiae Kyokai no. 7                           K7_Ssn2p [Saccharomyces cerevisiae Kyokai no. 7].     365766228       VIN7_1127               1418    eukaryota>fungi>ascomycota                              Saccharomyces cerevisiae x Saccharomyces kudriavzevii VIN7      Ssn2p [Saccharomyces cerevisiae x Saccharomyces kudriavzevii VIN7].     401624082       SU7_2759                1418    eukaryota>fungi>ascomycota                              Saccharomyces arboricola H-6                                    ssn2p [Saccharomyces arboricola H-6].     387513917       TBLA_0E03010            1409    eukaryota>fungi>ascomycota                              Tetrapisispora blattae CBS 6284                                 hypothetical protein TBLA_0E03010 [Tetrapisispora blattae CBS 6284].     365989526       NDAI_0H01760            1403    eukaryota>fungi>ascomycota                              Naumovozyma dairenensis CBS 421                                 hypothetical protein NDAI_0H01760 [Naumovozyma dairenensis CBS 421].     50303299        KLLA0B01353g            1387    eukaryota>fungi>ascomycota                              Kluyveromyces lactis NRRL Y-1140                                hypothetical protein [Kluyveromyces lactis NRRL Y-1140].     156844937       Kpol_1004p46            1375    eukaryota>fungi>ascomycota                              Vanderwaltozyma polyspora DSM 70294                             hypothetical protein Kpol_1004p46 [Vanderwaltozyma polyspora DSM 70294].     410082854       KAFR_0I00890            1372    eukaryota>fungi>ascomycota                              Kazachstania africana CBS 2517                                  hypothetical protein KAFR_0I00890 [Kazachstania africana CBS 2517].     254582050       ZYRO0D13266g            1360    eukaryota>fungi>ascomycota                              Zygosaccharomyces rouxii CBS 732                                ZYRO0D13266p [Zygosaccharomyces rouxii].     366994396       NCAS_0F01230            1360    eukaryota>fungi>ascomycota                              Naumovozyma castellii CBS 4309                                  hypothetical protein NCAS_0F01230 [Naumovozyma castellii CBS 4309].     363751547       Ecym_4094               1359    eukaryota>fungi>ascomycota                              Eremothecium cymbalariae DBVPG#7215                             hypothetical protein Ecym_4094 [Eremothecium cymbalariae DBVPG#7215].     45190925        AGOS_AER323W            1357    eukaryota>fungi>ascomycota                              Ashbya gossypii ATCC 10895                                      AER323Wp [Ashbya gossypii ATCC 10895].     255717903       KLTH0G04488g            1356    eukaryota>fungi>ascomycota                              Lachancea thermotolerans CBS 6340                               KLTH0G04488p [Lachancea thermotolerans].     50291469        CAGL0J10472g            1345    eukaryota>fungi>ascomycota                              Candida glabrata CBS 138                                        hypothetical protein [Candida glabrata CBS 138].     367008928       TDEL_0A04220            1334    eukaryota>fungi>ascomycota                              Torulaspora delbrueckii                                         hypothetical protein TDEL_0A04220 [Torulaspora delbrueckii].     294654361       DEHA2A01716g            1774    eukaryota>fungi>ascomycota                              Debaryomyces hansenii CBS767                                    DEHA2A01716p [Debaryomyces hansenii CBS767].     344233973       CANTEDRAFT_101756       1770    eukaryota>fungi>ascomycota                              Candida tenuis ATCC 10573                                       hypothetical protein CANTEDRAFT_101756 [Candida tenuis ATCC 10573].     359464708       GNLVRS01_PISO0F00753g   1760    eukaryota>fungi>ascomycota                              Millerozyma farinosa CBS 7064                                   Piso0_001918 [Millerozyma farinosa CBS 7064].     359379964       GNLVRS01_PISO0J00735g   1698    eukaryota>fungi>ascomycota                              Millerozyma farinosa CBS 7064                                   Piso0_001918 [Millerozyma farinosa CBS 7064].     260943486       CLUG_03282              1684    eukaryota>fungi>ascomycota                              Clavispora lusitaniae ATCC 42720                                hypothetical protein CLUG_03282 [Clavispora lusitaniae ATCC 42720].     255724008       CTRG_01239              1675    eukaryota>fungi>ascomycota                              Candida tropicalis MYA-3404                                     conserved hypothetical protein [Candida tropicalis MYA-3404].     68466611        CaO19.1451              1665    eukaryota>fungi>ascomycota                              Candida albicans SC5314                                         potential Ssn2p-like RNA Pol II transcription factor [Candida albicans SC5314].     238881952       CAWG_03919              1665    eukaryota>fungi>ascomycota                              Candida albicans WO-1                                           conserved hypothetical protein [Candida albicans WO-1].     150863729       PICST_66803             1660    eukaryota>fungi>ascomycota                              Scheffersomyces stipitis CBS 6054                               hypothetical protein PICST_66803 [Scheffersomyces stipitis CBS 6054].     241950783       CD36_16330              1656    eukaryota>fungi>ascomycota                              Candida dubliniensis CD36                                       SCA1 protein; suppressor of RNA polymerase B, putative [Candida dubliniensis CD36].     344303243       SPAPADRAFT_55383        1646    eukaryota>fungi>ascomycota                              Spathaspora passalidarum NRRL Y-27907                           hypothetical protein SPAPADRAFT_55383 [Spathaspora passalidarum NRRL Y-27907].     354547032       CPAR2_214090            1594    eukaryota>fungi>ascomycota                              Candida parapsilosis                                            hypothetical protein CPAR2_214090 [Candida parapsilosis].     380351500       CORT_0A13410            1589    eukaryota>fungi>ascomycota                              Candida orthopsilosis Co 90-125                                 Srb9 protein [Candida orthopsilosis Co 90-125].     146414045       PGUG_04948              1561    eukaryota>fungi>ascomycota                              Meyerozyma guilliermondii ATCC 6260                             hypothetical protein PGUG_04948 [Meyerozyma guilliermondii ATCC 6260].     190348403       PGUG_04948              1561    eukaryota>fungi>ascomycota                              Meyerozyma guilliermondii ATCC 6260                             hypothetical protein PGUG_04948 [Meyerozyma guilliermondii ATCC 6260].     302672423       SCHCODRAFT_238939       1821    eukaryota>fungi>basidiomycota                           Schizophyllum commune H4-8                                      hypothetical protein SCHCODRAFT_238939 [Schizophyllum commune H4-8].     389744296       STEHIDRAFT_169387       1793    eukaryota>fungi>basidiomycota                           Stereum hirsutum FP-91666 SS1                                   hypothetical protein STEHIDRAFT_169387 [Stereum hirsutum FP-91666 SS1].     393215315       FOMMEDRAFT_169058       1766    eukaryota>fungi>basidiomycota                           Fomitiporia mediterranea MF3/22                                 hypothetical protein FOMMEDRAFT_169058 [Fomitiporia mediterranea MF3/22].     409050049       PHACADRAFT_181524       1666    eukaryota>fungi>basidiomycota                           Phanerochaete carnosa HHB-10118-sp                              hypothetical protein PHACADRAFT_181524 [Phanerochaete carnosa HHB-10118-sp].     336373141       SERLA73DRAFT_70663      1629    eukaryota>fungi>basidiomycota                           Serpula lacrymans var. lacrymans S7.3                           hypothetical protein SERLA73DRAFT_70663 [Serpula lacrymans var. lacrymans S7.3].     299747209       CC1G_00012              1593    eukaryota>fungi>basidiomycota                           Coprinopsis cinerea okayama7#130                                hypothetical protein CC1G_00012 [Coprinopsis cinerea okayama7#130].     392592822       CONPUDRAFT_143634       1584    eukaryota>fungi>basidiomycota                           Coniophora puteana RWD-64-598 SS2                               hypothetical protein CONPUDRAFT_143634 [Coniophora puteana RWD-64-598 SS2].     403414339       FIBRA_03087             1529    eukaryota>fungi>basidiomycota                           Fibroporia radiculosa                                           predicted protein [Fibroporia radiculosa].     392569082       TRAVEDRAFT_63646        1433    eukaryota>fungi>basidiomycota                           Trametes versicolor FP-101664 SS1                               hypothetical protein TRAVEDRAFT_63646 [Trametes versicolor FP-101664 SS1].     393243223       AURDEDRAFT_182132       1421    eukaryota>fungi>basidiomycota                           Auricularia delicata TFB-10046 SS5                              hypothetical protein AURDEDRAFT_182132 [Auricularia delicata TFB-10046 SS5].     353234973       PIIN_00829              1414    eukaryota>fungi>basidiomycota                           Piriformospora indica DSM 11827                                 hypothetical protein PIIN_00829 [Piriformospora indica DSM 11827].     395330736       DICSQDRAFT_179767       1400    eukaryota>fungi>basidiomycota                           Dichomitus squalens LYAD-421 SS1                                hypothetical protein DICSQDRAFT_179767 [Dichomitus squalens LYAD-421 SS1].     170106341       LACBIDRAFT_294897       1335    eukaryota>fungi>basidiomycota                           Laccaria bicolor S238N-H82                                      predicted protein [Laccaria bicolor S238N-H82].     409075369       AGABI1DRAFT_131978      1317    eukaryota>fungi>basidiomycota                           Agaricus bisporus var. burnettii JB137-S8                       hypothetical protein AGABI1DRAFT_131978 [Agaricus bisporus var. burnettii JB137-S8].     321260623       CGB_G0310W              1443    eukaryota>fungi>basidiomycota                           Cryptococcus gattii WM276                                       hypothetical protein CGB_G0310W [Cryptococcus gattii WM276].     58269926        CNG04410                1432    eukaryota>fungi>basidiomycota                           Cryptococcus neoformans var. neoformans JEC21                   hypothetical protein CNG04410 [Cryptococcus neoformans var. neoformans JEC21].     134113597       CNBG0290                1432    eukaryota>fungi>basidiomycota                           Cryptococcus neoformans var. neoformans B-3501A                 hypothetical protein CNBG0290 [Cryptococcus neoformans var. neoformans B-3501A].     405121578       CNAG_03121              1432    eukaryota>fungi>basidiomycota                           Cryptococcus neoformans var. grubii H99                         hypothetical protein CNAG_03121 [Cryptococcus neoformans var. grubii H99].     71024333        UM06249.1               2199    eukaryota>fungi>basidiomycota                           Ustilago maydis 521                                             hypothetical protein UM06249.1 [Ustilago maydis 521].     343427997       sr16781                 2162    eukaryota>fungi>basidiomycota                           Sporisorium reilianum SRZ2                                      conserved hypothetical protein [Sporisorium reilianum SRZ2].     388857669       UHOR_08864              2100    eukaryota>fungi>basidiomycota                           Ustilago hordei                                                 uncharacterized protein [Ustilago hordei].     254572543       PAS_chr3_1134           1451    eukaryota>fungi>ascomycota                              Komagataella pastoris GS115                                     Subunit of the RNA polymerase II mediator complex [Komagataella pastoris GS115].     406607726       BN7_365                 1361    eukaryota>fungi>ascomycota                              Wickerhamomyces ciferrii                                        Mediator of RNA polymerase II transcription subunit 13 [Wickerhamomyces ciferrii].     320581145       HPODL_3739              1343    eukaryota>fungi>ascomycota                              Ogataea parapolymorpha DL-1                                     Subunit of the RNA polymerase II mediator complex [Ogataea parapolymorpha DL-1].     401881275       A1Q1_06023              1307    eukaryota>fungi>basidiomycota                           Trichosporon asahii var. asahii CBS 2479                        hypothetical protein A1Q1_06023 [Trichosporon asahii var. asahii CBS 2479].     392579006       TREMEDRAFT_58289        1258    eukaryota>fungi>basidiomycota                           Tremella mesenterica DSM 1558                                   hypothetical protein TREMEDRAFT_58289 [Tremella mesenterica DSM 1558].     406696928       A1Q2_05542              1042    eukaryota>fungi>basidiomycota                           Trichosporon asahii var. asahii CBS 8904                        hypothetical protein A1Q2_05542 [Trichosporon asahii var. asahii CBS 8904].     331243225       PGTG_15793              2617    eukaryota>fungi>basidiomycota                           Puccinia graminis f. sp. tritici CRL 75-36-700-3                hypothetical protein PGTG_15793 [Puccinia graminis f. sp. tritici CRL 75-36-700-3].     403175437       PGTG_22252              2269    eukaryota>fungi>basidiomycota                           Puccinia graminis f. sp. tritici CRL 75-36-700-3                hypothetical protein PGTG_22252 [Puccinia graminis f. sp. tritici CRL 75-36-700-3].     223634716       -                       1324    eukaryota>fungi>ascomycota                              Yarrowia lipolytica CLIB122                                     RecName: Full=Mediator of RNA polymerase II transcription subunit 13; AltName: Full=Mediator complex subunit 13.     210075691       YALI0D09086g            1175    eukaryota>fungi>ascomycota                              Yarrowia lipolytica CLIB122                                     YALI0D09086p [Yarrowia lipolytica].     207346279       AWRI1631_46600          414     eukaryota>fungi>ascomycota                              Saccharomyces cerevisiae AWRI1631                               YDR443Cp-like protein, partial [Saccharomyces cerevisiae AWRI1631].     365761266       VIN7_6510               326     eukaryota>fungi>ascomycota                              Saccharomyces cerevisiae x Saccharomyces kudriavzevii VIN7      Ssn2p [Saccharomyces cerevisiae x Saccharomyces kudriavzevii VIN7].     238487614       AFLA_035350             356     eukaryota>fungi>ascomycota                              Aspergillus flavus NRRL3357                                     hypothetical protein AFLA_035350 [Aspergillus flavus NRRL3357].     302407758       VDBG_07973              218     eukaryota>fungi>ascomycota                              Verticillium albo-atrum VaMs.102                                conserved hypothetical protein [Verticillium albo-atrum VaMs.102].     328768174       BATDEDRAFT_27048        1558    eukaryota>fungi>chytridiomycota                         Batrachochytrium dendrobatidis JAM81                            hypothetical protein BATDEDRAFT_27048 [Batrachochytrium dendrobatidis JAM81].     328766756       BATDEDRAFT_92392        1120    eukaryota>fungi>chytridiomycota                         Batrachochytrium dendrobatidis JAM81                            hypothetical protein BATDEDRAFT_92392 [Batrachochytrium dendrobatidis JAM81].     342320617       RTG_01089               1990    eukaryota>fungi>basidiomycota                           Rhodotorula glutinis ATCC 204091                                Proteophosphoglycan ppg4 [Rhodotorula glutinis ATCC 204091].     388580711       WALSEDRAFT_32990        621     eukaryota>fungi>basidiomycota                           Wallemia sebi CBS 633.66                                        hypothetical protein WALSEDRAFT_32990, partial [Wallemia sebi CBS 633.66].     402225946       DACRYDRAFT_102963       1505    eukaryota>fungi>basidiomycota                           Dacryopinax sp. DJM-731 SS1                                     hypothetical protein DACRYDRAFT_102963 [Dacryopinax sp. DJM-731 SS1].     164657774       MGL_2999                984     eukaryota>fungi>basidiomycota                           Malassezia globosa CBS 7966                                     hypothetical protein MGL_2999 [Malassezia globosa CBS 7966].     328849041       MELLADRAFT_118574       2099    eukaryota>fungi>basidiomycota                           Melampsora larici-populina 98AG31                               hypothetical protein MELLADRAFT_118574 [Melampsora larici-populina 98AG31].     358055802       E5Q_04830               1440    eukaryota>fungi>basidiomycota                           Mixia osmundae IAM 14324                                        hypothetical protein E5Q_04830 [Mixia osmundae IAM 14324].     213404492       SJAG_01778              1131    eukaryota>fungi>ascomycota                              Schizosaccharomyces japonicus yFS275                            predicted protein [Schizosaccharomyces japonicus yFS275].     302407756       VDBG_07972              720     eukaryota>fungi>ascomycota                              Verticillium albo-atrum VaMs.102                                conserved hypothetical protein [Verticillium albo-atrum VaMs.102].     149248454       LELG_01134              1860    eukaryota>fungi>ascomycota                              Lodderomyces elongisporus NRRL YB-4239                          hypothetical protein LELG_01134 [Lodderomyces elongisporus NRRL YB-4239].     295443004       SPAC589.02c             1225    eukaryota>fungi>ascomycota                              Schizosaccharomyces pombe 972h-                                 mediator complex subunit Srb9 [Schizosaccharomyces pombe 972h-].     385304966       AWRI1499_1089           655     eukaryota>fungi>ascomycota                              Dekkera bruxellensis AWRI1499                                   subunit of the rna polymerase ii mediator complex [Dekkera bruxellensis AWRI1499].     345560183       AOL_s00215g44           1647    eukaryota>fungi>ascomycota                              Arthrobotrys oligospora ATCC 24927                              hypothetical protein AOL_s00215g44 [Arthrobotrys oligospora ATCC 24927].     380486885       CH063_09501             400     eukaryota>fungi>ascomycota                              Colletotrichum higginsianum                                     hypothetical protein CH063_09501 [Colletotrichum higginsianum].     296419154       GSTUM_00007063001       980     eukaryota>fungi>ascomycota                              Tuber melanosporum Mel28                                        hypothetical protein [Tuber melanosporum Mel28].     154315535       BC1G_04340              153     eukaryota>fungi>ascomycota                              Botryotinia fuckeliana B05.10                                   hypothetical protein BC1G_04340 [Botryotinia fuckeliana B05.10].     384483763       RO3G_00647              1569    eukaryota>fungi                                         Rhizopus delemar RA 99-880                                      hypothetical protein RO3G_00647 [Rhizopus delemar RA 99-880].     320163917       CAOG_05948              2143    eukaryota                                               Capsaspora owczarzaki ATCC 30864                                predicted protein [Capsaspora owczarzaki ATCC 30864].     #;Med13_N+plant-specific+linker+MID+MedPIWI     12323178        F7A10.14                2655    eukaryota>viridiplantae                                 Arabidopsis thaliana                                            acetyl-CoA synthetase, putative; 45051-31547 [Arabidopsis thaliana].     297847896       ARALYDRAFT_314757       2638    eukaryota>viridiplantae                                 Arabidopsis lyrata subsp. lyrata                                hypothetical protein ARALYDRAFT_314757 [Arabidopsis lyrata subsp. lyrata].     168011095       PHYPADRAFT_161291       2216    eukaryota>viridiplantae                                 Physcomitrella patens subsp. patens                             predicted protein [Physcomitrella patens subsp. patens].     357462649       MTR_3g083500            2052    eukaryota>viridiplantae                                 Medicago truncatula                                             Mediator of RNA polymerase II transcription subunit [Medicago truncatula].     222631773       OsJ_18730               2039    eukaryota>viridiplantae                                 Oryza sativa Japonica Group                                     hypothetical protein OsJ_18730 [Oryza sativa Japonica Group].     218196888       OsI_20152               2010    eukaryota>viridiplantae                                 Oryza sativa Indica Group                                       hypothetical protein OsI_20152 [Oryza sativa Indica Group].     334183337       AT1G55325               2001    eukaryota>viridiplantae                                 Arabidopsis thaliana                                            RNA polymerase II transcription mediator [Arabidopsis thaliana].     359478798       LOC100263945            1932    eukaryota>viridiplantae                                 Vitis vinifera                                                  PREDICTED: uncharacterized protein LOC100263945 [Vitis vinifera].     242094348       SORBIDRAFT_10g000410    1923    eukaryota>viridiplantae                                 Sorghum bicolor                                                 hypothetical protein SORBIDRAFT_10g000410 [Sorghum bicolor].     42570236        AT1G55325               1921    eukaryota>viridiplantae                                 Arabidopsis thaliana                                            RNA polymerase II transcription mediator [Arabidopsis thaliana].     356566090       LOC100782017            1920    eukaryota>viridiplantae                                 Glycine max                                                     PREDICTED: uncharacterized protein LOC100782017 [Glycine max].     356540079       LOC100781873            1918    eukaryota>viridiplantae                                 Glycine max                                                     PREDICTED: uncharacterized protein LOC100781873 [Glycine max].     297746481       VIT_00025465001         1812    eukaryota>viridiplantae                                 Vitis vinifera                                                  unnamed protein product, partial [Vitis vinifera].     302816944       SELMODRAFT_447924       1714    eukaryota>viridiplantae                                 Selaginella moellendorffii                                      hypothetical protein SELMODRAFT_447924 [Selaginella moellendorffii].     302821757       SELMODRAFT_430729       1665    eukaryota>viridiplantae                                 Selaginella moellendorffii                                      hypothetical protein SELMODRAFT_430729 [Selaginella moellendorffii].     224075972       POPTRDRAFT_555194       1538    eukaryota>viridiplantae                                 Populus trichocarpa                                             predicted protein [Populus trichocarpa].     147858691       VITISV_024990           893     eukaryota>viridiplantae                                 Vitis vinifera                                                  hypothetical protein VITISV_024990 [Vitis vinifera].     168048099       PHYPADRAFT_193900       793     eukaryota>viridiplantae                                 Physcomitrella patens subsp. patens                             predicted protein [Physcomitrella patens subsp. patens].     357110896       LOC100841262            793     eukaryota>viridiplantae                                 Brachypodium distachyon                                         PREDICTED: uncharacterized protein LOC100841262 [Brachypodium distachyon].     26451642        At1g55320/F7A10_14      767     eukaryota>viridiplantae                                 Arabidopsis thaliana                                            putative acetyl-CoA synthetase [Arabidopsis thaliana].     326489047       -                       530     eukaryota>viridiplantae                                 Hordeum vulgare subsp. vulgare                                  predicted protein, partial [Hordeum vulgare subsp. vulgare].     308813732       Ot18g01410              652     eukaryota>viridiplantae>chlorophyta                     Ostreococcus tauri                                              unnamed protein product [Ostreococcus tauri].     145356022       OSTLU_18717             577     eukaryota>viridiplantae>chlorophyta                     Ostreococcus lucimarinus CCE9901                                predicted protein [Ostreococcus lucimarinus CCE9901].     307106572       CHLNCDRAFT_52772        720     eukaryota>viridiplantae>chlorophyta                     Chlorella variabilis                                            hypothetical protein CHLNCDRAFT_52772 [Chlorella variabilis].     159487743       CHLREDRAFT_186510       656     eukaryota>viridiplantae>chlorophyta                     Chlamydomonas reinhardtii                                       predicted protein, partial [Chlamydomonas reinhardtii].     302836101       VOLCADRAFT_90021        1618    eukaryota>viridiplantae>chlorophyta                     Volvox carteri f. nagariensis                                   hypothetical protein VOLCADRAFT_90021 [Volvox carteri f. nagariensis].     255564870       RCOM_0344660            794     eukaryota>viridiplantae                                 Ricinus communis                                                conserved hypothetical protein [Ricinus communis].     51854379        OSJNBb0012G21.6         1020    eukaryota>viridiplantae                                 Oryza sativa Japonica Group                                     hypothetical protein [Oryza sativa Japonica Group].     #;Med13_N+ZNF_with_insert+linker+MID+MedPIWI     195441007       Dwil_GK13446            2957    eukaryota>metazoa>hexapoda                              Drosophila willistoni                                           GK13446 [Drosophila willistoni].     195012105       Dgri_GH15559            2934    eukaryota>metazoa>hexapoda                              Drosophila grimshawi                                            GH15559 [Drosophila grimshawi].     195375016       Dvir_GJ12583            2860    eukaryota>metazoa>hexapoda                              Drosophila virilis                                              GJ12583 [Drosophila virilis].     198464423       Dpse_GA22138            2805    eukaryota>metazoa>hexapoda                              Drosophila pseudoobscura pseudoobscura                          GA22138 [Drosophila pseudoobscura pseudoobscura].     194875279       Dere_GG13266            2779    eukaryota>metazoa>hexapoda                              Drosophila erecta                                               GG13266 [Drosophila erecta].     161085371       Dmel_CG9936             2768    eukaryota>metazoa>hexapoda                              Drosophila melanogaster                                         skuld, isoform E [Drosophila melanogaster].     195348423       Dsec_GM22170            2761    eukaryota>metazoa>hexapoda                              Drosophila sechellia                                            GM22170 [Drosophila sechellia].     195592018       Dsim_GD12147            2731    eukaryota>metazoa>hexapoda                              Drosophila simulans                                             GD12147 [Drosophila simulans].     194749105       Dana_GF10192            2728    eukaryota>metazoa>hexapoda                              Drosophila ananassae                                            GF10192 [Drosophila ananassae].     195135539       Dmoj_GI16834            2687    eukaryota>metazoa>hexapoda                              Drosophila mojavensis                                           GI16834 [Drosophila mojavensis].     158295836       AgaP_AGAP006436         2679    eukaryota>metazoa>hexapoda                              Anopheles gambiae str. PEST                                     AGAP006436-PA [Anopheles gambiae str. PEST].     17864210        Dmel_CG9936             2618    eukaryota>metazoa>hexapoda                              Drosophila melanogaster                                         skuld, isoform D [Drosophila melanogaster].     195495715       Dyak_GE22364            2618    eukaryota>metazoa>hexapoda                              Drosophila yakuba                                               GE22364 [Drosophila yakuba].     170051282       CpipJ_CPIJ011676        517     eukaryota>metazoa>hexapoda                              Culex quinquefasciatus                                          conserved hypothetical protein [Culex quinquefasciatus].     195162909       Dper_GL26215            2374    eukaryota>metazoa>hexapoda                              Drosophila persimilis                                           GL26215 [Drosophila persimilis].     170044777       CpipJ_CPIJ007864        2034    eukaryota>metazoa>hexapoda                              Culex quinquefasciatus                                          conserved hypothetical protein [Culex quinquefasciatus].     312385608       AND_00565               1485    eukaryota>metazoa>hexapoda                              Anopheles darlingi                                              hypothetical protein AND_00565 [Anopheles darlingi].     #;Med13_N+linker+MID+MedPIWI     154418831       TVAG_198860             799     eukaryota>parabasalia                                   Trichomonas vaginalis G3                                        hypothetical protein [Trichomonas vaginalis G3].     123417375       TVAG_383300             791     eukaryota>parabasalia                                   Trichomonas vaginalis G3                                        hypothetical protein [Trichomonas vaginalis G3].     154422687       TVAG_070320             773     eukaryota>parabasalia                                   Trichomonas vaginalis G3                                        hypothetical protein [Trichomonas vaginalis G3].     123506133       TVAG_150650             771     eukaryota>parabasalia                                   Trichomonas vaginalis G3                                        hypothetical protein [Trichomonas vaginalis G3].     325185323       ALNC14_059530           1287    eukaryota>stramenopiles                                 Albugo laibachii Nc14                                           conserved hypothetical protein [Albugo laibachii Nc14].     348667661       PHYSODRAFT_261004       1282    eukaryota>stramenopiles                                 Phytophthora sojae                                              hypothetical protein PHYSODRAFT_261004 [Phytophthora sojae].     301110164       PITG_07956              1275    eukaryota>stramenopiles                                 Phytophthora infestans T30-4                                    conserved hypothetical protein [Phytophthora infestans T30-4].     9279805         amiB                    2678    eukaryota>amoebozoa>mycetozoa>dictyosteliida            Dictyostelium discoideum                                        AmiB [Dictyostelium discoideum].     66812080        DDB_G0282375            2678    eukaryota>amoebozoa>mycetozoa>dictyosteliida            Dictyostelium discoideum AX4                                    hypothetical protein DDB_G0282375 [Dictyostelium discoideum AX4].     330843345       DICPUDRAFT_158505       2186    eukaryota>amoebozoa>mycetozoa>dictyosteliida            Dictyostelium purpureum                                         hypothetical protein DICPUDRAFT_158505 [Dictyostelium purpureum].     281205290       PPL_07533               2062    eukaryota>amoebozoa>mycetozoa>dictyosteliida            Polysphondylium pallidum PN500                                  putative mediator complex subunit 13 [Polysphondylium pallidum PN500].     328870808       DFA_02429               2062    eukaryota>amoebozoa>mycetozoa>dictyosteliida            Dictyostelium fasciculatum                                      putative mediator complex subunit 13 [Dictyostelium fasciculatum].     290988095       NAEGRDRAFT_68069        1297    eukaryota>heterolobosea                                 Naegleria gruberi strain NEG-M                                  predicted protein [Naegleria gruberi].     326434915       PTSG_11128              1588    eukaryota>choanoflagellida                              Salpingoeca sp. ATCC 50818                                      hypothetical protein PTSG_11128 [Salpingoeca sp. ATCC 50818].     167516974       MONBRDRAFT_30987        1151    eukaryota>choanoflagellida                              Monosiga brevicollis MX1                                        hypothetical protein [Monosiga brevicollis MX1].     #;Med13_N?+linker+MID+MedPIWI     167376272       EDI_233510              752     eukaryota>amoebozoa>entamoebidae                        Entamoeba dispar SAW760                                         hypothetical protein [Entamoeba dispar SAW760].     407041070       ENU1_088270             752     eukaryota>amoebozoa>entamoebidae                        Entamoeba nuttalli P19                                          hypothetical protein ENU1_088270 [Entamoeba nuttalli P19].     67473598        EHI_008670              751     eukaryota>amoebozoa>entamoebidae                        Entamoeba histolytica HM-1:IMSS                                 hypothetical protein [Entamoeba histolytica HM-1:IMSS].     ```      Back to Contents       ---      **- C. Alignments for the MedPIWI family**      Alignments are preceded by secondary structure predictions and are followed by amino acid consensus at various thresholds.     **- 1) Med13\_N**       ```     FINAL           ----------EEEEEE-------------------------------------E----EEEEEEE----------------------------------------------------HHHHHHH-HHHHHH----------------EEEEEE----------------------------------------------------EEEEEE-----------------------------------------------------------------------------------------------------------------------------------------------------------------------------------------------------------------------------------------------------------------------------------------------------------------------------------------------------------------------------------------HHHHHHHHHHHHHHHHHHHHH------------------------------EE---EEEE---------------------------------------------------------------------------------EEEEEEEEEE-----------EEEEEEEE------EEE-----------------------------------------------------------------------------EEEEE----EEEEE-------------------------------------------------------------------HHHHHHHHHHHH-H-------------EEEEEE-------------EEEEE---------------------------------------------------------------------------------------------------------------EEEE---------------------------------------------------EEE-----EEEEE--     ALIGN           -----------HHHHH-------------------------------------HHH---EEEEEE-----------------------------------------------------HHHHHH-HHHHH-----------------EEEEEE----------------------------------------------------EEEEEE-----------------------------------------------------------------------------E---------------------------------------------------------------------------------------------------------------------------E-------------H-----------------------------------------------------------------------------------------------------------------------------------------------------------------HHHHHHHHHHHHHHHHHHHHH-----------------------------H-H---EEEE-----------------------------------------------------------------------------------EEEEEEEE------------EEEEEEE------EEE--H----HHHH------------------------------------------------------------------EEEEE-----------------------------------------------------------------------------HHHHHHHHHHH-H--------------------------------------------------------------------------------------------------------------------------------------------------HHHHEEHH-------------------------------------------------HEE-----EEEE---     HMM             ---------HHEEEEE-------------------------------------EE--EEEEEEEEE---------------------------------------------------HHHHHHH-HHHHHH----------------EEEEEEE---------------------------------------------------EEEEEEE------------------------------------------------------------------------------------------------------------------------------------------------------------------------------------------------------EEE-----EE------------------------------------------------------------------------------------------------------------------------------------------------------------------------HHHHHHHHHHHHHHHHHHHHH----------------------------------EEEEEE--------------------------------------------------------------------------------EEEEEEEEEE-----------EEEEEEEE-------EE-----------------------------------------------------------------------------EEEEE----EEEEE-------------------------------------------------------------------HHHHHHHHHHHH----------E-EEE-------------------EEEEEE-----------------------EEEEEE----------------H--HHHHHHHHHHHHH--HH--------------------EEEEE--------------------EEEEEE-------------------------------------------------EEE---HHHEE----     FREQ            ----------HHHHHH-------------------------------------HH---EEEEEEE------------------------------------------------------HHHHH-HHHHHH----------------EEEEEE----------------------------------------------------EEEEEE-----------------------------------------------------------------------------------------------------------------------------------------------------------------------------------------------------------------------------------------------------------------------------------------------------------------------------------------------------------------------------------------HHHHHHHHHHHHHHHHHHHHH-----------------------------EEEE--EEE-----------------------------------------------------------------------------------EEEEEEEEE-----------EEEEEEEE------EEEE----------------------------------------------------------------------------EEEE--------E---------------------------------------------------------------------HHHHHHHHHHH---------------EEEEEEE-----------EEEEEE------------------------------------------------------------------------------------------------------------------------------------------------------------------------------EEEEE--     PSSM            ----------HHEEEE------------------------------------------EEEEEE----------------------------------------------------HHHHHHHH-HHHHHH-----------------EEE------------------------------------------------------EEEEE------------------------------------------------------------------------------------------------------------------------------------------------------------------------------------------------------------------------------------------------------------------------------------------------------------------------------------------------------------------------------------------HHHHHHHHHHHHHHHHHHHHH------------------------------EE---EEEE---------------------------------------------------------------------------------EEEEEEEEEE-----------EEEEEEEE---------------------------------------------------------------------------------------EEEE----EEEE--------------------------------------------------------------------HHHHHHHHHHHH-H--------------EEEE-------------------------------------------------------------------------------------------------------------------------------------------------------------------------------------------------EEEE---     341879129       NNGGSLEDCVSNVYAL-------------------------------------LDLPGIKWKCFRPKPNA-----PRGLPLA----------------------------------SDLILKAY-SKCVLNE-------------ILATWRRKPLPPTDNTDMLPA-PYF-----------------------------SNDSAKELWVFWFDEE------------------------------------------------------------------PEELAKHCEG-----------------------------------------------------------------------------------------------------------------------LESDEELSSANQMNIVS-----------------------------------------------------------------------------------------------------------------------------------------------------------------YEVRTILFKALHIVLERDLIKD---------------------------GFVRFGRWFTVPL-------------------------SARE-------N-----Y--LHYLHS-------------------------NHSPAIRFNFFVHGS--------HTVCASVQAQ--RQPTLIALAK--RHIECKA------------------------------------------------------------VKRFPVVVGPWSMRGILVQDQ------------------------------------------------------LTILADPSMQENVEKEYNQWKE-YIQIEEKEPE-IIVEERPKTPDPEPSVLHP--TRVLLASSSDEEPTTSYET-DQANPESVPQRPRWMYEQDAPSEKPRELTYEEKEKRNQAREVRRRRRKNREERRKEQERQRSEVKNPEEYDSDVVTDPEV-ENKEEESVDSEIPKMVLVEIDN-------------------------------------------------VRMLYPSKLICVTLD     269849618       NNGGSLEDCISNVFSL-------------------------------------LELPGIKWKCFRPKLNA-----PRGVPL----------------------------------TSDLVLKAY-SRCLTD---------------GILCTWRRKPSPPTGNNELLPPTHFF---------------------------SNDSPKELWVFWYDAE------------------------------------------------------------------PTALGKYCEG-----------------------------------------------------------------------------------------------------------------------LDSDEELSSANQMNIVS-----------------------------------------------------------------------------------------------------------------------------------------------------------------YEVRTILFKALHVVLERDLTKD---------------------------GFVRFGRWFTMPL-------------------------VARD-------H-----Y--LHFMYP-------------------------SHSPAIRFNFFVHGT--------STICASIQAQ--RQPTLIKLAR--RHFECKT------------------------------------------------------------PKRFPVVIGPWSMRGYLIADQ------------------------------------------------------MTLLADQKIQEAAEKEWNQWKE-YLQLEEKEPE-NVTEERQKTPEAEPPAPPQ-TTRVLLASSSDEDQEKN--G-GELKSEEVSQKIRWMFEPDLRQDKPKEETAAEKEKRMAAREVRRLRRQRREERRREMEKQRRADDNLEDYDSDVVTDQDEDEAGKEEIVNNDVPKMILIEIDN-------------------------------------------------VRLLYPSKFVCITVE     308510662       INGGSLEDCLSNVYSL-------------------------------------LELPGIKWKCFRTKPNA-----PRGVPL----------------------------------TSDLVLKAY-SRCVLD---------------GILCTWRRKPSAPTDNDELLPATHFF---------------------------SNDSPKELWVFWYDDE------------------------------------------------------------------PIDLPKLCEG-----------------------------------------------------------------------------------------------------------------------LESDEELSSANQMNIVS-----------------------------------------------------------------------------------------------------------------------------------------------------------------YEVRTLIFKALHVVLERDLTKD---------------------------GFVRFGRWFTVPL-------------------------LARD-------N-----Y--LHFMYP-------------------------THSPAIRFNFFVHGT--------HTVCASVQAQ--RQPTLISLAK--RHFDCKT------------------------------------------------------------AKRFPVVVGPWSMRGYLIQDQ------------------------------------------------------MTLLADPKIQEAAEKEWNQWKE-YLQLEEKEPE-NVVDERQKSPDSEPTTAPPPAPRVLLASSSDEEQPKSDGN-DDVANDNIPQKFRWMYEPDYRQEKPKEETPEEKEKRMQAREVRRIRRKNREERLKELERQRAETTNPEDYDSDVVTDVEV-EVK-EESVSSEVPRMILIEIDN-------------------------------------------------VRLLYPSKFICITVE     268532428       NNGGSLEDCISNVFSL-------------------------------------LELPGIKWKCFRPKLNA-----PRGVPL----------------------------------TSDLVLKAY-SRCLTD---------------GILCTWRRKPSPPTGNNELLPPTHFF---------------------------SNDSPKELWVFWYDAE------------------------------------------------------------------PTALGKYCEG-----------------------------------------------------------------------------------------------------------------------LDSDEELSSANQMNIVS-----------------------------------------------------------------------------------------------------------------------------------------------------------------YEVRTILFKALHVVLERDLTKD---------------------------GFVRFGRWFTMPL-------------------------VARD-------H-----Y--LHFMYP-------------------------SHSPAIRFNFFVHGT--------STICASIQAQ--RQPTLIKLAR--RHFECKT------------------------------------------------------------PKRFPVVIGPWSMRGYLIADQ------------------------------------------------------MTLLADQKIQEAAEKEWNQWKE-YLQLEEKEPE-NVTEERQKTPEAEPPAPPQ-TTRVLLASSSDEDQEKN--G-GELKSEEVSQKIRWMFEPDLRQDKPKEETAAEKEKRMAAREVRRLRRQRREERRREMEKQRRADDNLEDYDSDVVTDQDEDEAGKEEIVNNDVPKMILIEIDN-------------------------------------------------VRLLYPSKFVCITVE     71992166        NNGGSLEDCISNVYAL-------------------------------------LELPGIKWKCYRTKPNA-----PRGVAL----------------------------------TADFVLKAY-SKCLLD---------------GILCTWRRKPLPPTDNKETPKLTNF----------------------------SNDAPKELWVFWYDDE------------------------------------------------------------------PALLQKNCEG-----------------------------------------------------------------------------------------------------------------------LDNDDELSSANQMNIVS-----------------------------------------------------------------------------------------------------------------------------------------------------------------YEVRTIIFKALHVVLERDLTKD---------------------------GFVRFGRWFTVPY-------------------------DARE-------N-----YLHFTYP---------------------------SHSPAIRFNFFVHGS--------STICASVQAQ--RQPTLITLAR--RHLECKT-------------------------------------------------------------KRVVVVVGPWSMRGQLVQDQ------------------------------------------------------IALLADPKIQESAEKEWNQWKD-YLQMEEKEPE-NVSEERQKSPDSDPPAPPQ-TTRVLLADSSDEEQSQSFEAIDESKKDDIPQRLRWMYEPDYRQEKSKEETPEEKEKREQSREVRKQRRKLREERRKEVERQRTEVKNPDDYDSDVVTDEDV--GEKEECVNNDVPKMVLLDIDG-------------------------------------------------VRLLYPSKFLCVTID     326434915       -MPSKPQEERTNVIKL-------------------------------------GTLHDVRWSQLVWNKAD--------------------------------------------VLGTGAAVNH-EHELFQ--------LQAQYDESNFLTLPVLRTTKASV-------------------------------------LPTRKRQAALYIFSLT--------------------------------------------------------------DKDPPNLSSSSQAT-----------------------------------------------------------------------------------------------------------------------------FHKGS---ALDAEEN-------------------------------------------------------------------------------------------------------------------------------------------------------------CDIVQLLFRAIMSMLERHMGGD---------------------------GYVRVNDCFFPRE---------------------------------------------------------------------DDEPRRLQNHGVRVRPFLIRS---------SNVCVRVQAV--TNPYQLLSA---PDYDVAA--------------------------------------------------------------HTHARLVPTGDVCELVHPPRPTSSGSISDCDDDNDASDNTAGVAAVATHPRPHQGNSTSPLPLSSARPAAAAAAAAAAALPFTYPRPACVAAWER-VAPHLASAVDAAP--------------------------------------------------------------------------------------------------------------------------------------AHLVRVRL-DG----------------------------------------------VAGVYPERLLVVPRH     348667661       ERAADAPTLTTTTFAL-------------------------------------CELASLEWHVYSVDKDK-----------------SVDKDKDTSSGKKKKHGKKLPSNSGVKQGEEAALAVF-SQRLQQ--------------RNALCVLQ--------------------------------------DDDANKEEDTASLSQLWVFIANNM-------------------------------------------------------------QSSVPVEPPSGVLET--------------------------------------------------------------------------------------------------------------------------LSGSWLESANEALD-----------------------------------------------------------------------------------------------------------------------------------------------------------------SIVQQQFFAALASVLSRKLLAQE--------------------------EFNLSTDGFYEPN-------------------------------------------------------------------DAKFIFRCPSLTRLNHVEVYLEEE-------FPTPAFQLHFQ------LFRPSN------------------------------------------------------------------------LLAVAVDVVKRERGLSEK------------------------------------------------------------LQHGQELGDPRCSWER-LVGLPSLTD------------------------------------------------------------------------------------------------------------------------------------------HSMVDVWHLKDGLV-------------------------------------------PRIIAETKYTDVAPW     301110164       ERVRSAPTLTTNCFAL-------------------------------------CELVSLQWHVYGVAPVE-----------------GVAPVEETS---KKKTNSKKAAKSGSKQAEDAALSIF-HQRLRQ--------------RNAVCVLT--------------------------------------EDQRTEQDAASSSSELWVFIVNGM-------------------------------------------------------------PSAVPAEPPTGVCET---------------------------------------------------------------------------------------------------------------------------STGSWTGNEESLD-----------------------------------------------------------------------------------------------------------------------------------------------------------------RNIQQQVFAALDSILSKKLVAQE--------------------------EFVLTEGGFFEPN-------------------------------------------------------------------DAKFIFRCPSLTRLNHVEVYLEEE-------LPVPAFQLHFQ------LFEPSH------------------------------------------------------------------------LLTVTVDVVRRDGSLSSF---------------------------------------------------------------GDELGDTRCSWER-LVGLPSQKD------------------------------------------------------------------------------------------------------------------------------------------HSVVDVWHLKDGLV-------------------------------------------PRIVAETKYTDVSPW     325185323       NPAAEASQLETNVTIV-------------------------------------CELQSLKWQRYEAVLTDVRHNNTAENCKDDIASFSAGFDSAHDTDPKRSTNVRATAFRAEEKELENDVKKT-RDRMQSQ-------VERLLHLNFPCYVL----------------------------------------------IDSEKLTFWVFNVNEK-------------------------------------------------------------HSFPTLDSPLSLTEQ---------------------------------------------------------------------------------------------------------------------------GCWKPNGTANTMR-----------------------------------------------------------------------------------------------------------------------------------------------------------------LDVKKYFEEALQIQWIRSLTQSREFELQPLNPACIPRIERSTHFSQEAFCVVPTQLPTFEYR--------------------------------------------------------------------------RFVRHHVFQTEVYLTEPH------LPLPVFRFHFQ------IVNFGN----------------------------------------------------------IAQIRMNMEMCIRQLEATNKDVEDRERSLD-------------------------------------------------------------------HLGNPKSAWLR-LMGLPPASK------------------------------------------------------------------------------------------------------------------------------------------PEIVDVWH-QDGVLP------------------------------------------ARIAAQTKFTSVHPQ     393215315       QQLTPDSKLSASCISL-------------------------------------SPGFSVHFAVFSAQNA--------------------------------------------AQSHLDVDLAR-RK-VHESNSGRPIY------NCLLTHPH----------------------------------------------LAKDEATLWVFTIDQGIPESDGIYASP---------------------------------------------------QL-RDLELPGLARN---------------------------------------------------------------------------------------------------------------------------TNGSLTY--AELY-----------PCSTTCSSQRIPCPNCLRISAGSEITAHAQTSPGSINPEPEETTPAISPTSSHGRTFDN-----------------------------PSVTC----HLPRKPL----------------------------------------------RQALAFFLSAVQERVISDICEDAQL-S------------------GKVARRLQTG-ILWGLP--------------------------------------------------------------SLSSNWGGGWEQHAKTRPLTFTHIDVTLFP-------SGLFIRPQAQL-TNFLPFLPS---LPLPP----------------------------------------------------------------GTPINLLPFSTPAYYLNMY---------------------------------------------------------------SGSTSALTRHFQANLVGLGAGDVL----------------------------------------------------------------------------------------------------------------------PSRGTDQTTSSSENTTQFVIAWISVQNKQGEEKG------------------------------------------MMVIWPAPLCISLMS     302672423       PRISYADELLTSAFDL-------------------------------------TRTPCLVWCRYSSYADA-------------------------------------------IDGAEAVERAR-LA-IPESNAGKGLL------ESDLTSVR----------------------------------------------AGSR-SRIYVFRLGAKEALREMKTAMA-------------------------------------------------------KLSFEGLEAC-------------------------------------------------------------------------------------------------------------------------------IAL--DEIY-----------GPT-------------------------------------------------------------------------------------NSSTA----RIPSKTT----------------------------------------------RTILSHFFEAIRTRFVDDVVRQATN-V------------------KRDVQRFKTG-FLFGAT---------------------------------------------------VPMARSTKEQQSTEDNWSAGWDAYASARPLTFVHLDIQLDA------RLRLLINPIFRP-TSHRPLPLPTPFHRHSRTS-----------------------------------------------------HASHSSLSAGIPITLLPHGTAAHFLAEY--------------------------------------------------------------TPTDSRALDREFEKGFRGTGVLFHT----------------------------------------------------------------------------------------------------------------------SISSSPQDHNAHIPSTPYIIGYLAIENRHGEPKG------------------------------------------LTFVYPRQLCLLSTD     393243223       LSAASVPGIVLSFLAL-------------------------------------PKDALVLCSSYAPLGDPA-------------------------------------------THCHSIEHAR-RHLLHDRRQPSLV-------DSLLAHVD----------------------------------------------SSSHDTRFWVFAVASRATQPSRAHALA-------------------------------------------------------SLALHNLIAV---------------------------------------------------------------------------------------------------------------------------DSASFDF--RQLY-----------SSN--------------------------------------------------------------------------------------SQSP----AFVTPGL----------------------------------------------HRLYLMFLAALRSRILADICDNVPD-R----------------VRASH-VRMRDG-FLLVPR--------------------------------------------------------------PVNSEWSVGWD--DPQRRLAYCHLHLHWMN------ASTIVLRPVFST-SQYSQLSPK----HCPA----------------------------------------------------------------GQPVALLPYAVPAYFIAPY---------------------------------------------------------------TGSTAALSQHFRDSLAGLGVGPTW-----------------------------------------------------------------------------------------------------------------------------------ESQGYILCWIAVHNSQGDDKA------------------------------------------VLAVWPAALAVACPD     299747209       ---MAQNTVLAAILHL-------------------------------------QNSPGFVYRQYN------------------------------------------------APDYDACELAR-RTVVDASTDI----------LNSVHVAV----------------------------------------------HGSPSPCLYLFLITSAEAVHDAIARVN-------------------------------------------------------TYAFDYLQGT---------------------------------------------------------------------------------------------------------------------------EPAFLPL--DQLS-----------RPT-----------------------------------------------------------------------------------------------TTDAST----------------------------------------------TEIYSLFLDAVRSRAIADIVDTAST-L------------------PRHVHRFKDG-FIIHRT--------------------------------------------------------------PAVSDWAAGWEHKLLNRPVVYFHLQIHFACSAGADSPSHLLIHLTPIS-TPFSNIPIN---TALRP----------------------------------------------------------------GSPIILLPYGTPAYFLASY---------------------------------------------------------------NGPSNALTKLFDDSLRGLGAGRWS-----------------------------------------------------------------------------------------------------------------------------LDGQSASSPPFIIGWIRVENKQGEDKG------------------------------------------TPFVWPTRLCLSYLP     409050049       LSQHPQRISLSSPILA--------------------------------SAIDLPPNPLISCSVFIATRAPTP-----------------------------------------TEQLTAIENAR-RRIVADYT-PLPMA------QSLLPSVH----------------------------------------------VTKDRVSLYLFAFGSTMDASAAQMPLSLGTRKTRH---LLQLKMLLCESGAVLYRQSFTYISCVRVAERNGPCTMGGPGL-LAHAKRCIQKF---------------------------------------------------------------------------------------------------------------------------DTYSFTP--DGIY-----------PCSLSCATQRVPCAVCIQQREAS---SFATPSSQ------------------------------------------------------ASPTC----SLPRKPL----------------------------------------------RLPLVQFIQAVRDRIIDDIVRSSSE-T----------------S-ERWTGRLQGG-FLLSPL---------------------------------------------------SP---------ADDGEWGAEWEHYGRSRPFVHCELQISLSQ-------TRLVILPILRT-TQYLPLTTS---LSLPT----------------------------------------------------------------GTPIVLLPHGTPAFYLNTY---------------------------------------------------------------SGALGNLTHQFEDALFGLGVGNWK---------------------------------------------------------------------------------------------------------------------------SAPVNESTNSPCYLIAWVSVQNKQGEEKG------------------------------------------LPVIWPTTLSVLPDG     389744296       PNISLSSHVLSSALKF-------------------------------------PSEPVIAYAIYSTSGLST-------------------------------------------VHSENVELAR-REVLRRRTTATPNT-KHGILESLLASVL----------------------------------------------VERETLHLYVFGVSSVGETMQWSETLK-------------------------------------------------------ALNFDGLTLS---------------------------------------------------------------------------------------------------------------------------KVSSFSP--ADLY-----------PCSPECASLDSACNHCIDPSVKLETLHWKSEARY------------------------------------------------------PPSLR----HLPREPL----------------------------------------------RRPYAQFLQAVRERLTDDMCESSKD--------------------GRRLRRLKDG-FLIFPP--------------------------------------------------------------SDVSDWGLGWEHHSRNRPLIHCYLHLFSTP-------TRILIQPIFRP-SYFLPILPS---LPLPA----------------------------------------------------------------GTPVTLLPYGTPAYYLTTY---------------------------------------------------------------AGPTSALTAQFSQSLAGLGCSDWH------------------------------------------------------------------------------------------------------------IEYSP----RPPHNRDPDASSSTKGPTYIIAWINVQNKQGEEKG------------------------------------------LTVIWPASLTLTFVP     336373141       PHVSLSEQVLSSVVSL-------------------------------------PDNPVIAYTTFTTQSTT-------------------------------------------IRPYEALELAR-RLLVNRNK-GLPLL------DSLLPCVN----------------------------------------------VSQDLSALQVFAITSRDHVSAKLAAIK-------------------------------------------------------DISFDGLTVS---------------------------------------------------------------------------------------------------------------------------GESSFTP--HDLY-----------PCSLACSDDQSPCPSCLKRDPSSLSQSTL-----------------------------------------------------------SSPAC----LLPRKPL----------------------------------------------RSAYYHFLDAVRKRLIDDISEASRK-T----------------PHGRCARGFQDG-FLLSPP--------------------------------------------------------------RSYFDWGVDWQHHVNTRPFIHCHLEIHLAT-------SRLEIHPIIRP-THFSPLSVH---LPLAP----------------------------------------------------------------GTPIALMPYSTPAFFLATY---------------------------------------------------------------TGPTSALSAQFEQSLAGLGAGDWK------------------------------------------------------------------------------------------------------------TMTSPYSHSVSNGKHRSADPSARKYPMYIIAWLAVENKQGEDKG------------------------------------------MNIIWPTELCLSYLP     392592822       PQLAPSDKLLSSVVNL-------------------------------------PSDPSIVYATFFPTGFLA------------------------------------------LPQYDVLEHAR-RKLVARNQ-SSSPS----LWDSLLPSVY----------------------------------------------ISQD-SSLYVFSIVSSDHTEAVKSSIE-------------------------------------------------------ALELDGLAVS---------------------------------------------------------------------------------------------------------------------------DVLSFKP--DDLY-----------PCSAECSDRAAPCPNCISGSGSP-----------------------------------------------------------------NSTAP----LLPRKPL----------------------------------------------RIVYSHFLDAVRSRLIDDLANTSVP--------------------GTIIQRCKNG-FLLGSI--------------------------------------------------------------PSTYEWADDWQDTTRTRSMLLVQLDLHLAR-------TRLEIHFRLKP-TYFLPLHLT---LPLSA----------------------------------------------------------------GSPIILLPYATPAFFLTTY---------------------------------------------------------------AGPTSAITKLFEQALAGLGTGEWK------------------------------------------------------------------------------------------------------------PHPHS-----SAKGKHHRVDDADRQPMYIIAWLAVQNKQGEDKG------------------------------------------MTIIWPRRLCLGYHP     242094348       DGSTGSQEAAAQLR---------------------------------------GELQTVSWFQFLPFEPDASAASEKSSKAE---------------------------------QKDALNSIVLSAYLHLQS------------EGFLSTWTNSFVGPWDPSQGEH--------------------------------NPDEKIKLWLFLPGCH-----------------------------------------------------------------SSVSEKAQPAV---------------------------------------------------------------------------------------------------------------------NKLRVASNGLWVA--PGNS-----------------------------------------------------------------------------------------------------------------------------------------------------------------EEVAAALSQALRNSLERSLKGL---------------------------SYARFGDVFTKYN-----------------------PPTRNQ-------N--------S------------------------------FRRAQPTVEFVFAAT-------EEAIFVHVLIS--ARYVRNLCS---DDIEKVL-----------------------------------------------------THSPPSIGEGLPVVVAPSGMLGRLVGCC---------------------------------------------------------------------PSDLVRQ-VYSSKSSAPN-------------------------------------------------------------------------------------------------------------------------LPGFSQPTV-CQLRGQSYYVEVAL-GFPAASADKVSESEHIQIKKELDPVKDAQVGADGQRKVESPDSLPVFERTFIYPPEAILVPMV     12323178        IVPEFPRTASSKLLRR-------------------------------------GGLHNVSWFQFLPSETELNPGFDRSSRAE---------------------------------QNEVATYLVLSSHLRLQK------------EGFLTTWTNSFVGPWDPSQGLY--------------------------------NPDEKIKLWLFLPGRH-----------------------------------------------------------------SSISDKAQAAV----------------------------------------------------------------------------------------------SKLRVNMPVVYGYLGILVLTFLFLLLKVVASGIWVA--PGDS-----------------------------------------------------------------------------------------------------------------------------------------------------------------EEISVAFSQSLRNCIERALSGI---------------------------SYMRFGDVFSKFS-------------------------PQSE-------E---------------------------------------YLRGQPTVEFIFAAT-------EEAVFVHVIIS--AKNVRTLSS---GDAERML-----------------------RSSLKNSSYRLPAFRKCLGLAKSEDNRLCYINTSHRPMLFPPVIVSPHGMRGSLTGFC---------------------------------------------------------------------PNDLVKQVYFSSGNLKTS--------------------------------------------------------------------------------------------------------------------TGYVGLPSHIGRGS-RLINGNHCYVEVTL-GCCQNRNDNTSQANSTFAVNLPHNQCPEPSVGSKDHRKGQSDLSSVCEKKFIYPAEAVLVPIL     334183337       --------MWTNVFRI-------------------------------------GGLHNVSWFQFLPSETELNPGFDRSSRAE---------------------------------QNEVATYLVLSSHLRLQK------------EGFLTTWTNSFVGPWDPSQGLY--------------------------------NPDEKIKLWLFLPGRH-----------------------------------------------------------------SSISDKAQAAV---------------------------------------------------------------------------------------------------------------------SKLRVVASGIWVA--PGDS-----------------------------------------------------------------------------------------------------------------------------------------------------------------EEISVAFSQSLRNCIERALSGI---------------------------SYMRFGDVFSKFS-------------------------PQSE-------E--------Y------------------------------LRRGQPTVEFIFAAT-------EEAVFVHVIIS--AKNVRTLSS---GDAERML-----------------------------------------------------RSSLKNSSYRLPVIVSPHGMRGSLTGFC---------------------------------------------------------------------PNDLVKQVYFSSGNLKTS--------------------------------------------------------------------------------------------------------------------TGYVGLPSHIGRGS-RLINGNHCYVEVTL-GCCQNRNDNTSQANSTFAVNLPHNQCPEPSVGSKDHRKGQSDLSSVCEKKFIYPAEAVLVPIL     42570236        --------MWTNVFRI-------------------------------------GGLHNVSWFQFLPSETELNPGFDRSSRAE---------------------------------QNEVATYLVLSSHLRLQK------------EGFLTTWTNSFVGPWDPSQGLY--------------------------------NPDEKIKLWLFLPGRH-----------------------------------------------------------------SSISDKAQAAV---------------------------------------------------------------------------------------------------------------------SKLRVVASGIWVA--PGDS-----------------------------------------------------------------------------------------------------------------------------------------------------------------EEISVAFSQSLRNCIERALSGI---------------------------SYMRFGDVFSKFS-------------------------PQSE-------E--------Y------------------------------LRRGQPTVEFIFAAT-------EEAVFVHVIIS--AKNVRTLSS---GDAERML-----------------------RSSLKNSSYRLPAFRKCLGLAKSEDNRLCYINTSHRPMLFPPVIVSPHGMRGSLTGFC---------------------------------------------------------------------PNDLVKQVYFSSGNLKTS--------------------------------------------------------------------------------------------------------------------TGYVGLPSHIGRGS-RLINGNHCYVEVTL-GCCQNRNDNTSQANSTFAVNLPHNQCPEPSVGSKDHRKGQSDLSSVCEKKFIYPAEAVLVPIL     359478798       --------MWTNVFKI-------------------------------------GGLHHISWFQFLPHESDLNPPNDKSVKVE---------------------------------QKDPATLVVLSTHLQLQR------------EGFLSTWTNSFVGPWDPSQGLH--------------------------------NPDEKIKLWLFLPGRH-----------------------------------------------------------------SSVAEAAQVAV---------------------------------------------------------------------------------------------------------------------SRLRVVASGFWLA--PGDS-----------------------------------------------------------------------------------------------------------------------------------------------------------------EEVAAALSQALRNCIERALIGL---------------------------NYMRFGDVFSKYH-----------------------PFSQSE-------E--------L------------------------------FRRGQPTIEFIFAAT-------EEAIFVHVIIS--AKHVRALAS---GDMEMVL-----------------------------------------------------KHSSNKYSESLPVIVSPHGMLGRFTGCC---------------------------------------------------------------------PSDLVKQVYFS--KFKTS--------------------------------------------------------------------------------------------------------------------NGFIGLPYHLSQGSGCQLRGQNCYVEVTL-GCPSAGTDKMLQSNSNSIRNFPKYHVADPHAMGKGAQKGLPDHVSE--RTFIYPAEAVLVPVL     297746481       --------MWTNVFKI-------------------------------------GGLHHISWFQFLPHESDLNPPNDKSVKVE---------------------------------QKDPATLVVLSTHLQLQR------------EGFLSTWTNSFVGPWDPSQGLH--------------------------------NPDEKIKLWLFLPGRH-----------------------------------------------------------------SSVAEAAQVAV---------------------------------------------------------------------------------------------------------------------SRLRVVASGFWLA--PGDS-----------------------------------------------------------------------------------------------------------------------------------------------------------------EEVAAALSQALRNCIERALIGL---------------------------NYMRFGDVFSKYH-----------------------PFSQSE-------E--------L------------------------------FRRGQPTIEFIFAAT-------EEAIFVHVIIS--AKHVRALAS---GDMEMVL-----------------------------------------------------KHSSNKYSESLPVIVSPHGMLGRFTGCC---------------------------------------------------------------------PSDLVKQVYFS--KFKTS--------------------------------------------------------------------------------------------------------------------NGFIGLPYHLSQGSGCQLRGQNCYVEVTL-GCPSAGTDKMLQSNSNSIRNFPKYHVADPHAMGKGAQKGLPDHVSE--RTFIYPAEAVLVPVL     357462649       --------MWTNVFKI-------------------------------------GSLHQISWFQFLPHEPDLNPLPDKSVKAD---------------------------------QKDAAMLVVLSSHLQLQK------------EGFLSAWTNSFVGPWDPSQGLH--------------------------------NPDEKIKLWLFLPGRH-----------------------------------------------------------------LTVSETAQPAL-----------------------------------------------------------------------------------------------------------TGLRGITLNRTVVLAVASGLWLA--PGDS-----------------------------------------------------------------------------------------------------------------------------------------------------------------EEVAAALSQALRNCIERALLGL---------------------------YYMRFGDVFLKVH-----------------------QFQSEE-----L-----------------------------------------LRRGHPAFEFVFAAT-------EEAILIHVIVS--SKNIRMLSS---GDLEKLL------------------------------------------------------KHSMETTYTLPVIVSPHGIRGNLTGCS---------------------------------------------------------------------SSDLVKQSYFSSSAKFRV-------------------------------------------------------------------------------------------------------------------SNGIIGLPYHVSQGVGCQLRGQNCFVEVSL-GFPRSETDKALQSNKNIRNLLKSPVTGHNDGKGSPDHLSDNE------KTFLYPAEAVLVPVF     356566090       --------MWTNVFKI-------------------------------------GSMHQISWFQFLPHEPDLNPLPDKSVKVD---------------------------------QKDAAMLLVLSSHLQLQK------------EGFLSTWTNSFVGPWDPSQGLH--------------------------------NPDEKIKLWLFLRGRH-----------------------------------------------------------------SSVVETAQTAV---------------------------------------------------------------------------------------------------------------------SGLRVVASGLWLA--PGDS-----------------------------------------------------------------------------------------------------------------------------------------------------------------EEVAAALSQALRNCIERALLGL---------------------------YYMRFGDVFSKFH-----------------------QFQREE-----I-----------------------------------------FRRGQPAVEFVFAAT-------EEAIFIHVIVS--SKHIRMLST---ADLEKVL------------------------------------------------------KHSMESTYRLPVIVSPHGIRGSLTGCS---------------------------------------------------------------------PSDLVKQSYFSSTKFRVS--------------------------------------------------------------------------------------------------------------------NGIIGLPYHVSQGVGCQLRGQNCYVEVSL-GFPRSGTDNTLQPNKNSVRNLPKLHVAESPVVGRSDHKGPPDHLLDYDKTFLYPAEAVLVPVL     356540079       --------MWTNVFKI-------------------------------------GSLHQISWFQFLPHEPDLNPLPDKSVKVD---------------------------------QKDAAMLLVLSSHLQLQK------------EGFLSTWTNSFVGPWDPSQGLH--------------------------------NPDEKIKLWLFLPGRH-----------------------------------------------------------------SSVVETAQTAV---------------------------------------------------------------------------------------------------------------------SGLRVVASGLWLA--PGDS-----------------------------------------------------------------------------------------------------------------------------------------------------------------EEVAAALSQALRNCVERALFGL---------------------------YYMRFGDVFSKFH-----------------------QFQREE----------------L------------------------------FRRGQPAVEFVFAAT-------EEAIFIHVIVS--SKHIRMLST---ADLEKVL------------------------------------------------------QHSMEFTYRLPVIVSPHGICGSLTGCS---------------------------------------------------------------------PSDLVKQSYFSSTKFRVS--------------------------------------------------------------------------------------------------------------------NGIIGLPYHVSQGVGCQLRGQNCYVEVSL-GFPRSGTDNTLQPNKNSVRNLPKLHVAESPIVGRSDHKGSPDHLLDYDKTFLYPAEAVLVPVL     221129420       ENVDFLADCSTNVFFL-------------------------------------TDLRGLRWKRYTSSC-------KNVTSI----------------------------------TDDPILSAY-SAALHA---------------DILCVWQRVAVHGISVDSVGAH-------------------------------LLKCPKELILFWSGEK------------------------------------------------------------------PTLNDILSSN-----------------------------------------------------------------------------------------------------------------------LQEVDYGTYED---GLS-----------------------------------------------------------------------------------------------------------------------------------------------------------------EEILELFFRSLHNMIEKYLTEN---------------------------EFVRLGKWFVSPL-------------------------KNDA-------N-----N--NSSV---------------------------FSSLAFSFSLFLHGE--------TSVVASINIQ-EKSSIRHLSA---KDLHMST---------------------------------------------------------LPMSSESHVLLAPYGLNAQLTGHN-----------------------------------------------------------LGESDTSSLKILEEWQR-FFPSVPALST--------------------------------------------------------------------------------------------------------------------------------------FGQPNVVEVVL-GD----------------------------------------------IKMLYPAKLVLIPSS     198415621       ANGARLDDCHSSVFSL-------------------------------------ADLSGIKWHRYS-----------SISPGL---------------------------------ADDPVLAAF-TKCLAL---------------DILAVWRKSSKKSPMQHGLP---------------------------------QEGIERELWIFWWGEE------------------------------------------------------------------PNIDEISQQR----------------------------------------------------------------------------------------------------------------------GLNDIGRGSWDE---GLT-----------------------------------------------------------------------------------------------------------------------------------------------------------------YECRTLLFKAIHNLVERCLLNE---------------------------NFVRIGRWFVKPL-------------------------NKDN-------E--------EDI----------------------------SDRFSFSFSFFLHGE--------SYVCTTIEIA-KHQPLERLTY---QHFQHVT-----------------------------------------------------------SSTNNSVVLAPYGLAATLTGTA----------------------------------------------------------YRNPHEPTVKKLLEEWKP-FYPMSTSDDF------------------------------------------------------------------------------------------------------------------------SMGDLCDDETEDDNIPVPPVVEVVI-AG----------------------------------------------VQMNYPSHYILVPVE     348535272       PNGASLEDCHSNLFCL-------------------------------------ADLTGIKWRRFVWQGPTSS---PILFPVT---------------------------------EEDPILCSF-SRCLAA---------------DVLSVWRRHHTP--------------------------------------------GRRELWLFWWGDD------------------------------------------------------------------PSFAELIHNE-----------------------------------------------------------------------------------------------------------------------LLSEEDGEWES---GLS-----------------------------------------------------------------------------------------------------------------------------------------------------------------YECRTLLFKAIHNLLERCLMNR---------------------------GFVRIGKWFVKPY-------------------------QKEE-------K-----T--INK----------------------------SEHLSCAFNFFVHGD--------SNVCTSVEIA-QHQPLQRLSE---EHLSLAQ----------------------------------------------------------QSSSPLQVILSPYGLNGTLTGQA-----------------------------------------------------------FKMSDHPTQKLIEEWRQ-FYPISPNPKE-------------------------------------------------------------------------------------------------------------------------VQEEKLE-DADWEDDSLAAVEVLV-AG----------------------------------------------VRMVYPSCLVLLPLS     47210173        PNGASLEDCHSNLFCL-------------------------------------ADLTGIKWRRFVWQGPTSS---PILFPVT---------------------------------EEDPILCSF-SRCMAA---------------DVLSVWRRHHTQ--------------------------------------------GRRELWLFWWGDD------------------------------------------------------------------PSFAELIHNE-----------------------------------------------------------------------------------------------------------------------LSSEEDGEWES---GLS-----------------------------------------------------------------------------------------------------------------------------------------------------------------YECRTLLFKAIHNLLERCLMNR---------------------------GFVRIGKWFVKPY-------------------------QKEE-------K-----I--INK----------------------------SEHLSCAFTFFVHGD--------SNVCTSVEIA-QHQPLQRLSE---EHLSLAQ----------------------------------------------------------QSSSPLQVILSPYGLNGTLTGQA-----------------------------------------------------------FKMSDHPTQKLIEEWRQ-FYPISPNPKE-------------------------------------------------------------------------------------------------------------------------VQEDKME-DSDWEDDSLAAVEVLV-AG----------------------------------------------VRMVYPSCLVLLPLT     410910158       PNGASLEDCHSNLFCL-------------------------------------ADLTGIKWRRFVWQGPTSS---PILFPVT---------------------------------EEDPILCSF-SRCMAA---------------DVLSVWRRHHTP--------------------------------------------GRRELWLFWWGDD------------------------------------------------------------------PSFAELIHNE-----------------------------------------------------------------------------------------------------------------------LSSEEDGEWES---GLS-----------------------------------------------------------------------------------------------------------------------------------------------------------------YECRTLLFKAIHNLLERCLMNR---------------------------GFVRIGKWFVKPY-------------------------QKEE-------K-----S--INK----------------------------SEHLSCAFTFFVHGD--------SNVCTSVEIA-QHQPLQRLSE---EHLSLAQ----------------------------------------------------------QSSSPLQVILSPYGLNGTLTGQA-----------------------------------------------------------FKMSDHPTQKLIEEWRQ-FYPISPNPKE-------------------------------------------------------------------------------------------------------------------------VPEDKME-DSDWEDDSLAAVEVLV-AG----------------------------------------------VRMVYPSCLVLLPLT     139948785       PNGASLEDCHSNLFCL-------------------------------------ADLTGIKWKCFVWQGPTSS---PILFPVT---------------------------------EEDPILCSF-SRCLKA---------------DVLSVWRRHQTP--------------------------------------------GRRELWIFWWGDD------------------------------------------------------------------PNFAELVHHD-----------------------------------------------------------------------------------------------------------------------LSCNEDGSWES---GLT-----------------------------------------------------------------------------------------------------------------------------------------------------------------YECRTLLFKAIHNLLERCLMNR---------------------------SFVRIGKWFVKPY-------------------------EKDE-------K-----P--INK----------------------------SEHLSCSFTFFVHGD--------SNVCTSVEIN-QHQPVYLLSE---EHLTLAQ----------------------------------------------------------QSSSSVQVILSPYGLSGTLTGQS-----------------------------------------------------------FKLSDPPTQKLIEEWKQ-FYPIGPNTKE-------------------------------------------------------------------------------------------------------------------------VTDDKMD-DLDWEDDSLAAVEVVV-AG----------------------------------------------VRMVYPASLVLVAQS     410914654       PNGASLEDCHSNLFCL-------------------------------------ADLTGIKWKRFVWQGPTSA---PMLFPVT---------------------------------EEDPILCSF-SRCLKA---------------DVLCVWRRSQRQ--------------------------------------------GRRELWMFWWGDD------------------------------------------------------------------PNFADLIHHE-----------------------------------------------------------------------------------------------------------------------LAAEDDGLWEN---GLS-----------------------------------------------------------------------------------------------------------------------------------------------------------------YECRTLLFKAIHNLLERCLMNR---------------------------SFVRIGKWFVKPY-------------------------EKDE-------K-----P--INK----------------------------SEHLSCAFTFFLHGD--------SNVCTSVEVN-QHQPVYHLTE---EHLTLAQ----------------------------------------------------------QSSSPFQVILSPFGLNGTLTGQS-----------------------------------------------------------FKMSDPPTQKLIEEWNQ-FYPISSKAKE------------------------------------------------------------------------------------------------------------------------GVSEDKLE-DMDWEDDSLASVEVLV-GG----------------------------------------------VRMVYPACLVLVPQS     4530437         PNGASLEDCHCNLFCL-------------------------------------ADLTGIKWKKYVWQGPTSA---PILFPVT---------------------------------EEDPILSSF-SRCLKA---------------DVLGVWRRDQRP--------------------------------------------GRRELWIFWWGED------------------------------------------------------------------PVLLTLFTMT-----------------------------------------------------------------------------------------------------------------------YQKKKMECGRM---DFP-----------------------------------------------------------------------------------------------------------------------------------------------------------------MNAVLCFSKAVHNLLERCLMNR---------------------------NFVRIGKWFVKPY-------------------------EKDE-------K-----P--INK----------------------------SEHLSCSFTFFLHGD--------SNVCTSVEIN-QHQPVYLLSE---EHITLAQ----------------------------------------------------------QSNSPFQVILCPFGLNGTLTGQA-----------------------------------------------------------FKMSDSATKKLIGEWKQ-FYPISCCLKE-------------------------------------------------------------------------------------------------------------------------MSEEKQE-DMDWEDDSLAAVEVLV-AG----------------------------------------------VRMIYPACFVLVPQS     326931507       SVLEKLPARDREFYNR-----------------------EAVNFSEIAEEWQNADLTGIKWKRYVWQGPTSA---PILFPVT---------------------------------EEDPILSSF-SRCLKA---------------DVLSVWRRDQRP--------------------------------------------GRRELWIFWWGDD------------------------------------------------------------------PNFADLIHHD-----------------------------------------------------------------------------------------------------------------------LSEEEDGVWEN---GLS-----------------------------------------------------------------------------------------------------------------------------------------------------------------YECRTLLFKAVHNLLERCLMNR---------------------------NFVRIGKWFVKPY-------------------------EKDE-------K-----P--INK----------------------------SEHLSCSFTFFLHGD--------SNVCTSVEIS-QHQPVYLLSE---EHLTLAQ----------------------------------------------------------QSNSPFQVILSPFGLNGTLTGQS-----------------------------------------------------------FKLSDSSTKKLIGEWKQ-FYPVTSNLKE-------------------------------------------------------------------------------------------------------------------------GSEEKQE-DMDWEDDSLAAVEVLV-AG----------------------------------------------VRMVYPACFVLVPQT     301608632       PNGASLEDCHSNLFCL-------------------------------------ADLTGIKWKRYIWQGPTSA---PILFPVT---------------------------------EEDPILSSF-SRCLKA---------------DVLSVWRRDERP--------------------------------------------GRRELWIFWWGED------------------------------------------------------------------PNFADLIHHD-----------------------------------------------------------------------------------------------------------------------LADEEDGIWDN---GLS-----------------------------------------------------------------------------------------------------------------------------------------------------------------YECRTLLFKAIHNLLERCLMNR---------------------------NFVRIGKWFVKPY-------------------------EKDE-------K-----P--VNK----------------------------SEHLSCSFAFFLHGD--------SNVCTSVEIN-QHQPVYLLSE---EHLTLAQ----------------------------------------------------------QSNSPFQVILSPFGLNGTLTGQS-----------------------------------------------------------FKMSDSSTKKLIGEWKQ-FYPISSSVKE-------------------------------------------------------------------------------------------------------------------------CSEEKQE-DMDWEDDSLAAVEVLV----------------------------------------------------------------     224076629       PNGASLEDCHSNLFCL-------------------------------------ADLTGIKWKRYVWQGPTSA---PILFPVT---------------------------------EEDPILSSF-SRCLKA---------------DVLSVWRRDQRP--------------------------------------------GRRELWIFWWGDD------------------------------------------------------------------PNFADLIHHD-----------------------------------------------------------------------------------------------------------------------LSEEEDGVWEN---GLS-----------------------------------------------------------------------------------------------------------------------------------------------------------------YECRTLLFKAVHNLLERCLMNR---------------------------NFVRIGKWFVKPY-------------------------EKDE-------K-----P--INK----------------------------SEHLSCSFTFFLHGD--------SNVCTSVEIS-QHQPVYLLSE---EHLTLAQ----------------------------------------------------------QSNNPFQVILSPFGLNGTLTGQS-----------------------------------------------------------FKLSDSSTKKLIGEWKQ-FYPVTSNLKE-------------------------------------------------------------------------------------------------------------------------GSEEKQE-EMDWEDDSLAAVEVLV-AG----------------------------------------------VRMVYPACFVLVPQT     363741191       PNGASLEDCHSNLFCL-------------------------------------ADLTGIKWKRYVWQGPTSA---PILFPVT---------------------------------EEDPILSSF-SRCLKA---------------DVLSVWRRDQRP--------------------------------------------GRRELWIFWWGDD------------------------------------------------------------------PNFADLIHHD-----------------------------------------------------------------------------------------------------------------------LSEEEDGVWEN---GLS-----------------------------------------------------------------------------------------------------------------------------------------------------------------YECRTLLFKAVHNLLERCLMNR---------------------------NFVRIGKWFVKPY-------------------------EKDE-------K-----P--INK----------------------------SEHLSCSFTFFLHGD--------SNVCTSVEIS-QHQPVYLLSE---EHLTLAQ----------------------------------------------------------QSNSPFQVILSPFGLNGTLTGQS-----------------------------------------------------------FKLSDSSTKKLIGEWKQ-FYPVTSNLKE-------------------------------------------------------------------------------------------------------------------------GSEEKQE-DMDWEDDSLAAVEVLV-AG----------------------------------------------VRMVYPACFVLVPQT     395531834       PRPPLAASCRSSLASR--------------------------------PFSPLADLTGIKWKRYVWQGPTSA---PILFPVT---------------------------------EEDPILSSF-SRCLKA---------------DVLGVWRRDQRP--------------------------------------------GRRELWIFWWGED------------------------------------------------------------------PNFADLIHHD-----------------------------------------------------------------------------------------------------------------------LAEEEDGVWEN---GLS-----------------------------------------------------------------------------------------------------------------------------------------------------------------YECRTLLFKAVHNLLERCLMNR---------------------------NFVRIGKWFVKPY-------------------------EKDE-------K-----P--INK----------------------------SEHLSCSFTFFLHGD--------SNVCTSVEIN-QHQPVYLLSE---EHITLAQ----------------------------------------------------------QSNNPFQVILSPFGLNGTLTGQA-----------------------------------------------------------FKMSDSSTKKLIGEWKQ-FYPISSCLKE-------------------------------------------------------------------------------------------------------------------------VSEEKQE-DMDWEDDSLAAVEVLV-AG----------------------------------------------VRMIYPACFVLVPQS     124286862       SNGASLEDCHCNLFCL-------------------------------------ADLTGIKWKRYVWQGPTSA---PILFPVT---------------------------------EEDPILSSF-SRCLKA---------------DVLGVWRRDQRP--------------------------------------------GRRELWIFWWGKD------------------------------------------------------------------PNFADLIHHD-----------------------------------------------------------------------------------------------------------------------LSEEEDGVWEN---GLS-----------------------------------------------------------------------------------------------------------------------------------------------------------------YECRTLLFKAVHNLLERCLMNR---------------------------NFVRIGKWFVKPY-------------------------EKDE-------K-----P--INK----------------------------SEHLSCSFTFFLHGD--------SNVCTSVEIN-QHQPVYLLSE---EHVTLAQ----------------------------------------------------------QSNSPFQVILSPFGLNGTLTGQA-----------------------------------------------------------FKMSDSATKKLIGEWKQ-FYPISCGLKE-------------------------------------------------------------------------------------------------------------------------MSEEKQD-DMDWEDDSLAAVEVLV-AG----------------------------------------------VRMIYPACFVLVPQS     348567424       LLGLRTSPLPGPAAR--------------------------------------ADLTGIKWKRYVWQGPTSA---PILFPVT---------------------------------EEDPILSSF-SRCLKA---------------DVLSVWRRDQRP--------------------------------------------GRRELWIFWWGED------------------------------------------------------------------PNFADLIHHD-----------------------------------------------------------------------------------------------------------------------LSEEEDGVWEN---GLS-----------------------------------------------------------------------------------------------------------------------------------------------------------------YECRTLLFKAVHNLLERCLMNR---------------------------NFVRIGKWFVKPY-------------------------EKDE-------K-----P--INK----------------------------SEHLSCSFTFFLHGD--------SNVCTSVEIN-QHQPVYLLSE---EHITLAQ----------------------------------------------------------QSNSPFQVILSPFGLNGTLTGQA-----------------------------------------------------------FKMSDSATKKLIGEWKQ-FYPISCCLKE-------------------------------------------------------------------------------------------------------------------------MSEEKQE-DMDWEDDSLAAVEVLV-AG----------------------------------------------VRMIYPACFVLVPQS     351714843       --------------------------------------------------FLQADLTGIKWKRYVWQGPTSA---PILFPVT---------------------------------EEDPILSSF-SRCLKA---------------DVLSVWRRDQRP--------------------------------------------GRRELWIFWWGED------------------------------------------------------------------PNFADLIHHD-----------------------------------------------------------------------------------------------------------------------LSEEEDGVWEN---GLS-----------------------------------------------------------------------------------------------------------------------------------------------------------------YECRTLLFKAVHNLLERCLMNR---------------------------NFVRIGKWFVKPY-------------------------EKDE-------K-----P--VNK----------------------------SEHLSCSFTFFLHGD--------SNVCTSVEIN-QHQSVYLLSE---EHITLAQ----------------------------------------------------------QSNSPFQVILSPFGLNGTLTGQA-----------------------------------------------------------FKMSDSATKKLIGEWKQ-FYPISCCLKE-------------------------------------------------------------------------------------------------------------------------MSEEKQE-DMDWEDDSLAAVEVLV-AG----------------------------------------------VRMIYPACFVLVPQS     194217212       -MFAVRCPLITISSDV-------------------------------------ADLTGIKWKRYVWQGPTSA---PILFPVT---------------------------------EEDPILSSF-SRCLKA---------------DVLGVWRRDQRP--------------------------------------------GRRELWIFWWGED------------------------------------------------------------------PNFADLIHHD-----------------------------------------------------------------------------------------------------------------------LSEEEDGVWEN---GLS-----------------------------------------------------------------------------------------------------------------------------------------------------------------YECRTLLFKAVHNLLERCLMNR---------------------------NFVRIGKWFVKPY-------------------------EKDE-------K-----P--INK----------------------------SEHLSCSFTFFLHGD--------SNVCTSVEIN-QHQPVYLLSE---EHITLAQ----------------------------------------------------------QSNSPFQVILSPFGLNGTLTGQA-----------------------------------------------------------FKMSDSATKKLIGEWKQ-FYPISSCLKE-------------------------------------------------------------------------------------------------------------------------MSEEKQE-DMDWEDDSLAAVEVLV-AG----------------------------------------------VRMIYPACFVLVPQS     194675789       PRPPSRAALAASAFSP-------------------------------------ADLTGIKWKRYVWQGPTSA---PILFPVT---------------------------------EEDPILSSF-SRCLKA---------------DVLGVWRRDQRP--------------------------------------------GRRELWIFWWGED------------------------------------------------------------------PNFADLIHHD-----------------------------------------------------------------------------------------------------------------------LSEEEDGVWEN---GLS-----------------------------------------------------------------------------------------------------------------------------------------------------------------YECRTLLFKAVHNLLERCLMNR---------------------------NFVRIGKWFVKPY-------------------------EKDE-------K-----P--INK----------------------------SEHLSCSFTFFLHGD--------SNVCTSVEIN-QHQPVYLLSE---EHITLAQ----------------------------------------------------------QSNSPFQVILSPFGLNGTLTGQA-----------------------------------------------------------FKMSDSATKKLIGEWKQ-FYPISSCLKE-------------------------------------------------------------------------------------------------------------------------MSEEKQE-DMDWEDDSLAAVEVLV-AG----------------------------------------------VRMIYPACFVLVPQS     345805272       RDQLEQNLEEMFTECA-------------------------------------ADLTGIKWKRYVWQGPTSA---PILFPVT---------------------------------EEDPILSSF-SRCLKA---------------DVLGVWRRDQRP--------------------------------------------GRRELWIFWWGED------------------------------------------------------------------PNFADLIHHD-----------------------------------------------------------------------------------------------------------------------LSEEEDGVWEN---GLS-----------------------------------------------------------------------------------------------------------------------------------------------------------------YECRTLLFKAVHNLLERCLMNR---------------------------NFVRIGKWFVKPY-------------------------EKDE-------K-----P--INK----------------------------SEHLSCSFTFFLHGD--------SNVCTSVEIN-QHQPVYLLSE---EHITLAQ----------------------------------------------------------QSNSPFQVILSPFGLNGTLTGQA-----------------------------------------------------------FKMSDSATKKLIGEWKQ-FYPISSCLKE-------------------------------------------------------------------------------------------------------------------------MSEEKQE-DMDWEDDSLAAVEVLV-AG----------------------------------------------VRMIYPACFVLVPQS     297486414       -----------MAVVL-------------------------------------ADLTGIKWKRYVWQGPTSA---PILFPVT---------------------------------EEDPILSSF-SRCLKA---------------DVLGVWRRDQRP--------------------------------------------GRRELWIFWWGED------------------------------------------------------------------PNFADLIHHD-----------------------------------------------------------------------------------------------------------------------LSEEEDGVWEN---GLS-----------------------------------------------------------------------------------------------------------------------------------------------------------------YECRTLLFKAVHNLLERCLMNR---------------------------NFVRIGKWFVKPY-------------------------EKDE-------K-----P--INK----------------------------SEHLSCSFTFFLHGD--------SNVCTSVEIN-QHQPVYLLSE---EHITLAQ----------------------------------------------------------QSNSPFQVILSPFGLNGTLTGQA-----------------------------------------------------------FKMSDSATKKLIGEWKQ-FYPISSCLKE-------------------------------------------------------------------------------------------------------------------------MSEEKQE-DMDWEDDSLAAVEVLV-AG----------------------------------------------VRMIYPACFVLVPQS     291405670       GDGVRSPAVPGSVLG--------------------------------------ADLTGIKWKRYVWQGPTSA---PILFPVT---------------------------------EEDPILSSF-SRCLKA---------------DVLGVWRRDQRP--------------------------------------------GRRELWIFWWGED------------------------------------------------------------------PNFADLIHHD-----------------------------------------------------------------------------------------------------------------------LSEEEDGVWEN---GLS-----------------------------------------------------------------------------------------------------------
[truncated: 346,962 more chars]
